# Synthesis of rare-earth borolide sandwich complexes by multi-electron borole reduction

**DOI:** 10.1039/d6sc05163d

**Published:** 2026-08-27

**Authors:** Xiao-Han Peng, Tao Shang, Yan-Cong Chen, Arpan Mondal, Ming Liu, Yu-Han Du, Ying Liu, Ming-Liang Tong, Richard A. Layfield, Fu-Sheng Guo

**Affiliations:** a Institute of Fundamental and Frontier Sciences, University of Electronic Science and Technology of China Xiyuan Avenue 2006 Chengdu 611731 China guofush@hotmail.com; b Key Laboratory of Bioinorganic and Synthetic Chemistry of the Ministry of Education, School of Chemistry, IGCME, GBRCE for Functional Molecular Engineering, Sun Yat-Sen University Guangzhou 510006 China tongml@mail.sysu.edu.cn; c Department of Chemistry, School of Life Sciences, University of Sussex Brighton BN1 9QR UK r.layfield@sussex.ac.uk

## Abstract

The limited redox chemistry of rare-earth (RE) elements means that multi-electron transfer reactions with RE compounds are a major synthetic challenge. Here, we demonstrate multi-electron reduction of a borole in two complementary RE reaction systems. In the first approach, the dimetallic lanthanum(iii) compound [(*η*^5^-C_5_^*i*^Pr_5_)La(*µ*:*η*^6^:*η*^6^-C_6_H_6_)La(*η*^5^-C_5_^*i*^Pr_5_)], containing a benzene tetra-anion ligand, reduces two equivalents of the ferrocenyl–borole FcBC_2_(SiMe_3_)_2_C_2_Me_2_ (1) to give the double sandwich complex [{(C_5_^*i*^Pr_5_)RE}FcBC_2_(SiMe_3_)_2_C_2_Me_2_] (3-RE, RE = La), containing a 6π-aromatic borolide di-anion. In the second, complementary reaction type, combining KC_8_ with 1 and the half-sandwich complexes [(C_5_^*i*^Pr_5_)RE(BH_4_)_2_(THF)] (RE = Y, La, Dy) also gives 3-RE but with a broader range of rare-earth elements. In 3-RE, evidence for two-electron reduction of the borole is supported by comparisons with the magnesium borolide [Mg(FcBC_2_(SiMe_3_)_2_C_2_Me_2_)(THF)_3_] (2). In contrast, salt-metathesis approaches to 3-RE were ineffective, with alkali metal reduction of the borole proving to be unsuccessful, and 2 does not undergo salt metathesis with rare-earth precursors. The dysprosium analogue 3-Dy exhibits single-molecule magnet behaviour, with an effective barrier of 1222(8) cm^−1^ and magnetic hysteresis up to 60 K.

## Abstract 2

Two synthetic strategies enable two-electron reduction of an antiaromatic borole to give rare-earth borolide sandwich complexes inaccessible by salt metathesis. The dysprosium complex is an SMM with *U*_eff_ = 1222 cm^−1^ and hysteresis up to 60 K.

## Graphical abstract

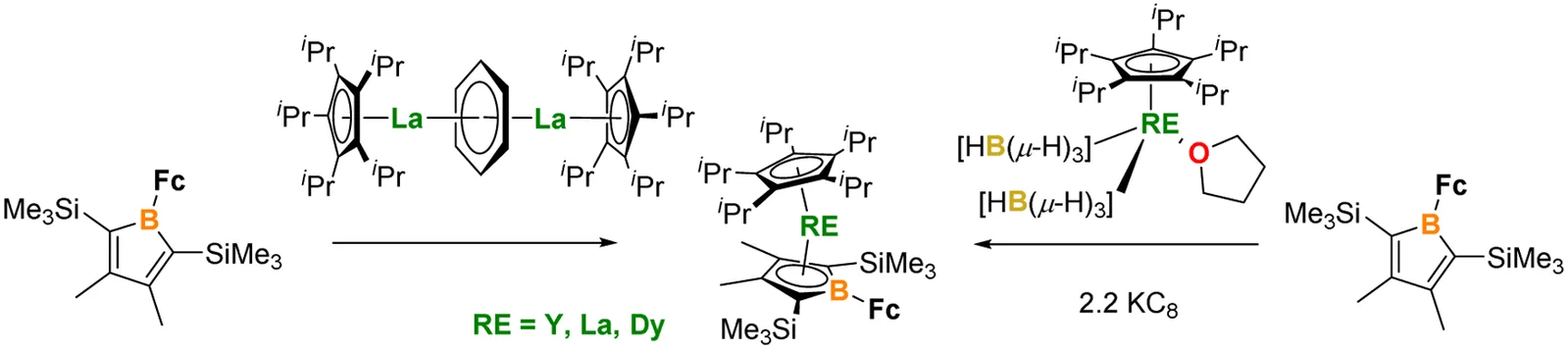

## Introduction

Divalent rare-earth (RE) compounds have extensive applications in organic synthesis,^1^ small-molecule activation,^2,3^ and catalysis,^4^ and the europium(iii)/(ii) redox couple has recently been used to enhance the stability of perovskite solar cells.^5^ The potential applications of divalent RE compounds as molecular spin qubits and single-molecule magnets (SMMs) have also begun to attract attention.^6–8^ In contrast, well-defined monovalent RE compounds have evaded capture,^9^ hence RE-based redox chemistry is effectively limited to one-electron transfer. To achieve multi-electron transfer in RE chemistry, an alternative approach in which redox-active ligands store and release electrons is an attractive alternative.^10–23^ For instance, Mazzanti *et al.* recently reported a series of ytterbium complexes supported by a tripodal *tris*(siloxide)arene ligand, in which the metal adopts well-defined +3 and +2 oxidation states but also exhibits reactivity consistent with lower formal oxidation states, enabling two-electron transfer processes.^14^ In related studies, trivalent RE elements have shown a remarkable propensity to stabilize highly reduced benzene anions, including bimetallic inverse sandwich complexes of 10π-aromatic benzene tetra-anions.^16–22^ Huang *et al.* reported that such compounds can reduce unsaturated organic molecules, with concomitant arene elimination,^17^ showing that trivalent RE compounds of arene tetra-anions can act as effective multi-electron reductants.

Previously, some of us reported the triple-decker benzene tetra-anion compounds [(*η*^5^-C_5_^*i*^Pr_5_)RE(*µ*:*η*^6^:*η*^6^-C_6_H_6_)RE(*η*^5^-C_5_^*i*^Pr_5_)] for the trivalent rare-earth elements Y, La, Sm, Gd, and Dy.^21,22^ Crystallographic studies, theoretical calculations, and magnetic susceptibility measurements confirmed the presence of a benzene tetra-anion ligand and its 10π-aromaticity. Given the reducing ability of benzene tetra-anions and the strong axial ligand field of [C_5_^*i*^Pr_5_]^−^, we envisioned that [(C_5_^*i*^Pr_5_)RE(*µ*:*η*^6^:*η*^6^-C_6_H_6_)RE(C_5_^*i*^Pr_5_)] could serve as multi-electron reductants towards electron-deficient organic molecules, transferring the {(C_5_^*i*^Pr_5_)RE} units into new sandwich compounds and thereby providing a novel route to SMMs. To test this idea, we identified neutral, five-membered, 4π-antiaromatic boroles, with the vacant boron 2p_*z*_ orbital serving as a two-electron acceptor to give 6π-aromatic borolide dianions bound to the rare-earth metal.^24–26^ Beyond the synthetic advance, borolide dianions have also been shown to provide a strong axial ligand field to RE ions,^27–31^ suggesting that our approach could provide a complementary method for the synthesis of borolide-ligated SMMs. Based on these considerations, we aimed to obtain heteroleptic RE sandwich compounds *via* two-electron reduction of a borole, and to study the slow magnetic relaxation properties of the dysprosium version.

## Results and discussion

**Scheme 1:**
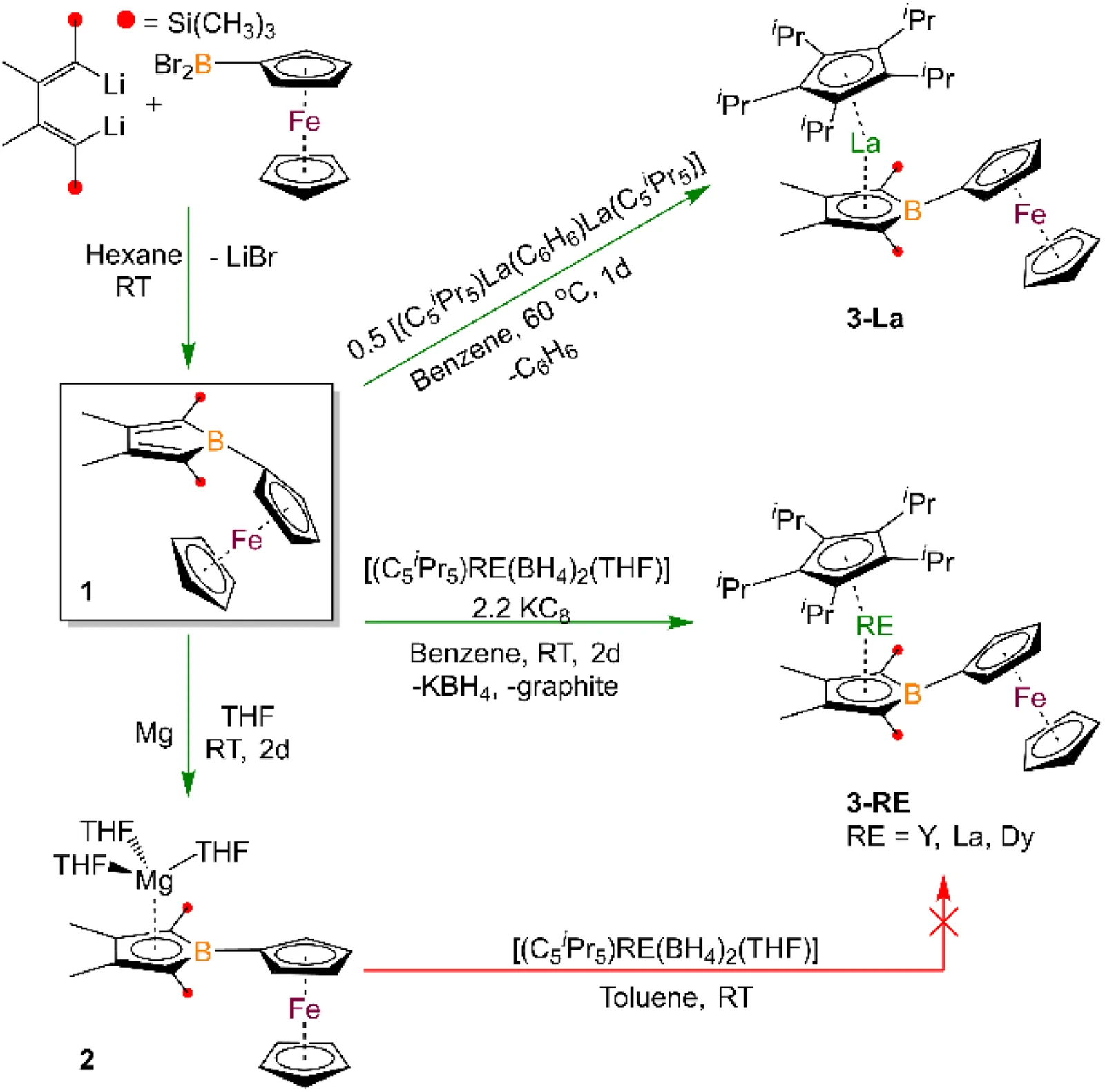
Synthesis of FcBC_2_(SiMe_3_)_2_C_2_Me_2_ (1), [Mg(FcBC_2_(SiMe_3_)_2_C_2_Me_2_)(THF)_3_] (2) and [(C_5_^*i*^Pr_5_)RE(FcBC_2_(SiMe_3_)_2_C_2_Me_2_)] (3-RE, RE = Y, La, Dy). Red crosses denote unsuccessful reactions.

Our first aim was to design and synthesize a new borole compound,^25^ hence we targeted the ferrocenyl–borole, FcBC_2_(SiMe_3_)_2_C_2_Me_2_ (1) (Scheme 1). The rationale for the choice of 1 is its relative ease of synthesis, with the ferrocenyl substituent providing some steric protection and aiding crystallization of the products. Salt elimination reactions of [Fc(BBr_2_)] and 1,4-dilithio-1,4-bis(trimethylsilyl)-2,3-dimethyl-1,3-butadiene in hexane at room temperature produced 1 as dark red crystals in 47% isolated yield. Next, we attempted to isolate the di-sodium or di-potassium salts of the dianion [FcBC_2_(SiMe_3_)_2_C_2_Me_2_]^2−^ using the approach employed previously by some of us for [(Me_2_N)BC_4_Et_4_]^2−^.^30^ Despite a rapid colour change from deep orange to dark red upon mixing the reagents in THF, only intractable oils were obtained. This may be caused by excessive side reactions, including the migration of [CpFe]^+^ from the Cp anion to the borole dianion stimulated by further reduction,^26^ as well as the ring-opening of THF.

Next, magnesium was used to reduce 1, allowing the piano–stool complex [Mg(FcBC_2_(SiMe_3_)_2_C_2_Me_2_)(THF)_3_] (2) to be isolated (Scheme 1). The successful synthesis of 2 can be attributed to the reduced steric hindrance in this complex compared to the nominal alkali metal complexes, which would need to coordinate to both π-faces of the borole dianion. However, the salt elimination reaction between 2 and [(C_5_^*i*^Pr_5_)RE(BH_4_)_2_THF] in toluene also does not proceed, possibly due to stronger binding of Mg^2+^ to the borolide ligand. These results highlight the limitations of salt elimination reactions in certain systems, underscoring the importance of exploring novel reactions.

Following the unsuccessful attempts to synthesize RE borole complexes using salt metathesis chemistry, we used an alternative route with [(C_5_^*i*^Pr_5_)La(C_6_H_6_)La(C_5_^*i*^Pr_5_)] as the reductant. Reacting two equivalents of 1 with one of [(C_5_^*i*^Pr_5_)La(C_6_H_6_)La(C_5_^*i*^Pr_5_)] in hot benzene for 24 hours produced the double sandwich compound [(C_5_^*i*^Pr_5_)La(FcBC_2_(SiMe_3_)_2_C_2_Me_2_)] (3-La) (Scheme 1). After removal of the solvent *in vacuo*, the residual solid was extracted into toluene, filtered and crystallized by storing the solution at −35 °C, yielding pure 3-La as dark purple crystals. Diamagnetic 3-La was characterized by NMR spectroscopy (Fig. S15–18). All resonances in the ^1^H, ^13^C{^1^H} and ^29^Si{^1^H} NMR spectrum can be assigned to the [C_5_^*i*^Pr_5_]^−^ ligand and borole dianion. The ^11^B{^1^H} NMR spectrum of 3-La (Fig. S17) features a single broad resonance at *δ* = 49.3 ppm, which is shifted upfield by 12 ppm relative to 1 (Fig. S8) owing to the reduced nature of the boron center in 3-La.^26,32–34^ Notably, the ^11^B{^1^H} NMR signal of 3-La shows a significant downfield shift compared to that of 2 (*δ* = 33.8 ppm) (Fig. S12). The reaction of 1 with [(C_5_^*i*^Pr_5_)La(C_6_H_6_)La(C_5_^*i*^Pr_5_)] was investigated by ^1^H NMR spectroscopy at room temperature and at 60 °C (Fig. S20 and 21). After mixing the two materials at room temperature for 24 hours, the formation of 3-La was confirmed by the presence of signals at *δ* = 0.56 ppm (Fig. S20), which correspond to the SiMe_3_ resonances of 3-La. Heating at 60 °C accelerates the reaction rate, with the signals from [(C_5_^*i*^Pr_5_)La(C_6_H_6_)La(C_5_^*i*^Pr_5_)] disappearing after 24 hours (Fig. S22).

Surprisingly, the approach used to synthesize 3-La does not work for dysprosium. Given the similar ionic radii of dysprosium(iii) and yttrium(iii), the reaction of [(C_5_^*i*^Pr_5_)Y(C_6_H_6_)Y(C_5_^*i*^Pr_5_)] with 1 was monitored with ^1^H NMR spectroscopy. Whether at room temperature or after heating at 60 °C for up to twenty days, the ^1^H NMR spectrum of this reaction shows virtually no change, displaying only the signals of the two starting materials (Fig. S27–29). Considering the only major difference between La^3+^ and Y^3+^/Dy^3+^ is the larger radius of the former, the contrasting behaviour of this limited selection of rare earths is presumably a size effect. An alternative approach to 3-Y and 3-Dy was therefore pursued.

To synthesize [(C_5_^*i*^Pr_5_)Dy(C_6_H_6_)Dy(C_5_^*i*^Pr_5_)],^22^ two methods were developed. One method involves the reaction of [(C_5_^*i*^Pr_5_)Dy(Cp*)(BH_4_)] with an excess of KC_8_, which, with short reaction times, produces the divalent dysprosium compound [(C_5_^*i*^Pr_5_)Dy(Cp*)],^22^ indicating the involvement of reduced dysprosium species under the reaction conditions. Thus, the reactions of [(C_5_^*i*^Pr_5_)RE(BH_4_)_2_THF] (RE = Y, La, Dy), 1, and 2.2 equivalents of KC_8_ in benzene at room temperature were attempted (Scheme 1), giving the target compounds [(C_5_^*i*^Pr_5_)RE(FcBC_2_(SiMe_3_)_2_C_2_Me_2_)] (3-RE, RE = Y, La, Dy). The formation of the borolide sandwich complexes 3-RE*via* this method is consistent with two-electron reduction of 1.

**Fig. 1:**
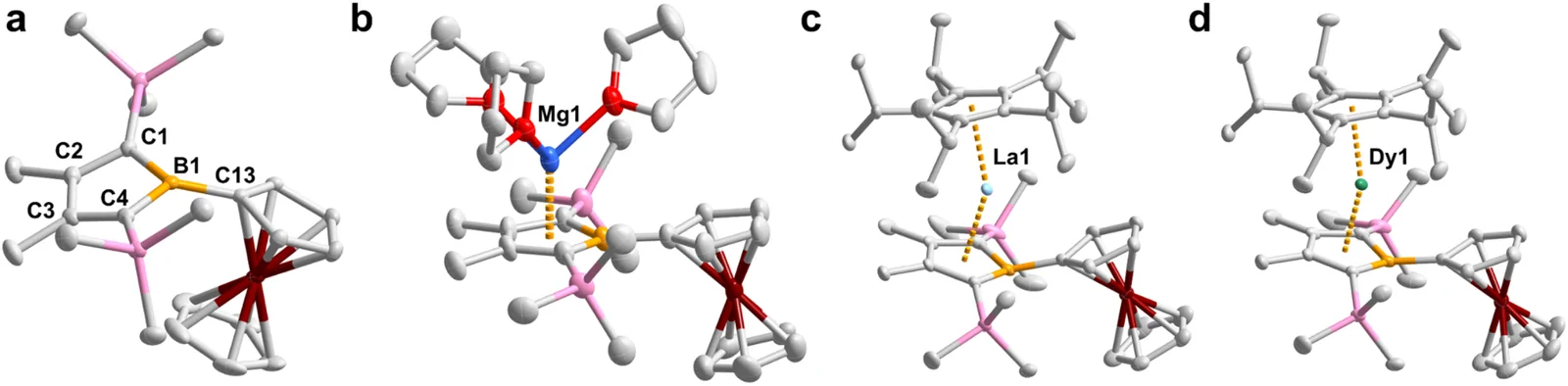
Thermal ellipsoid representation (50% probability) of the molecular structures of compounds 1 (a), 2 (b), 3-La (c) and 3-Dy (d). Hydrogen atoms were omitted for clarity. C: grey; O: red; B: orange; Si: rose; Fe: dark red; Mg: blue; La: pale blue; Dy: green.

**Scheme 2:**
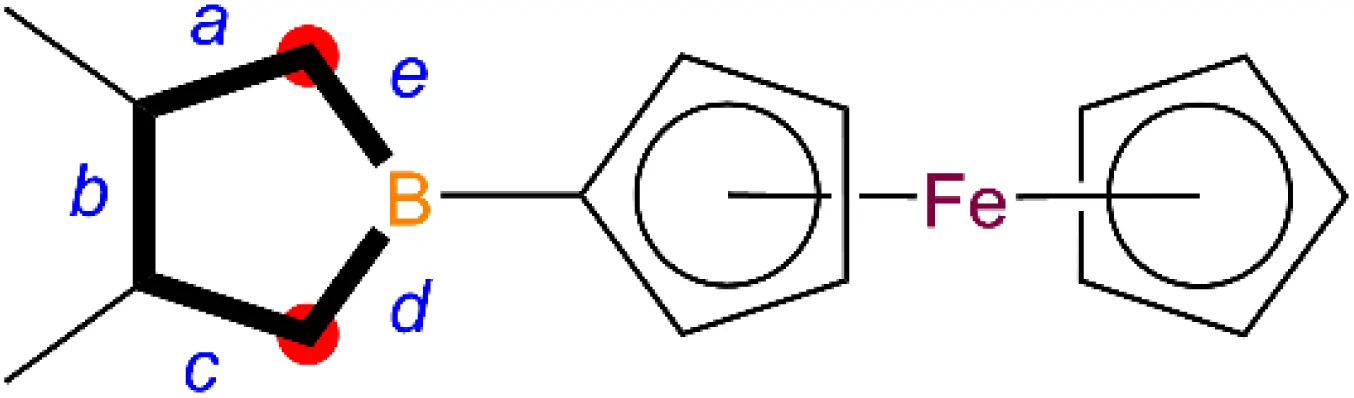
Bonds a–d and e of the C_4_B rings in 1, 2, and 3-RE.

Single crystal X-ray structure analysis reveals that compound 1 crystallizes in the triclinic *P*-1 space group (Table S1), featuring a ferrocenyl-substituted borole ring (Fig. 1a and S30). Steric interactions between the ferrocenyl and SiMe_3_ groups result in a structural distortion, with the boron atom residing 0.298(2) Å out of the C1–C2–C3–C4 mean plane. Furthermore, the dihedral angle defined by C1–B1–C4 and C1–C2–C3–C4 planes is 18.37(13)°. The C_4_B ring in 1 exhibits the characteristic structural features of an antiaromatic system, as evidenced by pronounced alternation of C=C and C–C/C–B bond lengths (C1–C2: 1.353(2) Å; C2–C3: 1.517(2) Å; C3–C4: 1.358(2) Å; C4–B1: (C1–C2: 1.353(2) Å; C2–C3: 1.517(2) Å; C3–C4: 1.358(2) Å; C4–B1: 1.594(2) Å; B1–C1: 1.590(2) Å) (Scheme 2 and Table 1). The B1–C13 bond to the ferrocenyl substituent is 1.540(2) Å, which is longer than the corresponding bond (average 1.521 Å) in the related ferrocenylborole FcBC_4_Ph_4_,^25^ confirms the higher steric hindrance in 1.

**Table 1:** Selected bond lengths [Å] of C_4_B rings in compounds 1, 2, and 3-RE. Bond labels refer to the structure shown in Scheme 2

	Bond a/Å	Bond b/Å	Bond c/Å	Bond d/Å	Bond e/Å
1	1.353(2)	1.517(2)	1.358(2)	1.594(2)	1.590(2)
2	1.462(5)/1.459(5)	1.392(4)/1.393(7)	1.471(6)/1.476(6)	1.558(5)/1.549(6)	1.565(5)/1.557(6)
3-La	1.449(2)	1.408(2)	1.454(2)	1.547(2)	1.558(2)
3-Dy	1.460(3)	1.404(3)	1.459(3)	1.546(3)	1.565(3)
3-Y	1.458(4)	1.402(4)	1.455(4)	1.561(3)	1.547(4)

The magnesium borolide 2 crystallizes in the triclinic space group *P*-1 (Table S1) with two independent molecules in the asymmetric unit. The C_4_B ring is planar, and nearly coplanar with the adjacent Cp ligand (Fig. 1b and S31). The magnesium ion is capped by the *η*^5^-C_4_B ring, and the coordination environment is completed by three THF ligands. The average B–C bond lengths in the C_4_B ring of 2 decrease by 0.035 Å to 1.557 Å relative to 1.592 Å in 1, and the bond alternation in the butadiene group is much less pronounced (Scheme 2 and Table 1). These bond changes support the 6π-aromatic character of the C_4_B ring in 2. Noting that two-electron reduction of boroles with magnesium has been reported by Braunschweig *et al.*,^26^ compound 2 does not react with [(C_5_^*i*^Pr_5_)RE(BH_4_)_2_THF], rendering it ineffective for accessing the target complexes. Instead, 2 is a valuable structural benchmark for the borole dianion, enabling direct comparison with 3-RE and supporting assignment of two-electron reduction of the ligand.

Compounds 3-RE (RE = Y, La, Dy) are isostructural (Table S2, Fig. 1c, d, S32 and 33), consistent with their similar FTIR spectra (Fig. S3–5). Hence, only 3-La is described in detail. The double sandwich compound 3-La crystallizes in the monoclinic *P*2_1_/*n* space group, with one sandwich unit consisting of lanthanum(iii) bound by one [*η*^5^-C_5_^*i*^Pr_5_]^−^ anion and one *η*^5^-borolide dianion. The disordered C_5_^*i*^Pr_5_ ligand was refined assuming that the isopropyl groups are disordered over two opposite orientations in a ratio of 0.745 : 0.255. Compared to 2, the bulky [*η*^5^-C_5_^*i*^Pr_5_]^−^ ligand in 3-La forces the two SiMe_3_ substituents on the borole ring to bend more markedly below the C_4_B plane, with the ferrocenyl substituent then forced to bend above the plane (Fig. S32). The borole dianion exhibits similar aromatic characteristics to that in 2, with comparable bond lengths (Table 1). The La–(C_5_) and La–(C_4_B) bond lengths are in the range 2.7572(15)–2.8916(16) Å and 2.6571(17)–2.7719(15) Å, respectively (Table S5). The La–(C_5_)_cent_ and La–(C_4_B)_cent_ distances are 2.5562(8) and 2.4067(8) Å, respectively, resulting in a wide (C_5_)_cent_–La–(C_4_B)_cent_ angle of 152.22(3)°. For comparison, the Dy–(C_5_)_cent_ and Dy–(C_4_B)_cent_ distances, and the (C_5_)_cent_–Dy–(C_4_B)_cent_ angles in 3-Dy (Table S6) are 2.3653(10) Å, 2.2403(10)Å, and 157.34(4)°, respectively. The shorter distances and the wider bending angle in the dysprosium sandwich component of 3-Dy compared to 3-La can be attributed to the smaller radius of the heavier RE element. A similar trend was found for 3-Y (Table S7), and the corresponding parameters are 2.3570(12) Å, 2.2300(12)Å, and 157.68(5)°, respectively.

In addition to the above characteristics, a noticeable structural distortion in the C_4_B ring is apparent. Specifically, as the metal ion radius decreases according to the lanthanide contraction, the distance between boron and the C_4_ mean plane increases in the order 0.032(2) Å, 0.068(3) Å and 0.078(4) Å for 3-La, 3-Dy, and 3-Y, respectively.

To account for the formation of 3-RE, we note that comparable studies in which rare-earth cyclopentadienyl complexes act as multi-electron reductants are uncommon. Building on the four-electron transfer reaction of 
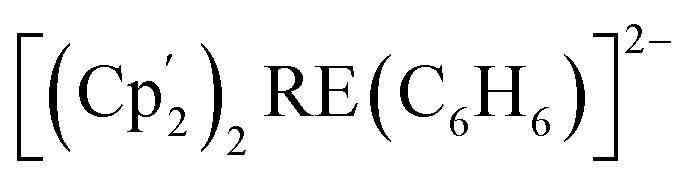
 towards two equivalents of naphthalene to give 
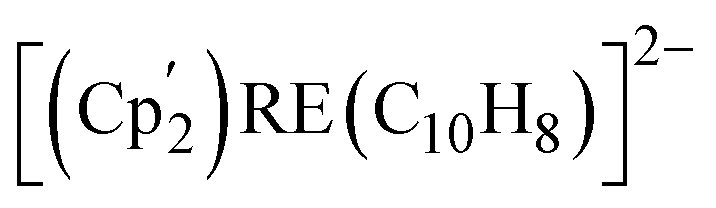
 (RE = La, Ce; Cp′ = C_5_H_4_SiMe_3_),^35^ three-electron reduction of Cp*Fe(P_5_) was shown to generate the heterobimetallic complexes 
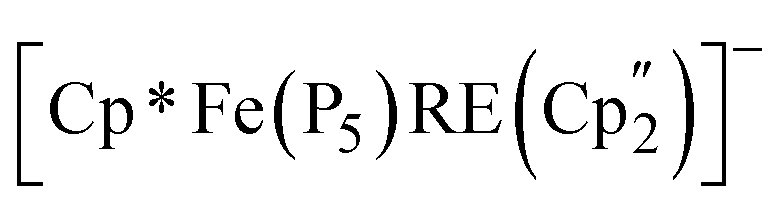
 (RE = Ce, Nd; Cp″ = 1,3-C_5_H_3_(SiMe_3_)_2_).^36^ Recently, multi-electron reduction reactivity was also reported for benzene tetra-anion complexes of samarium(iii) with supporting β-diketiminate ligands.^17,37^ For example, the benzene tetra-anion complex [{(^DIPeP^BDI)Sm}_2_(*µ*-C_6_H_6_)} (^DIPeP^BDI = HC[C(Me)N-DIPeP]_2_, DIPeP = 2,6-CHEt_2_-phenyl) reacts with two equivalents of cyclo-octatetraene (COT) to give the half-sandwich complex [(^DIPeP^BDI)Sm(*η*^8^-COT)].^37^ Our findings extend this valuable reactivity concept to the transfer of half-sandwich RE building blocks and, in the case of dysprosium, utilize the reactivity to target the synthesis of a high-performance SMM (see below).

### Computational studies

To gain more insight into the aromaticity and electronic structures of 1, 2, 3-La and 3-Y, density functional theory (DFT) calculations were carried out (see SI for computational details). Nucleus-independent chemical shift (NICS) calculations were performed to judge the aromaticity of the C_4_B rings. Normally, positive values indicate anti-aromaticity, while negative values indicate aromaticity.^38^ The NICS(1)_*ZZ*_ value is defined as the negative value of the shielding tensor component perpendicular to the ring plane, measured at 1 Å above it. The centre of mass for the C_4_B group was located using the least-squares method within Multiwfn.^39^ The NICS(1)_*ZZ*_ value of the C_4_B ring in 1 was calculated to be +18.94 ppm, reflecting antiaromatic character. In contrast, the aromaticity of the 6π-electron C_4_B rings in 2, 3-La and 3-Y is indicated by their negative NICS(1)_*ZZ*_ values of −17.05, −18.20 and −15.95 ppm, respectively. The slight reduction in aromaticity of the C_4_B ring from 3-La to 3-Y may be attributed to the aforementioned structural distortions caused by the relative sizes of the metal ions.

**Fig. 2:**
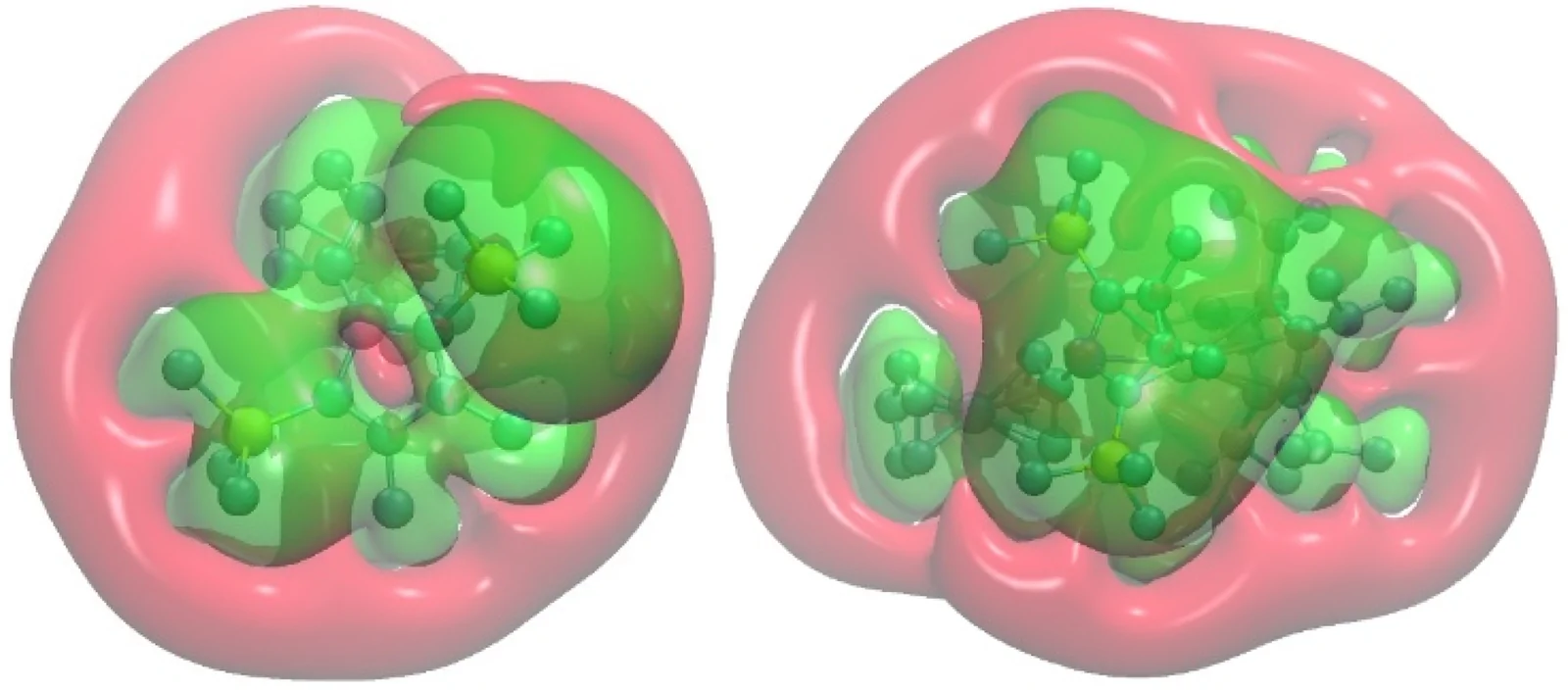
Isosurface maps of ICSS_*zz*_ for 1 (left) and 3-La (right). The green isosurface is a shielding area and the red isosurface is a deshielding area.

To visualize the magnetic shielding and deshielding areas in three-dimensional space, the iso-chemical shielding surface (ICSS) method was also employed.^40^ The *ZZ* component of the ICSS (ICSS_*ZZ*_)^41^ for compounds 1, 2, 3-La and 3-Y are presented in Fig. 2 and S82–85. There is a clear deshielding (red) region in the direction perpendicular to the C_4_B ring in 1. In contrast, the ICSS_*ZZ*_ maps of compound 2, 3-La and 3-Y exhibit strong magnetically shielded areas (green) surrounded by deshielding loops (red), confirming the 6π-aromaticity.

**Fig. 3:**
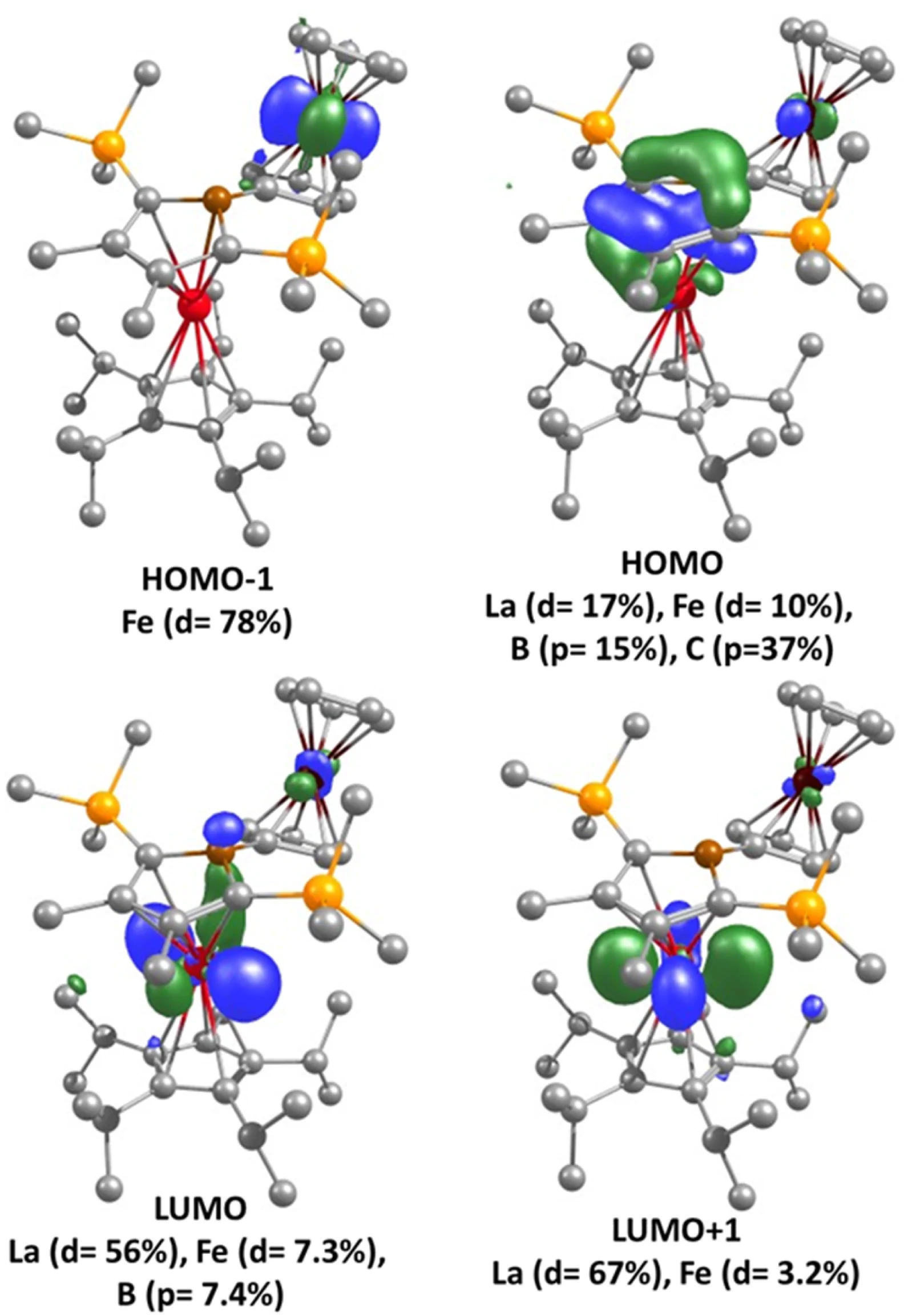
DFT-calculated frontier MOs and orbital compositions (only from La, Fe and borolide ligand) for 3-La (isovalue = 0.057).

The calculated frontier MOs (molecular orbitals) for 3-La and 3-Y correspond to the RE-C_4_B interactions and are similar (Fig. 3, S86-87). Their HOMOs (highest-occupied MOs) are dominated by the borolide 2p orbitals (52% for 3-La; 45% for 3-Y), with additional contributions from iron 3d orbitals (10% for 3-La; 20% for 3-Y) and lanthanum 5d (17%) or yttrium 4d (14%) orbitals. In contrast, the LUMOs (lowest-unoccupied MOs) are predominantly localized on the RE centres, with minimal contributions from the boron 2p and iron 3d orbitals.

**Fig. 4:**
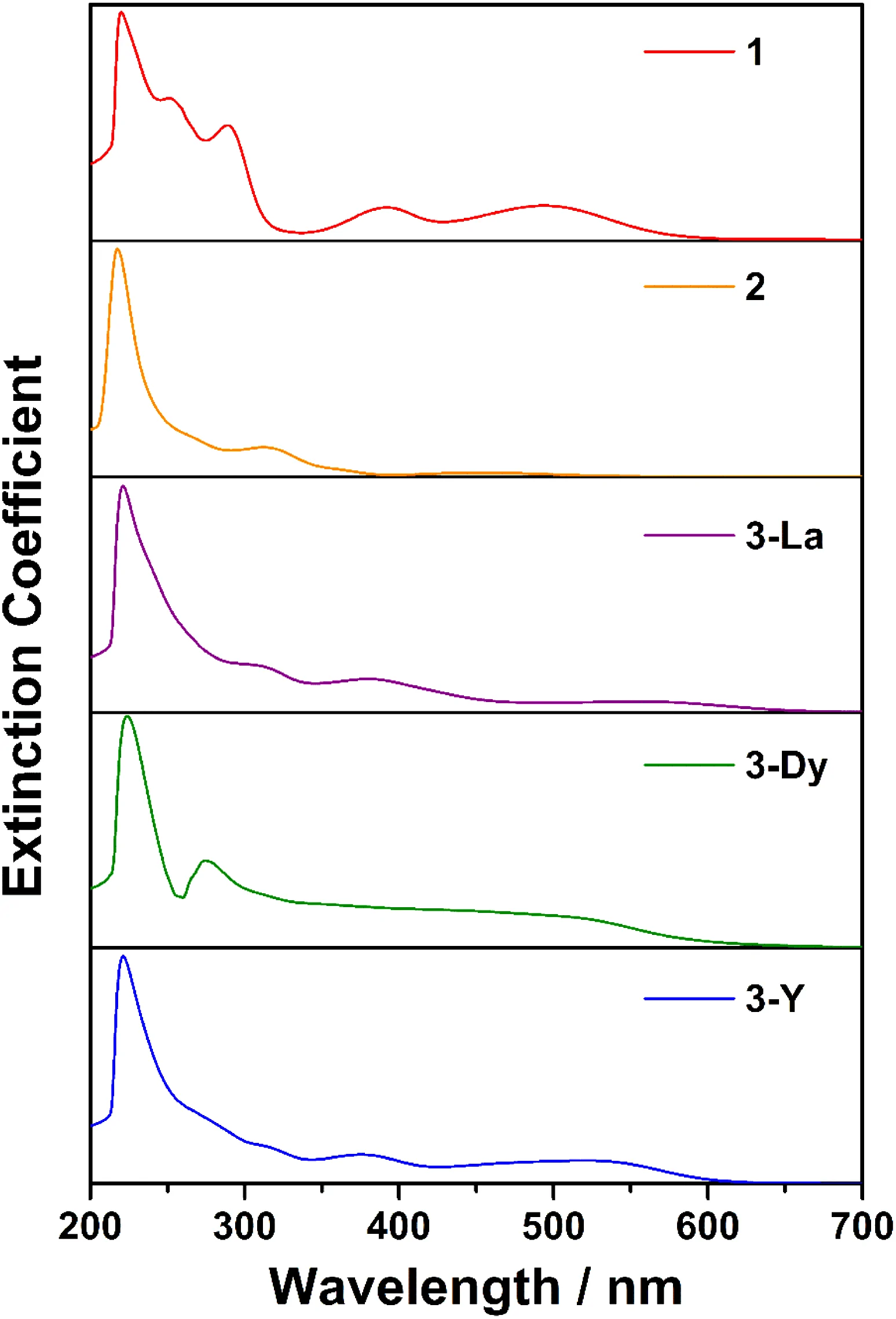
UV-vis-NIR spectrum of compounds 1 (hexane), 2 (THF), and 3-RE (hexane). The corresponding molar absorptivities are shown in Fig. S57–61.

The electronic structures of 1, 2, 3-La and 3-Y were investigated further using UV-vis-NIR spectroscopy (Fig. 4, S34-61) and time-dependent DFT calculations. For compound 1 (Fig. S90, 91 and Table S11), the assignments are as follows. The high-energy absorption at 221 nm is assigned to the HOMO − 2 → LUMO + 3 (borole to iron) transition calculated at 232 nm. The intermediate-energy absorptions at 251 and 289 nm originate from the lower-lying orbitals to the LUMO and LUMO + 1 transitions calculated at 245–306 nm. The low-energy absorptions at 391 and 494 nm are attributed to transitions from an iron-based 3d HOMO to the LUMO with both iron 3d and borole character, and the HOMO − 1 also with iron 3d character to the LUMO calculated at 463 and 525 nm, respectively. For compound 2 (Fig. S92, 93 and Table S12), the spectrum features a major absorption at 217 nm, assigned to the HOMO and HOMO − 1 to the LUMO + 3 (borolide to ferrocenyl) and other higher-lying MOs transitions calculated at 236 nm. The broad absorption spanning 300–400 nm corresponds to the HOMO and lower-lying MOs to the LUMO and higher-lying MOs transitions calculated at 298–419 nm. Another broad absorption in the 420–500 nm range is attributed to the HOMO and other lower-lying MOs to the LUMO and LUMO + 1 transitions calculated at 468 and 474 nm.

For compound 3-La (Fig. S88, S89 and Table S13), the high-energy absorption at 220 nm primarily corresponds to the HOMO − 8 → LUMO + 1 (ferrocenyl to lanthanum 5d) transitions calculated at 248 nm. The intermediate-energy broad absorption spanning 300–400 nm corresponds to transitions from the borolide-based HOMO and lower-lying MOs to the lanthanum-based LUMO + 1 and higher-lying MOs. The low-energy absorption at 552 nm is attributed to the HOMO to lanthanum-based LUMO transition calculated at 530 nm. The UV-vis-NIR spectrum of 3-Y is similar to that of 3-La, showing a major absorption at 220 nm with minor absorptions at 314, 376 and 521 nm. The UV-vis-NIR spectrum of 3-Dy exhibits a strong absorption at 226 nm, comparable to the intense band observed for the lanthanum and yttrium analogues. In addition, a well-resolved band at 275 nm is present for 3-Dy, likely corresponding to features that appear as weak shoulders in the spectra of 3-La and 3-Y but are partially obscured by the intense high-energy absorptions. The spectrum of 3-Dy also displays shoulder-like features in the lower-energy region.

### Magnetic properties

**Fig. 5:**
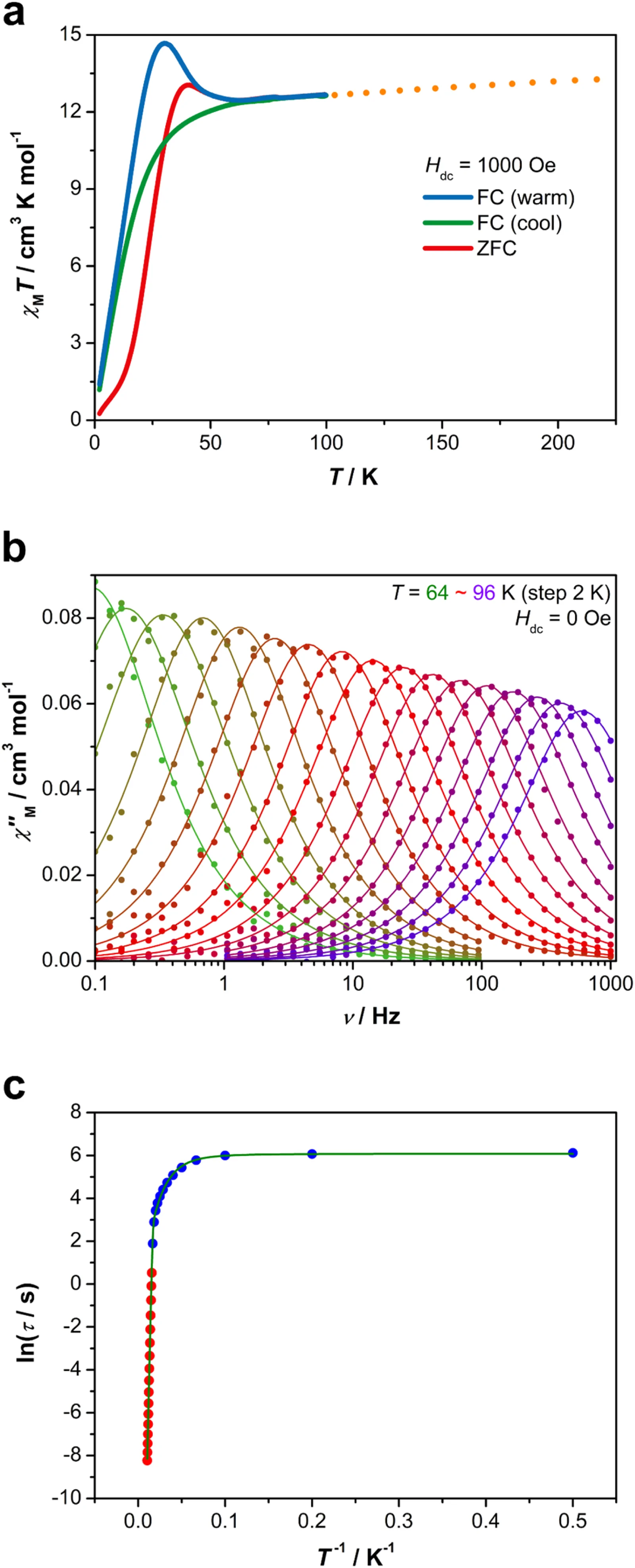
(a) Temperature-dependent DC magnetic susceptibility data for compound 3-Dy. (b) Frequency dependence of *χ*″ in zero DC field in the temperature range of 64–92 K. Solid lines represent fits to the data using a generalized Debye equation. (c) Plot of the natural log of the relaxation time *versus* 1/*T* for 3-Dy. The red dots represent data extracted from the Cole–Cole plots. The blue dots represent data obtained from fitting to the magnetization decay data. The green line represents the fit as described in the text.

The near-linear pseudo-2-coordinate nature of 3-Dy meets the basic requirements for a high-performance SMM.^27–30,42–53^ Specifically, the oblate Dy^3+^ ion is sandwiched between two anionic ligands and should experience a strong axial crystal field. The static and dynamic magnetic properties of 3-Dy were therefore examined. Variable-temperature direct current (DC) magnetic susceptibility data were collected upon warming the sample from 100 to 217 K in an applied field of 1000 Oe (Fig. 5a). The sample was restrained in Fomblin oil with a pour point of 238 K hence, with an abundance of caution, data were not collected above 217 K.^7^ The *χ*_M_*T* value (*χ*_M_ is the molar magnetic susceptibility) is 12.66 cm^3^ K mol^−1^ at 100 K, which increases slowly as the temperature rises, reaching 13.27 cm^3^ K mol^−1^ at 217 K. Magnetic blocking was identified through field-cooled (FC) and zero-field-cooled (ZFC) variable-temperature (2 K min^−1^) magnetic susceptibility measurements under an applied field of 1000 Oe in the range 2–100 K, (Fig. 5a and S62). The FC-ZFC bifurcation was observed at approximately 60 K in warming and cooling modes, suggesting prominent magnetic blocking.

The alternating current (AC) magnetic susceptibility was measured in zero DC field at frequencies of 0.1–999 Hz to determine the relaxation rate (Fig. 5b and S63–65). The frequency-dependent out-of-phase (the imaginary component, *χ*″) curves show a single set of well-defined maxima in the temperature range of 64–96 K (Fig. 5b and S66–68). The maxima shift to higher frequencies with increasing temperature, consistent with SMM behaviour. The magnetic relaxation time (*τ*) at each temperature (Table S8) can thus be extracted from the Cole–Cole plots of *χ*″ *vs. χ*′ (Fig. S66–68) using the generalized Debye model.^54^ The resulting *α* parameters fall within the range of 0.01–0.06, indicating a very narrow range of relaxation times.

As seen in Fig. 5b, the magnetic relaxation below 64 K is too slow (lower than 0.1 Hz) to be detected by the AC measurement. Hence, to obtain the relaxation times at lower temperatures, DC magnetic relaxation measurements were conducted (Fig. S69–81) and the relaxation times extracted from the magnetization decay data (Table S9). The natural log of the combined relaxation time *versus T*^−1^ is presented in Fig. 5c. In the high-temperature range, the curve shows a strong linear dependence on temperature, corresponding to the Orbach process. As the temperature decreases, the dependence gradually becomes curved over a narrow temperature range, and then becomes temperature independent, indicating Raman and then quantum tunnelling of the magnetization (QTM) processes, respectively. Therefore, the data were fitted by the equation *τ*^−1^ = *τ*_0_^−1^exp(−*U*_eff_/*k*_B_*T*) + *CT*^*n*^ + *τ*_QTM_^−1^, (where *τ*_0_, *U*_eff_, *k*_B_, *C* and *n*, are the attempt time, effective spin-reversal barrier, Boltzmann constant, the Raman coefficient and the Raman exponent, respectively, and *τ*_QTM_^−1^ is the rate of QTM). This analysis gave *U*_eff_ = 1222(8) cm^−1^, *τ*_0_ = 4.55(1) × 10^−12^ s, *C* = 1.49(1) × 10^−7^ s^−1^ K^−*n*^, *n* = 3.1(1) and *τ*_QTM_ = 435(19) s. Based on the magnetization decay measurements, the 100-second blocking temperature (*T*_B(100s)_) is 30 K. The Raman exponent of *n* = 3.1(1) is significantly lower than expected for a conventional Raman process in a Kramers ion, suggesting the involvement of low-energy optical phonons or other localized vibrational modes.^42,50^ The small Raman coefficient also indicates relatively weak spin-phonon coupling.

**Fig. 6:**
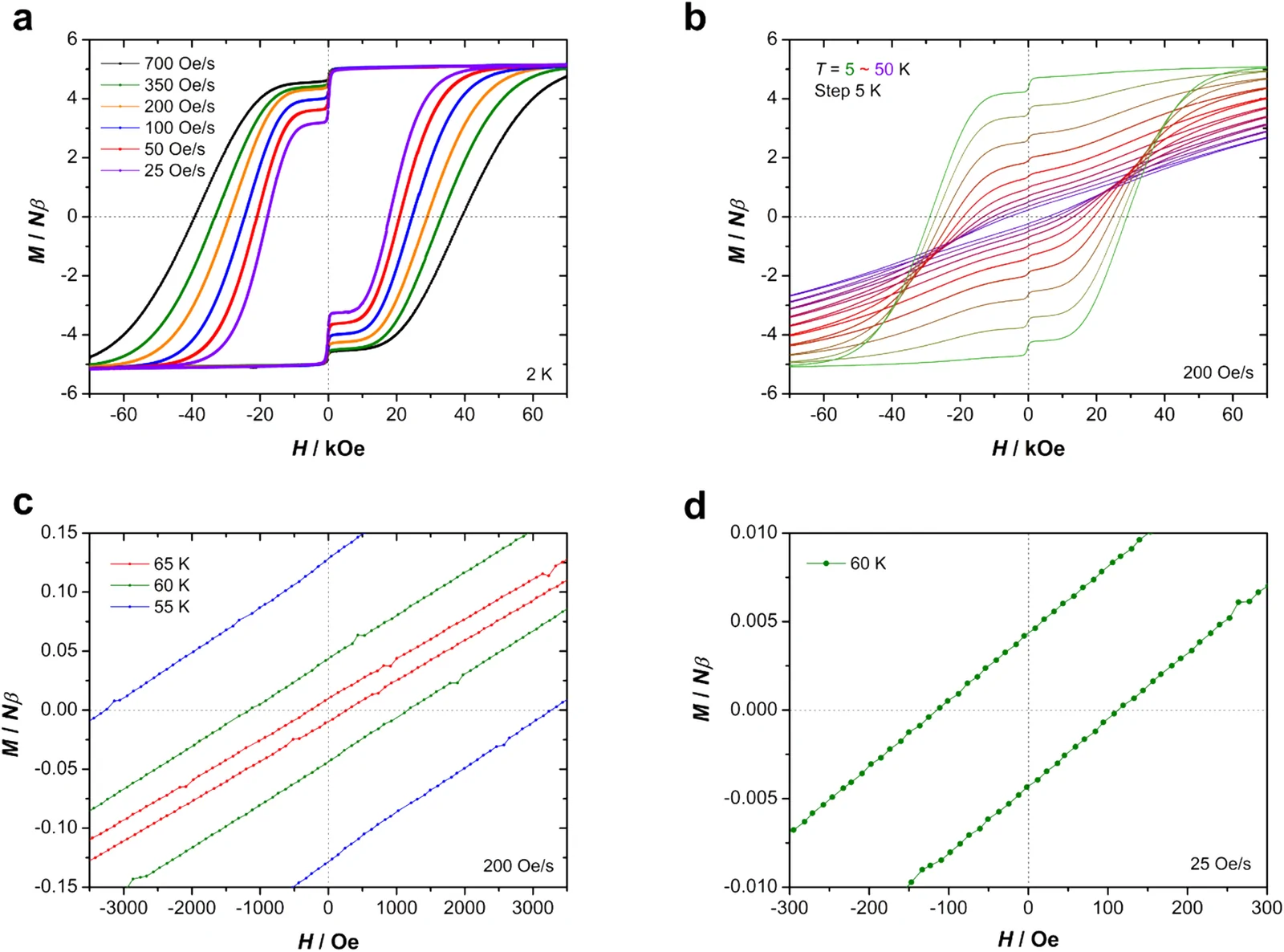
(a) Magnetic hysteresis loops for 3-Dy at 2 K under different sweep rates. (b) Magnetic hysteresis loops for 3-Dy in the temperature range of 5 to 50 K using a sweep rate of 200 Oe s^−1^. (c) Enlargement of the magnetic hysteresis loops for 3-Dy in the temperature range of 55 to 65 K using a sweep rate of 200 Oe s^−1^. (d) Enlargement of the magnetic hysteresis loops for 3-Dy at 60 K using a sweep rate of 25 Oe s^−1^.

Open hysteresis loops are an important characteristic of an SMM. At 2 K, under the maximum field of *H* = 70 kOe achievable with our magnetometer, the magnetization (*M*) saturates at 5.36 Nβ. As seen Fig. 6a, the *M*(*H*) hysteresis loops at 2 K under different sweep rates show significant openings. The coercive field (*H*_c_) and magnetic remanence (*M*_r_) of the hysteresis loop exhibit a positive correlation with the sweep rate (Table S10). For instance, the measured *H*_c_ and *M*_r_ values at 25 Oe s^−1^ are 17 857 Oe and 3.816 Nβ, respectively, increasing to 39 085 Oe and 4.639 Nβ at 700 Oe s^−1^. The steps around zero field are attributable to QTM, which is a common feature in SMMs and may be driven by hyperfine interactions with spin-active dysprosium nuclei.^55,56^ At a field sweep rate of 200 Oe s^−1^, the hysteresis loops gradually close from 2 to 65 K, accompanied by gradual reductions of *H*_c_ and *M*_r_ from 29 104 Oe and 4.542 Nβ to 237 Oe and 0.011 Nβ, respectively (Fig. 6 and Table S10). Using a lower field sweep rate of 25 Oe s^−1^, the hysteresis loop at 60 K is still open, with a coercive field of 111 Oe, indicating that the blocking temperature of hysteresis is approximately 60 K, in good agreement with the FC-ZFC susceptibility.

### Multireference calculations

**Fig. 7:**
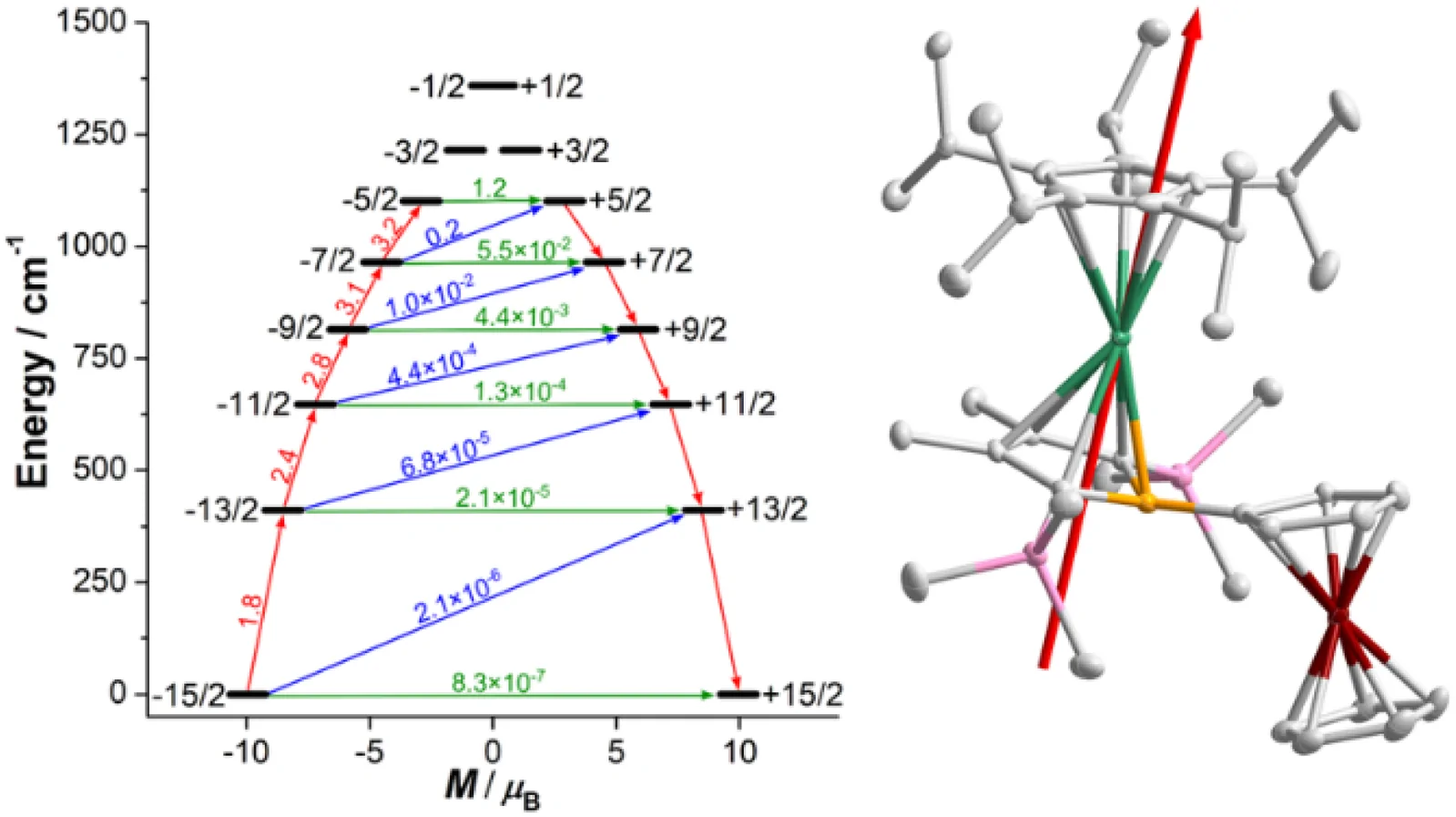
(left) Calculated magnetic relaxation barrier for 3-Dy. The black lines represent the KD energies as a function of magnetic moment along the magnetic axis. Green lines correspond to QTM. Blue lines represent off-diagonal relaxation. Numbers at each arrow are mean absolute values of the corresponding matrix element of transition magnetic moment. (right) Calculated orientation of the main magnetic axis in the ground KD.

To shed light onto the magnetic anisotropy and relaxation processes in 3-Dy, complete-active-space self-consistent field (CASSCF) calculation on the basis of X-ray determined geometry has been carried out with OpenMolcas^57^ and SINGLE_ANISO programs (see SI for details).^58–60^ The energy levels (cm^−1^), *g* (*g*_*x*_, *g*_*y*_, *g*_*z*_) tensors and the predominant *m*_J_ values of the lowest eight Kramers doublets (KDs) of 3-Dy are listed in Tables S15 and S16. The ground KD is predominantly the ±15/2 state (99.8%), with *g*-values approaching the Ising limits, *i.e.*, *g*_*x*_ = *g*_*y*_ ≈ 0 and *g*_*z*_ = 19.89. The main magnetic axis of the ground KD of 3-Dy is shown in Fig. 7. The anisotropy axis is nearly perpendicular to the center of the borolide ligand, highlighting a dominant axial crystal field. All higher-lying KDs exhibit magnetic anisotropy axes collinear with the ground KD until the fifth doublet, where a non-negligible component of *m*_J_ = ±1/2 (6.6%) is mixed with ±5/2 state (90.5%), leading to a large transverse magnetic moment. Hence, the expected energy barrier of 3-Dy is 1102 cm^−1^, which is within 10% of the experimental *U*_eff_, with relaxation most likely occurring through the fifth-excited KD.

The calculated crystal-field parameters provide further insight into the magnetic anisotropy of 3-Dy. The crystal field is strongly dominated by the axial B^0^_2_ parameter (60% of the total parameter weight, Table S17), consistent with the strong axial anisotropy of the Dy^3+^ centre. However, appreciable non-axial contributions are present, most notably the *B*_2_^2^ parameter (6.5%), together with other terms of higher rank. Although relatively small, these transverse components can promote mixing between states and thereby facilitate magnetic relaxation *via* a lower-lying KD.

Strongly axial crystal fields combined with weak equatorial crystal fields have proven to be a reliable blueprint for producing high-performance dysprosium SMMs, an approach that can significantly stabilize the *m*_J_ = ±15/2 ground doublet relative to the higher-lying doublets.^45^ Beyond traditional cyclopentadienyl ligands, heteroatom-containing alternatives have also accounted for some impressive advances.^52,53^ In the case of borolide-type ligands, for example, dysprosium *bis*(borolide) complexes have been shown to exhibit barriers to magnetization reversal in the region of 1500–1600 cm^−1^ and magnetic blocking temperatures of *ca.* 65 K, placing them amongst the best-performing systems.^27–30^ In this context, the magnetic behaviour of 3-Dy, with a *U*_eff_ value of 1222 cm^−1^ and magnetic blocking temperature of 60 K fits well within the emerging family of metallocene-like lanthanide SMMs based on hetero-cyclopentadienyl donor ligands. The primary role of the boron atom in 3-Dy is to enable a stronger axial crystal field, which is manifested through the formal di-anionic charge of the borolide ligand, which provides a highly electron-rich π-donor environment. The higher barriers in the *bis*(borolide) SMMs can therefore be attributed to the effects of two dianionic borolide ligands, which provide a stronger axial crystal field.

Relative to 3-Dy, the closely related SMM [Dy(*η*^5^-BC_4_Ph_5_)(*η*^5^-C_5_^*i*^Pr_5_)] displays a larger effective barrier of 1536(15) cm^−1^ despite being structurally very similar, with only minor differences in the key geometric parameters.^29^ Since the borolide substituents represent the principal difference between these two heteroleptic SMMs, the different barriers may reflect substituent-dependent changes in the crystal field and/or vibrational dynamics of the complexes. Differences in the effective charge distribution across the borolide ligand could modify the crystal-field splitting, while changes in ligand rigidity could alter spin-phonon coupling and hence the relaxation dynamics.

Whilst the ferrocenyl unit does not directly participate in the coordination sphere of dysprosium and is therefore not expected to significantly influence the magnetism, its redox-activity could potentially be used to modulate the electronic structure of the lanthanide. Further investigation of possible redox-switchable SMM behaviour is under investigation.

## Conclusions

Through two-electron reduction of the anti-aromatic borole 1 using complementary synthetic routes, the double RE sandwich compounds 3-RE (RE = La, Dy, Y) were isolated and fully characterized. The inability to obtain alkali metal salts of the borole and of the magnesium salt 2 to undergo salt elimination reactions highlights the importance of this complementary synthetic approach. The anti-aromaticity of 1, and the aromaticity of 2, 3-La and 3-Y are supported by X-ray structural analysis, NMR spectroscopy and theoretical calculations. The structural characteristics of 3-Dy give rise to excellent SMM performance, including a large energy barrier of 1222(8) cm^−1^ and with multireference calculations revealing that the dominant thermal relaxation process occurs *via* the fifth excited Kramers doublet. Magnetic hysteresis measurements on 3-Dy reveal open loops, with coercivity, up to 60 K, indicating that the mixed borole-cyclopentadienyl ligand system is effective at supporting slow magnetic relaxation, which may potentially be tuneable *via* modification to the boron substituent. This work demonstrates that reductive synthetic approaches can provide access to rare-earth sandwich compounds that are not accessible *via* conventional salt metathesis.

## Author contributions

X.-H. P. synthesized and characterized the compounds. T. S., A. M. and Y. L. performed the theoretical calculations. Y.-H. D. and M. L. assisted in experiments and synthesized some compounds. Y.-C. C. and M.-L. T. performed SQUID magnetometry measurements. F.-S. G. and M.-L. T. acquired funding. F.-S. G., M.-L. T. and R. A. L. supervised the study. X.-H. P., T. S., F.-S. G. and R. A. L. wrote the manuscript with input from all the authors.

## Conflicts of interest

There are no conflicts to declare.

## Supplementary Material

SC-OLF-D6SC05163D-s001

SC-OLF-D6SC05163D-s002

## Data Availability

Additional research data supporting this publication are available at DOI: 10.25377/sussex.32743809. CCDC 2523312–2523316 contain the supplementary crystallographic data for this paper.^61*a*–*e*^ Supplementary information (SI) is available. See DOI: https://doi.org/10.1039/d6sc05163d.

## References

[cit1] Szostak M., Fazakerley N. J., Parmar D., Procter D. J. (2014). Chem. Rev..

[cit2] Evans W. J., Fang M., Zucchi G., Furche F., Ziller J. W., Hoekstra R. M., Zink J. I. (2009). J. Am. Chem. Soc..

[cit3] Papangelis E., Demonti L., del Rosal I., Shephard A., Maron L., Nocton G., Simler T. (2025). J. Am. Chem. Soc..

[cit4] Allouche F., Chan K. W., Fedorov A., Andersen R. A., Copéret C. (2018). Angew. Chem., Int. Ed..

[cit5] Wang L., Zhou H., Hu J., Huang B., Sun M., Dong B., Zheng G., Huang Y., Chen Y., Li L., Xu Z., Li N., Liu Z., Chen Q., Sun L.-D., Yan C.-H. (2019). Science.

[cit6] Smith P. W., Hrubý J., Evans W. J., Hill S., Minasian S. G. (2024). J. Am. Chem. Soc..

[cit7] Kundu K., White J. R. K., Moehring S. A., Yu J. M., Ziller J. W., Furche F., Evans W. J., Hill S. (2022). Nat. Chem..

[cit8] Liu M., Chen Y.-C., Wang H., Shang T., Tong M.-L., Layfield R. A., Mansikkamäki A., Guo F.-S. (2025). J. Am. Chem. Soc..

[cit9] Arnold P. L., Cloke F. G. N., Hitchcock P. B., Nixon J. F. (1996). J. Am. Chem. Soc..

[cit10] Camp C., Guidal V., Biswas B., Pécaut J., Dubois L., Mazzanti M. (2012). Chem. Sci..

[cit11] Fedushkin I. L., Lukoyanov A. N., Baranov E. V. (2018). Inorg. Chem..

[cit12] Mondal A., Tang J., Layfield R. A. (2025). Angew. Chem., Int. Ed..

[cit13] Mondal A., Price C. G. T., Tang J., Layfield R. A. (2023). J. Am. Chem. Soc..

[cit14] Tricoire M., Sroka W., Rajeshkumar T., Scopelliti R., Sienkiewicz A., Maron L., Mazzanti M. (2025). J. Am. Chem. Soc..

[cit15] Luevano M. R., Stennett C. R., Ma E., Ziller J. W., Furche F., Evans W. J. (2025). J. Am. Chem. Soc..

[cit16] Huang W., Dulong F., Wu T., Khan S. I., Miller J. T., Cantat T., Diaconescu P. L. (2013). Nat. Commun..

[cit17] Wang Y., Zhang Y., Liang J., Tan B., Deng C., Huang W. (2024). Chem. Sci..

[cit18] Thakur S. K., Roig N., Monreal-Corona R., Langer J., Alonso M., Harder S. (2024). Angew. Chem., Int. Ed..

[cit19] Jin P.-B., Luo Q.-C., Gransbury G. K., Winpenny R. E. P., Mills D. P., Zheng Y.-Z. (2025). Chem. Sci..

[cit20] Richardson G. M., Rajeshkumar T., Burke F. M., Cameron S. A., Nicholls B. D., Harvey J. E., Keyzers R. A., Butler T., Granville S., Liu L., Langley J., Lim L. F., Cox N., Chilton N. F., Hicks J., Davis N. J. L. K., Maron L., Anker M. D. (2025). Nat. Chem..

[cit21] McClain K. R., Vincent A. H., Rajabi A., Ngo D. X., Meihaus K. R., Furche F., Harvey B. G., Long J. R. (2024). J. Am. Chem. Soc..

[cit22] Liu M., Chen Y.-C., Mondal A., Wang H., Tong M.-L., Layfield R. A., Guo F.-S. (2025). J. Am. Chem. Soc..

[cit23] Mondal A., Rajeshkumar T., Steiner A., Tang J., Maron L., Layfield R. A. (2026). J. Am. Chem. Soc..

[cit24] Eisch J. J., Galle J. E., Kozima S. (1986). J. Am. Chem. Soc..

[cit25] Braunschweig H., Fernández I., Frenking G., Kupfer T. (2008). Angew. Chem., Int. Ed..

[cit26] Braunschweig H., Chiu C. W., Gamon D., Kaupp M., Krummenacher I., Kupfer T., Müller R., Radacki K. (2012). Chem. – Eur. J..

[cit27] Vanjak J. C., Wilkins B. O., Vieru V., Bhuvanesh N. S., Reibenspies J. H., Martin C. D., Chibotaru L. F., Nippe M. (2022). J. Am. Chem. Soc..

[cit28] Vincent A. H., Whyatt Y. L., Chilton N. F., Long J. R. (2023). J. Am. Chem. Soc..

[cit29] Lu D., Kwon H., Held L. I., McClain K. R., Ngo D. X., Ye M., Tsai H., Vincent A. H., Harris T. D., Long J. R. (2025). Inorg. Chem..

[cit30] Liu M., Chen Y.-C., Zheng J., Peng X.-H., Tong M.-L., Mansikkamäki A., Guo F.-S. (2025). Sci. China:Chem..

[cit31] Wang H., Zhang H.-L., Chen Y.-C., Shang T., Liu M., Peng X.-H., Liu Y., Tong M.-L., Guo F.-S. (2026). Inorg. Chem. Front..

[cit32] Braunschweig H., Chiu C.-W., Damme A., Engels B., Gamon D., Hörl C., Kupfer T., Krummenacher I., Radacki K., Walter C. (2012). Chem. – Eur. J..

[cit33] So C.-W., Watanabe D., Wakamiya A., Yamaguchi S. (2008). Organometallics.

[cit34] Braunschweig H., Breher F., Chiu C.-W., Gamon D., Nied D., Radacki K. (2010). Angew. Chem., Int. Ed..

[cit35] Kotyk C. M., Fieser M. E., Palumbo C. T., Ziller J. W., Darago L. E., Long J. R., Furche F., Evans W. J. (2015). Chem. Sci..

[cit36] Reinfandt N., Michenfelder N., Schoo C., Yadav R., Reichl S., Konchenko S. N., Unterreiner A. N., Scheer M., Roesky P. W. (2021). Chem. – Eur. J..

[cit37] Thakur S. K., Langer J., Harder S. (2025). Chem. – Eur. J..

[cit38] Schleyer P. v. R., Maerker C., Dransfeld A., Jiao H., van Eikema Hommes N. J. R. (1996). J. Am. Chem. Soc..

[cit39] Lu T., Chen F. (2012). J. Comput. Chem..

[cit40] Klod S., Kleinpeter E. (2001). J. Chem. Soc., Perkin Trans. 2.

[cit41] Liu Z., Lu T., Chen Q. (2020). Carbon.

[cit42] Guo F.-S., Day B. M., Chen Y.-C., Tong M.-L., Mansikkamäki A., Layfield R. A. (2018). Science.

[cit43] Rinehart J. D., Long J. R. (2011). Chem. Sci..

[cit44] Ungur L., Chibotaru L. F. (2016). Inorg. Chem..

[cit45] Day B. M., Guo F.-S., Layfield R. A. (2018). Acc. Chem. Res..

[cit46] Pointillart F., Cador O., Le Guennic B., Ouahab L. (2017). Coord. Chem. Rev..

[cit47] Liu J.-L., Chen Y.-C., Tong M.-L. (2018). Chem. Soc. Rev..

[cit48] Durrant J. P., Day B. M., Tang J., Mansikkamäki A., Layfield R. A. (2022). Angew. Chem., Int. Ed..

[cit49] Guo F.-S., Day B. M., Chen Y.-C., Tong M.-L., Mansikkamäki A., Layfield R. A. (2017). Angew. Chem., Int. Ed..

[cit50] Goodwin C. A. P., Ortu F., Reta D., Chilton N. F., Mills D. P. (2017). Nature.

[cit51] Randall McClain K., Gould C. A., Chakarawet K., Teat S. J., Groshens T. J., Long J. R., Harvey B. G. (2018). Chem. Sci..

[cit52] Evans P., Reta D., Whitehead G. F. S., Chilton N. F., Mills D. P. (2019). J. Am. Chem. Soc..

[cit53] Guo F.-S., He M., Huang G.-Z., Giblin S. R., Billington D., Heinemann F. W., Tong M.-L., Mansikkamäki A., Layfield R. A. (2022). Inorg. Chem..

[cit54] Guo Y.-N., Xu G.-F., Guo Y., Tang J. (2011). Dalton Trans..

[cit55] Gatteschi D. , Sessoli R. and Villain J., Molecular Nanomagnets, Oxford University Press, 2006

[cit56] Moreno-Pineda E., Taran G., Wernsdorfer W., Ruben M. (2019). Chem. Sci..

[cit57] Aquilante F., Autschbach J., Carlson R. K., Chibotaru L. F., Delcey M. G., De Vico L., Galván I. F., Ferré N., Frutos L. M., Gagliardi L., Garavelli M., Giussani A., Hoyer C. E., Li Manni G., Lischka H., Ma D., Malmqvist P. Å., Müller T., Nenov A., Olivucci M., Pedersen T. B., Peng D., Plasser F., Pritchard B., Reiher M., Rivalta I., Schapiro I., Segarra-Martí J., Stenrup M., Truhlar D. G., Ungur L., Valentini A., Vancoillie S., Veryazov V., Vysotskiy V. P., Weingart O., Zapata F., Lindh R. (2016). J. Comput. Chem..

[cit58] Chibotaru L. F., Ungur L., Soncini A. (2008). Angew. Chem., Int. Ed..

[cit59] Ungur L., Van den Heuvel W., Chibotaru L. F. (2009). New J. Chem..

[cit60] Chibotaru L. F., Ungur L., Aronica C., Elmoll H., Pilet G., Luneau D. (2008). J. Am. Chem. Soc..

[cit61] (a) CCDC 2523312: Experimental Crystal Structure Determination, 2026, 10.5517/ccdc.csd.cc2qpq5b

