# Supplementary material for: Synthesis of rare-earth borolide sandwich complexes by multi-electron borole reduction
Source: Chem Sci. 2026 Aug 27;17(41):19764–72. doi: 10.1039/d6sc05163d (PMC13539729; doi:10.1039/d6sc05163d)
Supplement: Supplementary file 1 [file SC-017-D6SC05163D-s001.pdf]

Supplementary Information (SI) for Chemical Science.
This journal is © The Royal Society of Chemistry 2026

Supplementary Information for:

Synthesis of Rare-Earth Borolide Sandwich Complexes by Multi-Electron Borole Reduction

Xiao-Han Peng[+],[a] Tao Shang[+],[a] Yan-Cong Chen,[b] Arpan Mondal,[c] Ming Liu,[a] Yu-Han Du,[a] Ying Liu,[a] Ming-Liang Tong,*[b] Richard A. Layfield,*[c] and Fu-Sheng Guo*[a]

a. Institute of Fundamental and Frontier Sciences, University of Electronic Science and Technology of China, Xiyuan Avenue 2006, Chengdu 611731, China.
E-mail: guofush@hotmail.com

b. Key Laboratory of Bioinorganic and Synthetic Chemistry of the Ministry of Education, School of Chemistry, IGCME, GBRCE for Functional Molecular Engineering, Sun Yat-Sen University, Guangzhou 510006, China.
E-mail: tongml@mail.sysu.edu.cn

c. Department of Chemistry, School of Life Sciences, University of Sussex, Brighton, BN1 9QR, U.K.
E-mail: r.layfield@sussex.ac.uk

[+] These authors contributed equally to this work.

Contents

1. General Considerations and Synthesis Procedures S2-4
2. FT-IR spectroscopy S5-7
3. NMR spectroscopy S8-20
4. Single-crystal X-ray crystallography S21-27
5. UV-vis-NIR spectroscopy S28-43
6. Magnetic property measurements S44-56
7. Computational details S57-75
8. References S76-77

General Considerations

All experiments were conducted under rigorous anhydrous, anaerobic conditions under an atmosphere of purified argon (99.9995%) using standard Schlenk line or glovebox techniques. Solvents were purified by a MIKROUNA solvent purification system and dried over activated 4 Å molecular sieves before use. C_6D_6 and THF-D_8 was refluxed over CaH_2 for at least one week and stored in an ampoule under argon. $(C_5{}^iPr_5)RE(BH_4)_2(THF)]$ (RE = La, Dy, Y),[1, 2] $[(C_5{}^iPr_5)RE(C_6H_6)M(C_5{}^iPr_5)]$ (RE = La, Y),[2] $[Fc(BBr_2)]$[3] and KC_8[4] were prepared according to literature procedures.

Elemental analyses were performed on a Vario Micro Cube automatic element analyzer. FT-IR spectra were recorded on a Bruker Alpha II FT-IR spectrometer equipped with a Platinum ATR module. UV/vis/NIR absorption spectra were collected from 200-1000 nm on a Shimadzu UV-3600 Plus spectrophotometer at room temperature. NMR spectra were recorded on a Bruker Avance III HD 600 MHz spectrometer at room temperature. NMR spectra were referenced to the residual proton signals. Assignment of resonances in the ^1H and $^{13}C\{^1H\}$ NMR spectra were made based on two-dimensional ^1H-^{13}C HSQC NMR spectra.

Synthesis of $FcBC_2(SiMe_3)_2C_2Me_2$ (1)

An ampoule was charged with [Fc(BBr_2)] (580 mg, 1.62 mmol), *n*-hexane (25 mL) and a glass-coated stirrer bar, then cooled to 0 °C. To the above solution was added the hexane solution of 1,4-dilithio-1,4-bis(trimethylsilyl)-2,3-dimethyl-1,3-butadien (390 mg, 1.62 mmol) dropwise. A white precipitate of LiBr was produced immediately and the mixture was stirred for further 24 hours at ambient temperature. The solvent was removed under vacuum, and the dark red residue was extracted into *n*-hexane (2 × 30 mL) and filtered. The combined extracts were concentrated to ca. 10 mL and filtered. Dark red block X-ray quality crystals (320 mg, 47%) were grown at −35°C overnight.

Elemental analysis [%] calculated for [$C_{22}H_{33}BFeSi_2$] (420.33 g/mol): C 62.87, H 7.91; found: C 62.50, H 7.86.

FT-IR (cm^{-1}, Figure S1): 2941m, 2890m, 1554w, 1507m, 1435s, 1241s, 1197m, 1165m, 1107w, 1081m, 1030m, 998w, 929w, 834vs, 819vs, 752s, 677s, 619s, 539w, 492m, 474s, 437w.

^1H NMR (600.13 MHz, 298 K, C_6D_6, Figure S6): δ 4.64 (t, *J* = 1.9 Hz 2H, C_5H_4), 4.52 (m, 2H, C_5H_4), 3.96 (s, 5H, C_5H_5), 1.97 (s, 6H, C_4B-CH_3), 0.39 (s, 18H, Si-CH_3).

$^{13}C\{^1H\}$ NMR (150.92 MHz, 298 K, C_6D_6, Figure S7): δ 170.16 (B–C=C), 144.09 (B–C=C), 128.53 (C_4B-C_5H_4), 80.25 (C_5H_4), 76.45 (C_5H_4), 71.63 (C_5H_5), 19.38 (C_4B-CH_3), 2.44(Si-CH_3).

$^{11}B\{^1H\}$ NMR (192.55 MHz, 298 K, C_6D_6, Figure S8): δ 61.54.

$^{29}Si\{^1H\}$ NMR (119 MHz, 298 K, C_6D_6, Figure S9): δ -12.23.

Synthesis of [Mg($FcBC_2(SiMe_3)_2C_2Me_2$)$(THF)_3$]·THF (2)

THF (10 mL) was added to an ampoule containing $FcBC_2(SiMe_3)_2C_2Me_2$ (**1**) (221 mg 0.5 mmol), Mg chip (48 mg 2 mmol 4 eq) and a glass coated stirrer bar, and the resulting red mixture was stirred at ambient temperature for 2 days. The solvent was removed under vacuum, and the residue was washed with hexane to remove unreacted starting materials. The resulting solid was then dissolved in THF (3 mL) and filtered. The red crystals were grown at −35°C overnight. Yield: 205 mg, 28.0%.

Elemental analysis [%] calculated for [$C_{34}H_{57}BFeMgO_3Si_2$]·(C_4H_8O) (1466 g/mol): C 62.26, H 8.94; found: C 61.93, H 9.05.

FT-IR (cm^{-1}, Figure S2): 3087w, 2939m, 2886m, 1451w, 1301w, 1283w, 1231s, 1153m, 1102w, 1061w, 1020s, 866s, 839vs, 810vs, 749s, 654m, 603m, 490s, 439m.

^1H NMR (600.13 MHz, 298 K, THF-D_8, Figure S10): δ 4.44 (s, 2H, C_5H_4), 4.01 (s, 2H, C_5H_4), 3.88 (s, 5H, C_5H_5), 3.64-3.60 (m,12H, 3 C_4H_8O), 2.21 (s, 6H, C_4B-CH_3), 1.79–1.76 (m, 12H, 3 C_4H_8O), 0.34 (s, 18H, Si-CH_3).

$^{13}C\{^1H\}$ NMR (150.92 MHz, 298 K, THF-D_8, Figure S11): δ 123.08 (C_4B–C), 75.42 (C_5H_4), 68.43 (C_5H_5), 67.05 (C_5H_4), 68.43-66.46(C_4H_8O), 25.72-24.39 (C_4H_8O), 17.83 (C_4B-CH_3), 6.89 (Si-CH_3).

$^{11}B\{^1H\}$ NMR (192.55 MHz, 298 K, THF-D_8, Figure S12): δ 33.76.

$^{29}Si\{^1H\}$ NMR (119 MHz, 298 K, THF-D_8, Figure S13): δ -14.36.

Synthesis of [($C_5{}^iPr_5$)La($FcBC_2(SiMe_3)_2C_2Me_2$)]·2toluene (3-La)

Method A: Benzene (10 mL) was added to an ampoule containing [($C_5{}^iPr_5$)La(C_6H_6)La($C_5{}^iPr_5$)] (35 mg, 0.039 mmol), $FcBC_2(SiMe_3)_2C_2Me_2$ (34 mg, 0.077 mmol), and a glass-coated stirrer bar. The resulting suspension was heated at 60 °C for 24 hours, giving a dark purple solution. The solvent was removed under vacuum and then extracted into toluene. The combined toluene extracts were concentrated and filtered. X-ray quality crystals of [($C_5{}^iPr_5$)La($FcBC_2(SiMe_3)_2C_2Me_2$)]·2toluene (21 mg, 26.7%) were isolated by storing the solution at –35 °C for 5 days.

Method B: Benzene (20 mL) was added to an ampoule containing $FcBC_2(SiMe_3)_2C_2Me_2$ (70.9 mg, 0.16 mmol), [($C_5{}^iPr_5$)La$(BH_4)_2$(THF)] (81.3 mg, 0.16 mmol), KC_8 (48 mg, 0.35 mmol) and a glass-coated stirrer bar. The reaction mixture was stirred at ambient temperature for 2 days, resulting in a dark purple solution and a black precipitate. The solvent was removed under vacuum, and the residue was extracted into *n*-hexane (3 × 15 mL). The combined hexane extracts were evaporated to dryness *in vacuo*, giving a dark purple powder. Crystals of

$[(C_5{}^iPr_5)La(FcBC_2(SiMe_3)_2C_2Me_2)]\cdot 2$toluene (50 mg, 31.74%) suitable for X-ray crystallography were grown from a toluene solution at –35 °C.

Elemental analysis [%] calculated for $[C_{42}H_{68}BFeLaSi_2]\cdot 2(C_7H_8)]$ (1018.98 g/mol): C 66.01, H 8.31; found: 65.64, H 8.31.

FT-IR (cm^{-1}, Figure S3): 2970m, 2933m, 2867m, 1601w, 1494m, 1459m, 1365m, 1319m, 1239s, 1194m, 1155m, 1106w, 1080s, 1026m, 999m, 872m, 840vs, 825vs, 812vs, 753m, 728vs, 693s, 671s, 597m, 492vs, 463s.

^{1}H NMR (600 MHz, 298 K, C_6D_6, Figure S15): δ 7.13 (t, *J* = 7.6 Hz, 2H, C_6H_5-CH_3), 7.07 – 6.99 (m, 3H, C_6H_5-CH_3), 4.62 (s, 1H, C_5H_4), 4.51 (s, 1H, C_5H_4), 4.45 (s, 5H, C_5H_5), 4.41 (s, 2H, C_5H_4), 2.94 (hept, *J* = 7.3 Hz, 5H, *i*Pr-CH), 2.54 (d, *J* = 11.4 Hz, 6H, C_4B-CH_3), 2.11 (s, 6H, 2 C_6H_5-CH_3), 1.17 (d, *J* = 7.4 Hz, 15H, *i*Pr-CH_3), 1.05 (d, *J* = 7.2 Hz, 15H, *i*Pr-CH_3), 0.56 (d, *J* = 9.0 Hz, 18H, Si-CH_3).

^{13}C{^{1}H} NMR (151 MHz, 298 K, C_6D_6, Figure S16): δ 137.60 - 125.41 (C_6H5-CH_3), 77.87 (C_5H_4), 77.27 (C_5H_4), 71.68 (C_5H_4), 70.14 (C_5H_5), 26.25 ($(CH_3)_2CH$-C_5), 23.05 - 22.97 ($(CH_3)_2CH$-C_5), 21.08 (C_6H_5-CH_3), 19.66 - 19.56 (C_4B-CH_3), 5.68 - 5.60 (Si-CH_3).

^{11}B{^{1}H} NMR (192.55 MHz, 298 K, C_6D_6, Figure S17): δ 49.33.

^{29}Si{^{1}H} NMR (119 MHz, 298 K, C_6D_6, Figure S18): δ -15.69.

Synthesis of $[(C_5{}^iPr_5)Dy(FcBC_2(SiMe_3)_2C_2Me_2)]$ (3-Dy)

Following the general procedure used for **3-La** (Method B). $FcBC_2(SiMe_3)_2C_2Me_2$ (101.9 mg, 0.23 mmol), $[(C_5{}^iPr_5)Dy(BH_4)_2(THF)]$ (124.3 mg, 0.23 mmol) and KC_8 (68.8 g, 0.51 mmol) were combined to give **3-Dy** as dark red crystals. Yield: 52 mg, 22.0%.

Elemental analysis [%] calculated for $[C_{42}H_{68}BFeDySi_2]\cdot 2(C_7H_8)]$ (1042.57 g/mol): C 64.34, H 8.21; found: C 64.51, H 8.12.

FT-IR (cm^{-1}, Figure S4): 2931m, 2892m, 1601w, 1458m, 1367m, 1309m, 1242vs, 1214m, 1154s, 1106m, 1080s, 1206m, 998m, 873m, 840vs, 825vs, 821vs, 755s, 739vs, 693s, 669m, 618m, 599m, 490vs, 414w.

Synthesis of $[(C_5{}^iPr_5)Y(C_4B)^{FcTMS2Me2}]\cdot 2$toluene (3-Y)

Following the general procedure used for **3-La** (Method B). $FcBC_2(SiMe_3)_2C_2Me_2$ (99.5, 0.22 mmol), $[(C_5{}^iPr_5)Y(BH_4)_2(THF)]$ (102.9 mg, 0.22 mmol) and KC_8 (67 g, 0.49 mmol) were combined to give **3-Y** as dark red crystals. Yield: 55 mg, 25.8%.

Elemental analysis [%] calculated for $[C_{42}H_{68}BFeYSi_2]\cdot(C_7H_8)]$ (876.88 g/mol): C 69.41, H 8.74; found: 67.09, H 8.81.

FT-IR (cm^{-1}, Figure S5): 2970m, 2933m, 2894m, 1601w, 1494m, 1455m, 1367m, 1307m, 1242vs, 1211m,1194m, 1155s,1106m, 1081s, 1026m, 999m, 872m, 841vs, 827vs, 810vs, 757s, 728vs, 693s,669m, 618m, 597m, 517m, 490vs, 414w.

^{1}H NMR (600.13 MHz, 298 K, C_6D_6, Figure S22): δ 7.13 (t, *J* = 7.4 Hz, 2H, C_6H_5), 7.04 (dd, *J* = 24.4, 7.1 Hz, 3H, C_6H_5), 4.91 (s, 1H, C_5H_4), 4.84 (s, 1H, C_5H_4), 4.44 (t, *J* = 1.8 Hz, 2H, C_5H_4), 4.37 (s, 5H, C_5H_5), 2.91 (hept, *J* = 7.3 Hz, 6H, *i*Pr-CH), 2.53 (d, *J* = 8.3 Hz, 6H, C_4B-CH_3), 2.11 (s, 5H,C_6H_5-CH_3), 1.30 (d, *J* = 7.4 Hz, 15H, *i*Pr-CH_3), 0.94 (d, *J* = 7.3 Hz, 15H, *i*Pr-CH_3), 0.67 (s, 18H, Si-CH_3).

^{13}C {^{1}H} NMR (151 MHz, 298 K, C_6D_6, Figure S23): δ 137.60 - 125.41 (C_6H_5-CH_3), 78.27 (C_5H_4), 77.43 (C_5H_4), 69.50 (C_5H_5), 26.37 ($(CH_3)_2CH$-C_5), 23.59 - 23.44 ($(CH_3)_2CH$-C_5), 21.15-20.04 (C_4B-CH_3), 6.67 - 6.55 (Si-CH_3).

^{11}B{^{1}H} NMR(193 MHz, 298 K, C_6D_6, Figure S24): δ 52.59 ppm.

^{29}Si{^{1}H} NMR (119 MHz, 298 K, C_6D_6, Figure S25): δ -12.96 ppm.

FT-IR spectroscopy

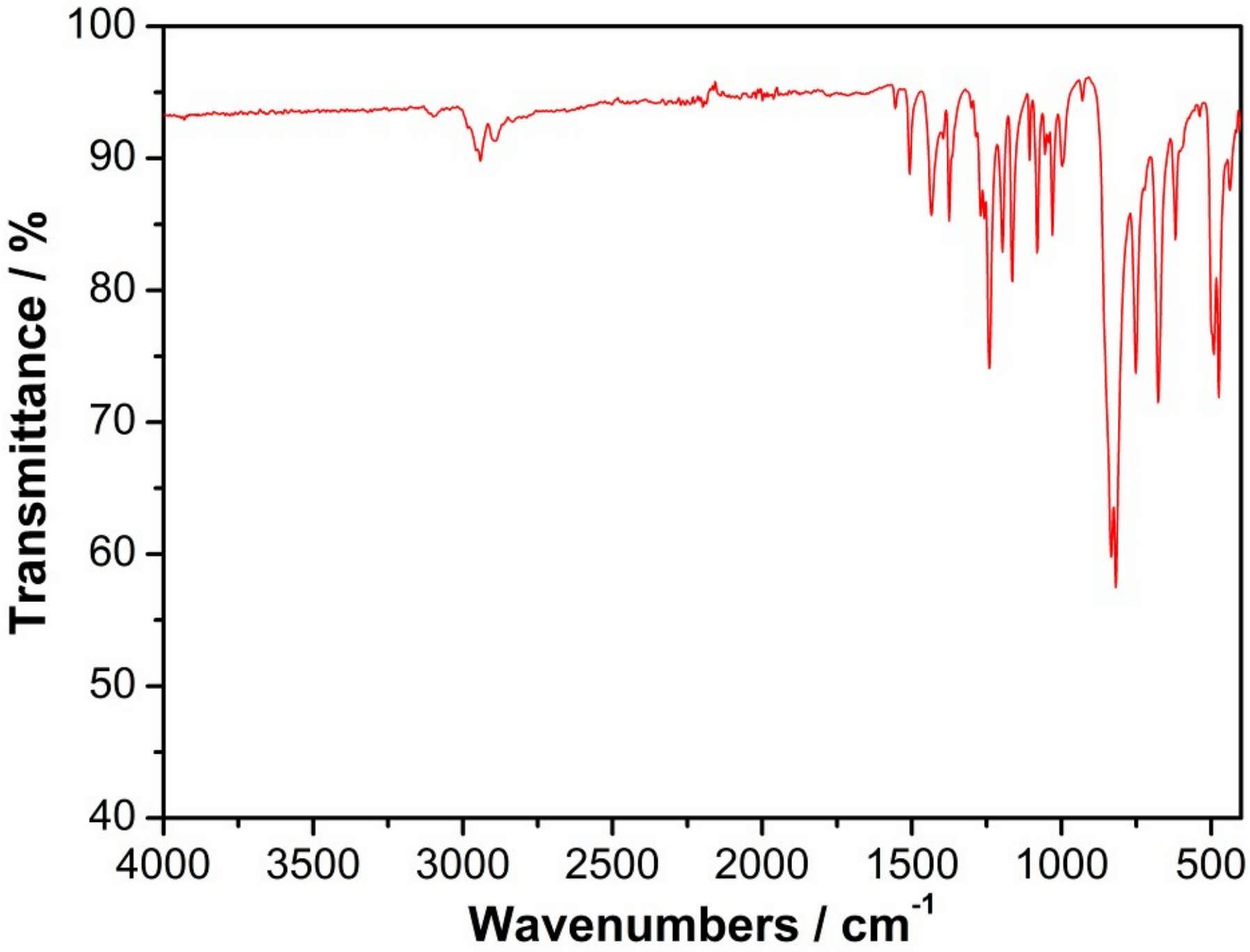

Figure S1. FT-IR spectrum of $FcBC_2(SiMe_3)_2C_2Me_2$ (**1**).

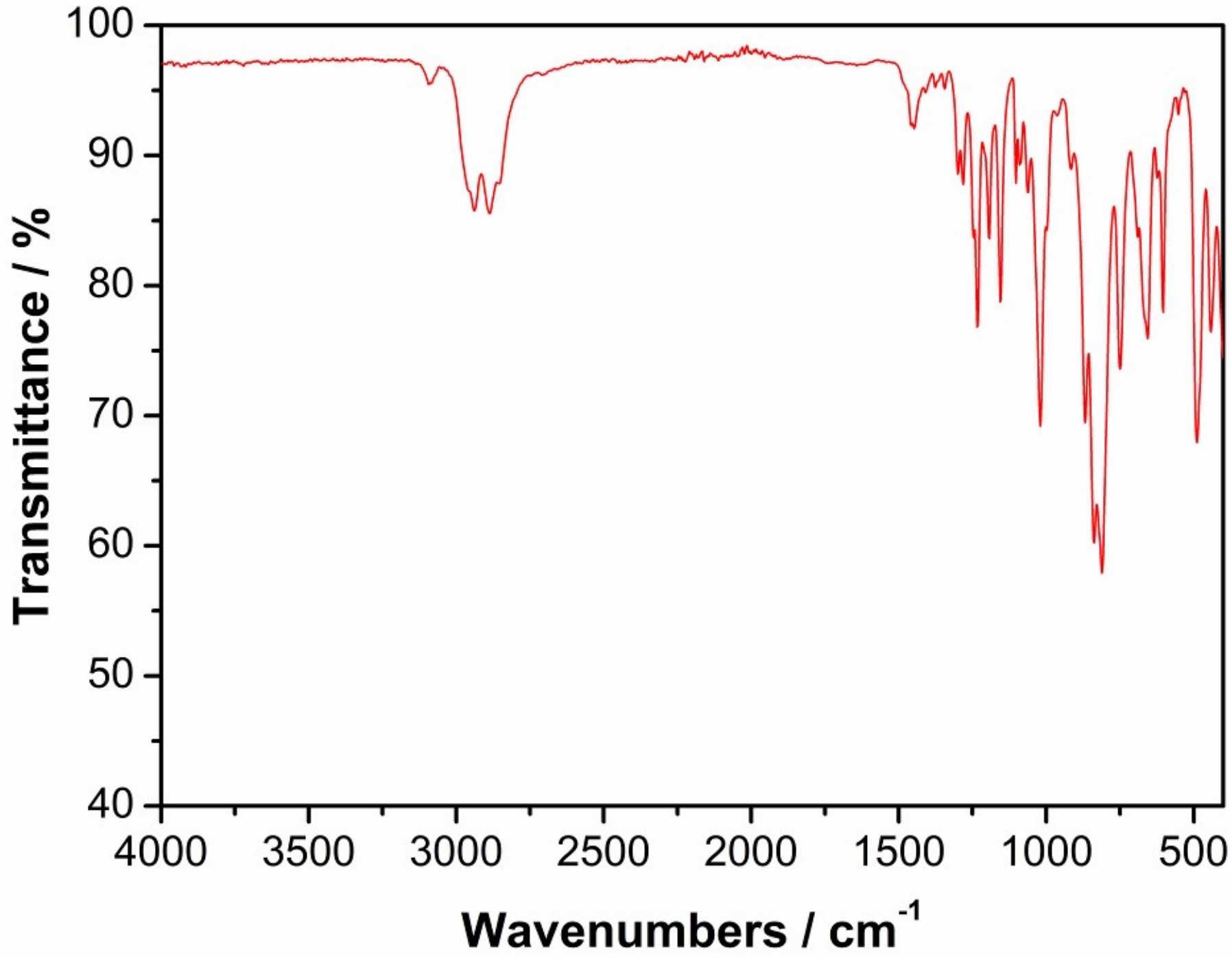

Figure S2. FT-IR spectrum of $[Mg(FcBC_2(SiMe_3)_2C_2Me_2)(THF)_3]\cdot THF$ (**2**).

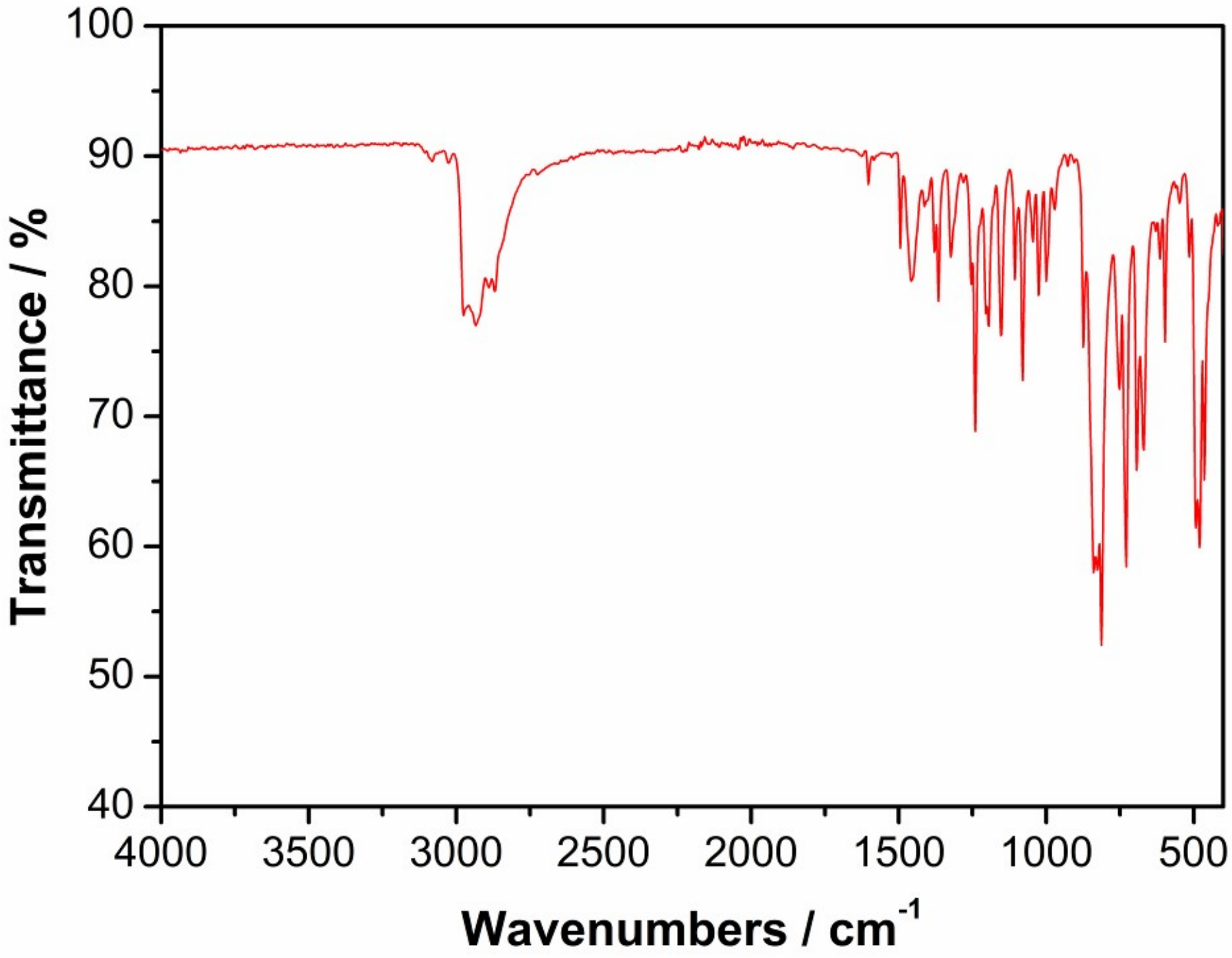

Figure S3. FT-IR spectrum of $[(C_5{}^iPr_5)La(FcBC_2(SiMe_3)_2C_2Me_2)]\cdot$2toluene (**3-La**).

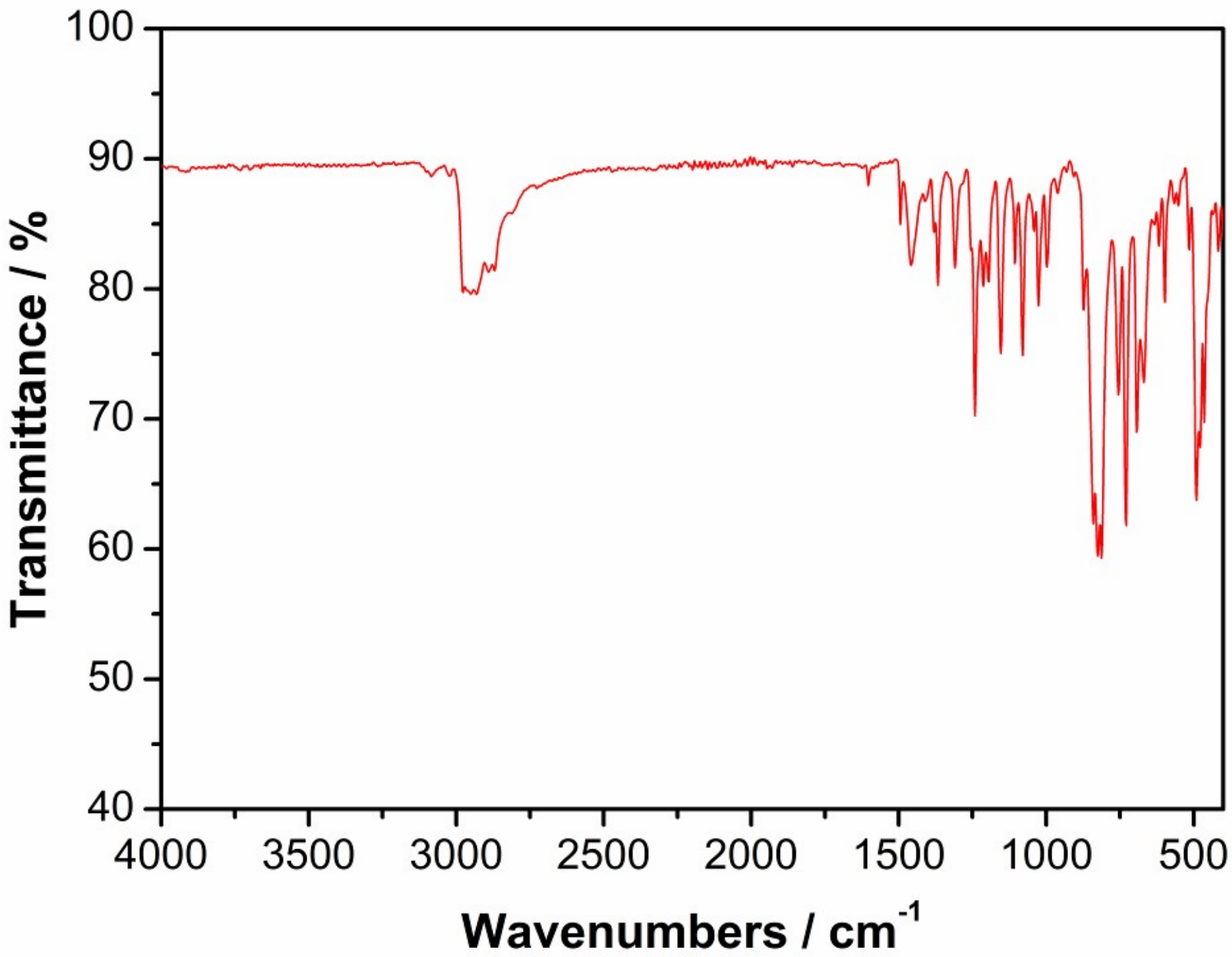

Figure S4. FT-IR spectrum of $[(C_5{}^iPr_5)Dy(FcBC_2(SiMe_3)_2C_2Me_2)]\cdot$2toluene (**3-Dy**).

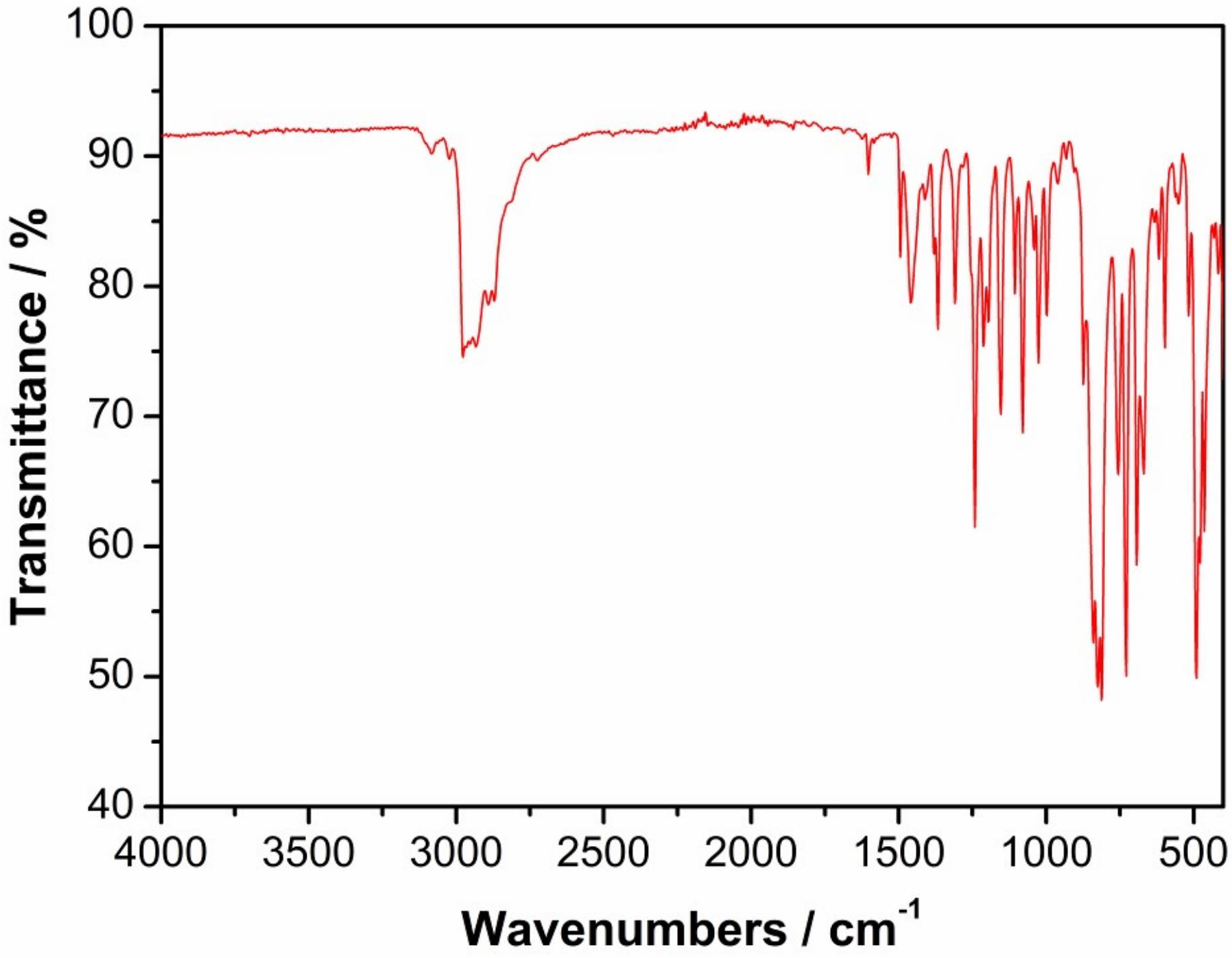

Figure S5. FT-IR spectrum of $[(C_5{}^iPr_5)Y(FcBC_2(SiMe_3)_2C_2Me_2)]\cdot 2toluene$ (**3-Y**).

NMR Spectroscopy

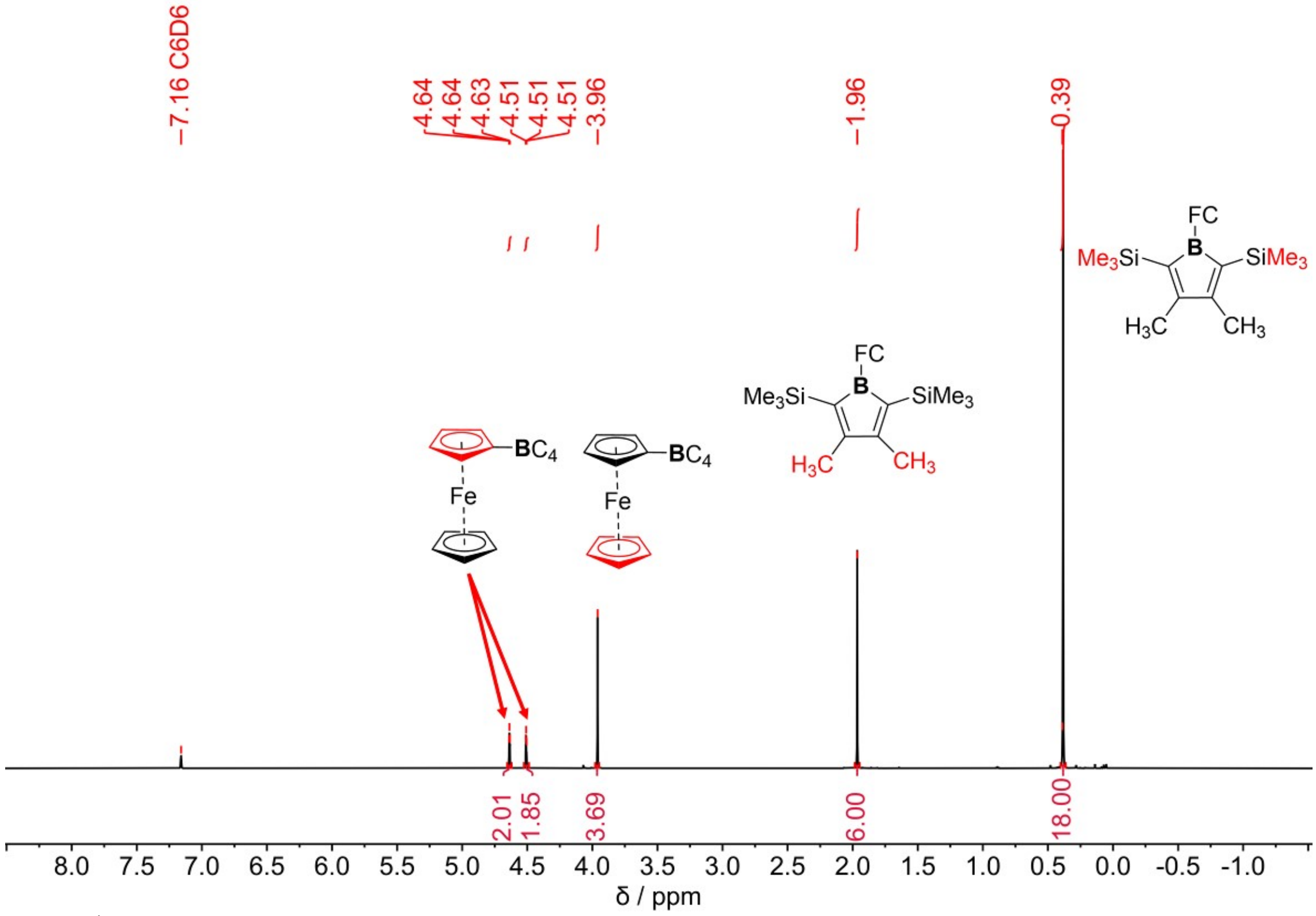

Figure S6. ^{1}H NMR spectrum of $FcBC_2(SiMe_3)_2C_2Me_2$ (**1**) in C_6D_6.

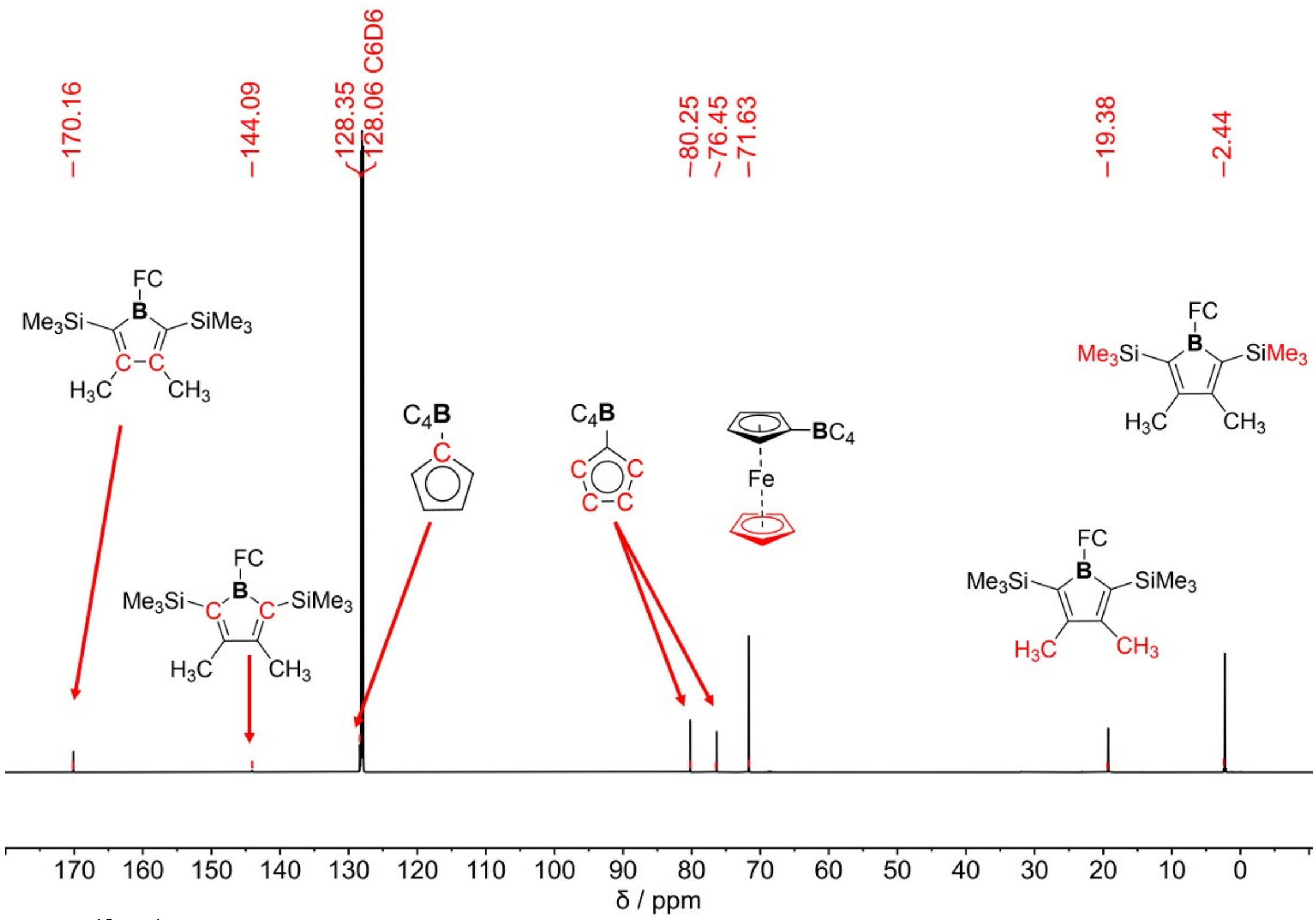

Figure S7. $^{13}C\{^{1}H\}$ NMR spectrum of $FcBC_2(SiMe_3)_2C_2Me_2$ (**1**) in C_6D_6.

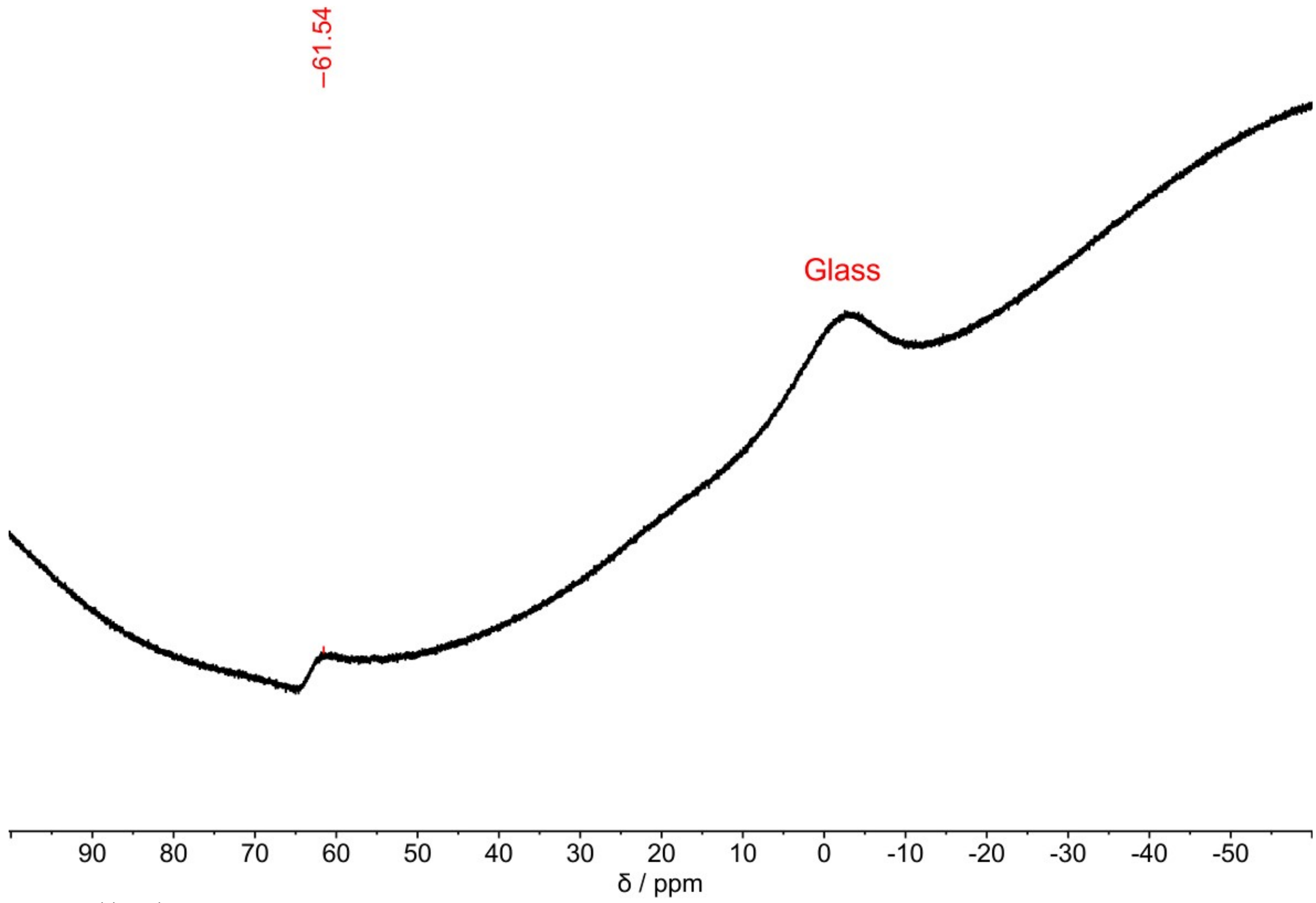

Figure S8. $^{11}B\{^1H\}$ NMR spectrum of $FcBC_2(SiMe_3)_2C_2Me_2$ (**1**) in C_6D_6.

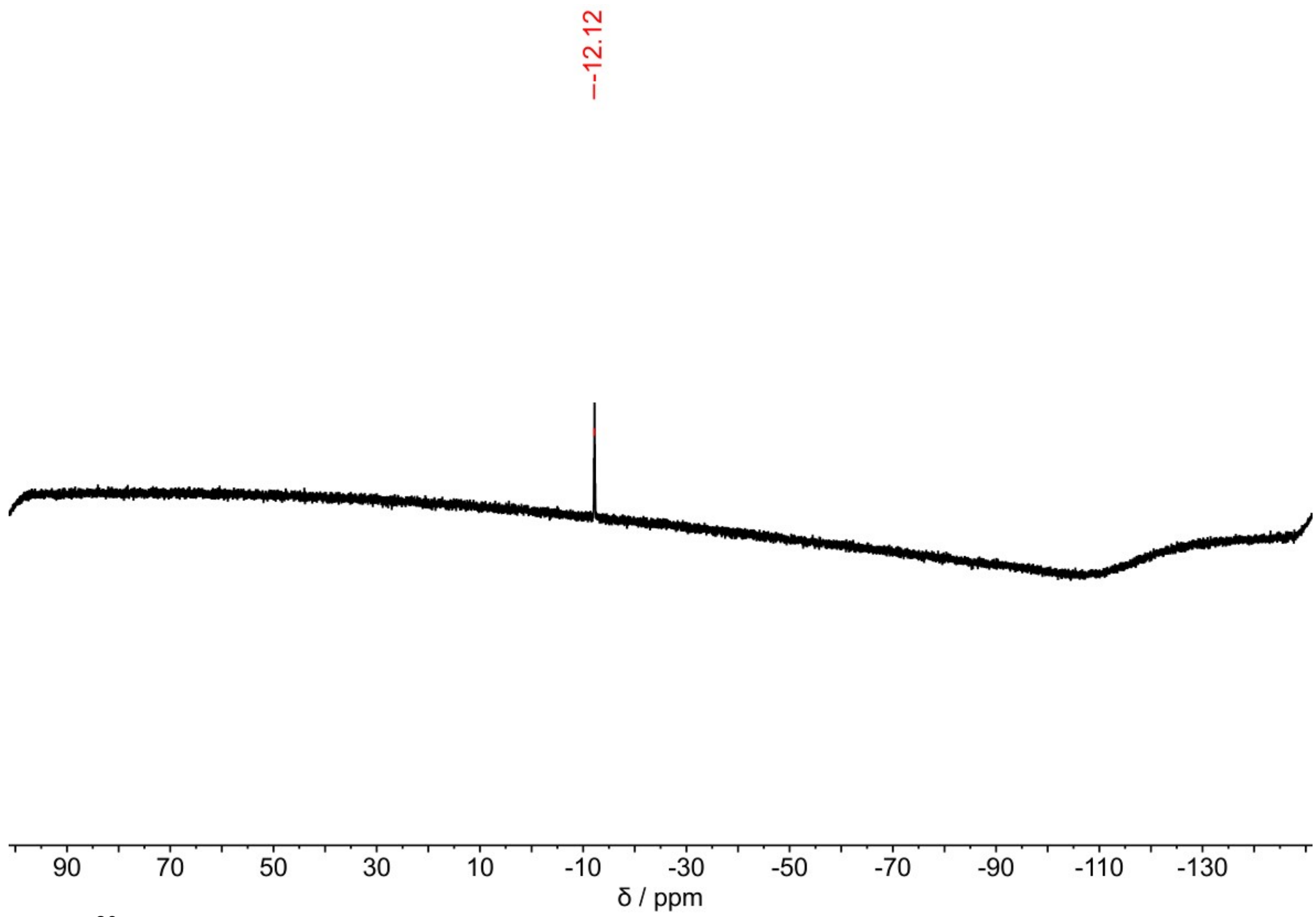

Figure S9. ^{29}Si NMR spectrum of $FcBC_2(SiMe_3)_2C_2Me_2$ (**1**) in C_6D_6.

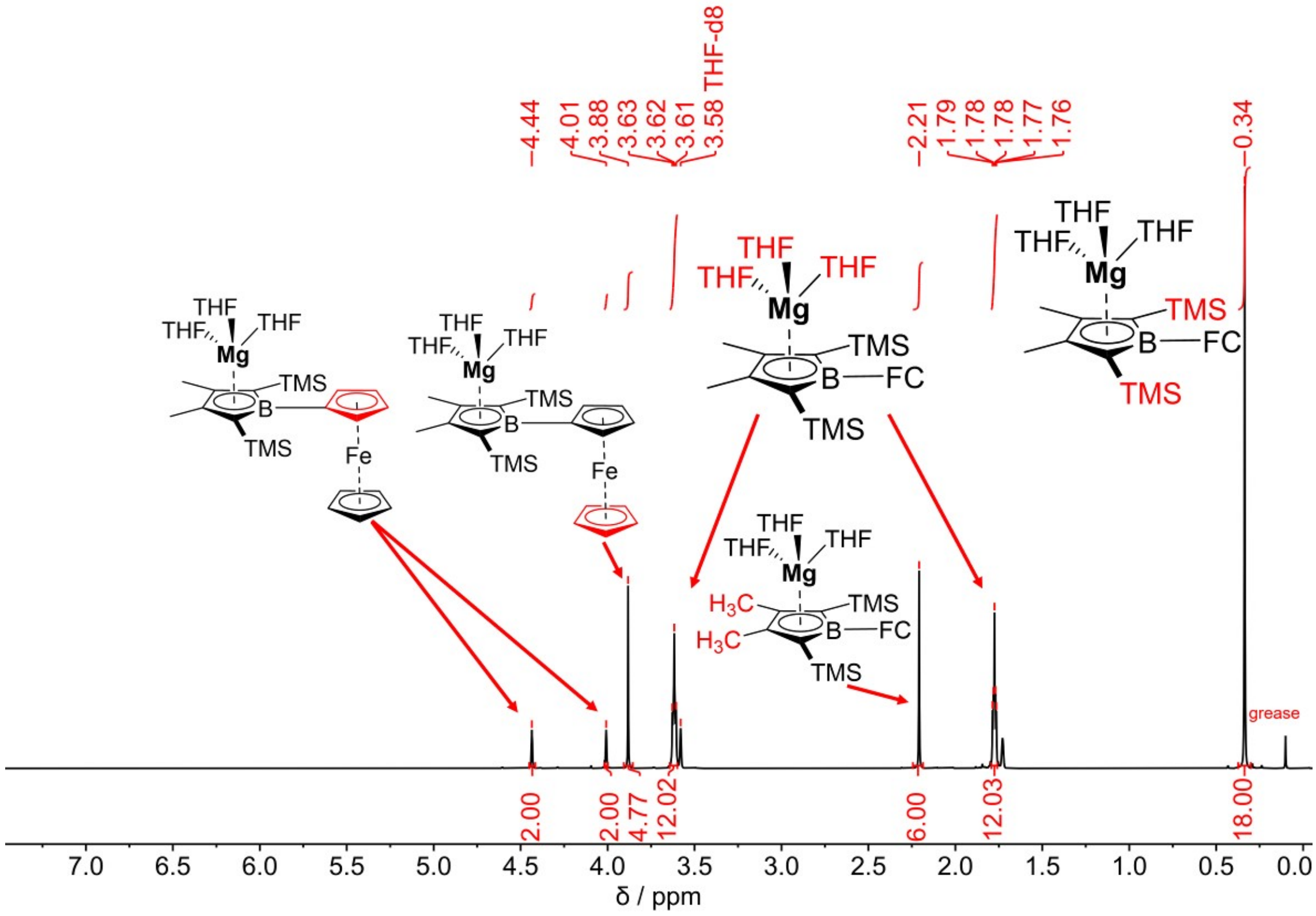

Figure S10. ^{1}H NMR spectrum of $[Mg(FcBC_2(SiMe_3)_2C_2Me_2)(THF)_3]\cdot THF$ (**2**) in THF-d_8.

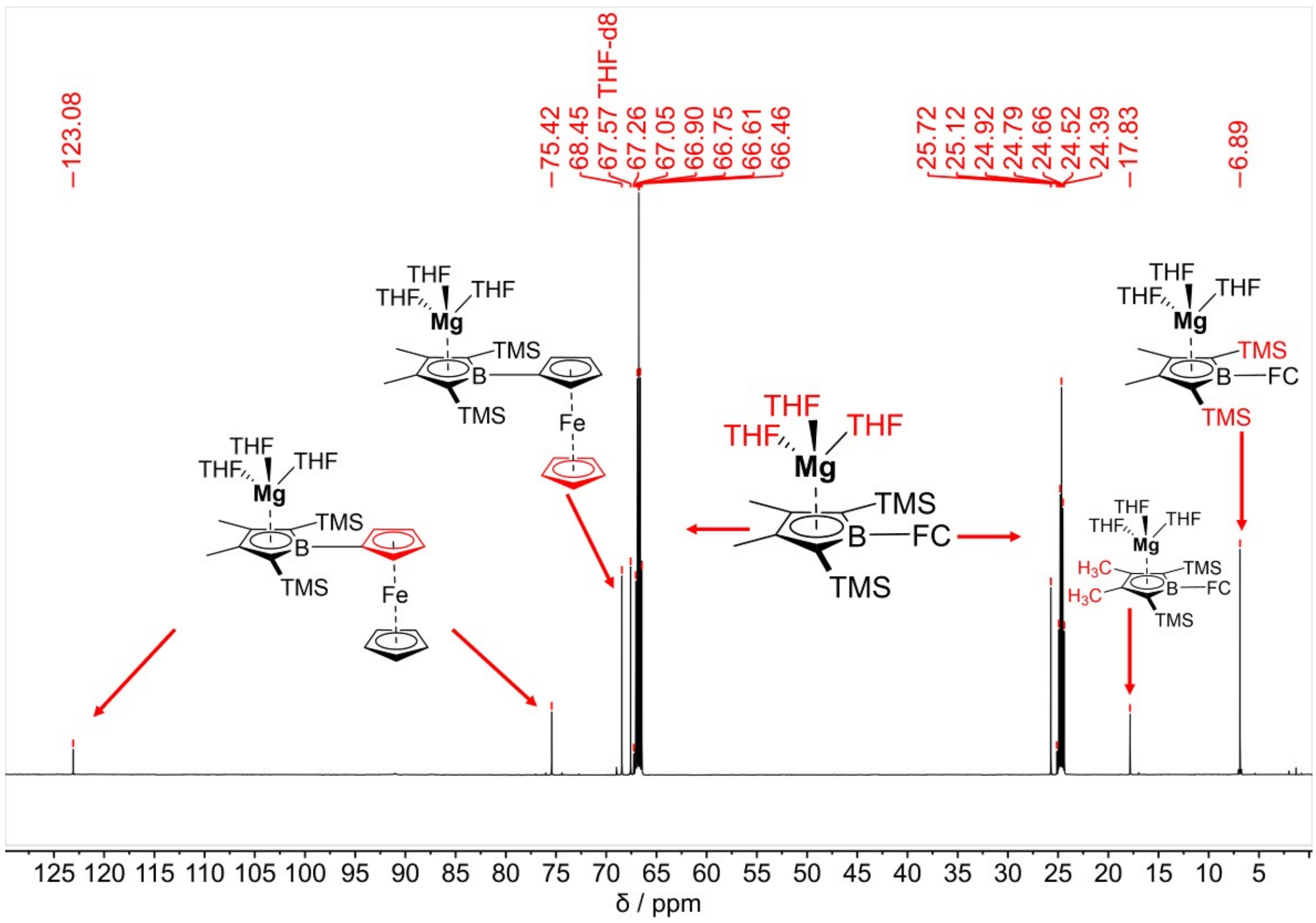

Figure S11. $^{13}C\{^{1}H\}$ NMR spectrum of $[Mg(FcBC_2(SiMe_3)_2C_2Me_2)(THF)_3]\cdot THF$ (**2**) in THF-d_8.

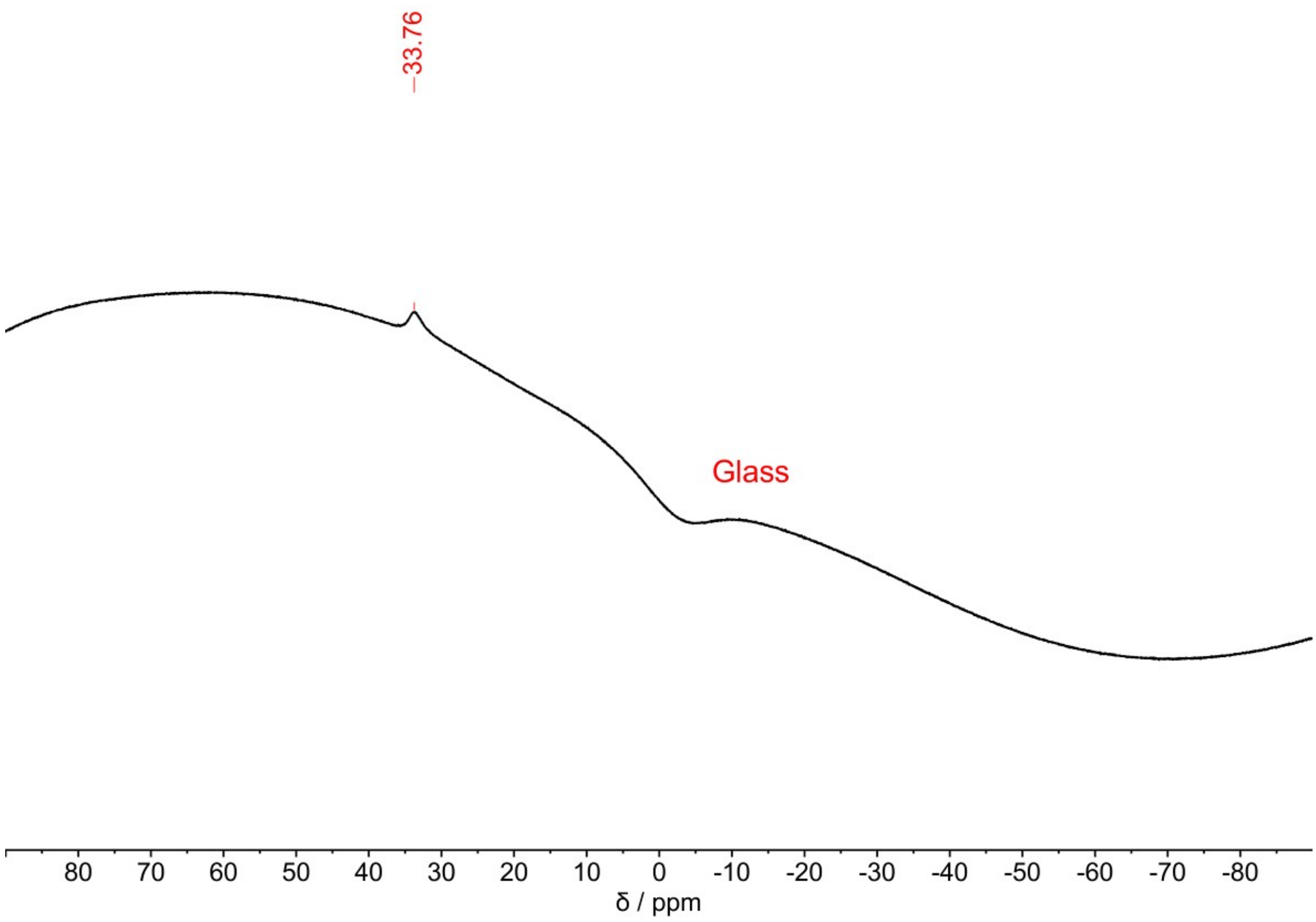

Figure S12. $^{11}B\{^{1}H\}$ NMR spectrum of $[Mg(FcBC_2(SiMe_3)_2C_2Me_2)(THF)_3]\cdot THF$ (**2**) in THF-d_8.

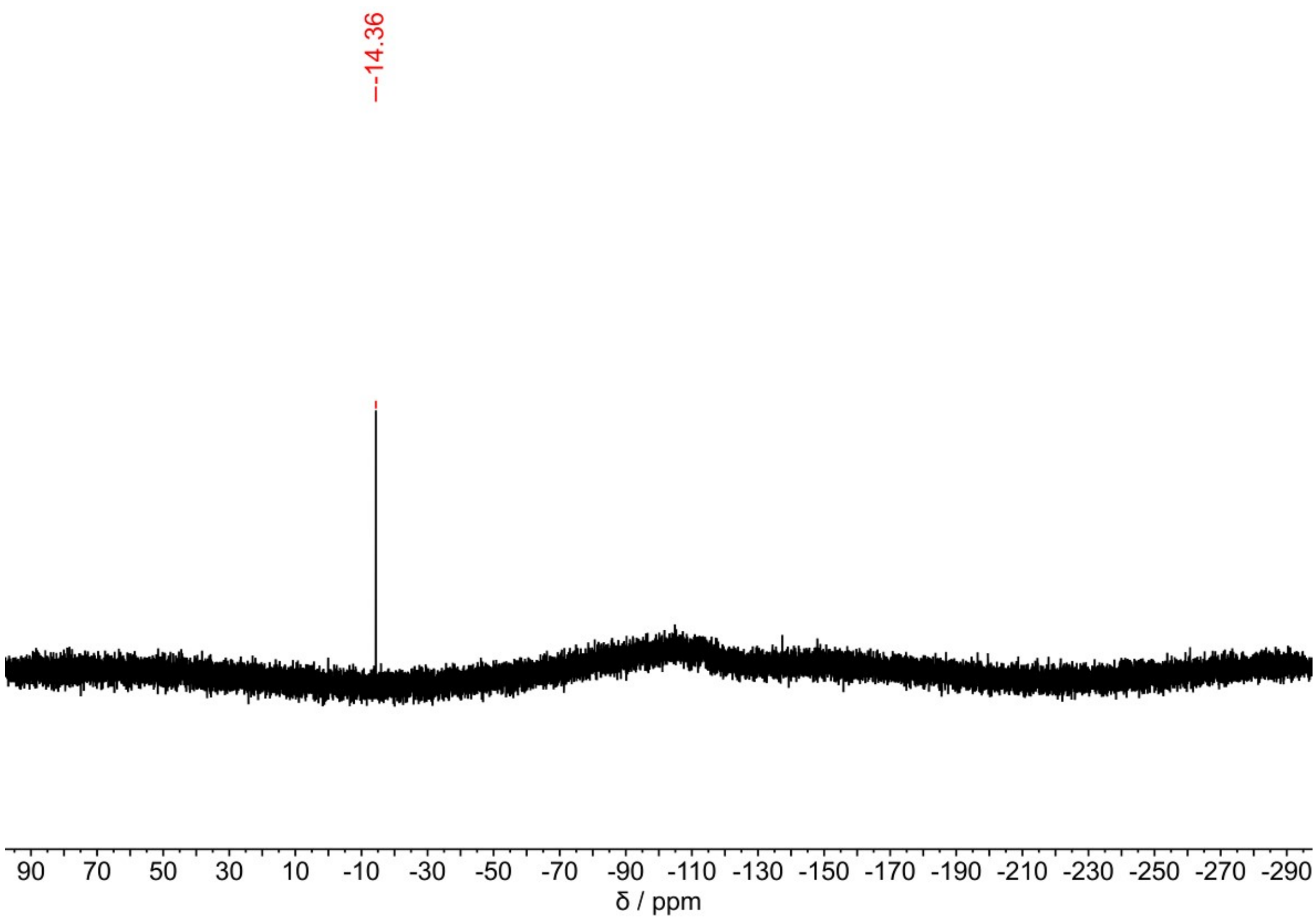

Figure S13. $^{29}Si\{^{1}H\}$ NMR spectrum of $[Mg(FcBC_2(SiMe_3)_2C_2Me_2)(THF)_3]\cdot THF$ (**2**) in THF-d_8.

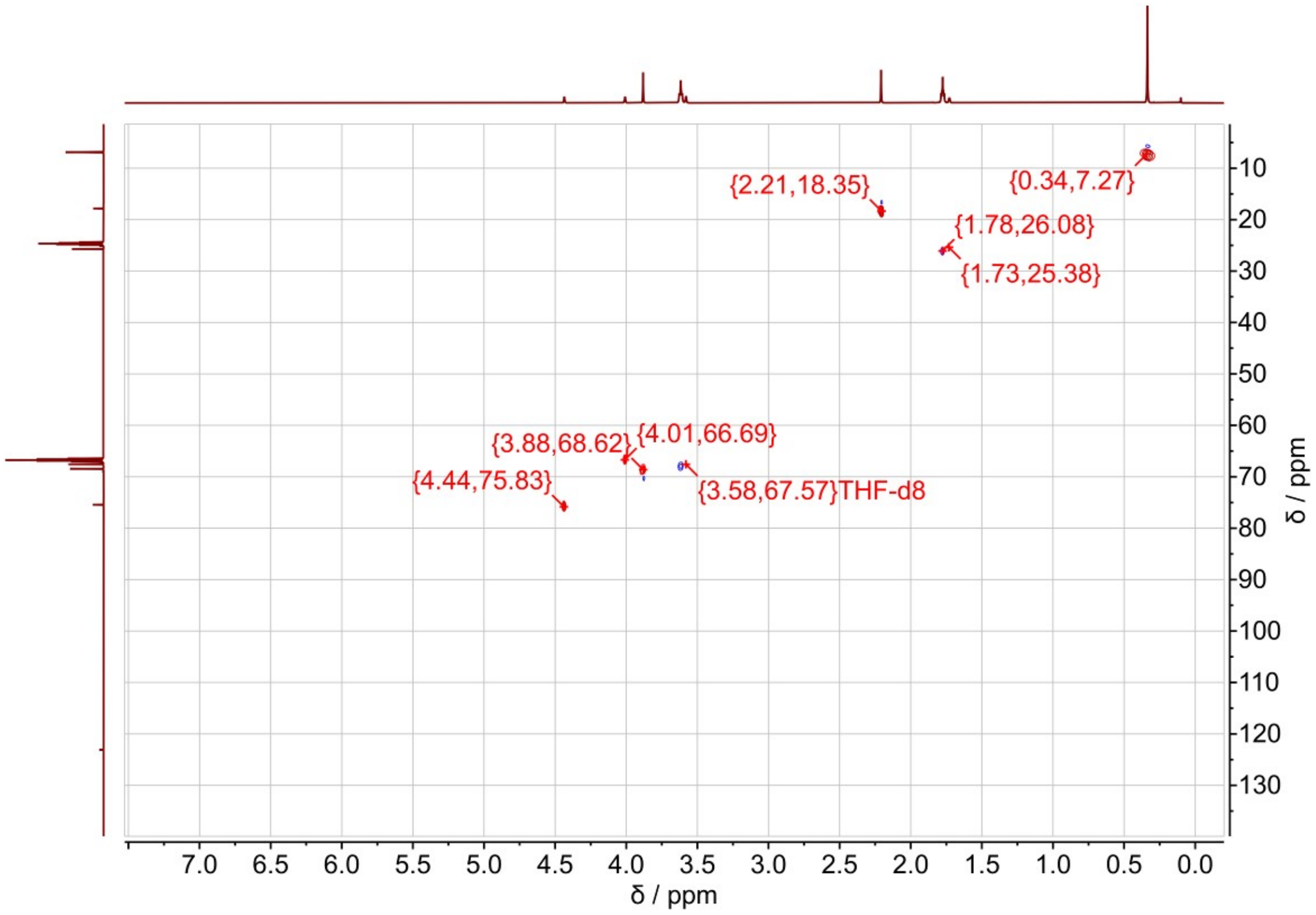

Figure S14. ^{1}H-^{13}C HSQC NMR spectrum of $[Mg(FcBC_2(SiMe_3)_2C_2Me_2)(THF)_3]\cdot THF$ (**2**) in THF-d_8.

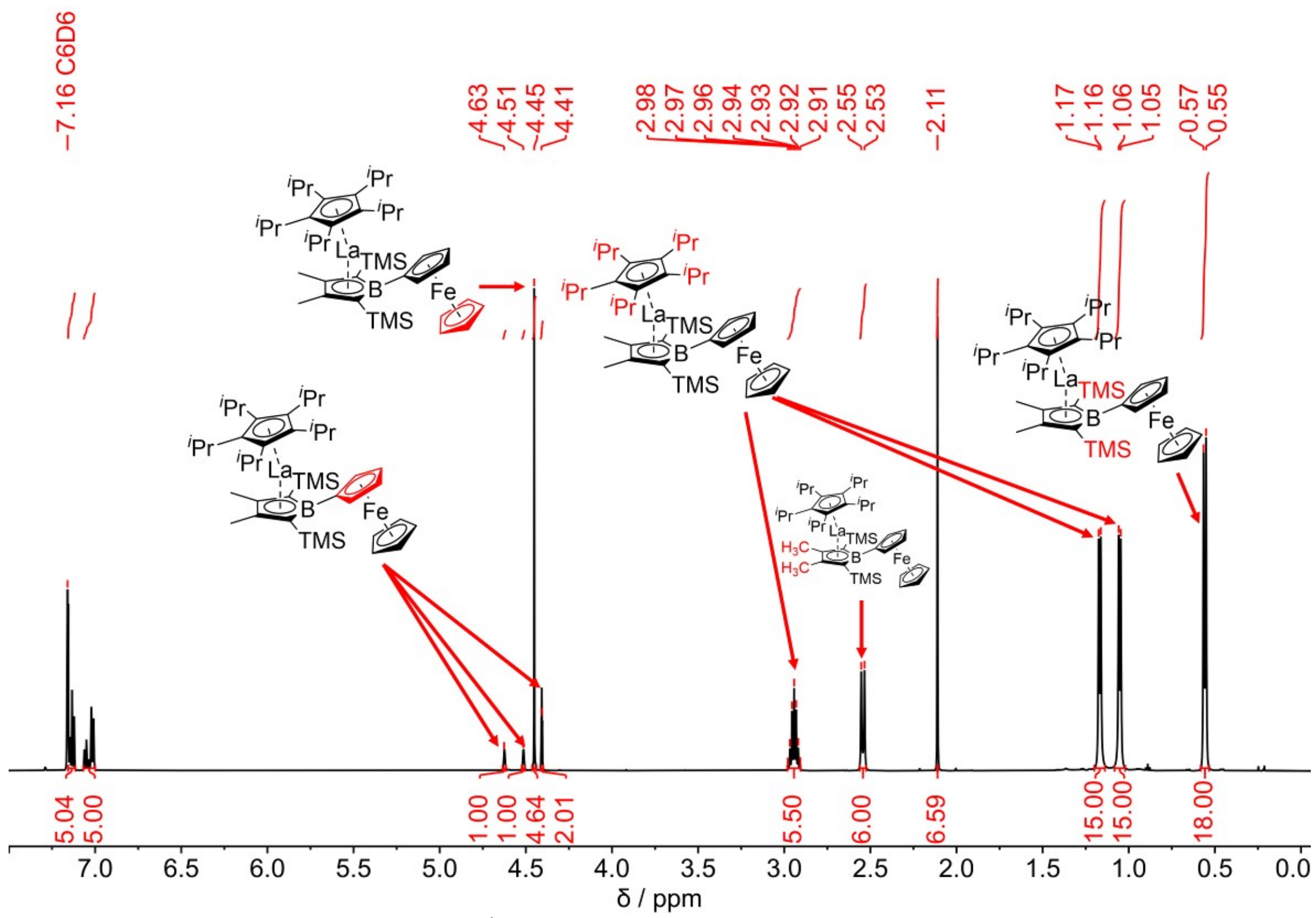

Figure S15. ^{1}H NMR spectrum of [($C_5{}^iPr_5$)La($FcBC_2(SiMe_3)_2C_2Me_2$)]·2toluene (**3-La**) in C_6D_6.

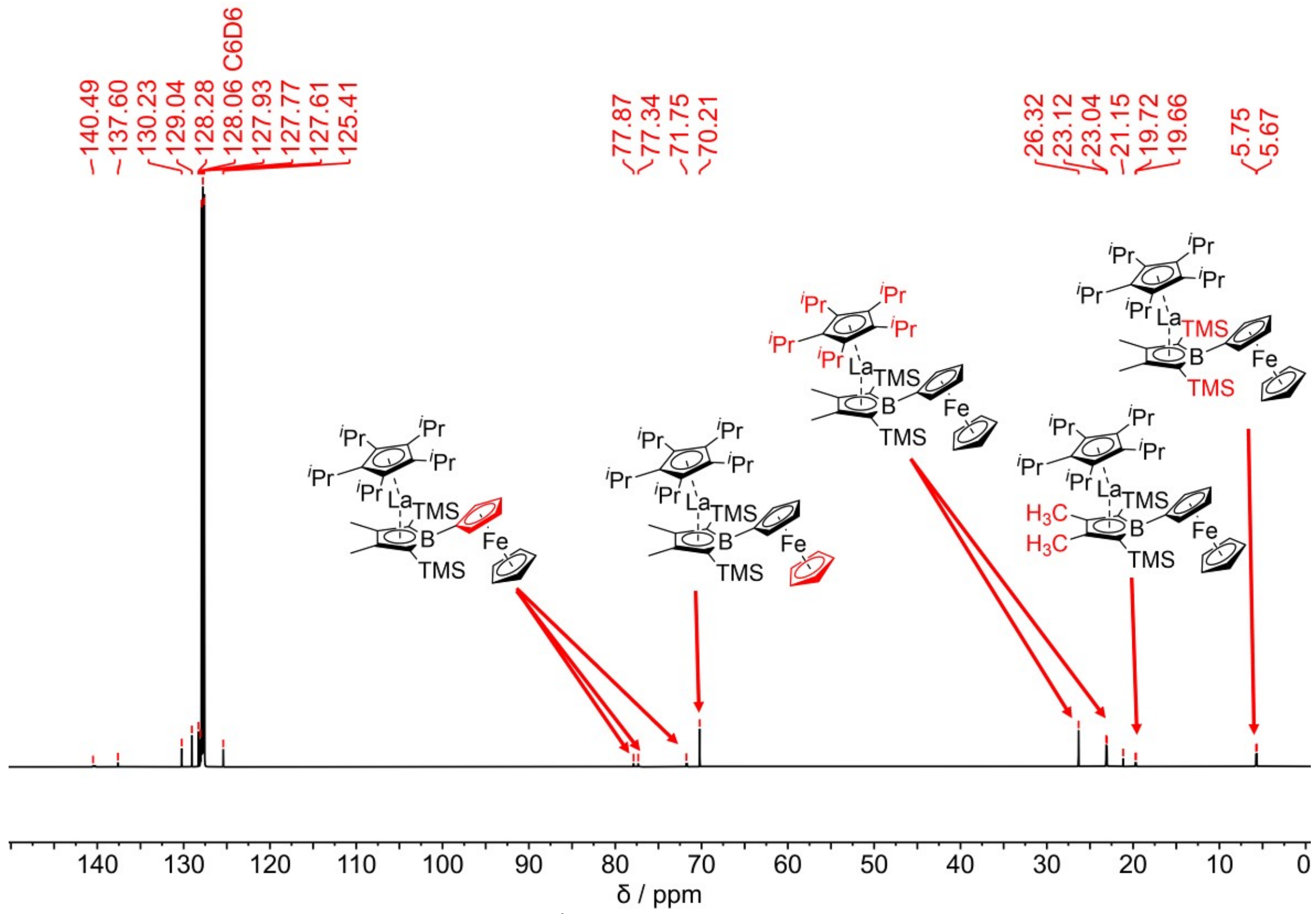

Figure S16. $^{13}C\{^{1}H\}$ NMR spectrum of [($C_5{}^iPr_5$)La($FcBC_2(SiMe_3)_2C_2Me_2$)]·2toluene (**3-La**) in C_6D_6.

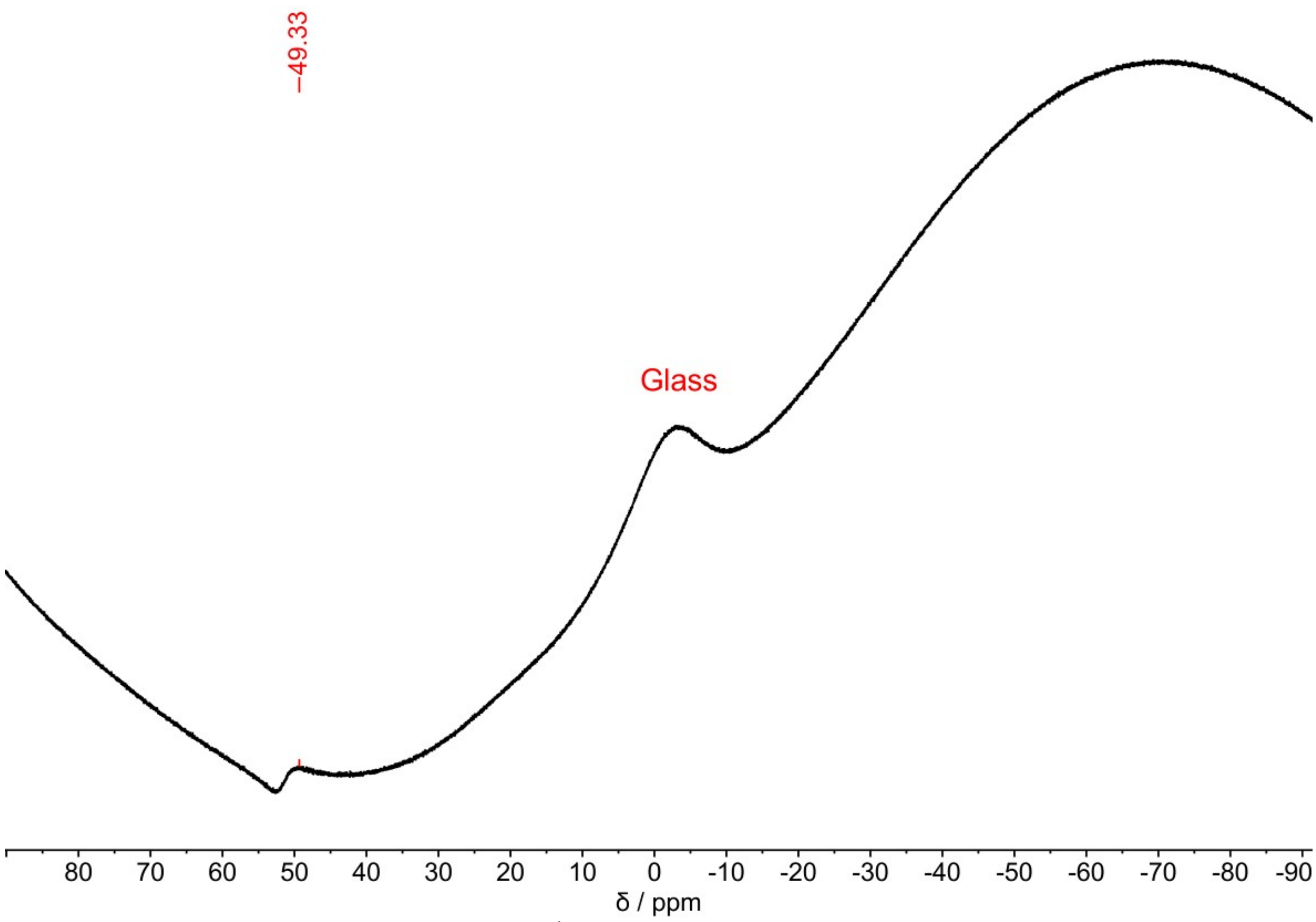

Figure S17. $^{11}B\{^1H\}$ NMR spectrum of [($C_5{}^iPr_5$)La($FcBC_2(SiMe_3)_2C_2Me_2$)]·2toluene (**3-La**) in C_6D_6.

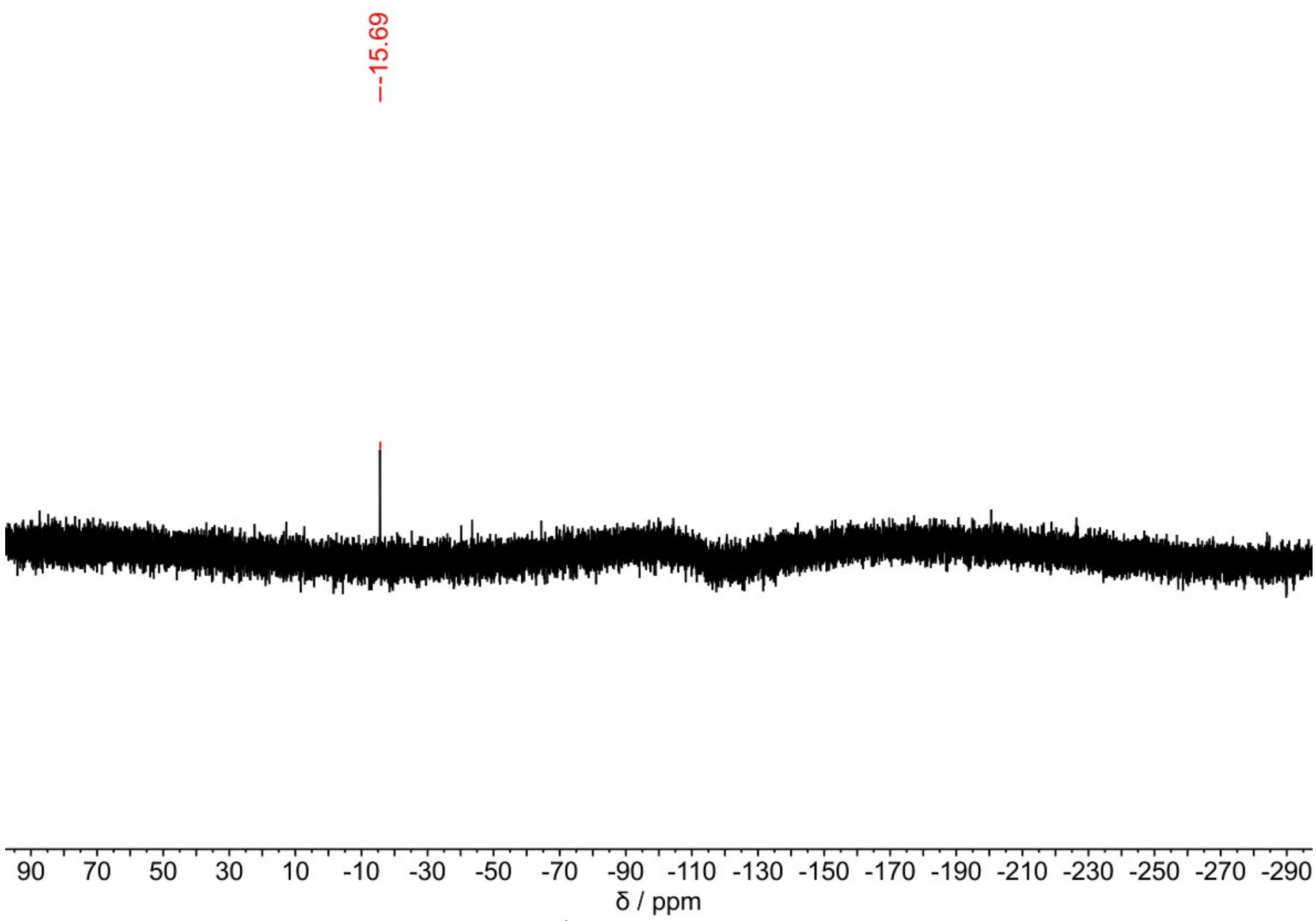

Figure S18. $^{29}Si\{^1H\}$ NMR spectrum of [($C_5{}^iPr_5$)La($FcBC_2(SiMe_3)_2C_2Me_2$)]·2toluene (**3-La**) in C_6D_6.

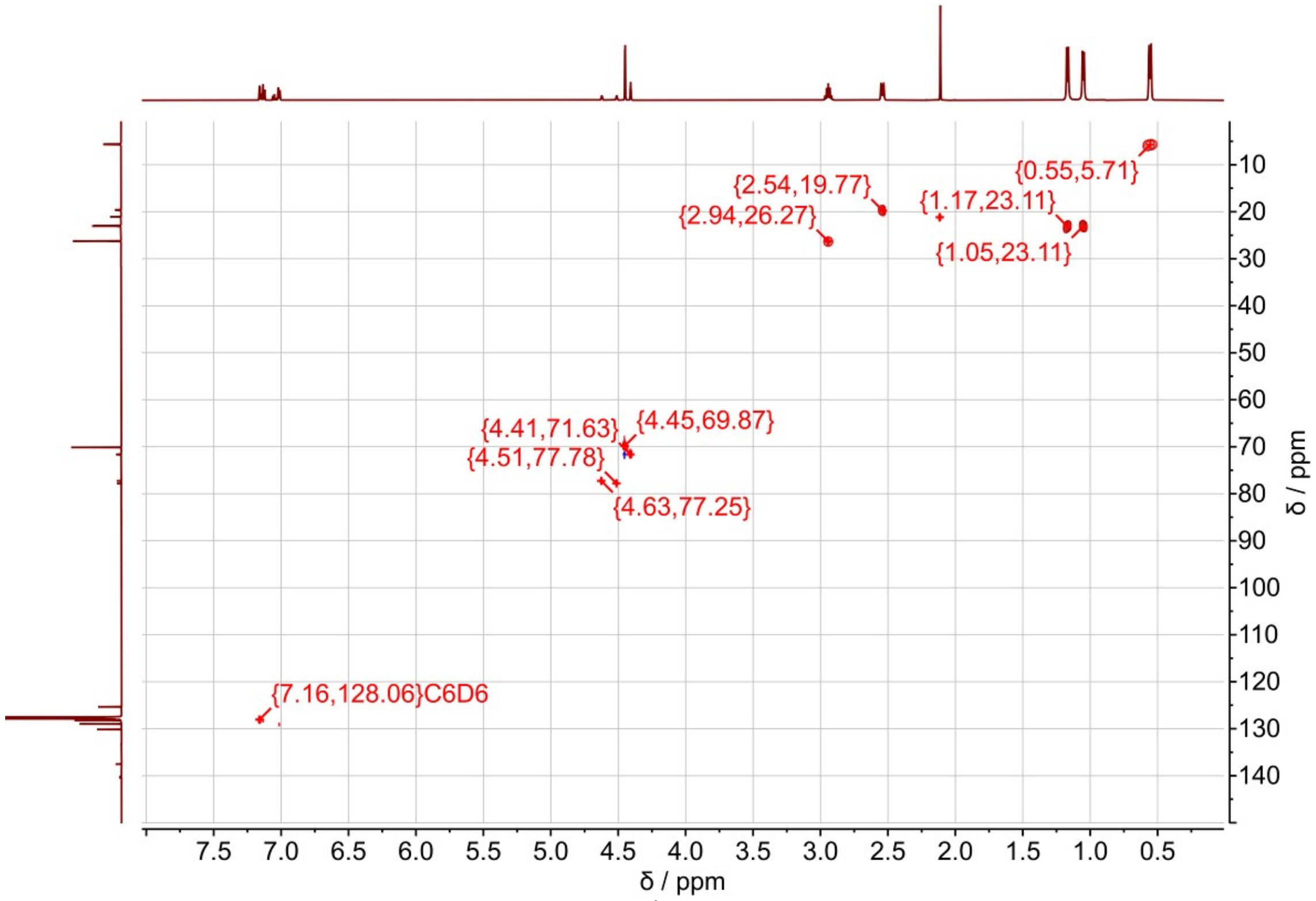

Figure S19. ^{1}H-^{13}C HSQC NMR spectrum of [($C_5{}^{i}Pr_5$)La($FcBC_2(SiMe_3)_2C_2Me_2$)]·2toluene (**3-La**) in C_6D_6.

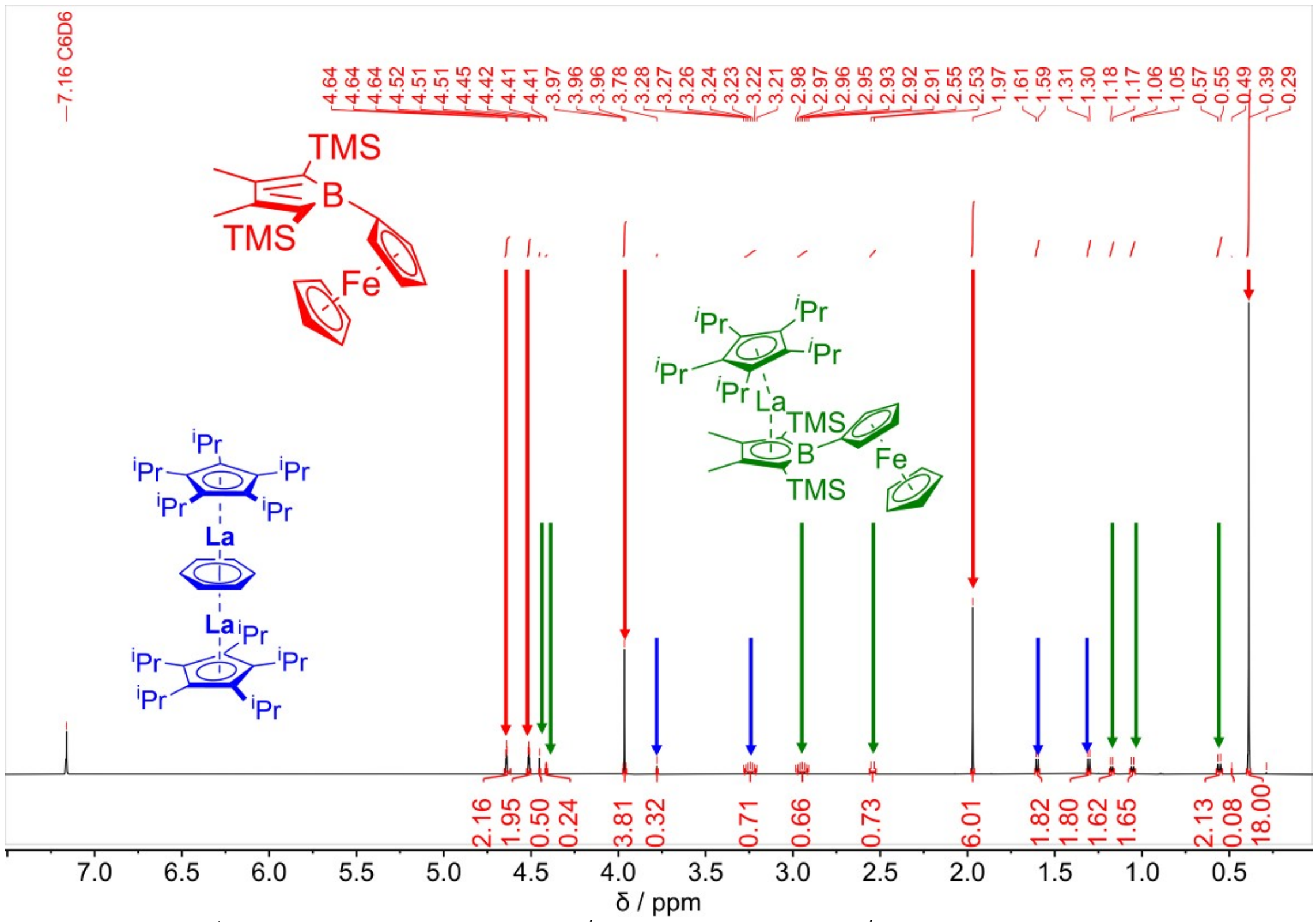

Figure S20. In-situ ^{1}H NMR spectrum of [(C$_5$^{i}Pr$_5$)La(C$_6$H$_6$)La(C$_5$^{i}Pr$_5$)] and $FcBC_2(SiMe_3)_2C_2Me_2$ at room temperature for 1 day. The colors of the arrows correspond to those of the compounds.

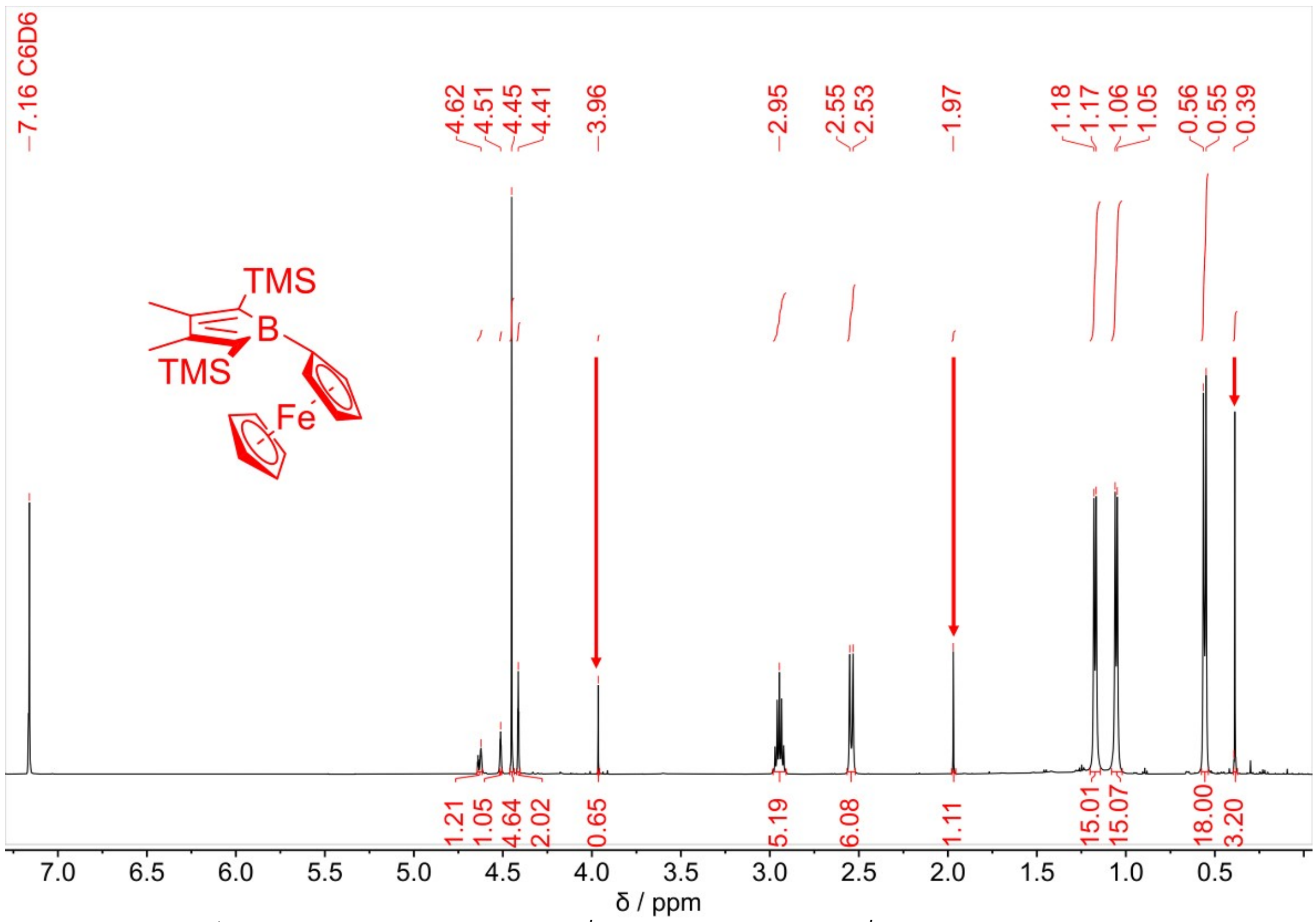

Figure S21. In-situ ^{1}H NMR spectrum of [(C$_5$^{i}Pr$_5$)La(C$_6$H$_6$)La(C$_5$^{i}Pr$_5$)] and $FcBC_2(SiMe_3)_2C_2Me_2$ at 60 °C for 1 day. Except for the three peaks of the remaining $FcBC_2(SiMe_3)_2C_2Me_2$, the other peaks are consistent with the ^{1}H NMR spectrum of **3-La**.

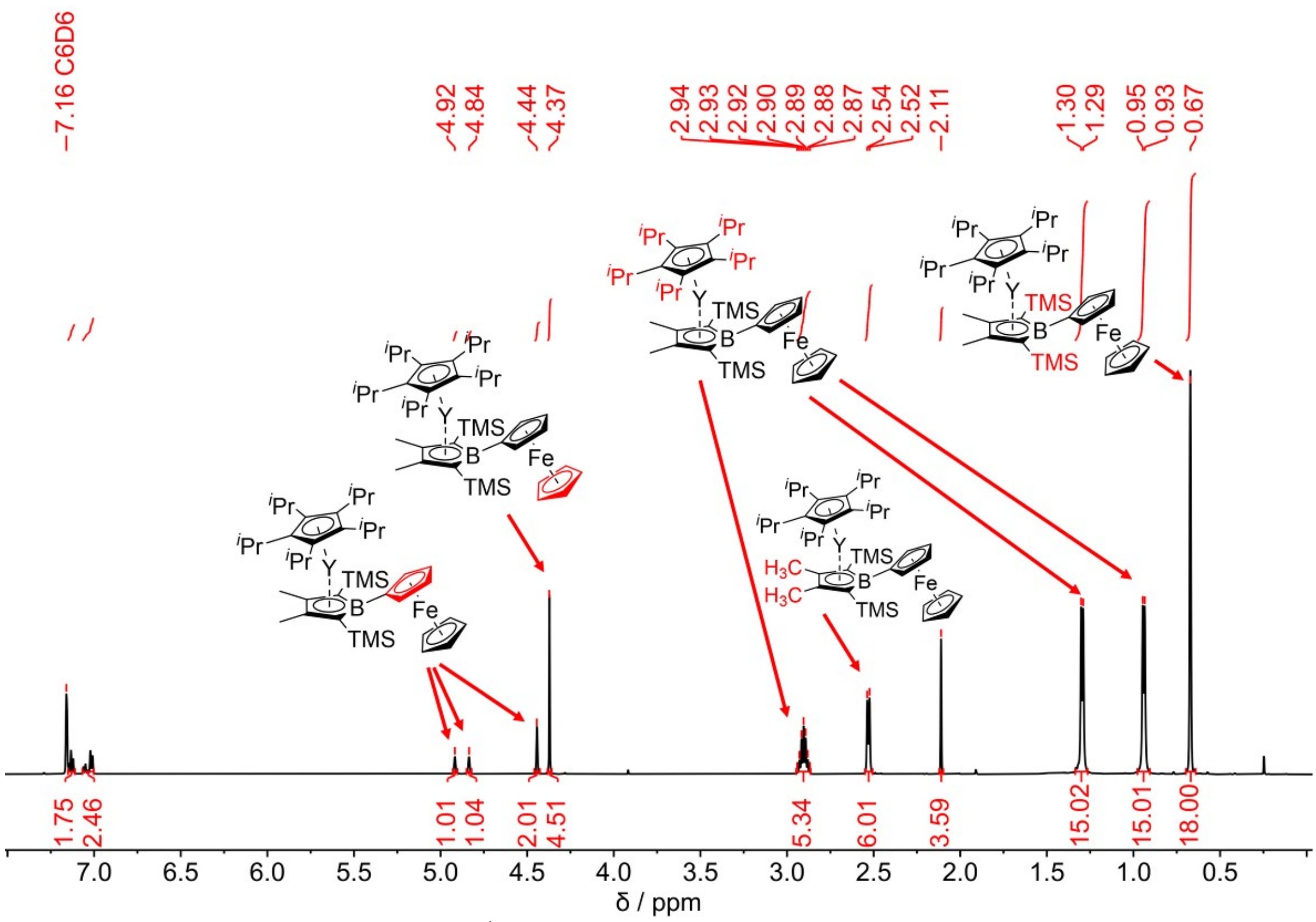

Figure S22. ^{1}H NMR spectrum of [(C$_5{}^i$Pr$_5$)Y(FcBC$_2$(SiMe$_3$)$_2$C$_2$Me$_2$)]·2toluene (**3-Y**) in C$_6$D$_6$.

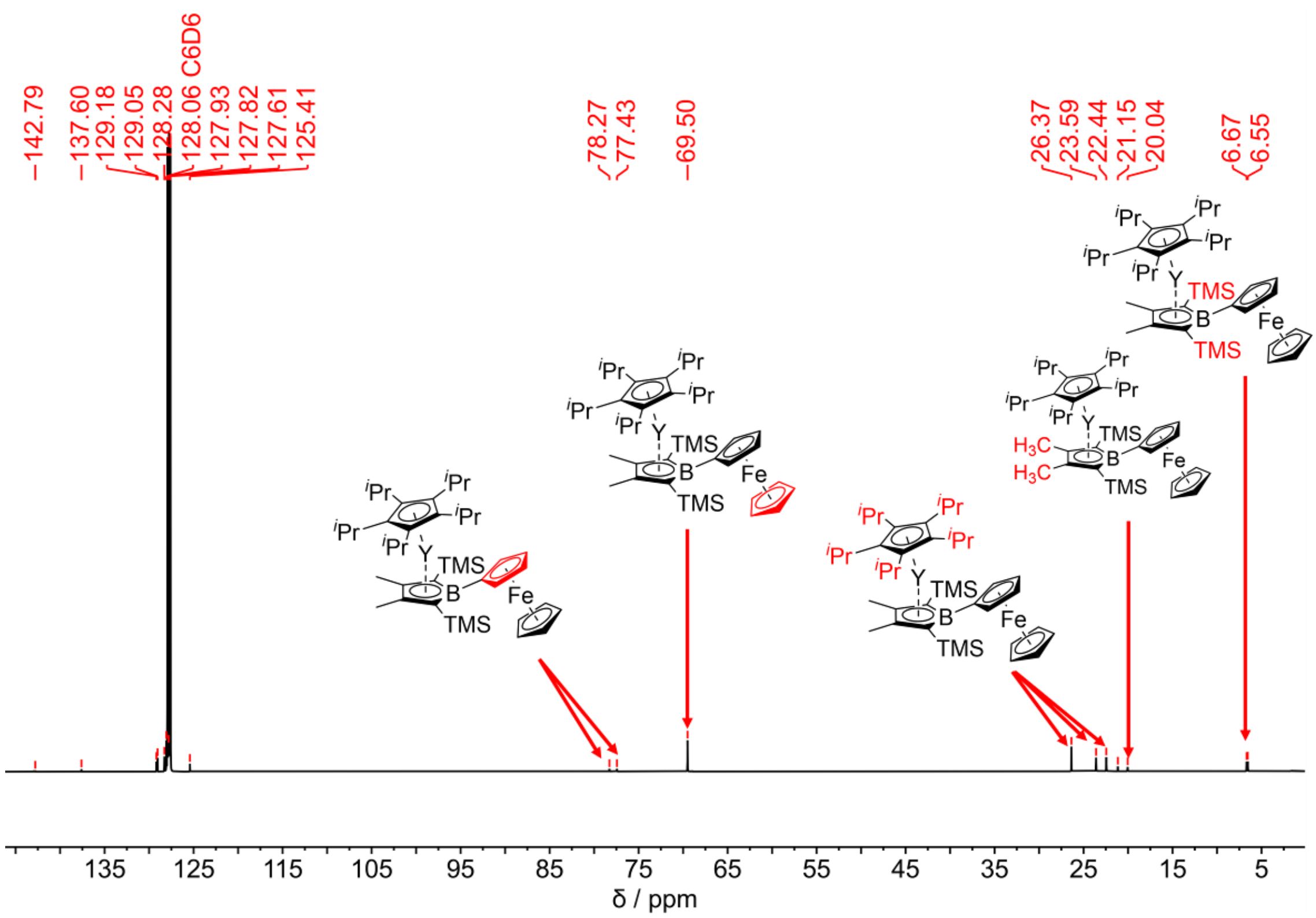

Figure S23. ^{13}C{^{1}H} NMR spectrum of [(C$_5{}^i$Pr$_5$)Y(FcBC$_2$(SiMe$_3$)$_2$C$_2$Me$_2$)]·2toluene (**3-Y**) in C$_6$D$_6$.

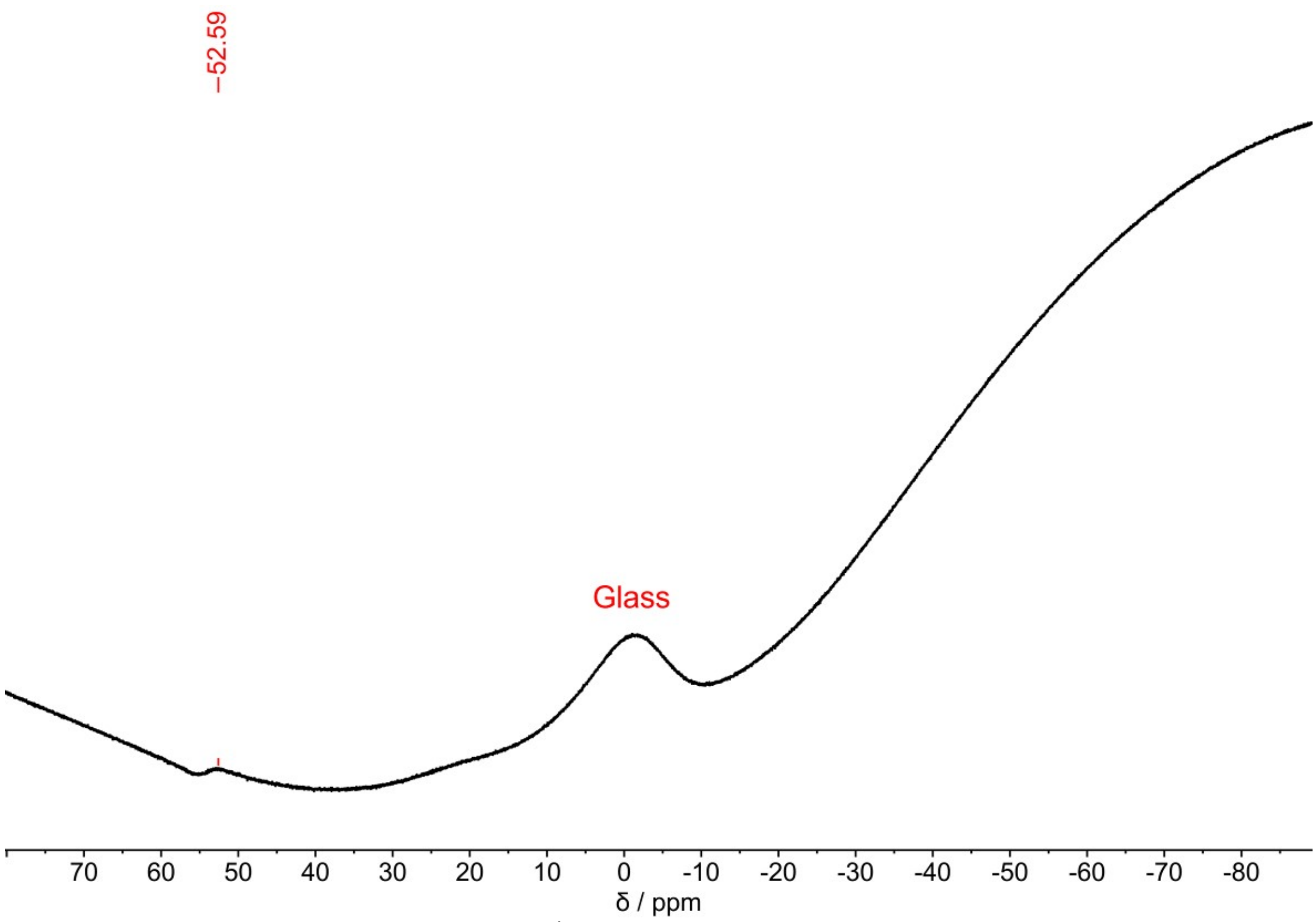

Figure S24. $^{11}B\{^1H\}$ NMR spectrum of $[(C_5{}^iPr_5)Y(FcBC_2(SiMe_3)_2C_2Me_2)]\cdot2toluene$ (**3-Y**) in C_6D_6.

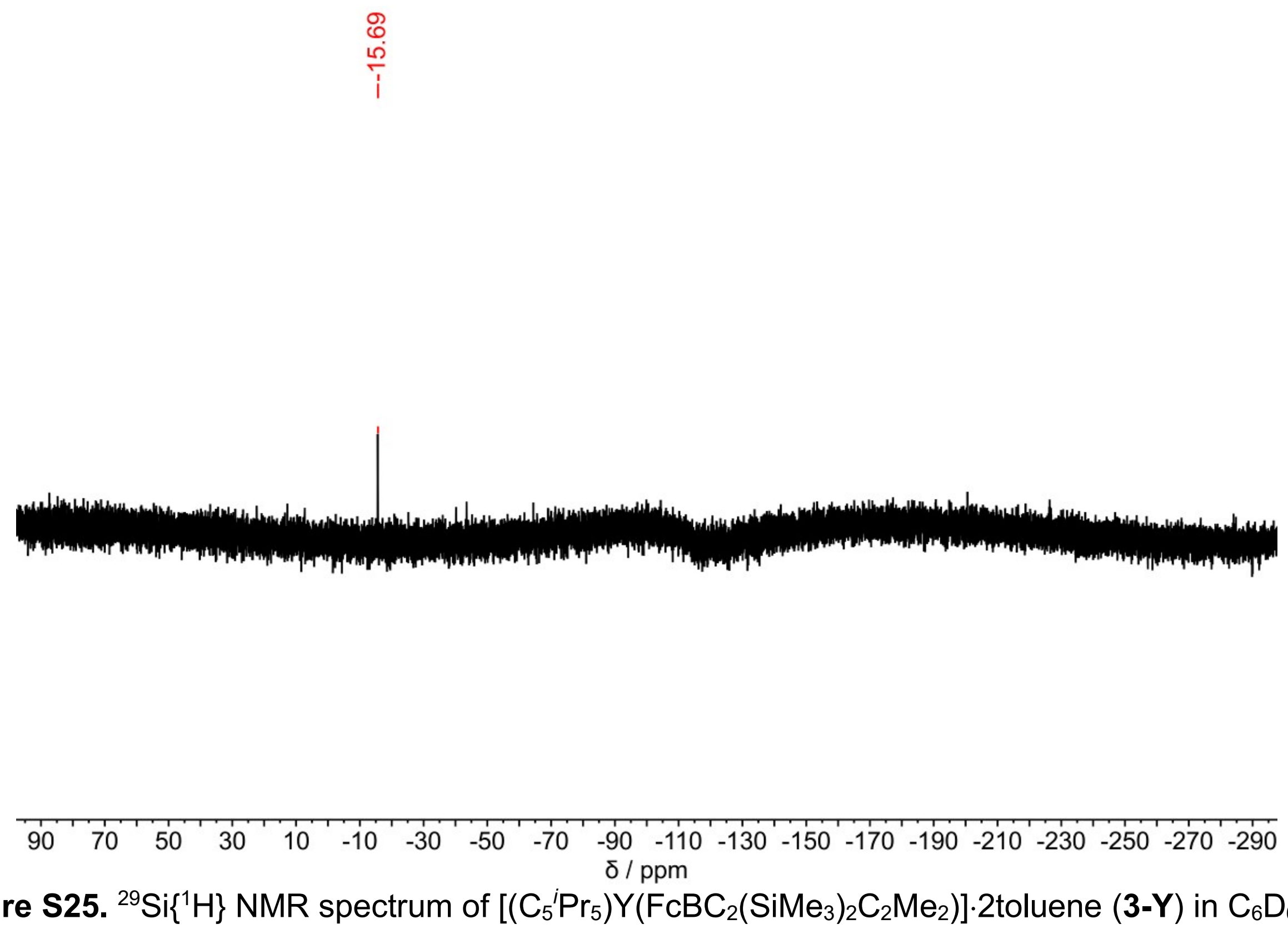

Figure S25. $^{29}Si\{^1H\}$ NMR spectrum of $[(C_5{}^iPr_5)Y(FcBC_2(SiMe_3)_2C_2Me_2)]\cdot2toluene$ (**3-Y**) in C_6D_6.

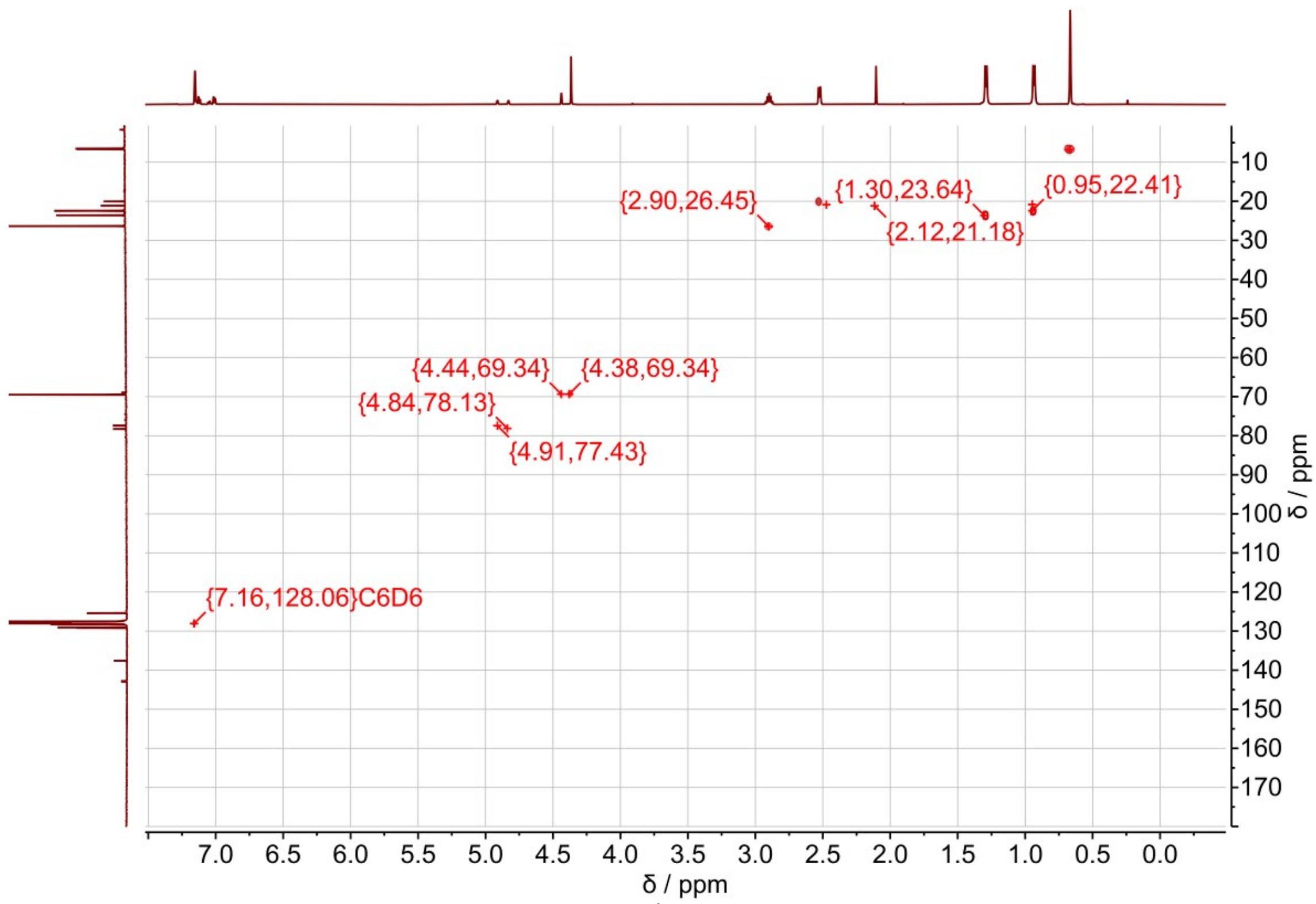

Figure S26. ^{1}H-^{13}C HSQC NMR spectrum of $[(C_5{}^{i}Pr_5)Y(FcBC_2(SiMe_3)_2C_2Me_2)]$·2toluene (**3-Y**) in C_6D_6.

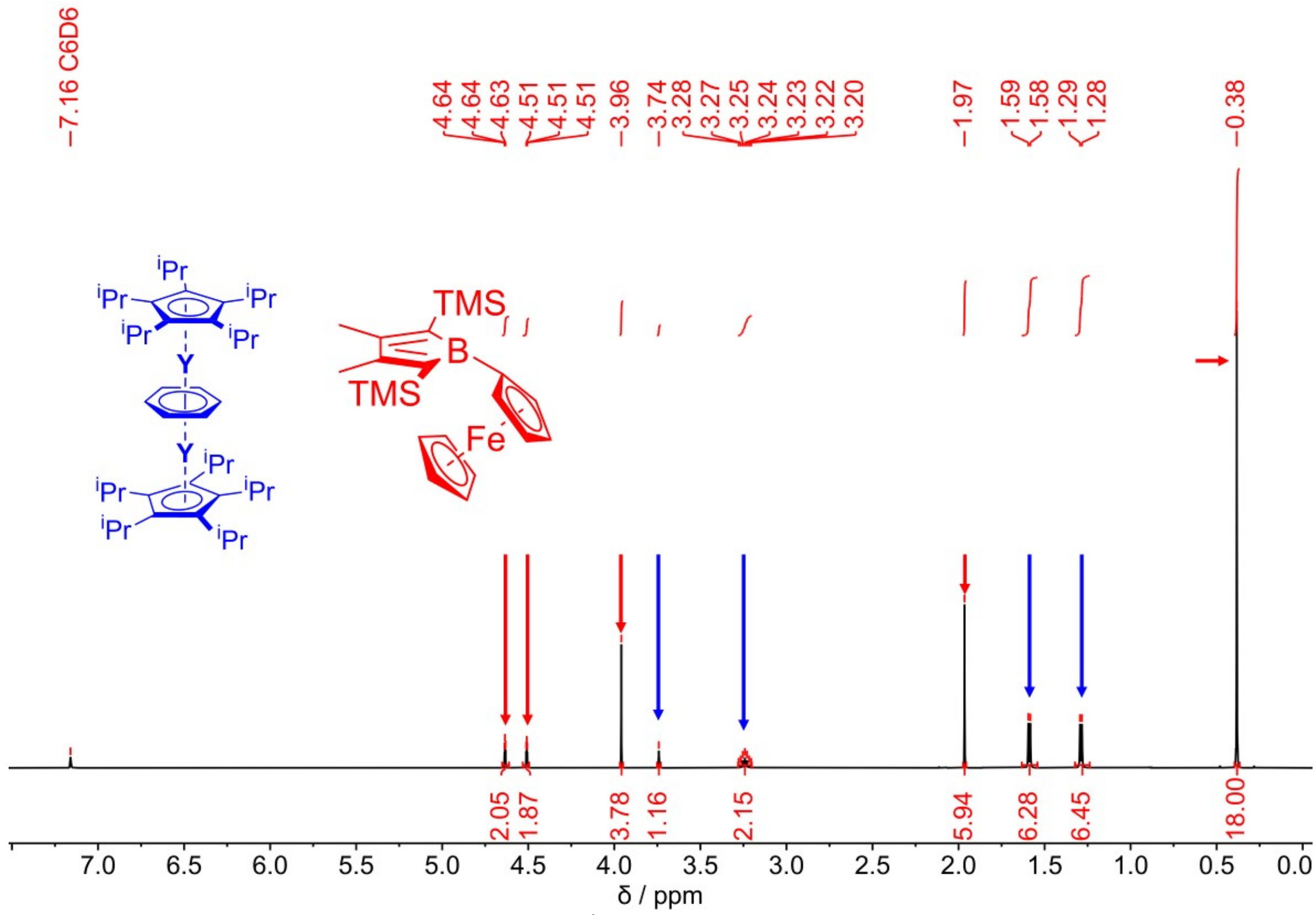

Figure S27. In-situ ^{1}H NMR spectrum of $[(C_5{}^{i}Pr_5)Y(FcBC_2(SiMe_3)_2C_2Me_2)]$·2toluene and $FcBC_2(SiMe_3)_2C_2Me_2$ at room temperature for 1 day. The colors of the arrows correspond to those of the compounds.

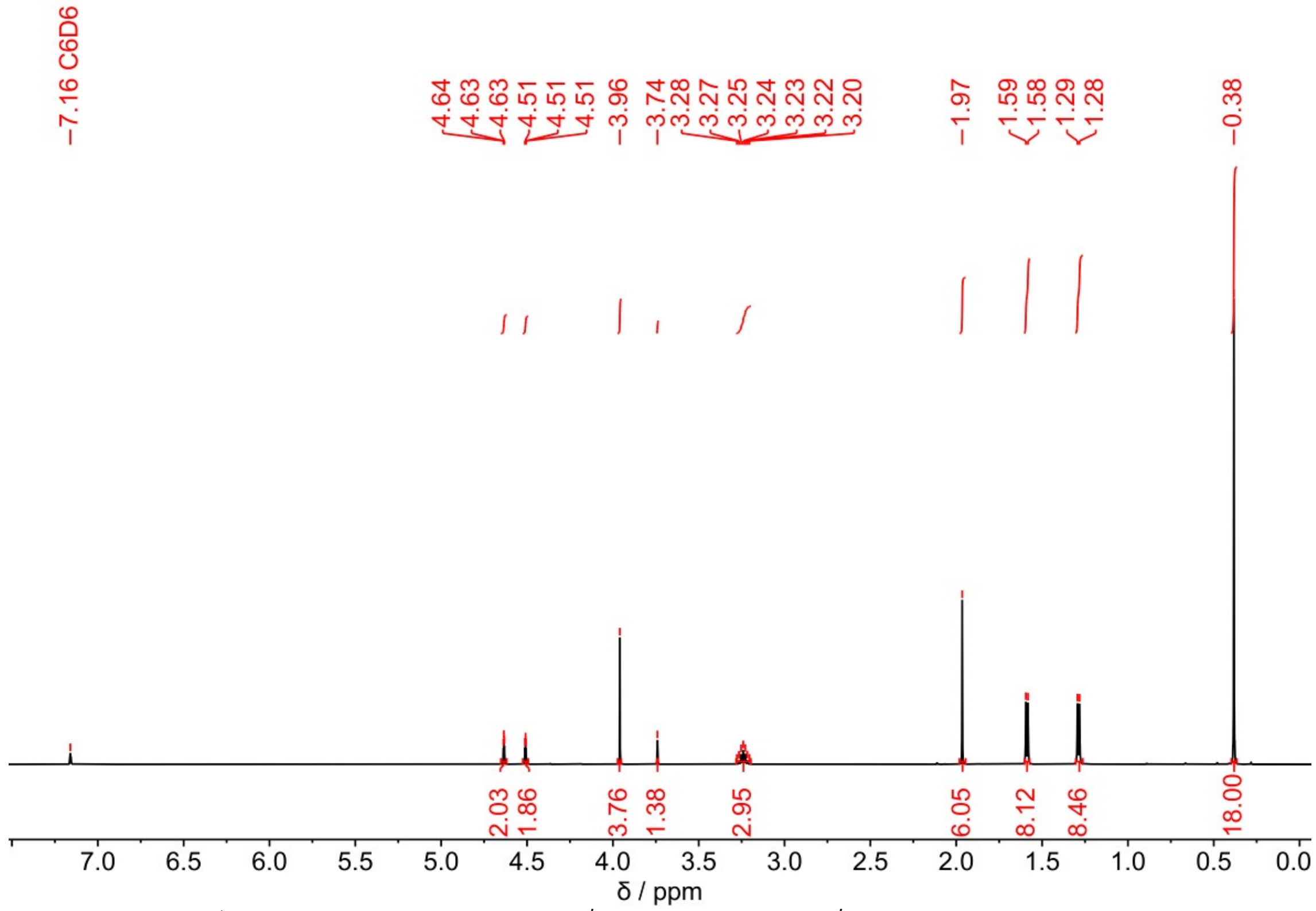

Figure S28. In-situ ^{1}H NMR spectrum of [($C_5{}^iPr_5$)Y(C_6H_6)Y($C_5{}^iPr_5$)] and $FcBC_2(SiMe_3)_2C_2Me_2$ at 60 °C for 13 days.

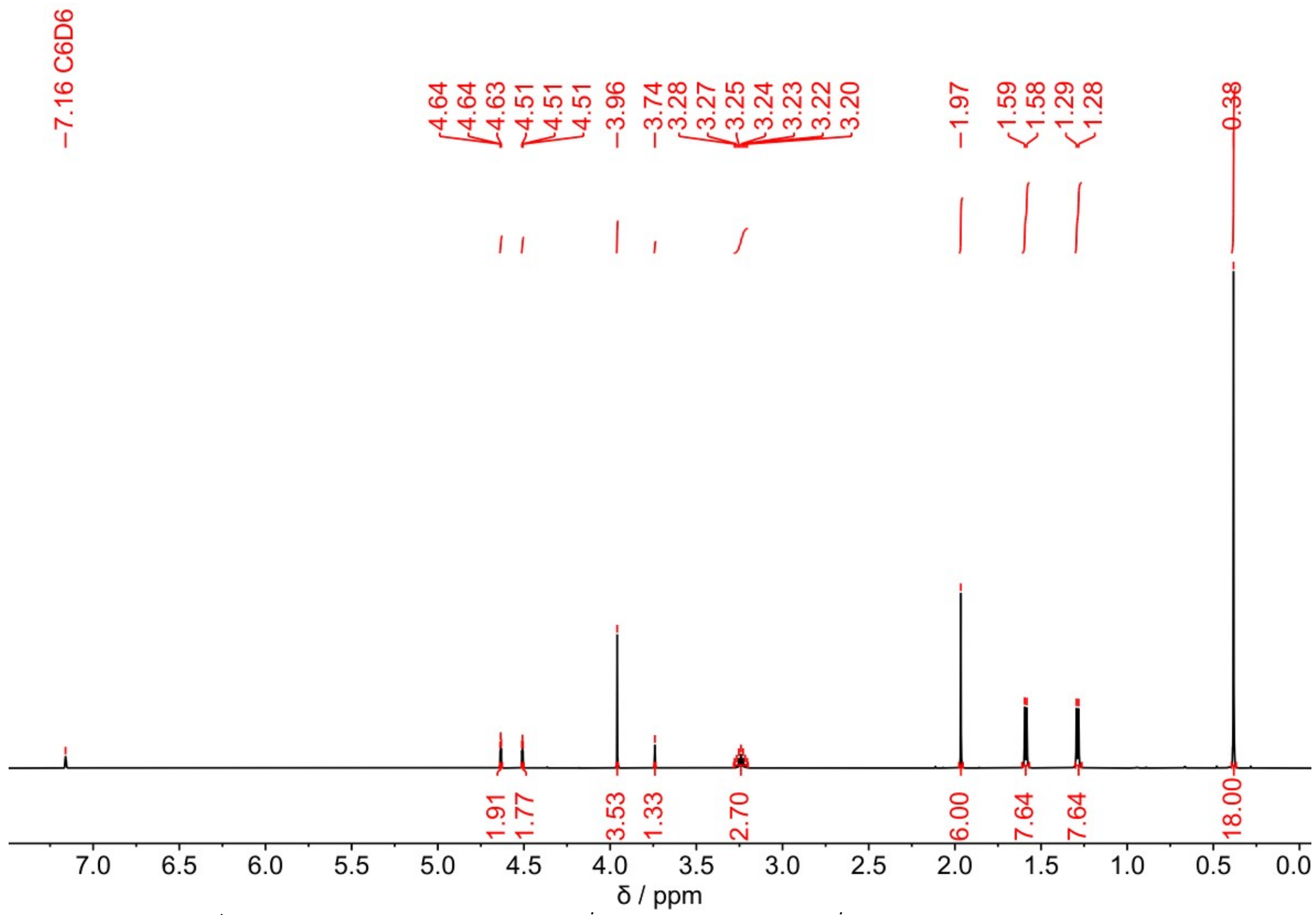

Figure S29. In-situ ^{1}H NMR spectrum of [($C_5{}^iPr_5$)Y(C_6H_6)Y($C_5{}^iPr_5$)] and $FcBC_2(SiMe_3)_2C_2Me_2$ at 60 °C for 20 days.

X-ray crystallography

X-ray diffraction data were collected on a Rigaku XtaLAB Synergy-R HyPix diffractometer for $FcBC_2(SiMe_3)_2C_2Me_2$ (**1**) and $[Mg(FcBC_2(SiMe_3)_2C_2Me_2)(THF)_3]\cdot THF$ (**2**). Data collection and processing (cell refinement, data reduction and absorption) of the two complexes were performed using the program *CrysAlisPro*. X-ray diffraction data were collected on Bruker APEX-II CCD diffractometer for $[(C_5{}^iPr_5)RE(FcBC_2(SiMe_3)_2C_2Me_2)]\cdot 2$toluene (**3-RE**, RE = Y, La, Dy). Data collection and processing (cell refinement, data reduction and absorption) were performed using the program *APEX 5*. Structures were solved in Olex2 with SHELXT using intrinsic phasing and were refined with SHELXL using least squares minimisation.[5-7] Anisotropic thermal parameters were used for the non-hydrogen atoms and isotropic parameters for the hydrogen atoms. Hydrogen atoms were added geometrically and refined using a riding model.

3-La. The $C_5{}^iPr_5$ ligand in the structure is disordered and was refined assuming all the isopropyl groups disorder over two opposite orientations, yielding a ratio of 0.745:0.255 for the two components. The distances (C6A-C1, C6-C1, C9-C2, C9A-C2, C5-C18, C5-C18A, C4-C15, C4-C15A, C3-C12A, C3-C12) between the ^{i}Pr groups and the Cp ring were fixed to be 1.54 Å using DFIX restraints. One toluene in the structure is disordered with a ratio of 0.612:0.388.

3-Dy. The $C_5{}^iPr_5$ ligand in the structure is clearly disordered and was refined assuming all the isopropyl groups disorder over two opposite orientations, yielding a ratio of 0.795:0.206 for the two components. The distances (C6A-C1 C6-C1, C9A-C2 C9-C2, C3-C12A C3-C12, C4-C15A C4-C15, C5-C18A C5-C18) between the ^{i}Pr groups and the Cp ring were fixed to be 1.54 Å using DFIX restraints. One toluene in the structure is disordered with a ratio of 0.647:0.353.

3-Y. One toluene in the structure is disordered with a ratio of 0.622:0.378.

Deposition numbers 2523312 (for **1**), 2523313 (for **2**), 2523314 (for **3-Y**), 2523315 (for **3-La**), and 2523316 (for **3-Dy**) contain the supplementary crystallographic data for this paper. These data are provided free of charge by the joint Cambridge Crystallographic Data Centre and Fachinformationszentrum Karlsruhe Access Structures service.

Table S1. Crystal data and structure refinement for $FcBC_2(SiMe_3)_2C_2Me_2$ (**1**) and $[Mg(FcBC_2(SiMe_3)_2C_2Me_2)(THF)_3]\cdot THF$ (**2**).

Compound	**1**	**2**
CCDC	2523312	2523313
Empirical formula	$C_{22}H_{33}BFeSi_2$	$C_{76}H_{130}B_2Fe_2Mg_2O_8Si_4$
Formula weight	420.32	1466.09
Crystal system	triclinic	triclinic
Space group	*P*-1	*P*-1
a/Å	7.94384(19)	11.8493(2)
b/Å	10.3049(3)	18.7451(4)
c/Å	15.3852(4)	19.5665(3)
α/°	85.255(2)	107.198(2)
β/°	76.609(2)	100.027(2)
γ/°	68.552(2)	100.210(2)
V/Å^3	1140.36(5)	3965.60(13)
Temperature/K	100	100
Z	2	2
ρ_{calc}/g cm^{-3}	1.224	1.228
Crystal size/mm^3	0.05 × 0.05 × 0.04	0.05 × 0.05 × 0.05
Radiation	Cu $K\alpha$(λ = 1.54184)	Cu $K\alpha$ (λ = 1.54184)
2θ range/°	5.906 to 153.554	4.872 to 149.952
Reflections collected	12979	50710
Independent reflections	4611 [R_{int} = 0.0206, R_{sigma} = 0.0217]	15870 [R_{int} = 0.0855, R_{sigma} = 0.0608]
Data/restraints/parameters	4611/0/243	15870/85/947
Goodness-of-fit on F^2	1.076	1.079
Final R indexes [I >=2σ (I)]	R_1 = 0.0281, wR_2 = 0.0756	R_1 = 0.0658, wR_2 = 0.1580
Final R indexes [all data]	R_1 = 0.0289, wR_2 = 0.0759	R_1 = 0.0953, wR_2 = 0.1782

Table S2. Crystal data and structure refinement for [($C_5{}^iPr_5$)Y($FcBC_2(SiMe_3)_2C_2Me_2$)]·2toluene (**3-Y**), [($C_5{}^iPr_5$)La($FcBC_2(SiMe_3)_2C_2Me_2$)]·2toluene (**3-La**) and [($C_5{}^iPr_5$)Dy($FcBC_2(SiMe_3)_2C_2Me_2$)]·2toluene (**3-Dy**).

Compound	**3-Y**	**3-La**	**3-Dy**
CCDC	2523314	2523315	2523316
Empirical formula	$C_{56}H_{84}BFeSi_2Y$	$C_{56}H_{84}BFeLaSi_2$	$C_{56}H_{84}BDyFeSi_2$
Formula weight	968.98	1018.98	1042.57
Crystal system	monoclinic	monoclinic	monoclinic
Space group	$P2_1/n$	$P2_1/n$	$P2_1/n$
a/Å	11.3619(5)	11.2558(7)	11.3651(8)
b/Å	19.0740(9)	19.0204(12)	19.0491(14)
c/Å	24.2987(11)	24.7712(15)	24.3354(18)
α/°	90	90	90
β/°	97.213(2)	96.454(2)	97.156(3)
γ/°	90	90	90
V/Å^3	5224.3(4)	5269.7(6)	5227.5(7)
Temperature/K	100	100	100
Z	4	4	4
ρ_{calc}/g cm^{-3}	1.232	1.284	1.325
Crystal size/mm^3	0.33 × 0.28 × 0.12	0.4 × 0.3 × 0.2	0.1 × 0.05 × 0.02
Radiation	Mo $K\alpha$ (λ = 0.71073)	Mo $K\alpha$ (λ = 0.71073)	Mo $K\alpha$ (λ = 0.71073)
2θ range/°	3.792 to 56.68	3.826 to 66.544	3.792 to 61.156
Reflections collected	100817	210428	136803
Independent reflections	13034 [R_{int} = 0.0603, R_{sigma} = 0.0371]	20294 [R_{int} = 0.0563, R_{sigma} = 0.0254]	16037 [R_{int} = 0.0539, R_{sigma} = 0.0288]
Data/restraints/parameters	13034/0/635	20294/10/681	16037/10/681
Goodness-of-fit on F^2	1.035	1.060	1.060
Final R indexes [I >=2σ (I)]	R_1 = 0.0459, wR_2 = 0.1098	R_1 = 0.0336, wR_2 = 0.0740	R_1 = 0.0301, wR_2 = 0.0706
Final R indexes [all data]	R_1 = 0.0669, wR_2 = 0.1210	R_1 = 0.0397, wR_2 = 0.0771	R_1 = 0.0392, wR_2 = 0.0751

Table S3. Selected bond lengths for $FcBC_2(SiMe_3)_2C_2Me_2$ (**1**).

Bond	Length/Å
C1-C2	1.354(2)
C1-B1	1.590(2)
C2-C3	1.517(2)
C3-C4	1.359(2)
C4-B1	1.594(2)
C13-B1	1.540(2)

Table S4. Selected bond lengths for $[Mg(FcBC_2(SiMe_3)_2C_2Me_2)(THF)_3]\cdot THF$ (**2**).

Bond	Length/Å	Bond	Length/Å
Mg1-C1	2.359(4)	Mg2-C35	2.348(4)
Mg1-C2	2.373(4)	Mg2-C36	2.385(4)
Mg1-C3	2.377(4)	Mg2-C37	2.383(4)
Mg1-C4	2.370(5)	Mg2-C38	2.367(4)
Mg1-B1	2.425(5)	Mg2-B2	2.416(4)
Mg1-C_4B(centroid)	2.0179(14)	Mg2-C_4B(centroid)	2.0152(13)
C1-C2	1.459(5)	C35-C36	1.462(5)
C1-C13	1.557(6)	C35-B2	1.565(5)
C2-C3	1.393(7)	C36-C37	1.392(5)
C3-C4	1.475(6)	C37-C38	1.471(5)
C4-B1	1.549(5)	C38-B2	1.558(5)

Table S5. Selected bond lengths (Å) and angle (degree) for [($C_5{}^iPr_5$)La(FcBC_2($SiMe_3$)$_2C_2Me_2$)]·2toluene (**3-La**).

**Bond**	**Length/Å**
La1-C1	2.8188(15)
La1-C2	2.8916(16)
La1-C3	2.8727(16)
La1-C4	2.7884(17)
La1-C5	2.7572(15)
La1-$C_5{}^iPr_5$(centroid)	2.5562(4)
La1-B1	2.6571(17)
La1-C21	2.6990(14)
La1-C22	2.7639(14)
La1-C23	2.7719(15)
La1-C24	2.6931(16)
La1-C_4B(centroid)	2.4067(4)

**Angle**	**degree**
$C_5{}^iPr_5$(centroid)-La1-C_4B(centroid)	152.215(16)

Table S6. Selected bond lengths (Å) and angle (deg) for $[(C_5{}^iPr_5)La(FcBC_2(SiMe_3)_2C_2Me_2)]\cdot$2toluene (**3-Dy**).

**Bond**	**Length/Å**
Dy1-C1	2.6477(19)
Dy1-C2	2.7277(19)
Dy1-C3	2.705(2)
Dy1-C4	2.614(2)
Dy1-C5	2.580(2)
Dy1-$C_5{}^iPr_5$(centroid)	2.3654(1)
Dy1-B1	2.575(2)
Dy1-C21	2.5577(18)
Dy1-C22	2.5892(19)
Dy1-C23	2.5886(19)
Dy1-C24	2.551(2)
Dy1-C_4B(centroid)	2.2406(1)

**Angle**	**degree**
$C_5{}^iPr_5$(centroid)-Dy-C_4B(centroid)	157.345(18)

Table S7. Selected bond lengths (Å) and angle (deg) for $[(C_5{}^iPr_5)La(FcBC_2(SiMe_3)_2C_2Me_2)]\cdot$2toluene (**3-Y**).

**Bond**	**Length/Å**
Y1-C1	2.579(3)
Y1-C2	2.612(3)
Y1-C3	2.697(3)
Y1-C4	2.712(3)
Y1-C5	2.632(2)
Y1-$C_5{}^iPr_5$(centroid)	2.3570(4)
Y1-B1	2.568(3)
Y1-C21	2.533(3)
Y1-C22	2.579(3)
Y1-C23	2.582(2)
Y1-C24	2.549(2)
Y1-C_4B(centroid)	2.2300(4)

**Angle**	**degree**
$C_5{}^iPr_5$(centroid)-Y1-C_4B(centroid)	157.685(18)

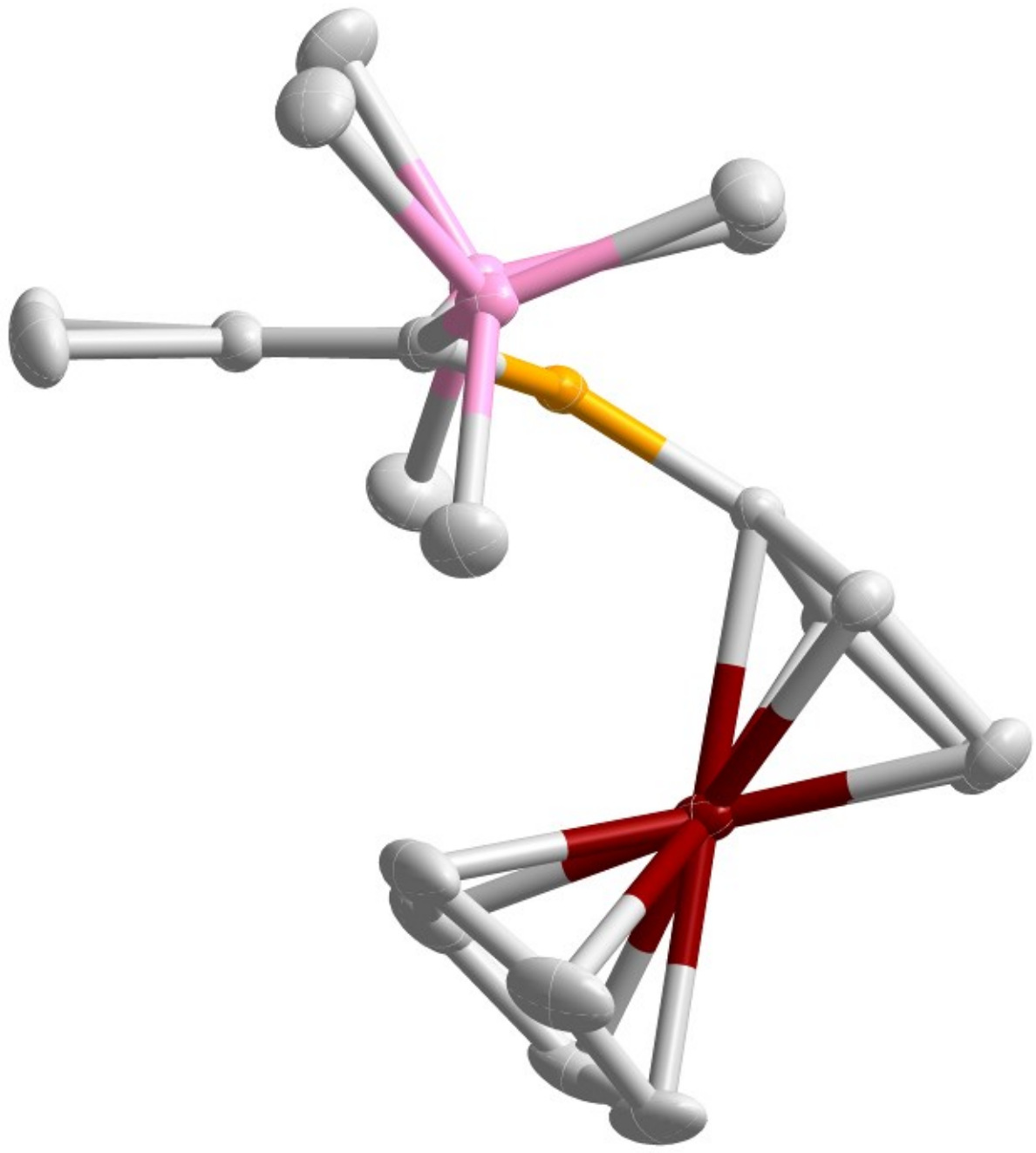

Figure S30. Molecular structure of compound **1** (sideview). Hydrogen atoms were omitted for clarity. C: grey; B: orange; Si: rose; Fe: dark red.

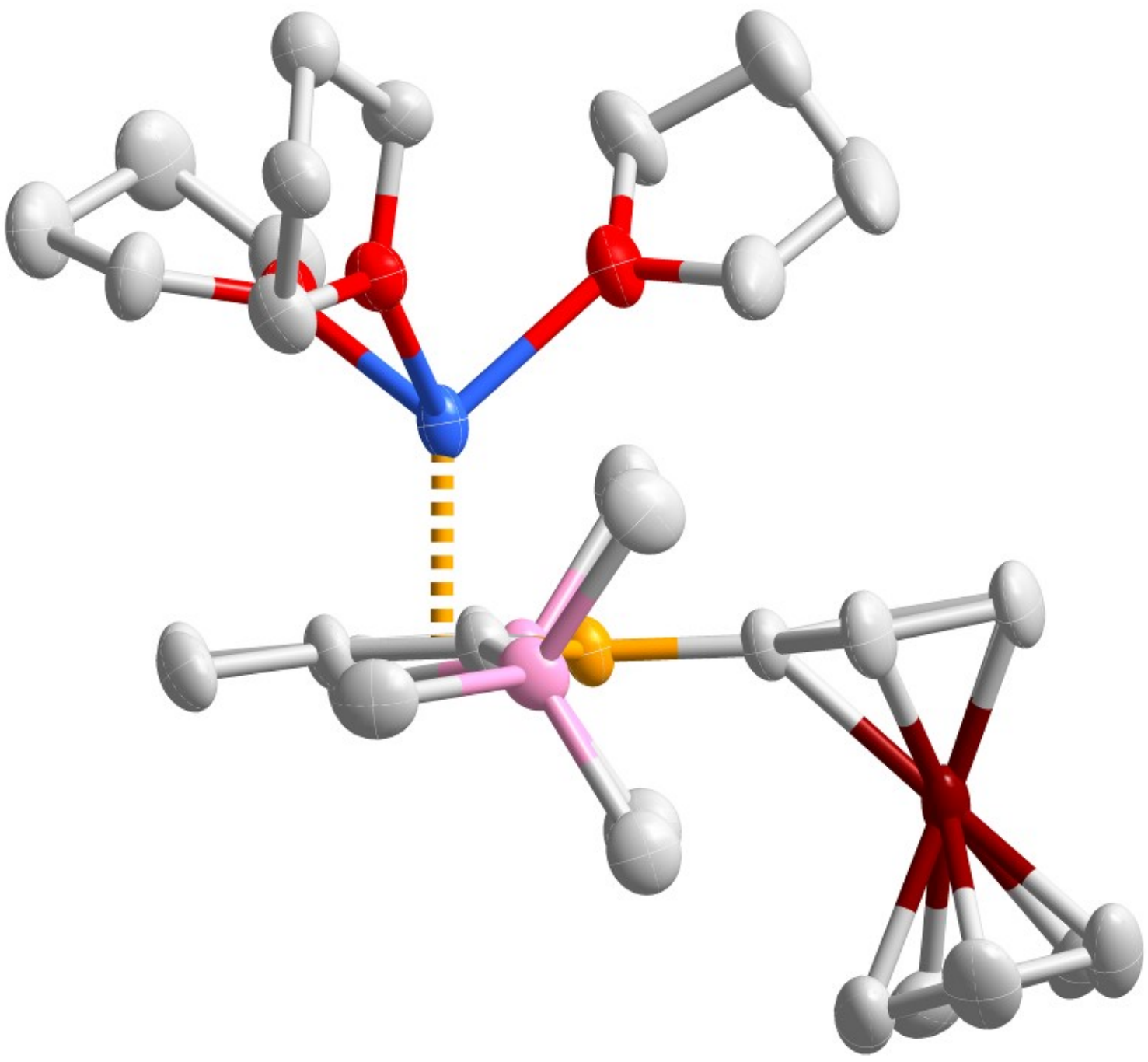

Figure S31. Molecular structure of compound **2** (sideview). Hydrogen atoms were omitted for clarity. C: grey; O: red; B: orange; Si: rose; Fe: dark red; Mg: blue.

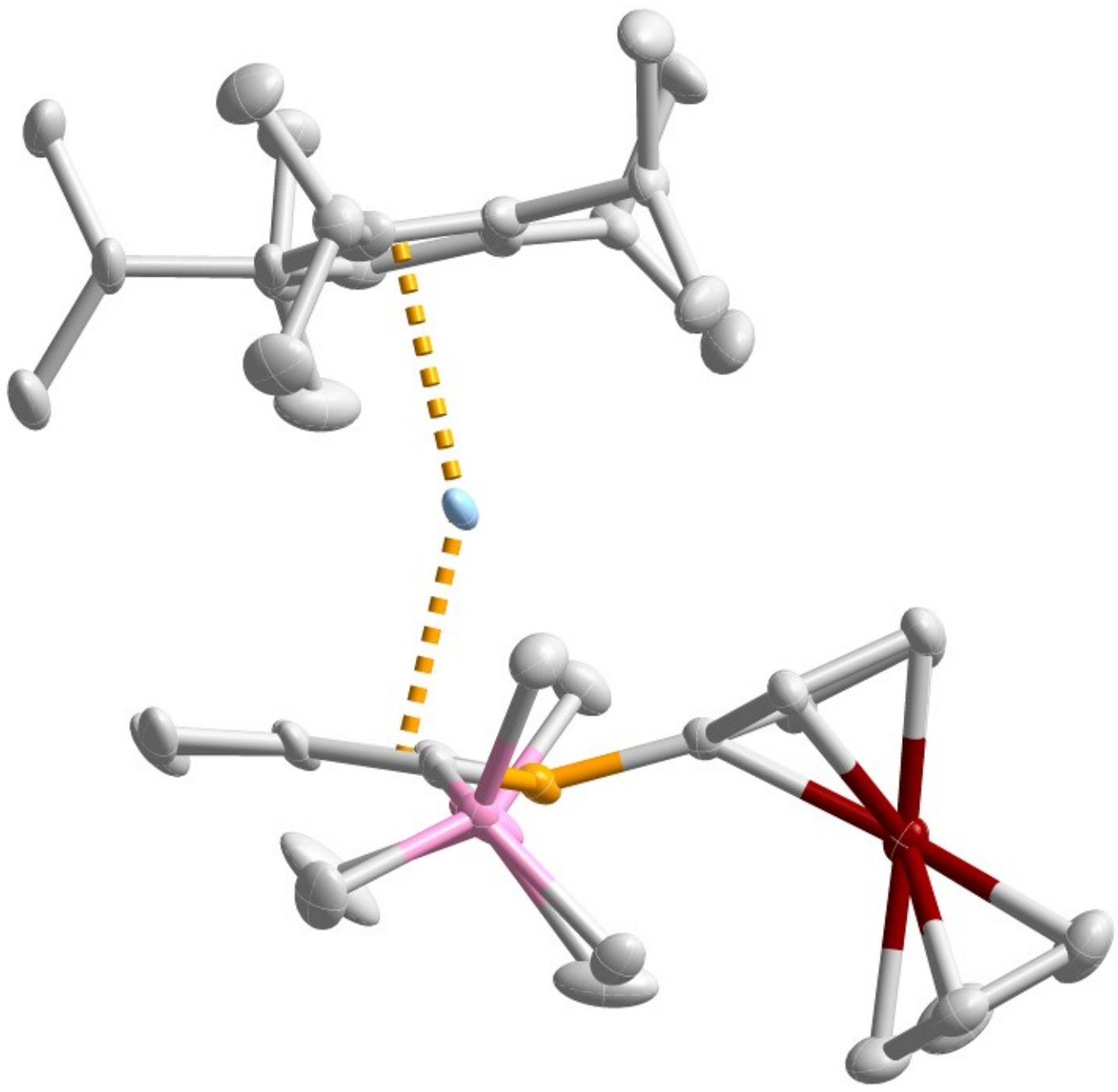

Figure S32. Molecular structure of compound **3-La** (sideview). Hydrogen atoms were omitted for clarity. C: grey; B: orange; Si: rose; Fe: dark red; La: pale bule.

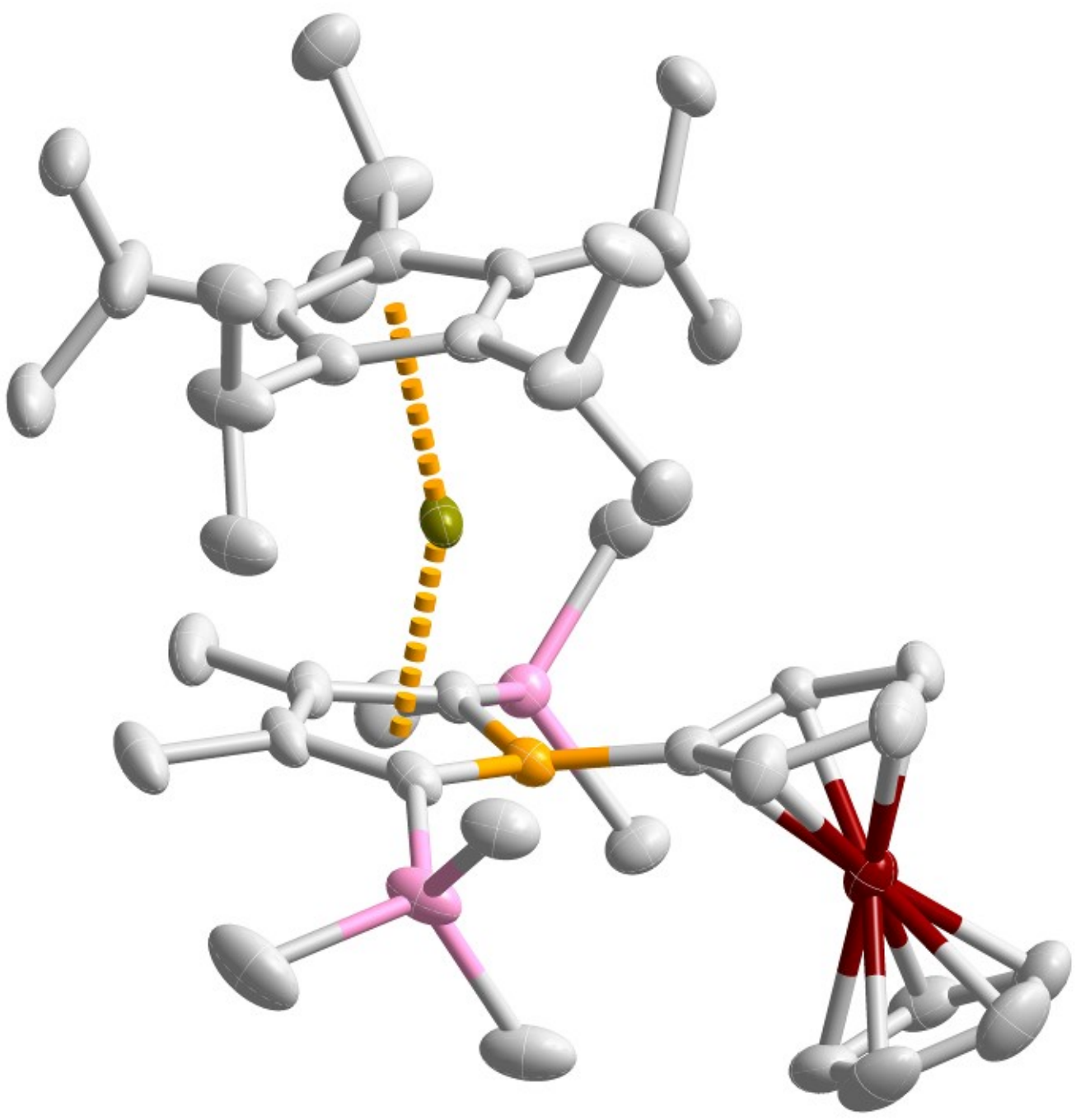

Figure S33. Molecular structure of compound **3-Y**. Hydrogen atoms were omitted for clarity. C: grey; B: orange; Si: rose; Fe: dark red; Y: dark yellow.

UV/vis/NIR Spectroscopy

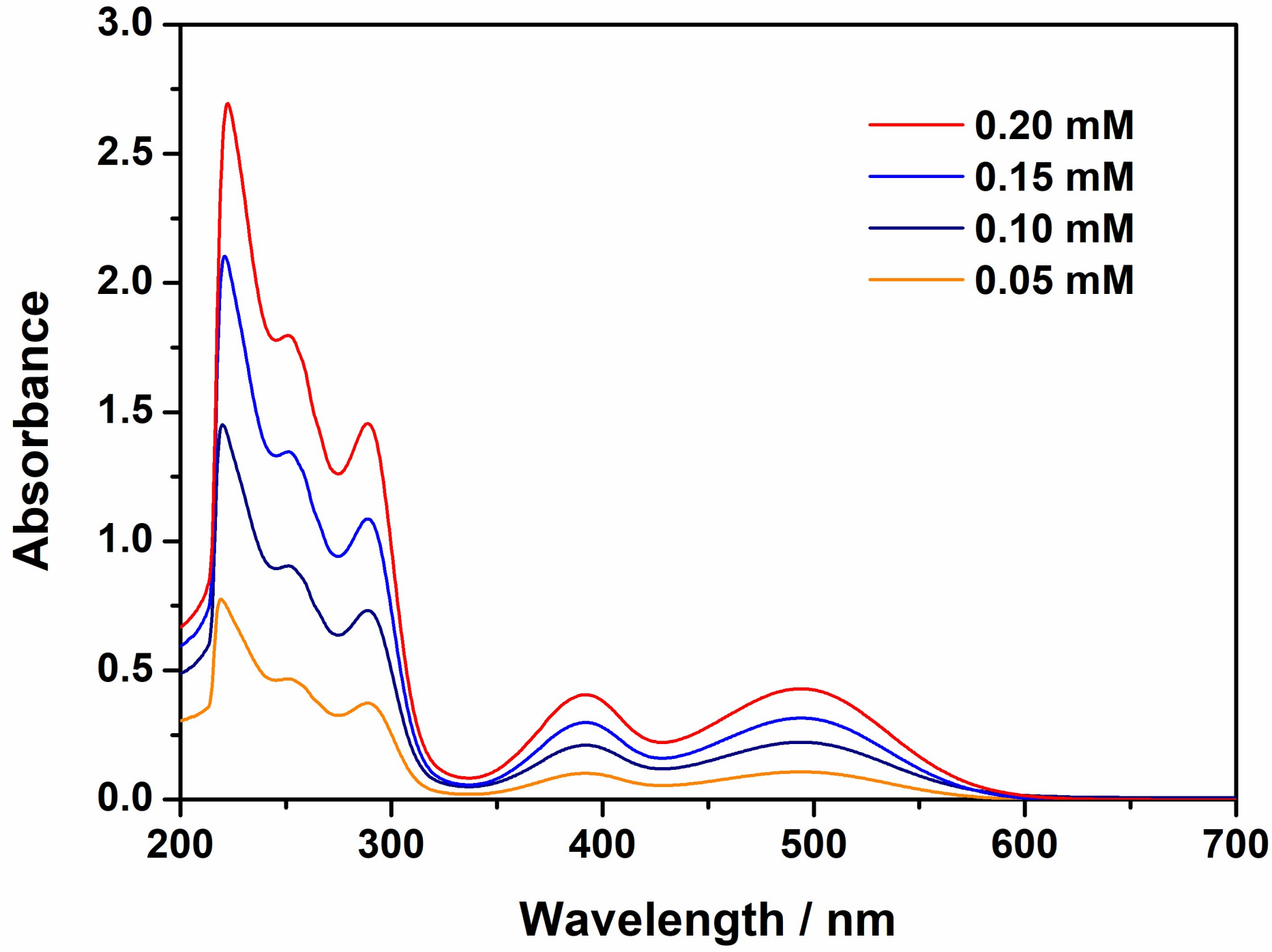

Figure S34. UV-vis-NIR spectrum of $FcBC_2(SiMe_3)_2C_2Me_2$ (**1**) in hexane at different concentrations. Significant absorptions occur at λ = 221, 251, 289, 391 and 494 nm.

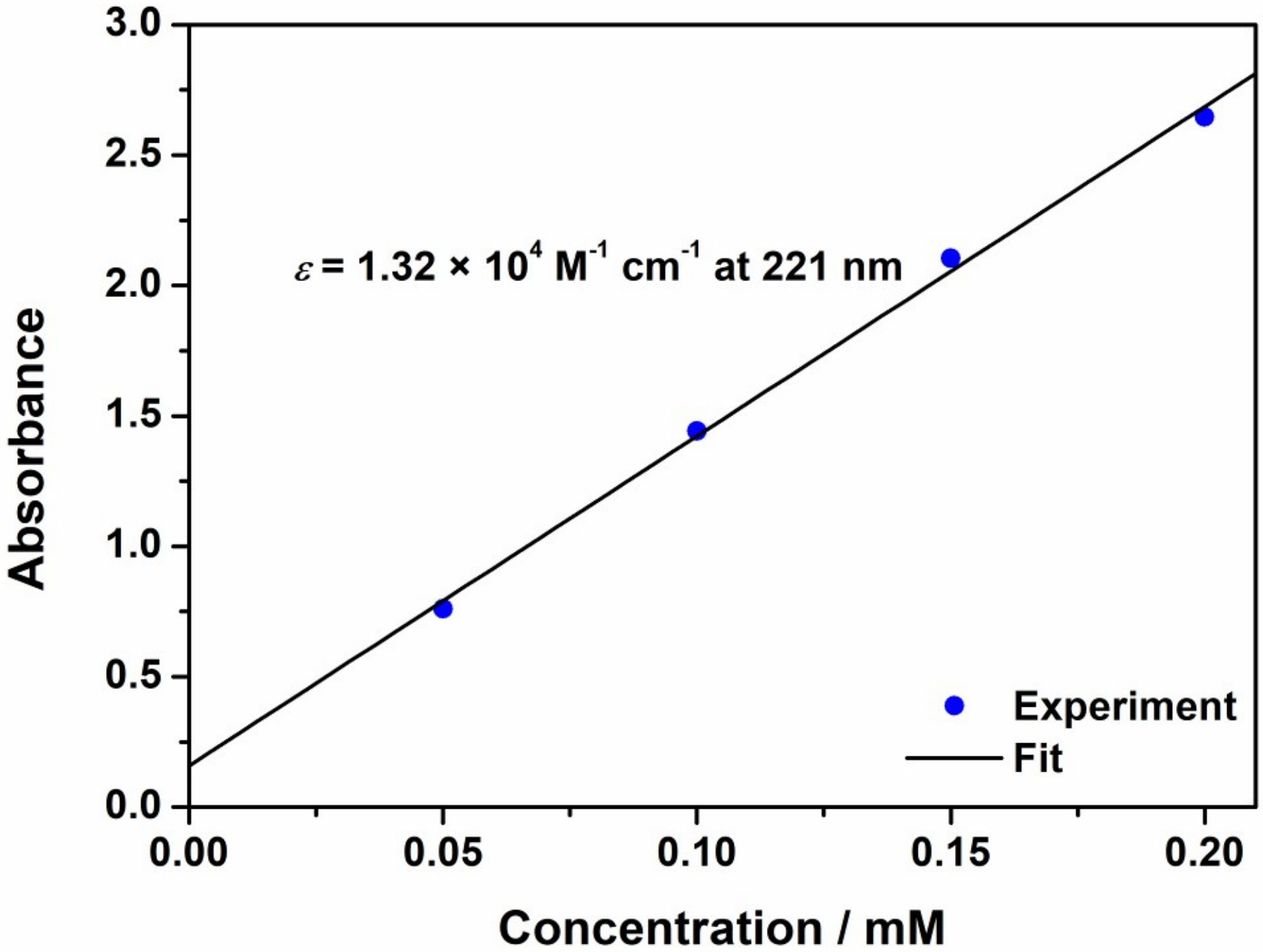

Figure S35. Plot of absorbance versus concentration for $FcBC_2(SiMe_3)_2C_2Me_2$ (**1**) at 221 nm in the UV-vis-NIR spectrum. The blue points are from the UV-vis-NIR spectrum (Figure S34). The solid black line is the best fit to the data to extract the extinction coefficient.

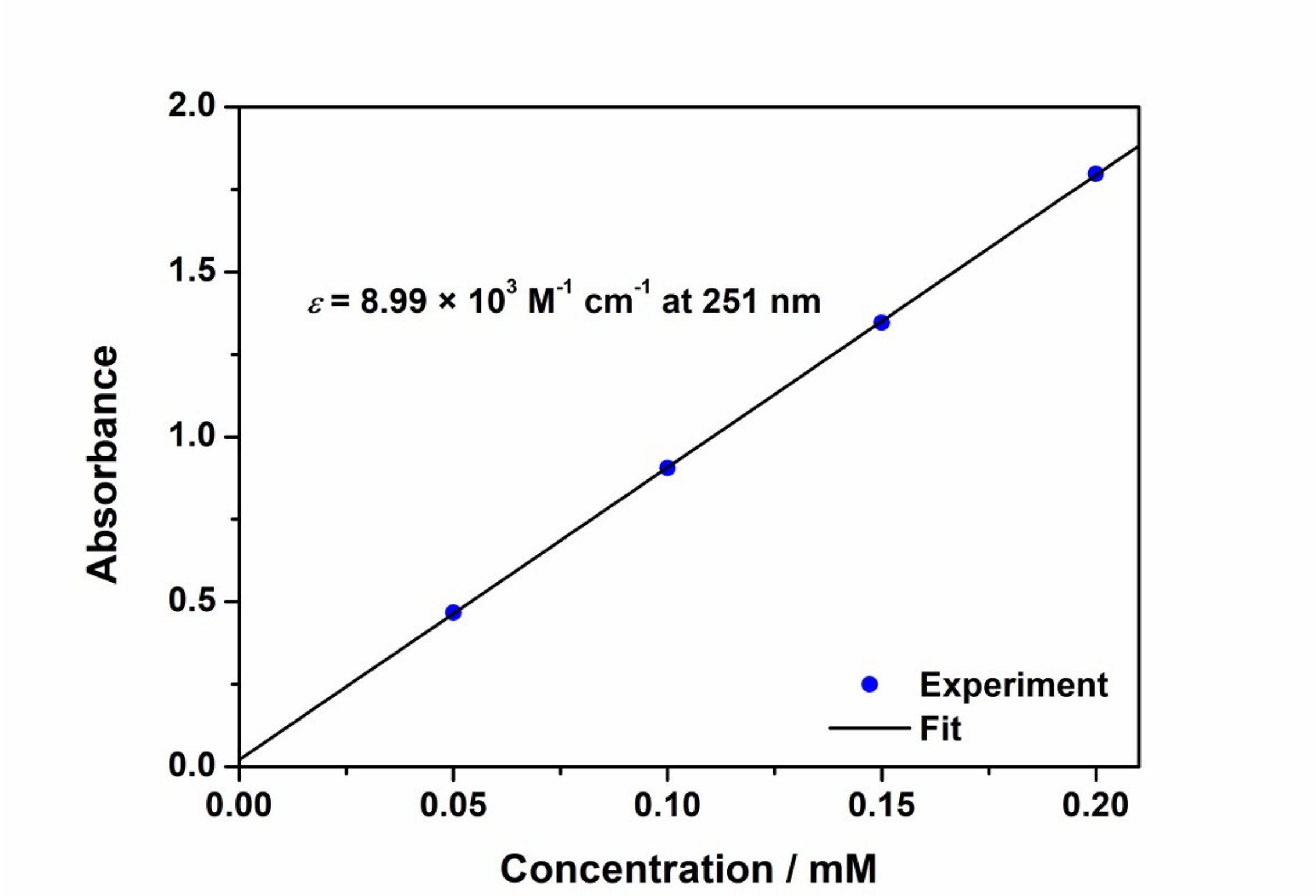

Figure S36. Plot of absorbance versus concentration for $FcBC_2(SiMe_3)_2C_2Me_2$ (**1**) at 251 nm in the UV-vis-NIR spectrum. The blue points are from the UV-vis-NIR spectrum (Figure S34). The solid black line is the best fit to the data to extract the extinction coefficient.

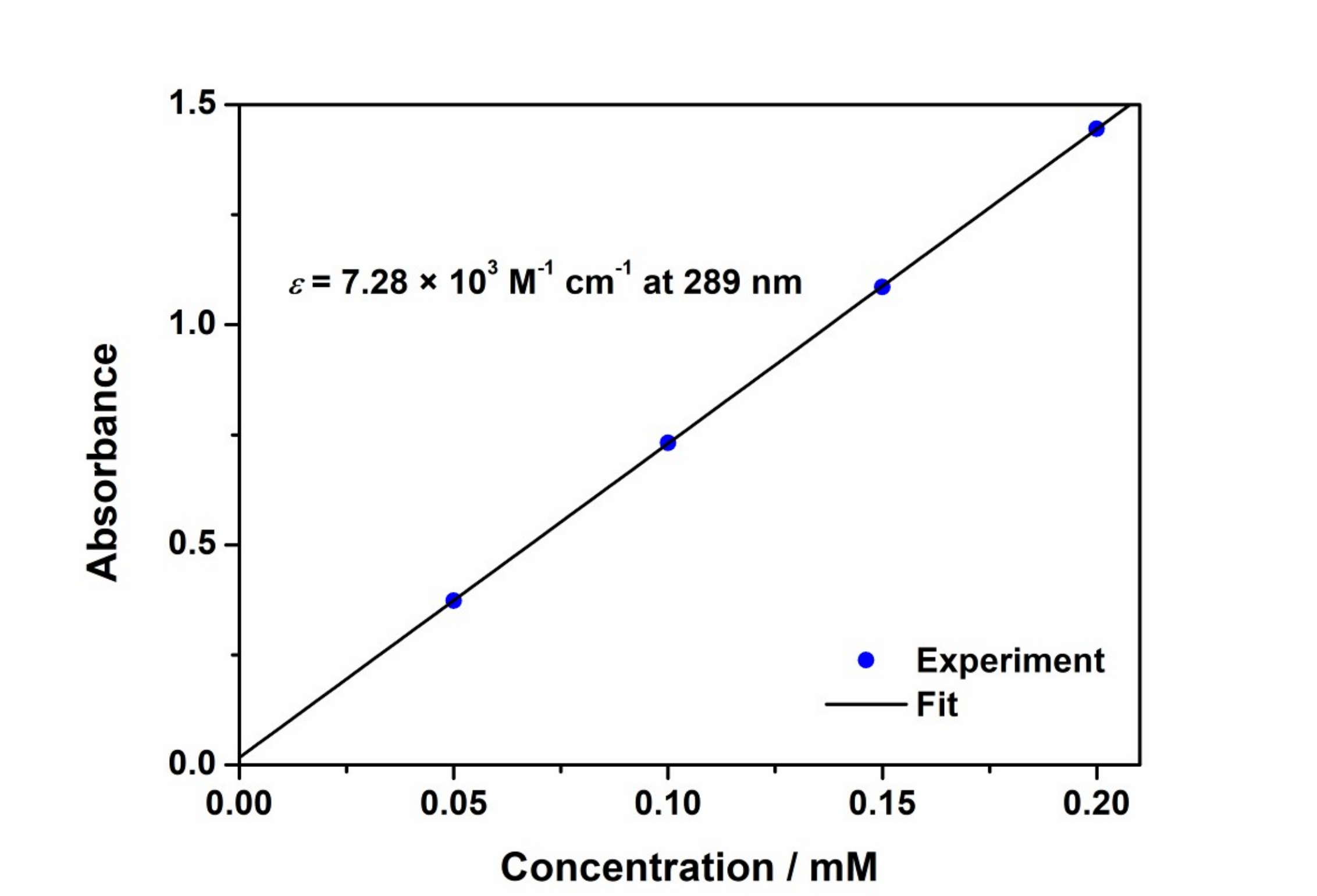

Figure S37. Plot of absorbance versus concentration for $FcBC_2(SiMe_3)_2C_2Me_2$ (**1**) at 289 nm in the UV-vis-NIR spectrum. The blue points are from the UV-vis-NIR spectrum (Figure S34). The solid black line is the best fit to the data to extract the extinction coefficient.

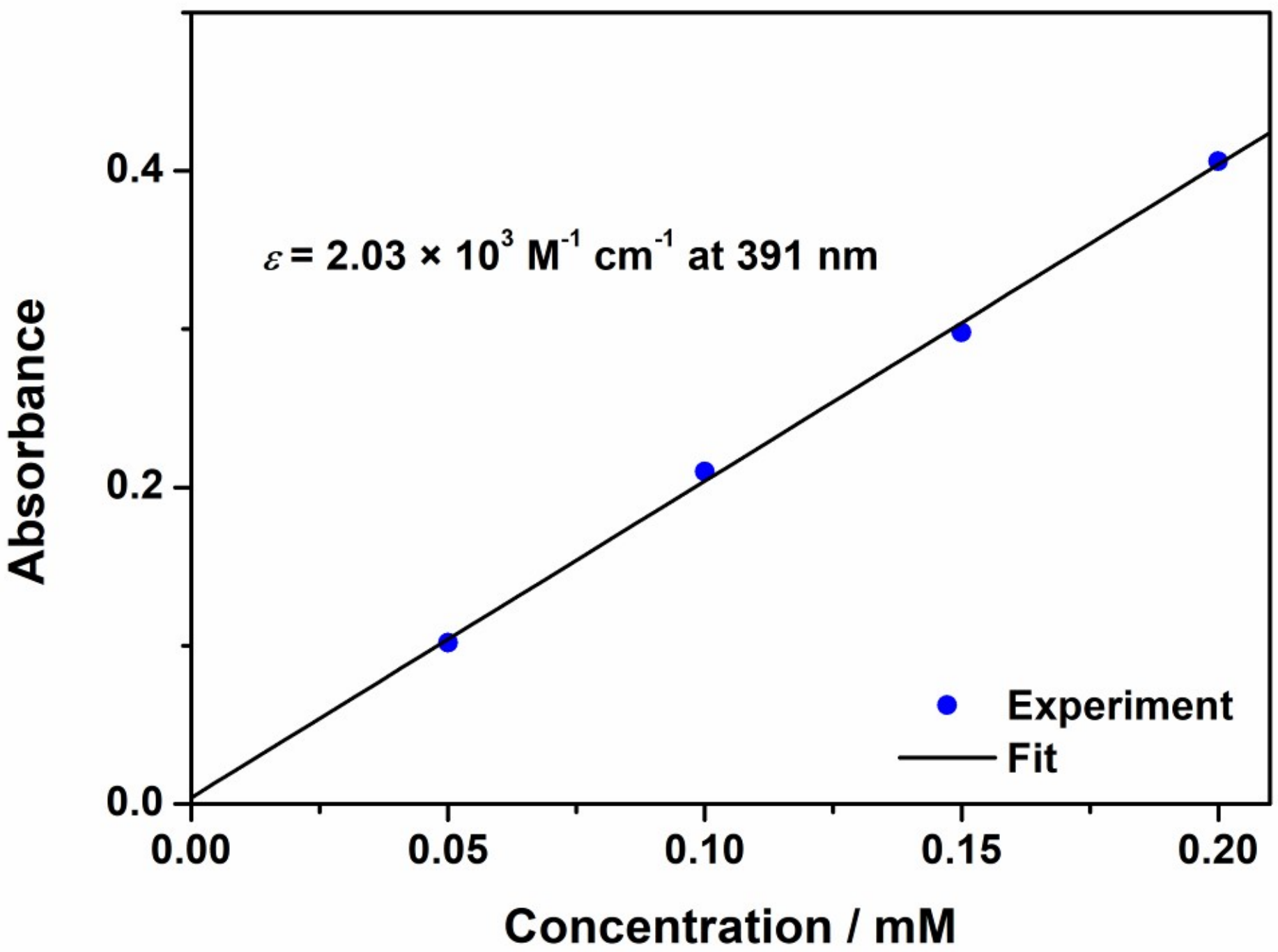

Figure S38. Plot of absorbance versus concentration for $FcBC_2(SiMe_3)_2C_2Me_2$ (**1**) at 391 nm in the UV-vis-NIR spectrum. The blue points are from the UV-vis-NIR spectrum (Figure S34). The solid black line is the best fit to the data to extract the extinction coefficient.

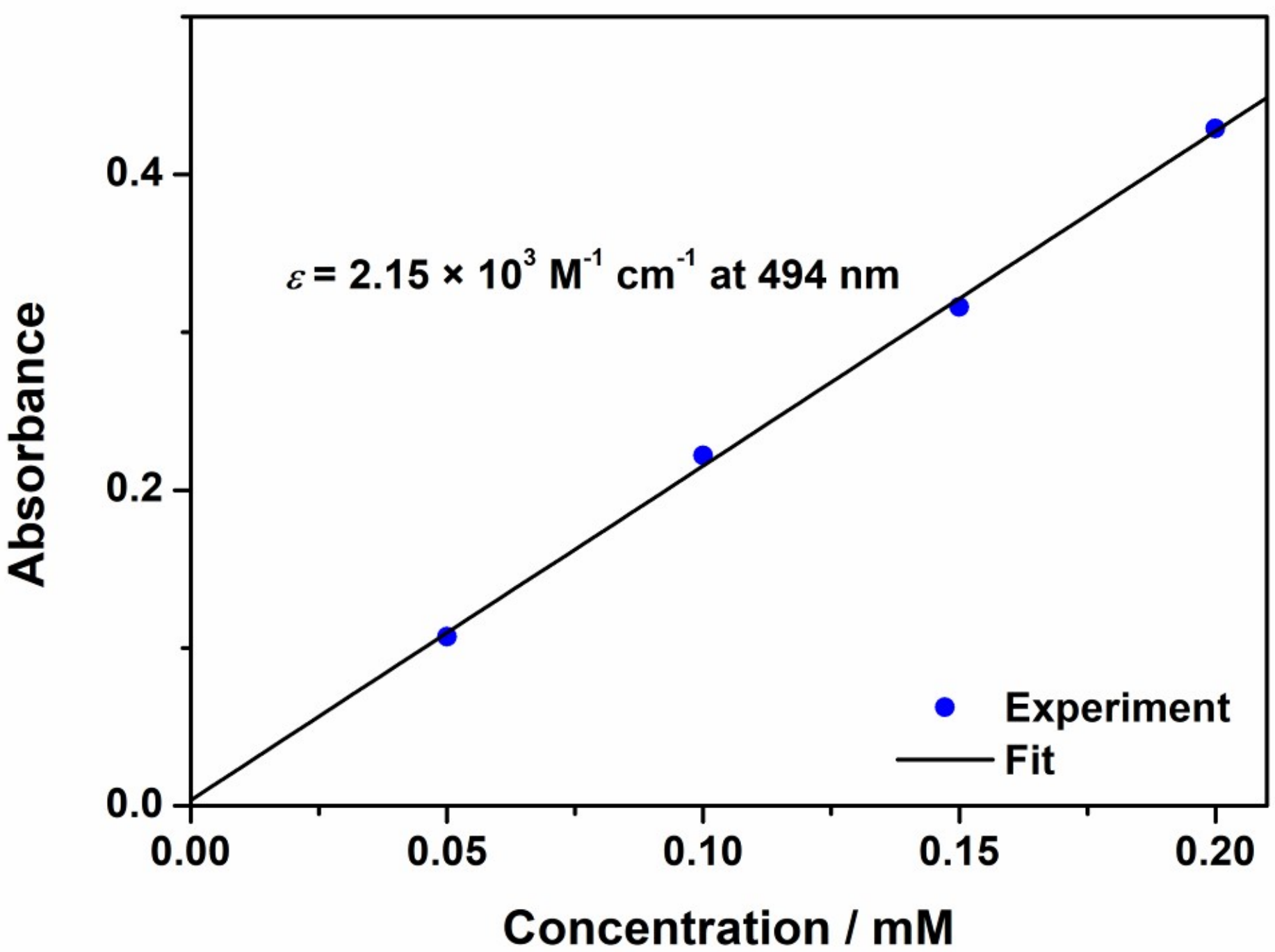

Figure S39. Plot of absorbance versus concentration for $FcBC_2(SiMe_3)_2C_2Me_2$ (**1**) at 494 nm in the UV-vis-NIR spectrum. The blue points are from the UV-vis-NIR spectrum (Figure S34). The solid black line is the best fit to the data to extract the extinction coefficient.

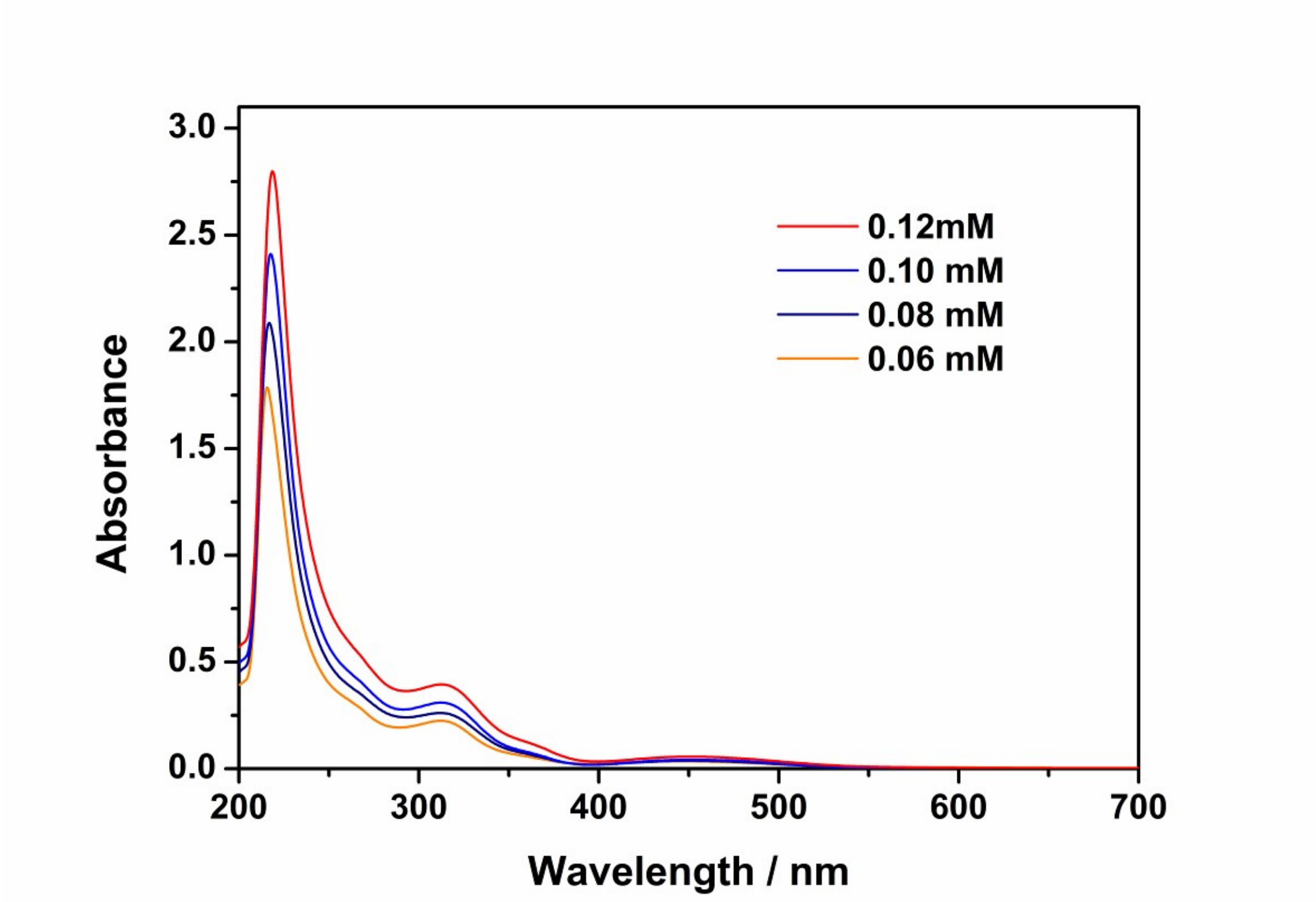

Figure S40. UV-vis-NIR spectrum of $[Mg(FcBC_2(SiMe_3)_2C_2Me_2)(THF)_3]\cdot THF$ (**2**) in THF at different concentrations. Significant absorptions occur at λ = 217, 314 and 450 nm.

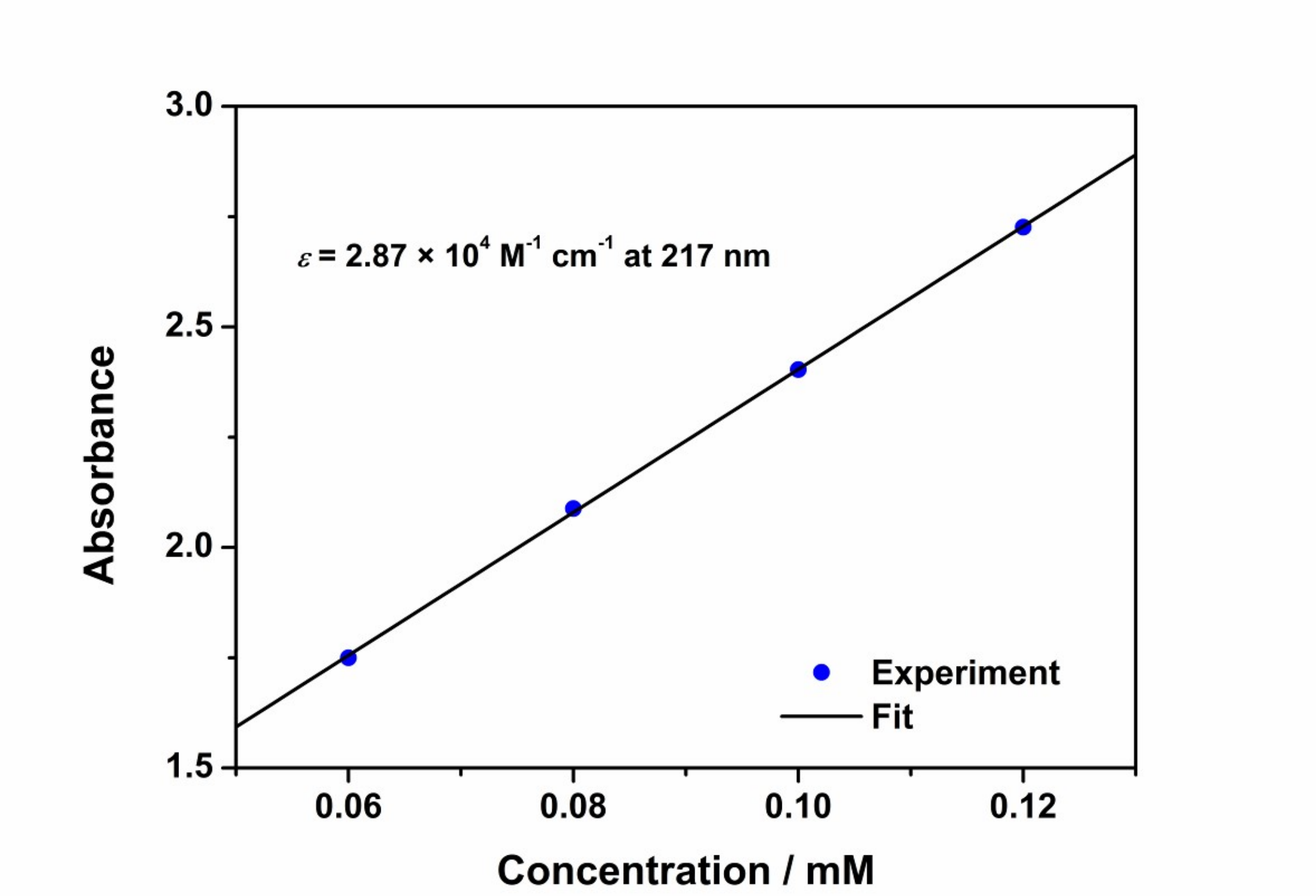

Figure S41. Plot of absorbance versus concentration for $[Mg(FcBC_2(SiMe_3)_2C_2Me_2)(THF)_3]\cdot THF$ (**2**) at 217 nm in the UV-vis-NIR spectrum. The blue points are from the UV-vis-NIR spectrum (Figure S40). The solid black line is the best fit to the data to extract the extinction coefficient.

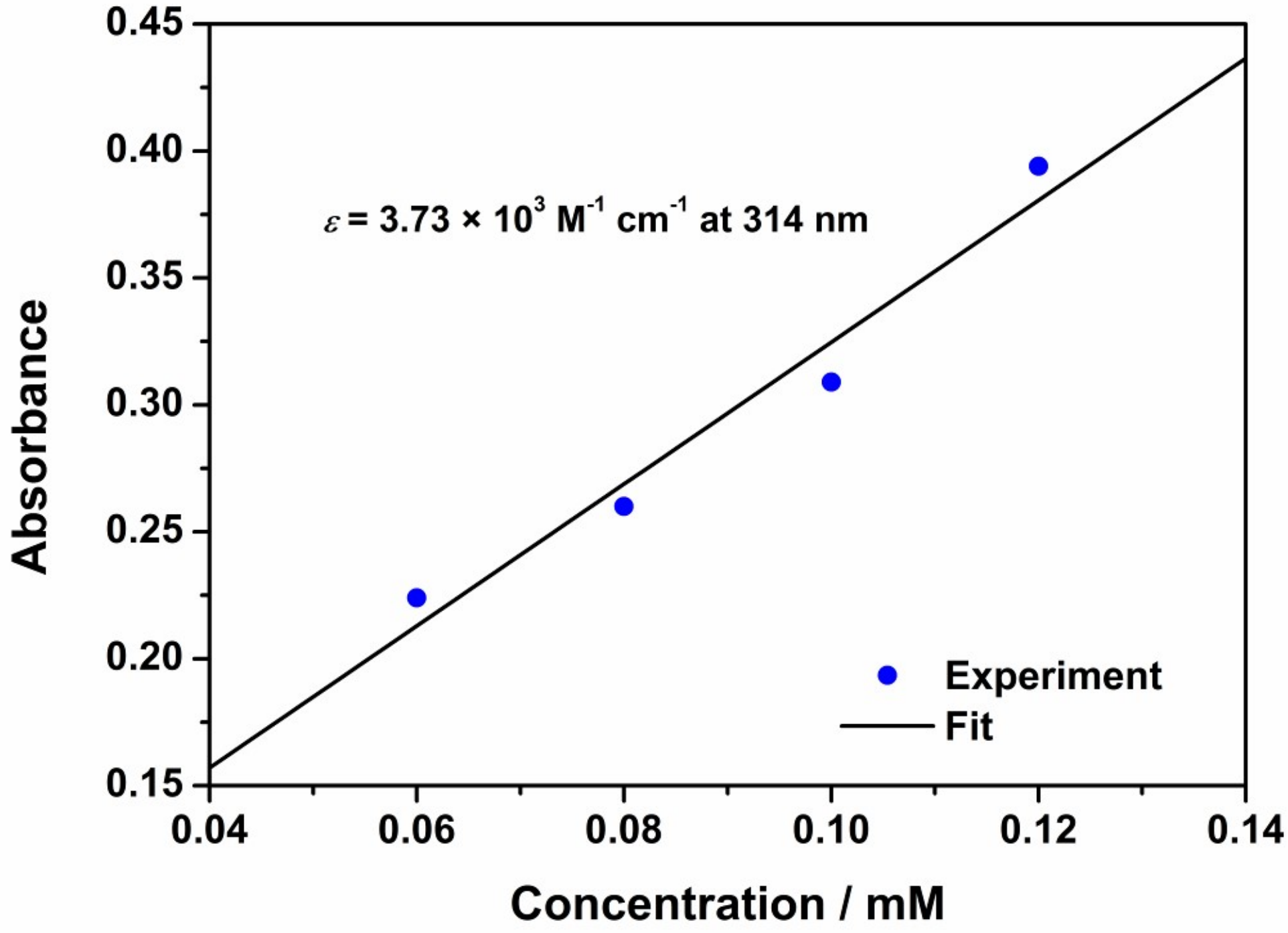

Figure S42. Plot of absorbance versus concentration for $[Mg(FcBC_2(SiMe_3)_2C_2Me_2)(THF)_3]\cdot THF$ (**2**) at 314 nm in the UV-vis-NIR spectrum. The blue points are from the UV-vis-NIR spectrum (Figure S40). The solid black line is the best fit to the data to extract the extinction coefficient.

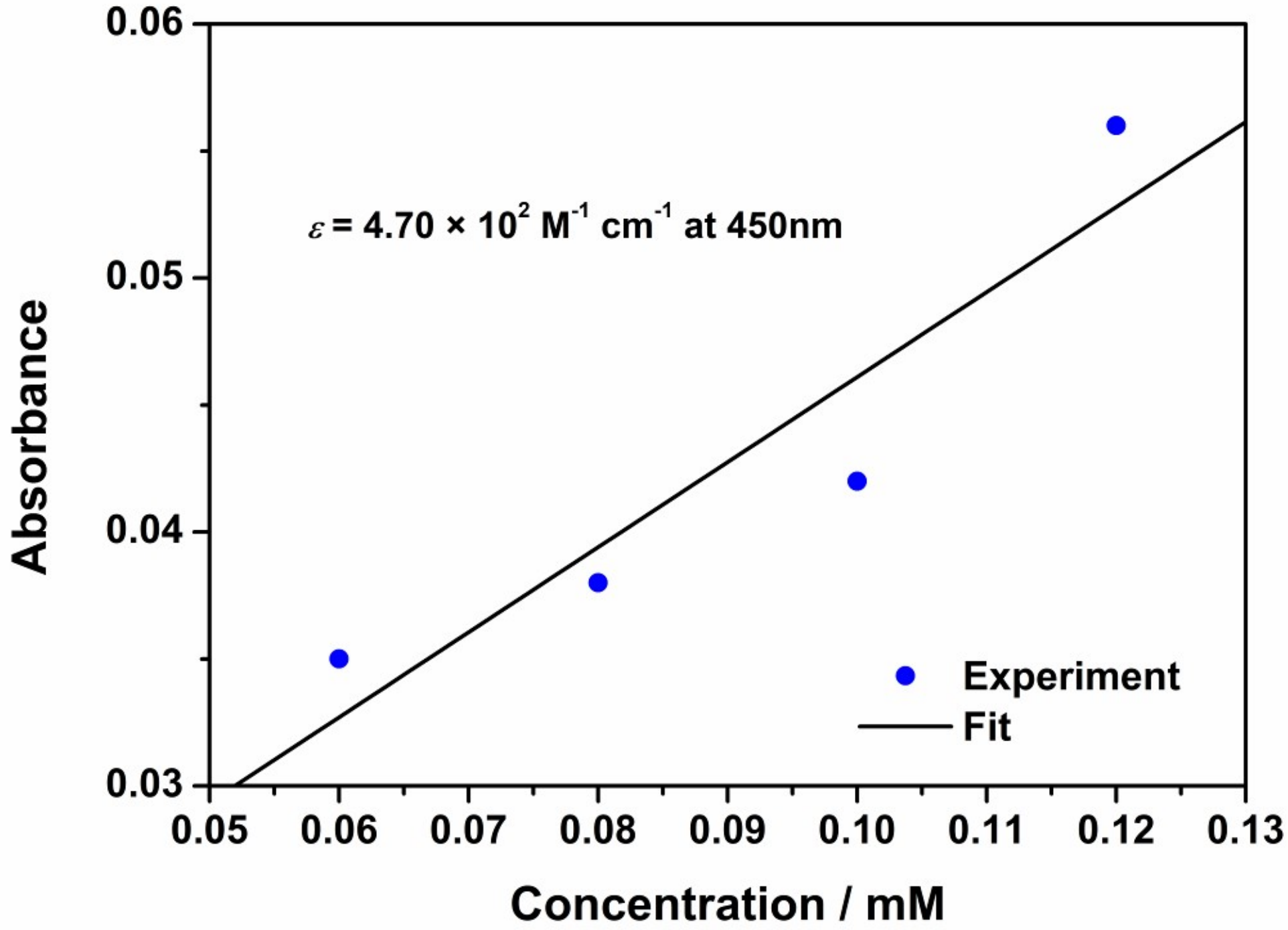

Figure S43. Plot of absorbance versus concentration for $[Mg(FcBC_2(SiMe_3)_2C_2Me_2)(THF)_3]\cdot THF$ (**2**) at 450 nm in the UV-vis-NIR spectrum. The blue points are from the UV-vis-NIR spectrum (Figure S40). The solid black line is the best fit to the data to extract the extinction coefficient.

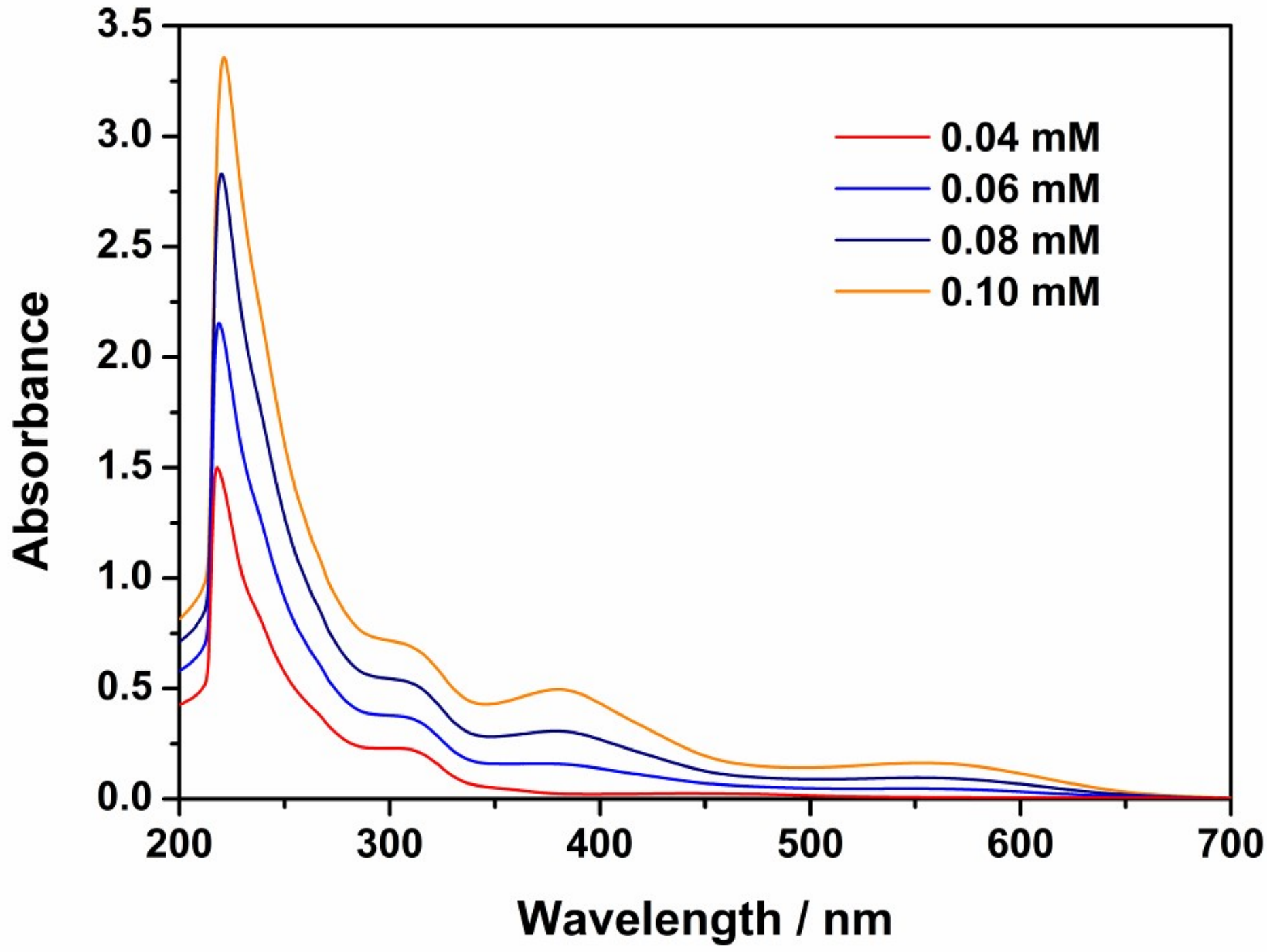

Figure S44. UV-vis-NIR spectrum of $[(C_5{}^iPr_5)La(FcBC_2(SiMe_3)_2C_2Me_2)]\cdot$2toluene (**3-La**) in hexane at different concentrations. Significant absorptions occur at λ = 220, 314, 380, 552 nm.

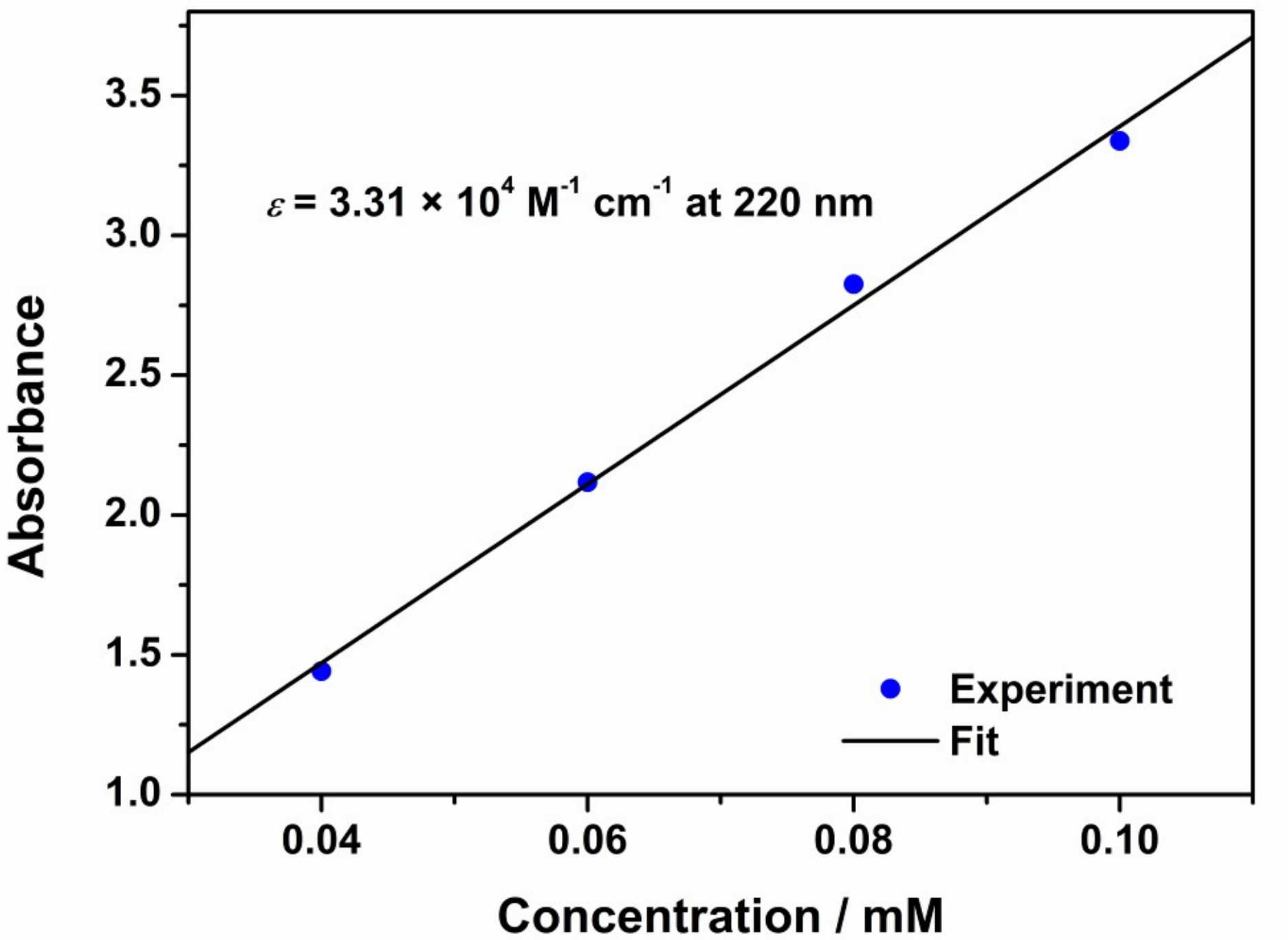

Figure S45. Plot of absorbance versus concentration for $[(C_5{}^iPr_5)La(FcBC_2(SiMe_3)_2C_2Me_2)]\cdot$2toluene (**3-La**) at 220 nm in the UV-vis-NIR spectrum. The blue points are from the UV-vis-NIR spectrum (Figure S44). The solid black line is the best fit to the data to extract the extinction coefficient.

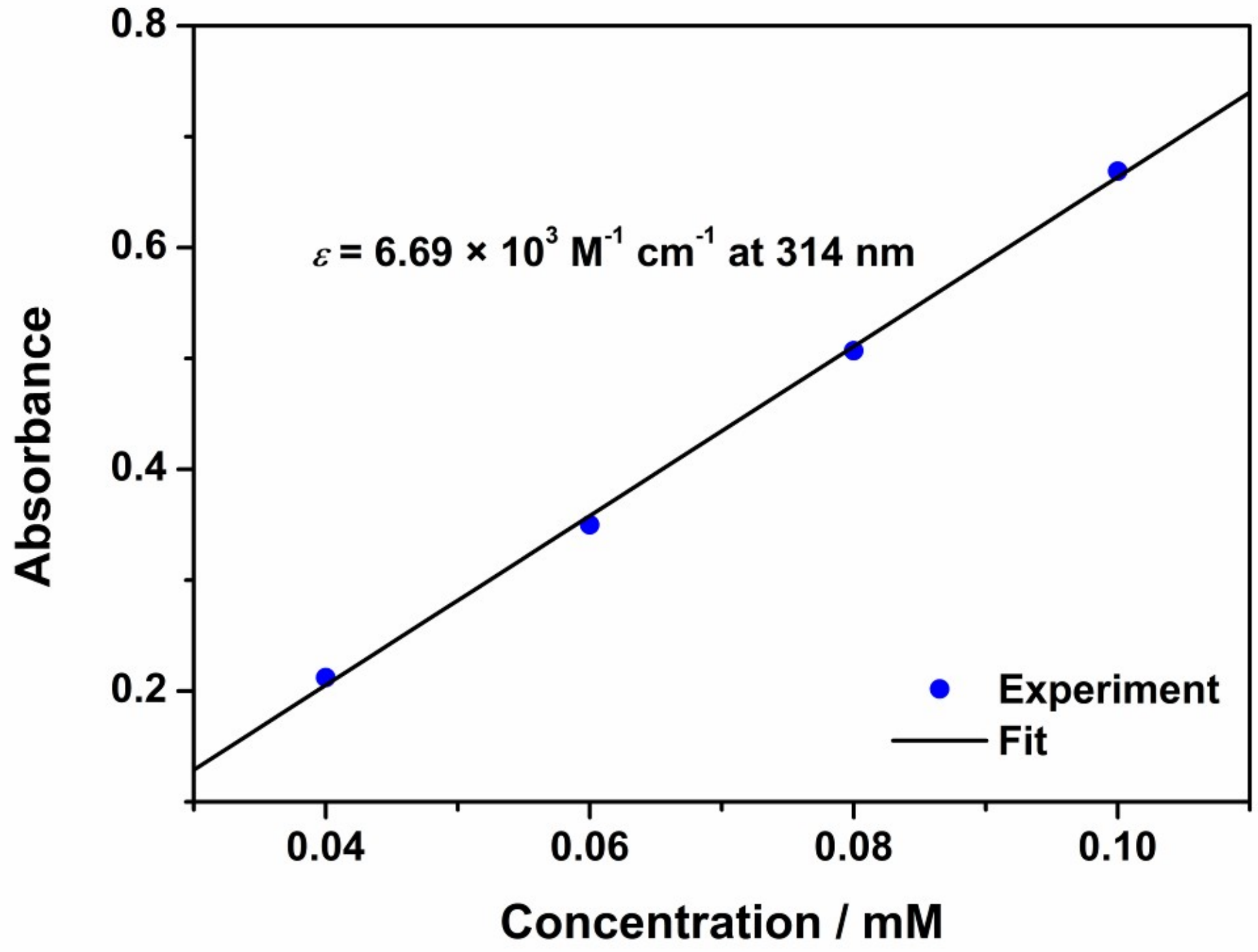

Figure S46. Plot of absorbance versus concentration for $[(C_5{}^{i}Pr_5)La(FcBC_2(SiMe_3)_2C_2Me_2)]{\cdot}2toluene$ (**3-La**) at 314 nm in the UV-vis-NIR spectrum. The blue points are from the UV-vis-NIR spectrum (Figure S44). The solid black line is the best fit to the data to extract the extinction coefficient.

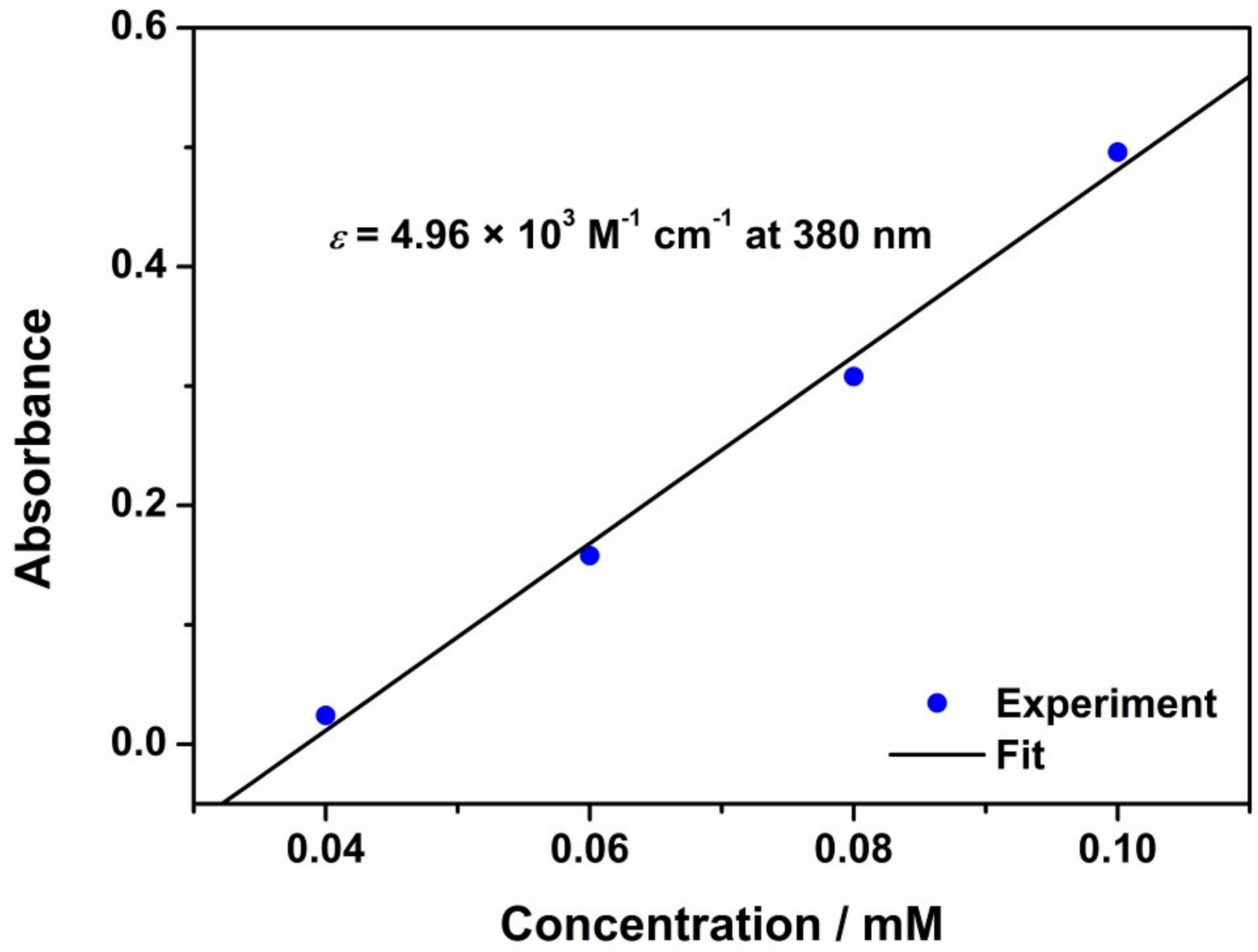

Figure S47. Plot of absorbance versus concentration for $[(C_5{}^{i}Pr_5)La(FcBC_2(SiMe_3)_2C_2Me_2)]{\cdot}2toluene$ at 380 nm in the UV-vis-NIR spectrum. The blue points are from the UV-vis-NIR spectrum (Figure S44). The solid black line is the best fit to the data to extract the extinction coefficient.

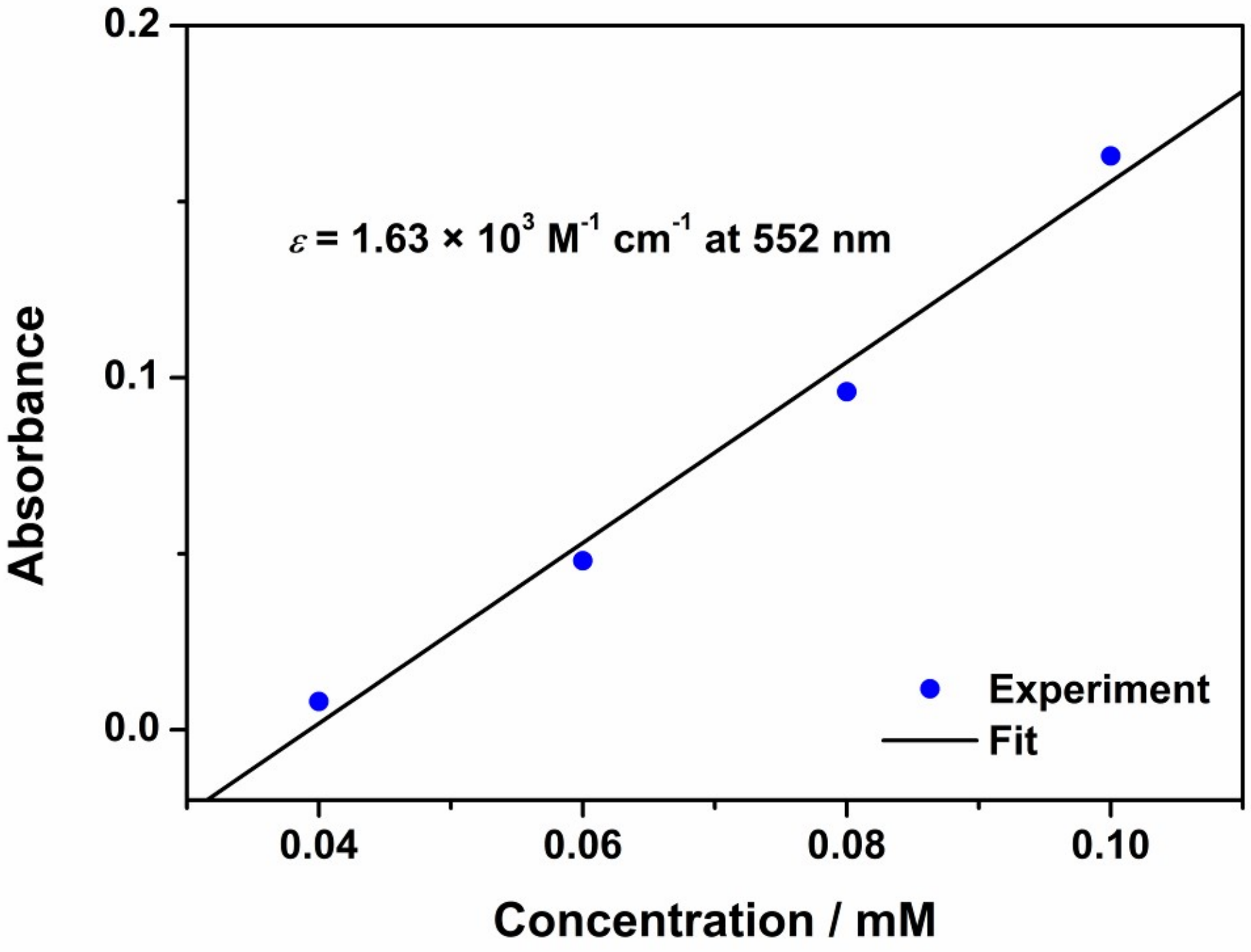

Figure S48. Plot of absorbance versus concentration for $[(C_5{}^iPr_5)La(FcBC_2(SiMe_3)_2C_2Me_2)]\cdot 2toluene$ (**3-La**) at 552 nm in the UV-vis-NIR spectrum. The blue points are from the UV-vis-NIR spectrum (Figure S44). The solid black line is the best fit to the data to extract the extinction coefficient.

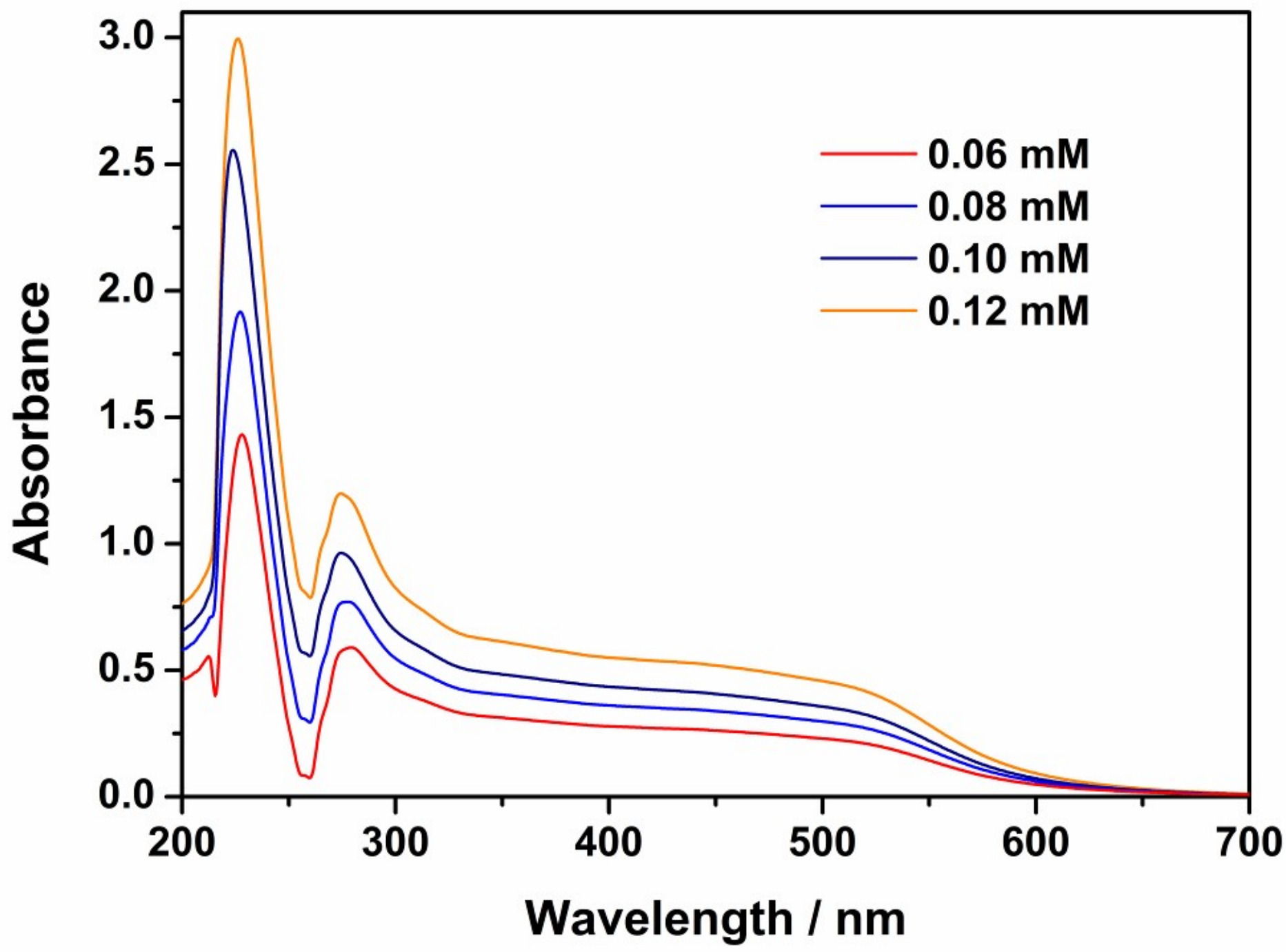

Figure S49. UV-vis-NIR spectrum of [($C_5{}^iPr_5$)Dy($FcBC_2(SiMe_3)_2C_2Me_2$)]·2toluene (**3-Dy**) in hexane at different concentrations. Significant absorptions occur at λ = 226, 275 nm.

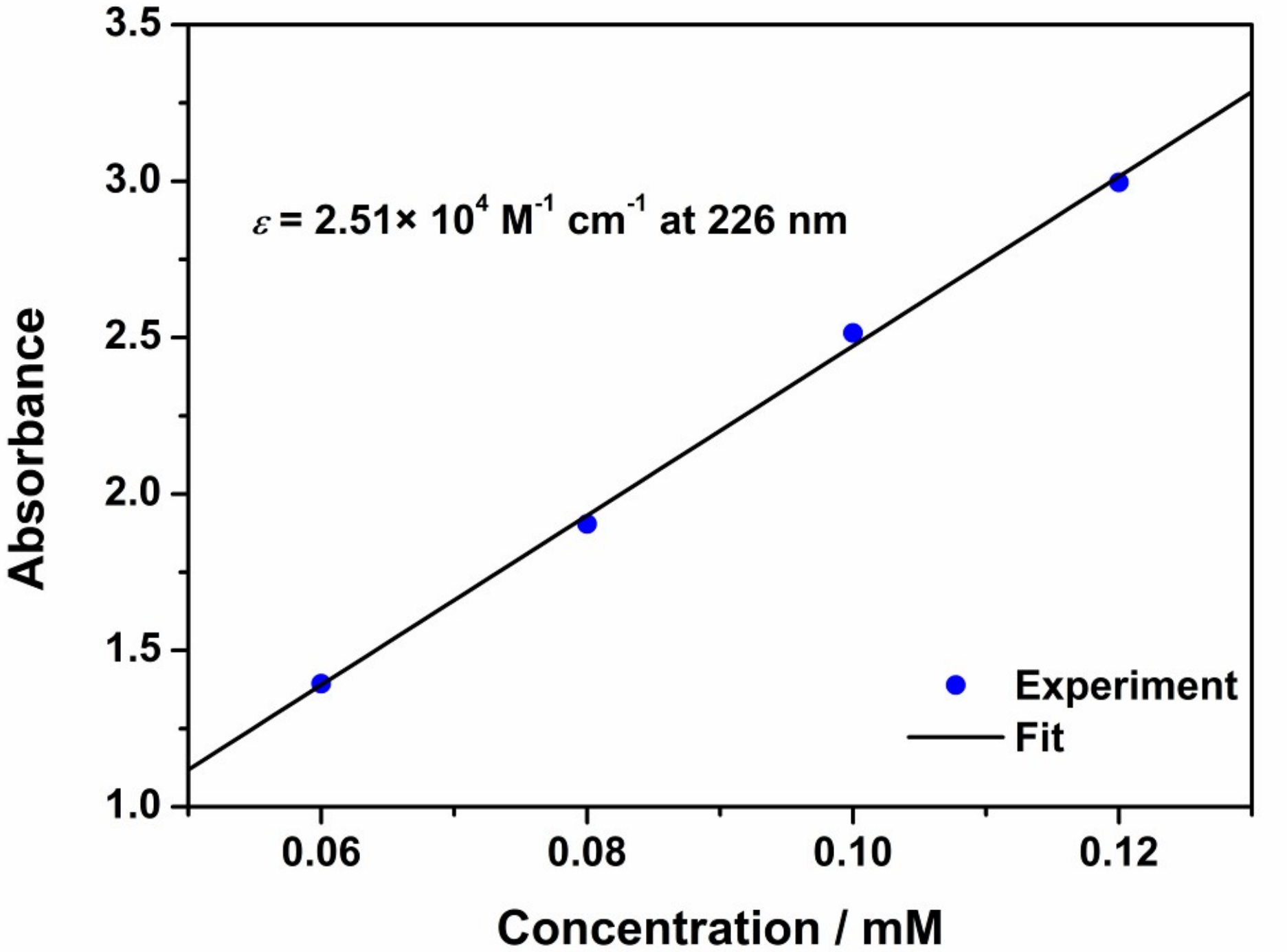

Figure S50. Plot of absorbance versus concentration for [($C_5{}^iPr_5$)Dy($FcBC_2(SiMe_3)_2C_2Me_2$)]·2toluene (**3-Dy**) at 226 nm in the UV-vis-NIR spectrum. The blue points are from the UV-vis-NIR spectrum (Figure S54). The solid black line is the best fit to the data to extract the extinction coefficient.

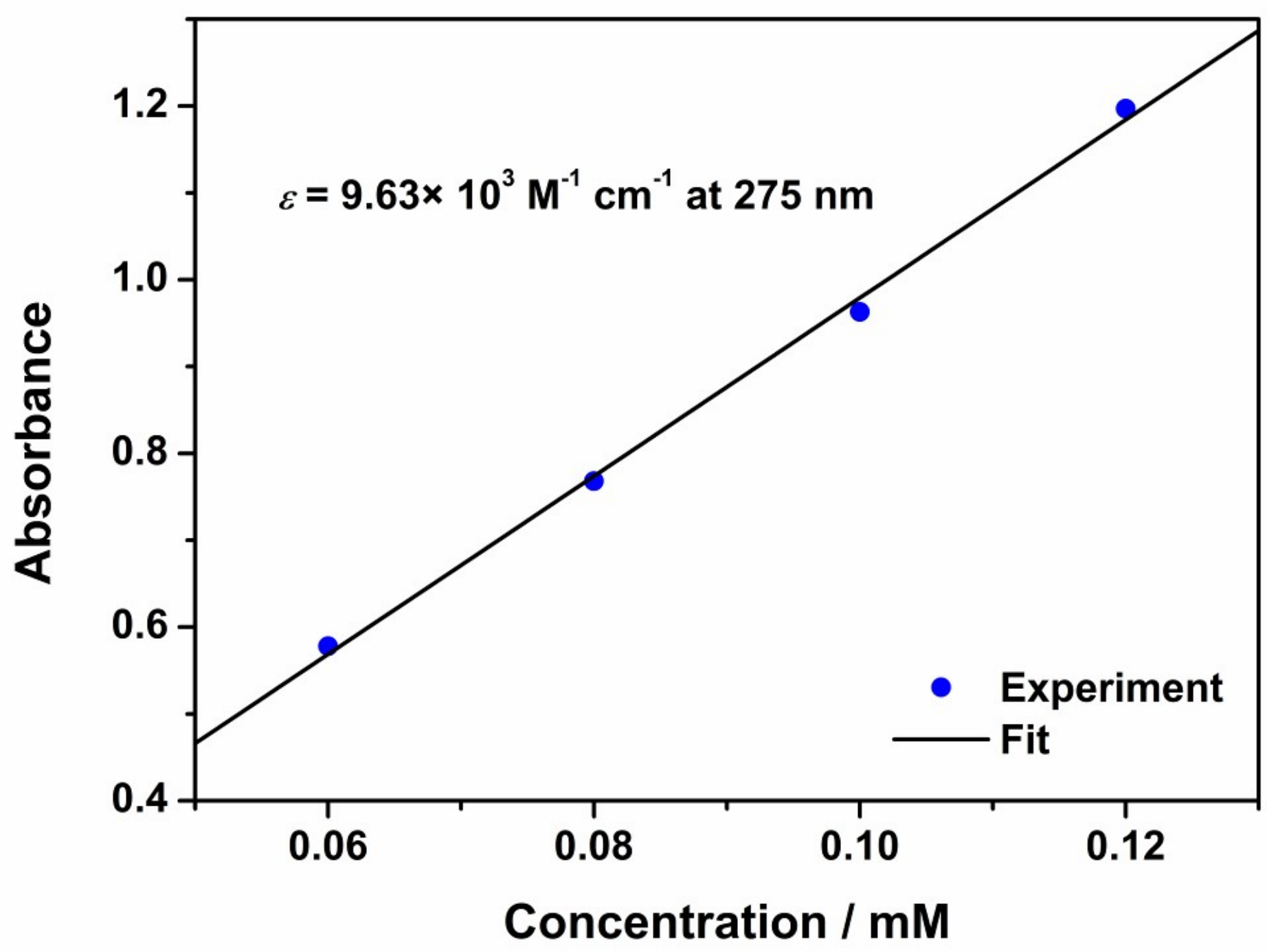

Figure S51. Plot of absorbance versus concentration for $[(C_5{}^iPr_5)Dy(FcBC_2(SiMe_3)_2C_2Me_2)]\cdot 2$toluene (**3-Dy**) at 275 nm in the UV-vis-NIR spectrum. The blue points are from the UV-vis-NIR spectrum (Figure S54). The solid black line is the best fit to the data to extract the extinction coefficient.

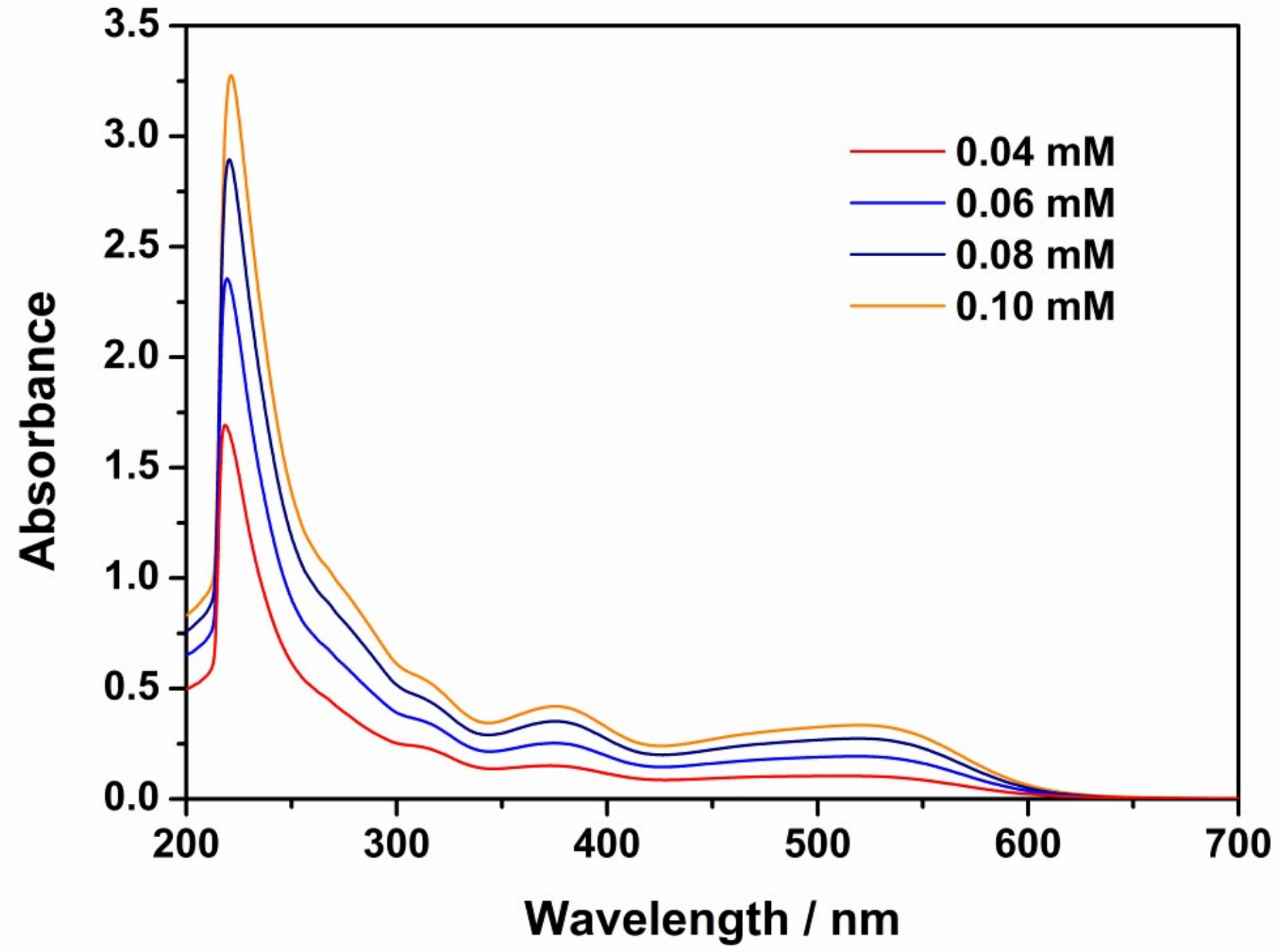

Figure S52. UV-vis-NIR spectrum of [($C_5{}^iPr_5$)Y($FcBC_2(SiMe_3)_2C_2Me_2$)]·2toluene (**3-Y**) in hexane at different concentrations. Significant absorptions occur at λ = 220, 314, 376, 521 nm.

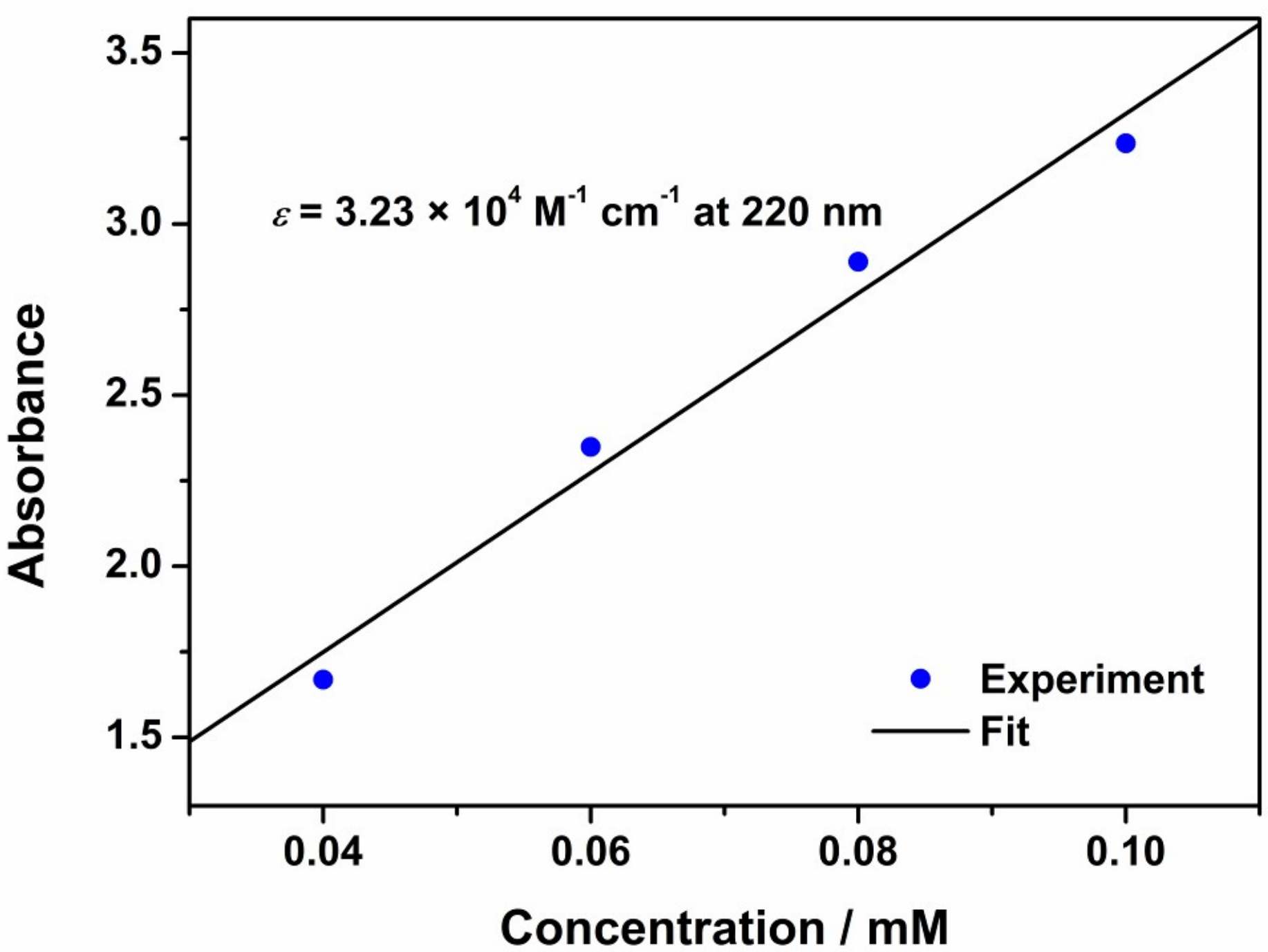

Figur Figure S53. Plot of absorbance versus concentration for [($C_5{}^iPr_5$)Y($FcBC_2(SiMe_3)_2C_2Me_2$)]·2toluene (**3-Y**) at 220 nm in the UV-vis-NIR spectrum. The blue points are from the UV-vis-NIR spectrum (Figure S49). The solid black line is the best fit to the data to extract the extinction coefficient.

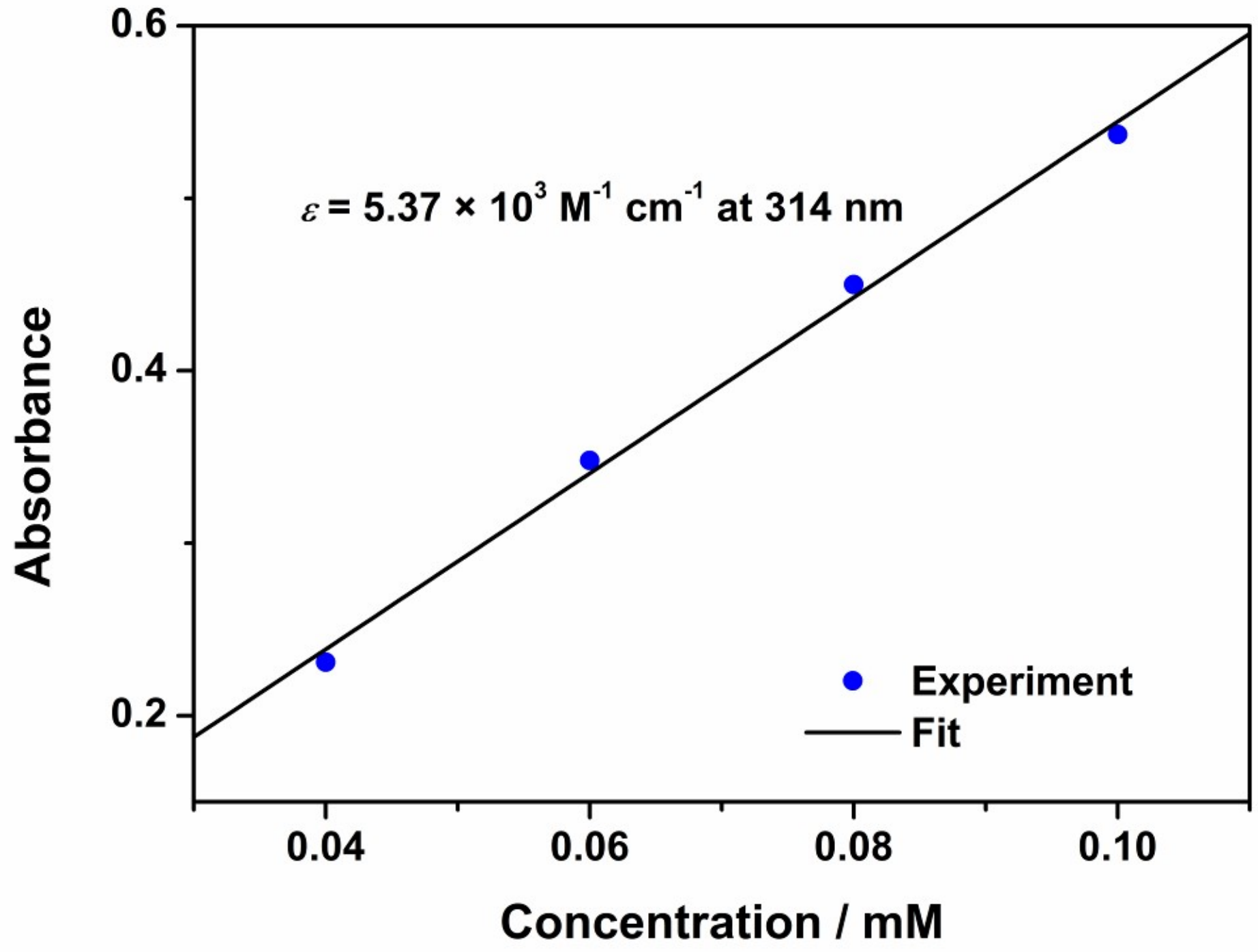

Figure S54. Plot of absorbance versus concentration for [($C_5{}^iPr_5$)Y($FcBC_2(SiMe_3)_2C_2Me_2$)]·2toluene (**3-Y**) at 314 nm in the UV-vis-NIR spectrum. The blue points are from the UV-vis-NIR spectrum (Figure S49). The solid black line is the best fit to the data to extract the extinction coefficient.

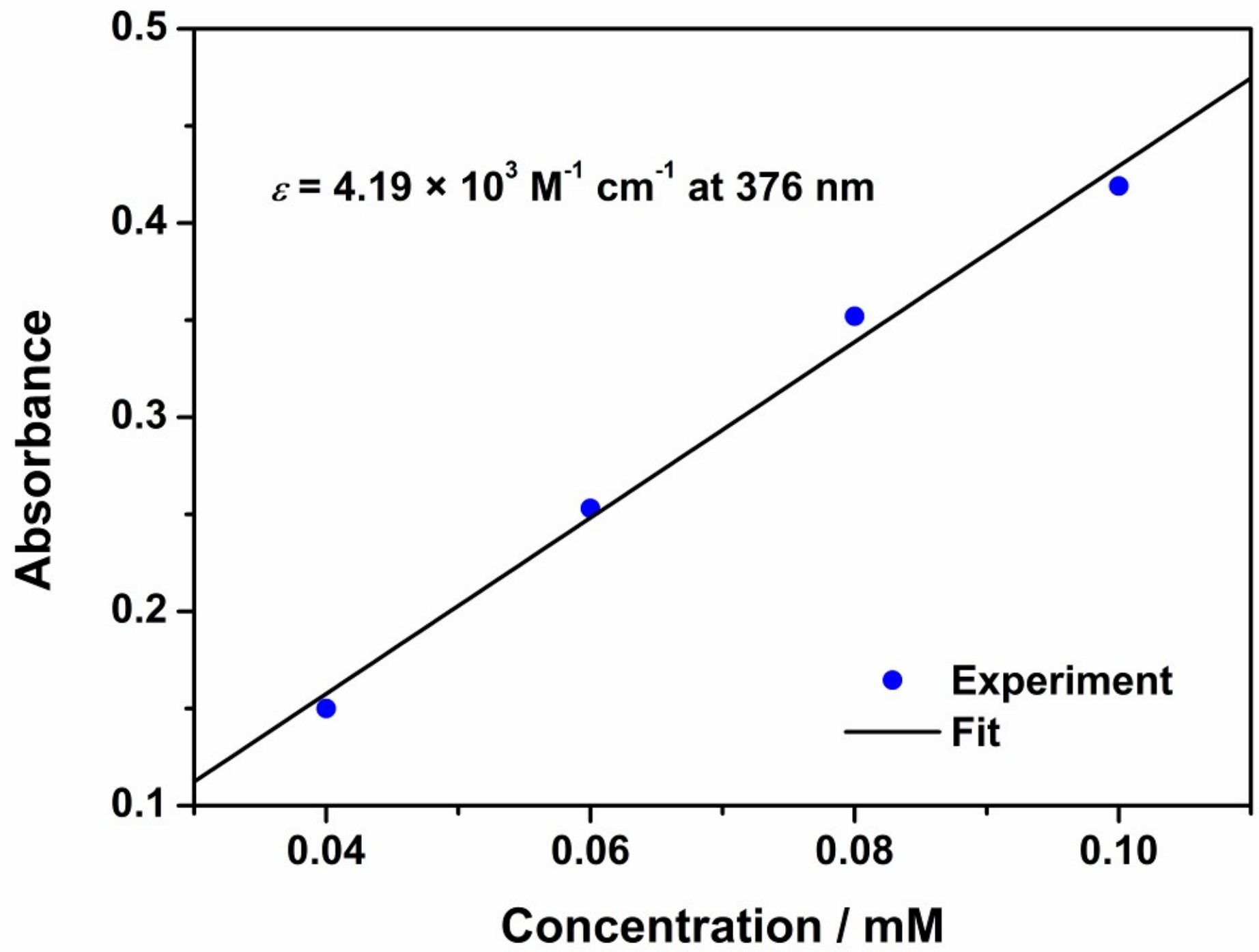

Figure S55. Plot of absorbance versus concentration for [($C_5{}^iPr_5$)Y($FcBC_2(SiMe_3)_2C_2Me_2$)]·2toluene (**3-Y**) at 376 nm in the UV-vis-NIR spectrum. The blue points are from the UV-vis-NIR spectrum (Figure S49). The solid black line is the best fit to the data to extract the extinction coefficient.

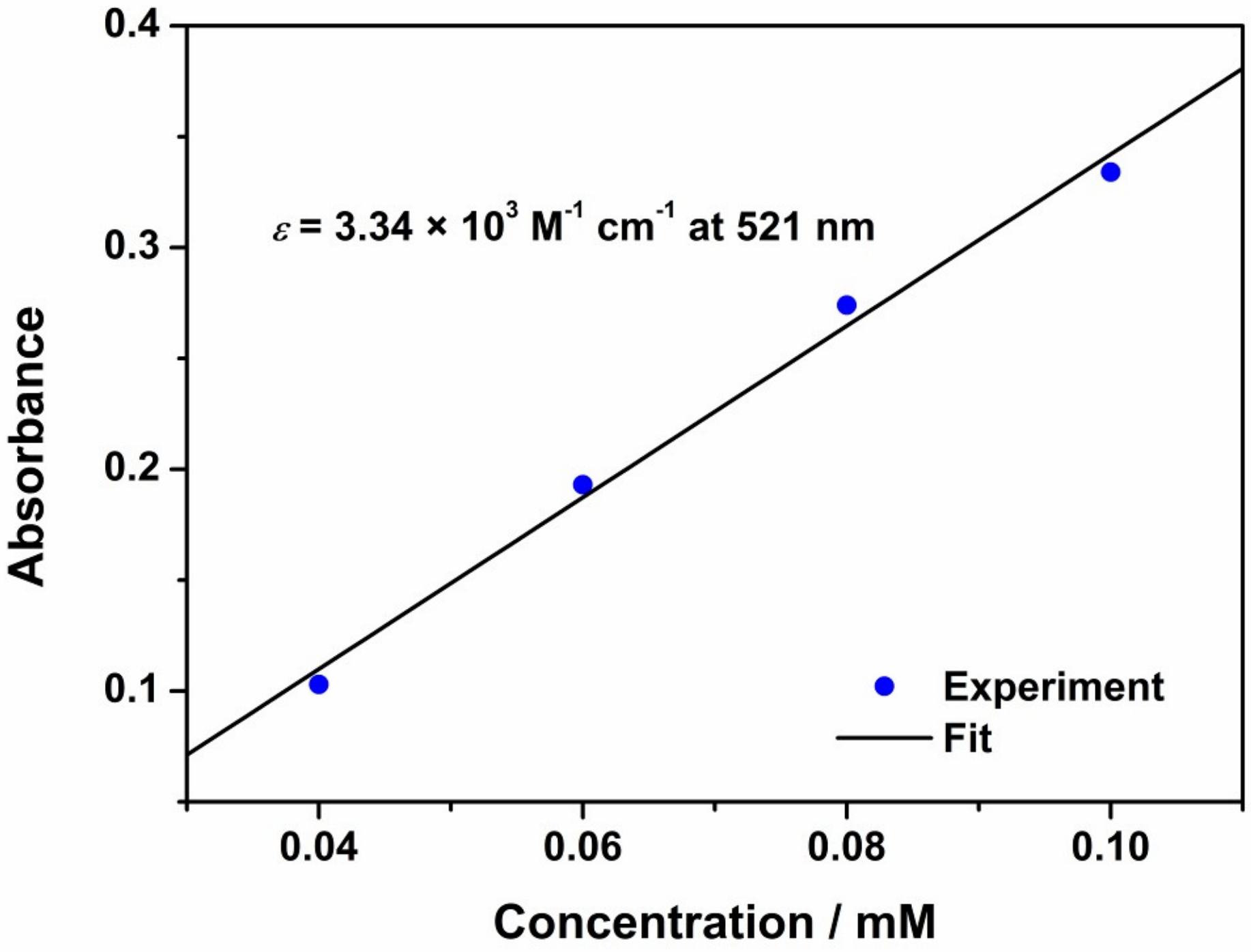

Figure S56. Plot of absorbance versus concentration for $[(C_5{}^iPr_5)Y(FcBC_2(SiMe_3)_2C_2Me_2)]{\cdot}2$toluene (**3-Y**) at 521 nm in the UV-vis-NIR spectrum. The blue points are from the UV-vis-NIR spectrum (Figure S49). The solid black line is the best fit to the data to extract the extinction coefficient.

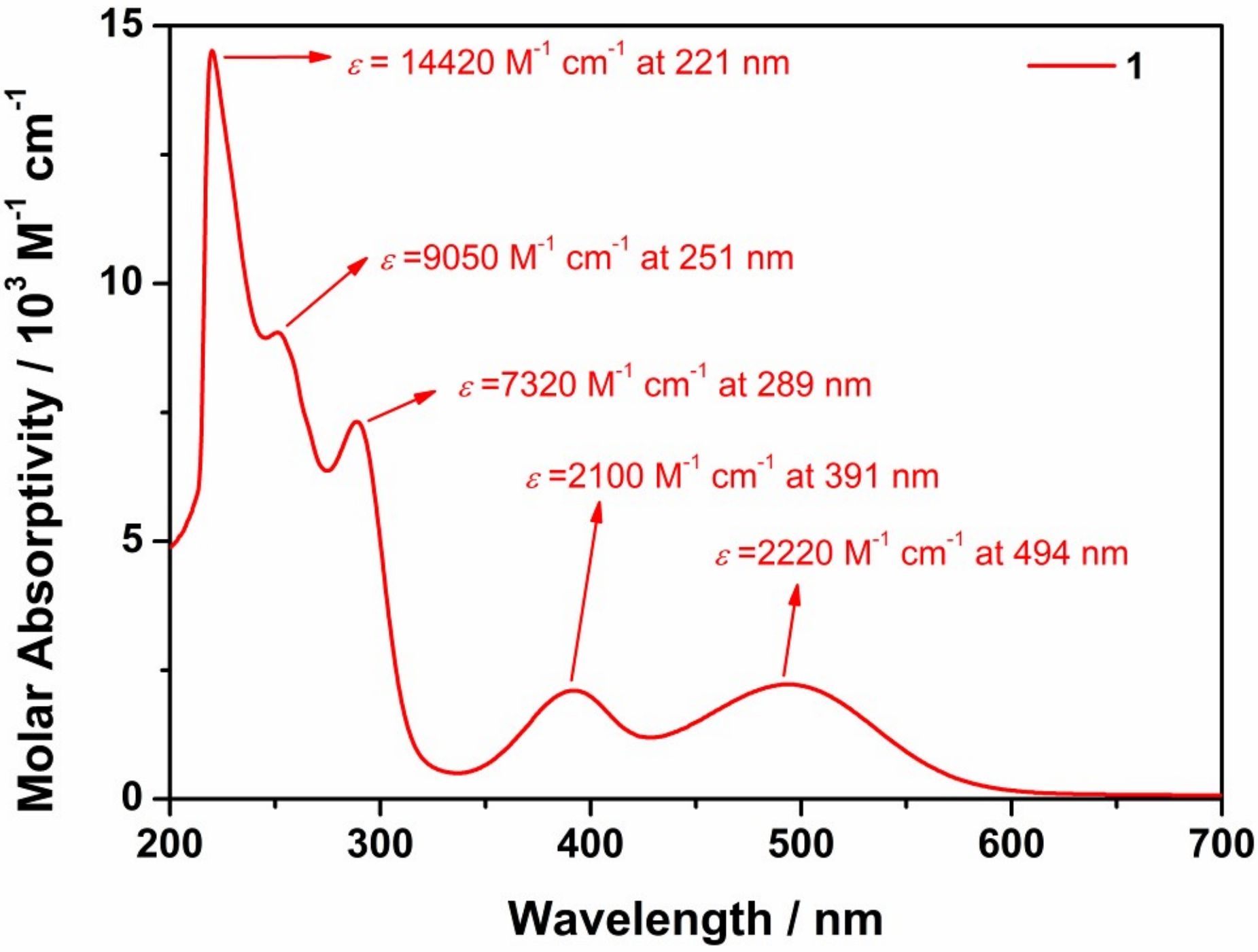

Figure S57. UV−vis-NIR spectrum of a 0.1 mM solution of **1** in hexane.

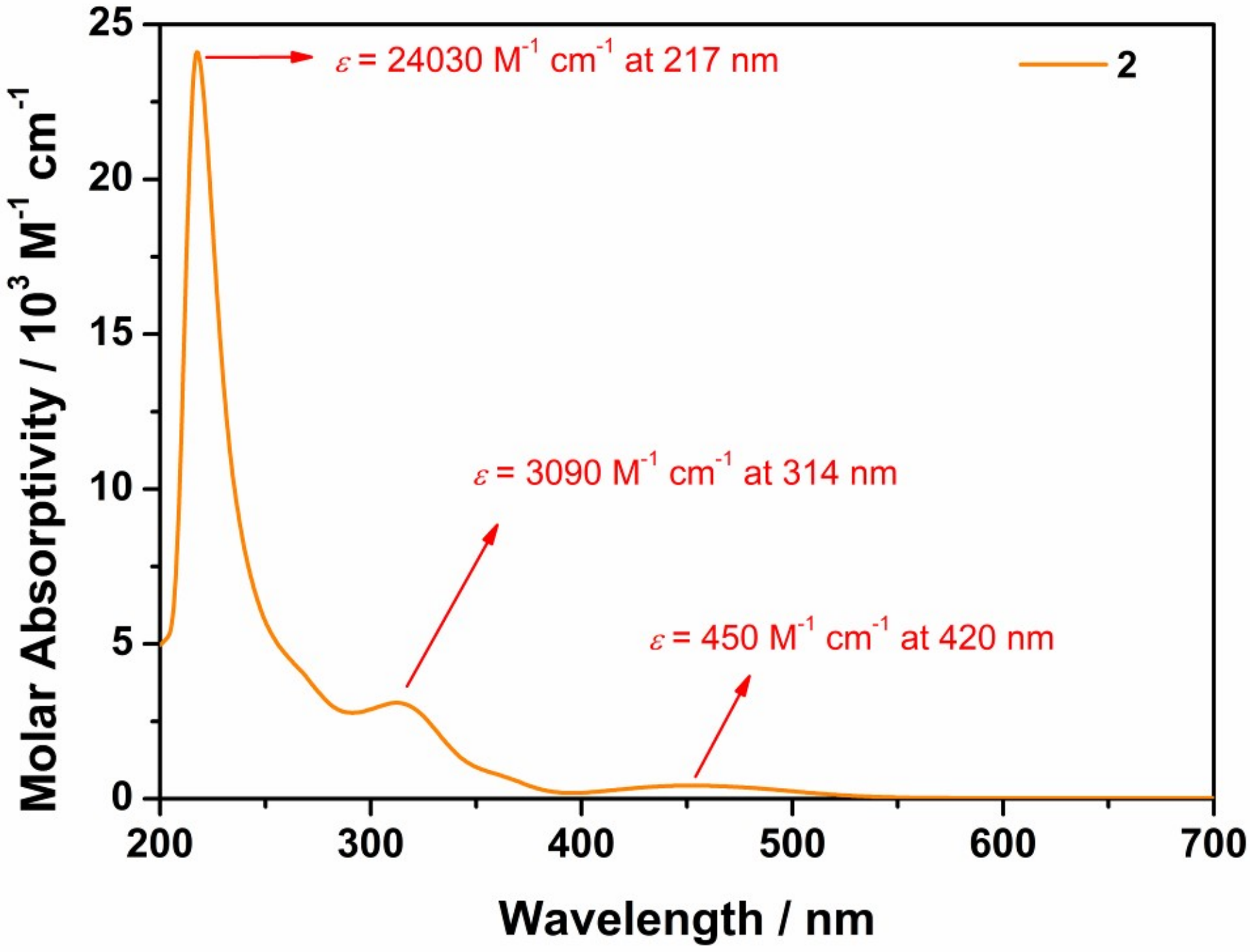

Figure S58. UV−vis-NIR spectrum of a 0.1 mM solution of **2** in THF.

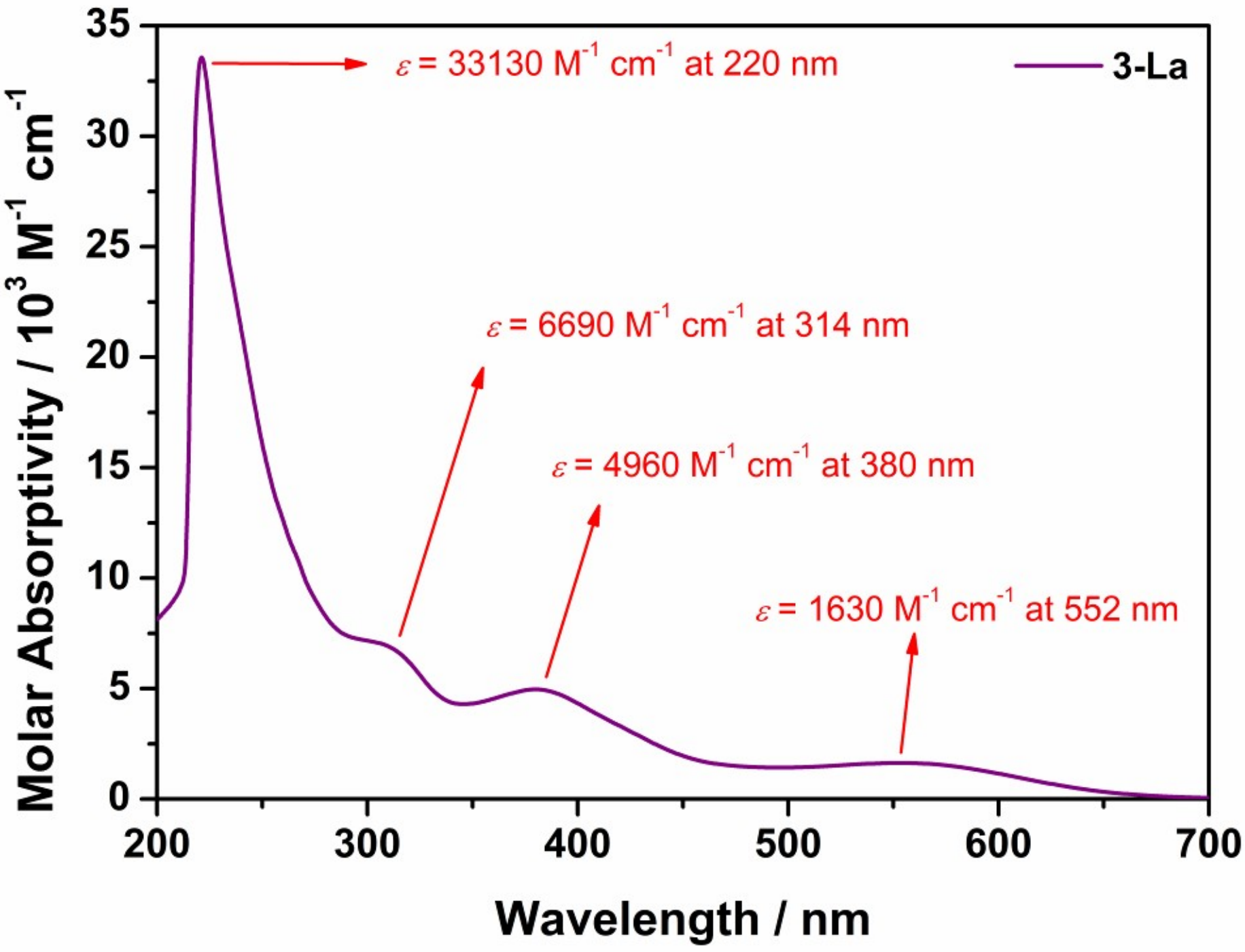

Figure S59. UV−vis-NIR spectrum of a 0.1 mM solution of **3-La** in hexane.

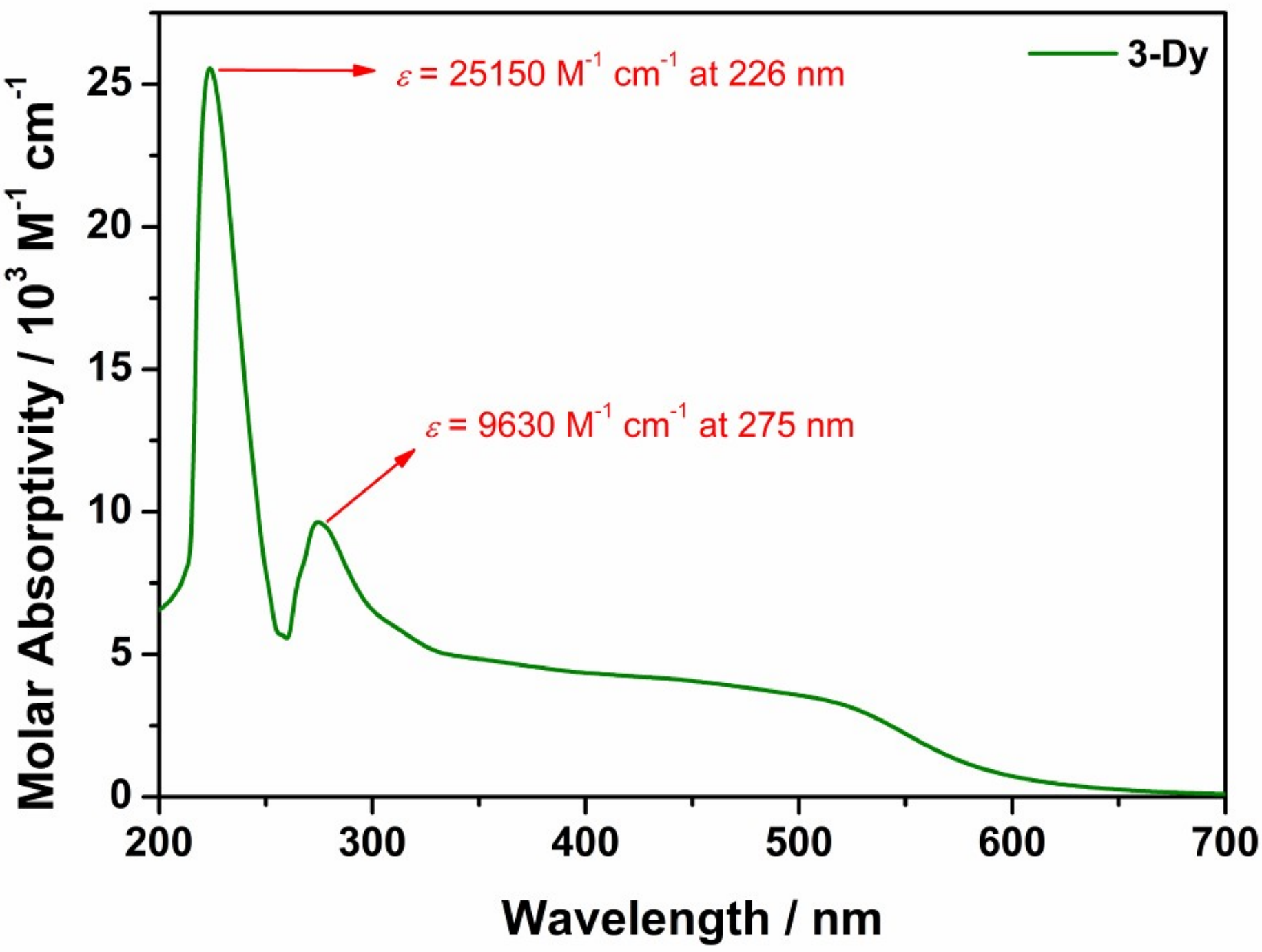

Figure S60. UV−vis-NIR spectrum of a 0.1 mM solution of **3-Dy** in hexane.

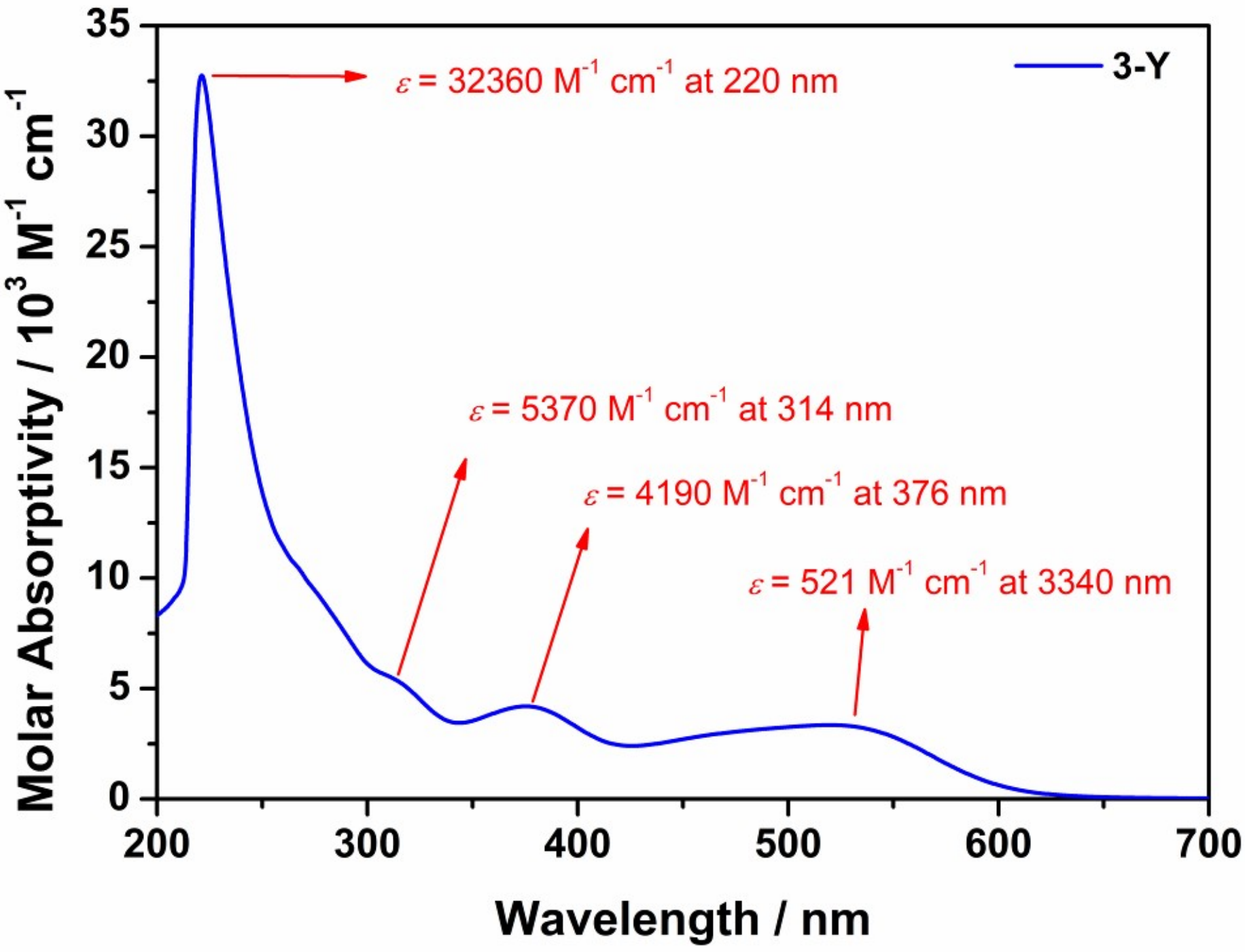

Figure S61. UV−vis-NIR spectrum of a 0.1 mM solution of **3-Y** in hexane.

Magnetic property measurements

the charge neutral compound **3-Dy** could be dissolved in melted eicosane. In order to characterize the magnetic behaviour in its crystalline state, The polycrystalline sample of **3-Dy** was immersed in Fomblin® Y (LVAC 25/6) and sealed in an NMR tube. Prior to centring and measurement, the sample was first cooled in zero field below the pour point of the Fomblin oil to prevent crystal orientation. Direct current (DC) magnetic susceptibility and magnetization data (VSM mode) were then collected using a Quantum Design MPMS3 magnetometer in warming mode. Alternating current (AC) magnetic susceptibility measurements were performed using a Quantum Design MPMS3 magnetometer using an oscillating field of 2 Oe. Diamagnetic corrections were performed using Pascal's constants.[8]

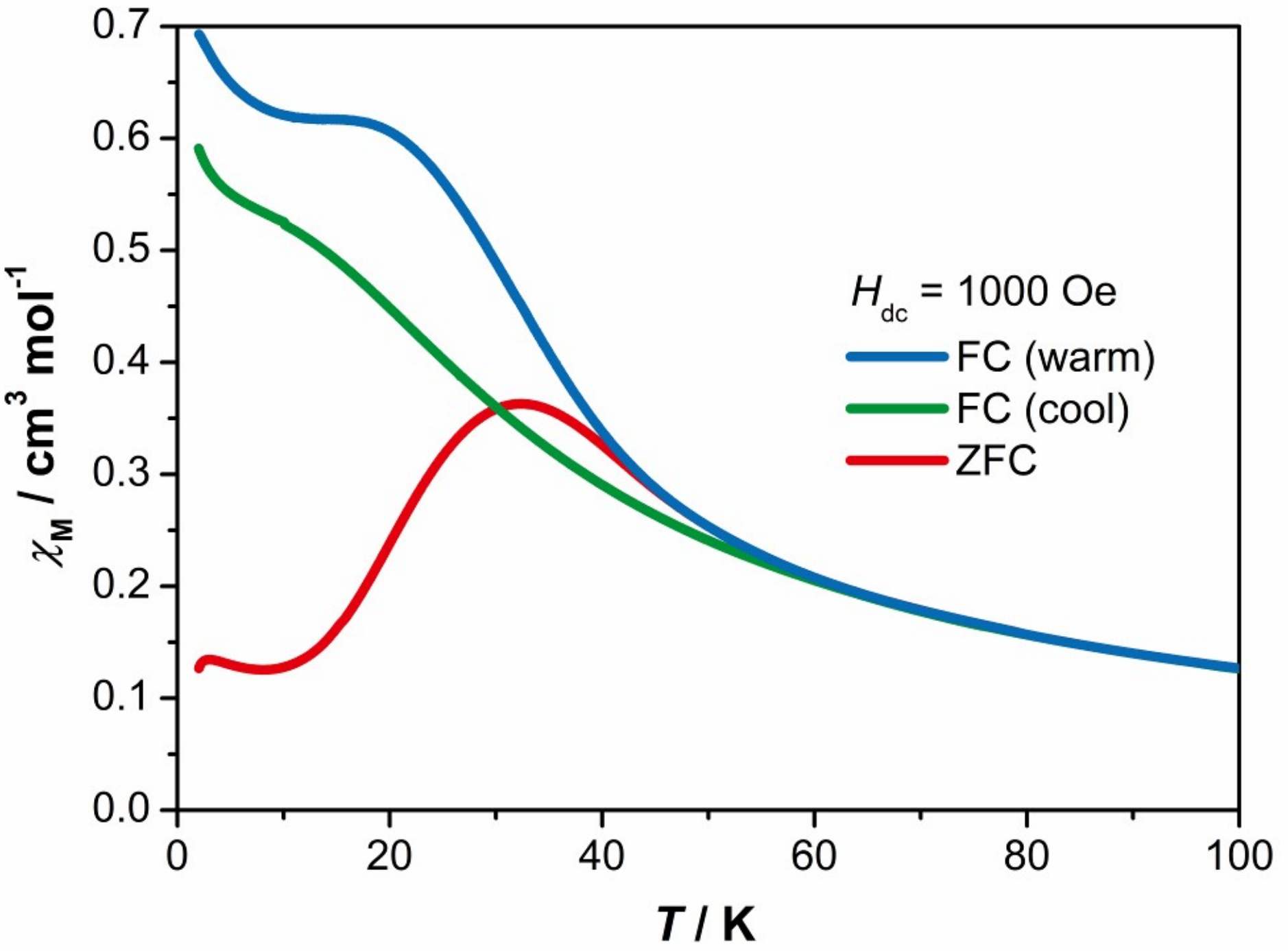

Figure S62. Field-cooled (FC, blue line) and zero-field-cooled (ZFC, red line) variable-temperature magnetic susceptibility for **3-Dy** under 1000 Oe DC filed in warm mode from 2 to 100 K. The green line represents FC variable-temperature magnetic susceptibility in cool mode from 100 to 2 K.

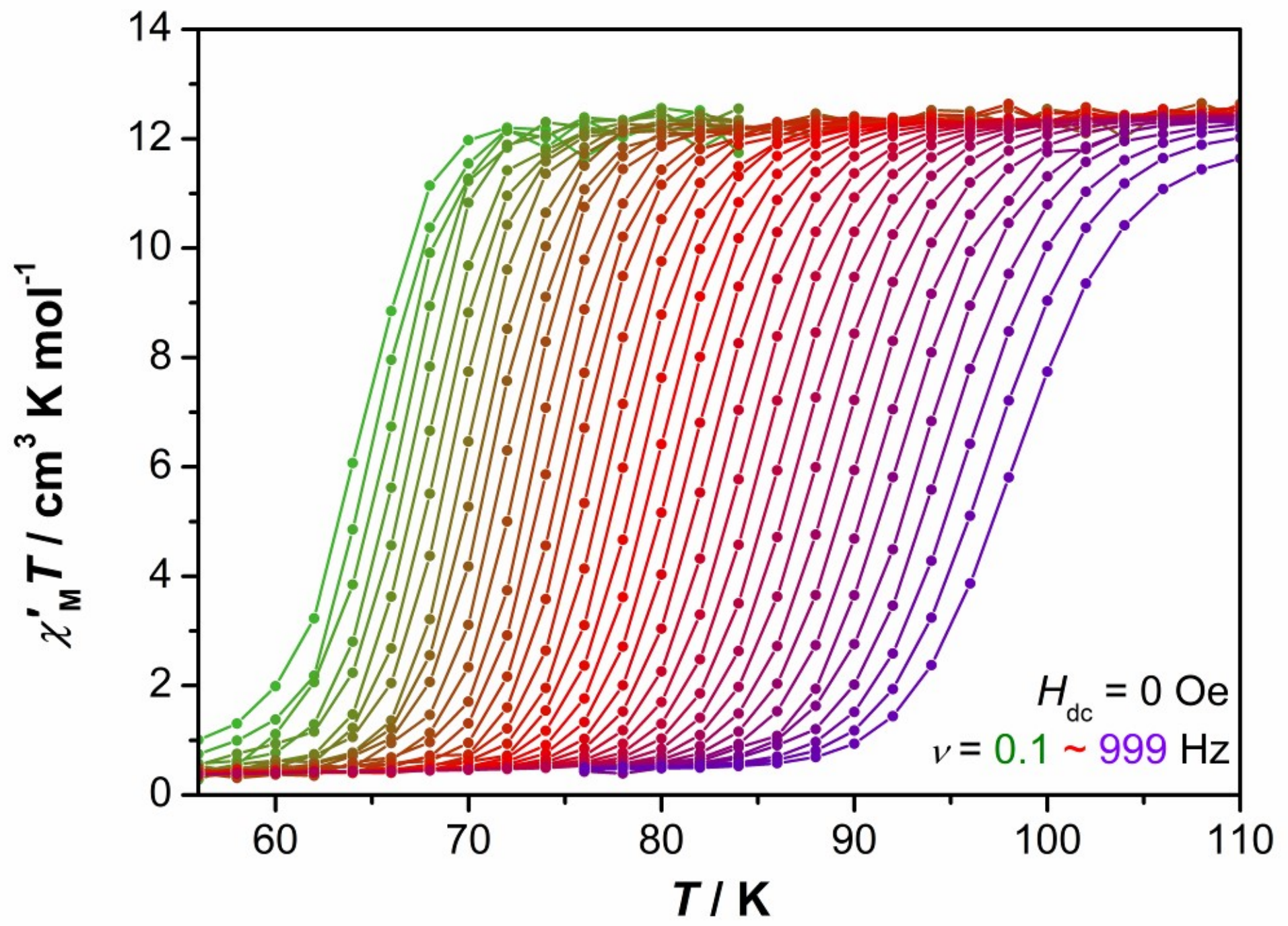

Figure S63. Temperature dependence of the in-phase susceptibility χ'_M for **3-Dy** collected under zero DC field at AC frequencies of ν = 1.1-999 Hz from 2-110 K. Solid lines are a guide to the eye.

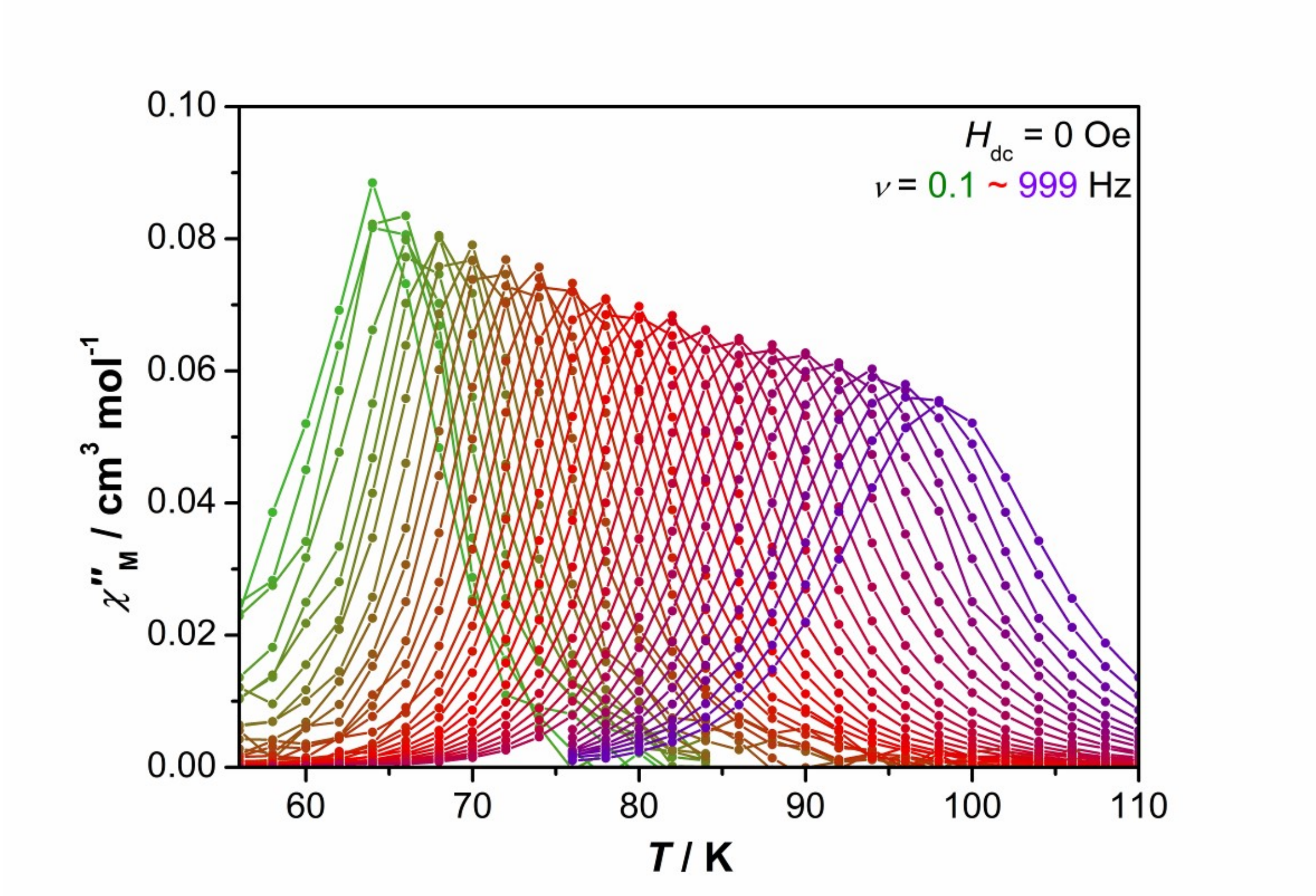

Figure S64. Temperature dependence of the out-of-phase susceptibility χ''_M for **3-Dy**, collected under zero DC field at AC frequencies of ν = 1.1-999 Hz from 2-110 K. Solid lines are a guide to the eye.

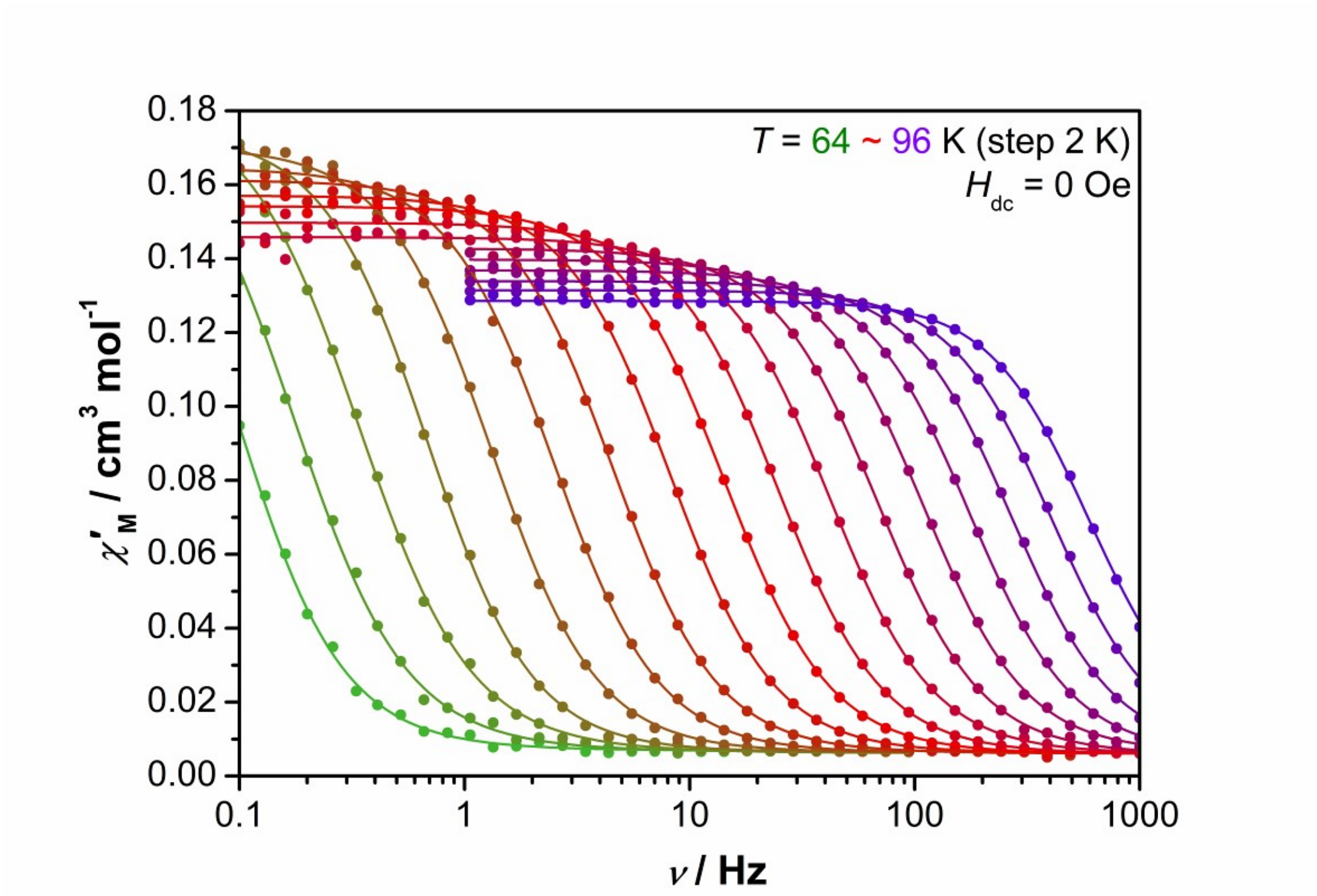

Figure S65. Frequency dependence of the in-phase susceptibility (χ'_M) for **3-Dy** in zero DC field at AC frequencies of 0.1-999 Hz from 64-96 K. Solid lines represent fits to the data using a generalized Debye equation.

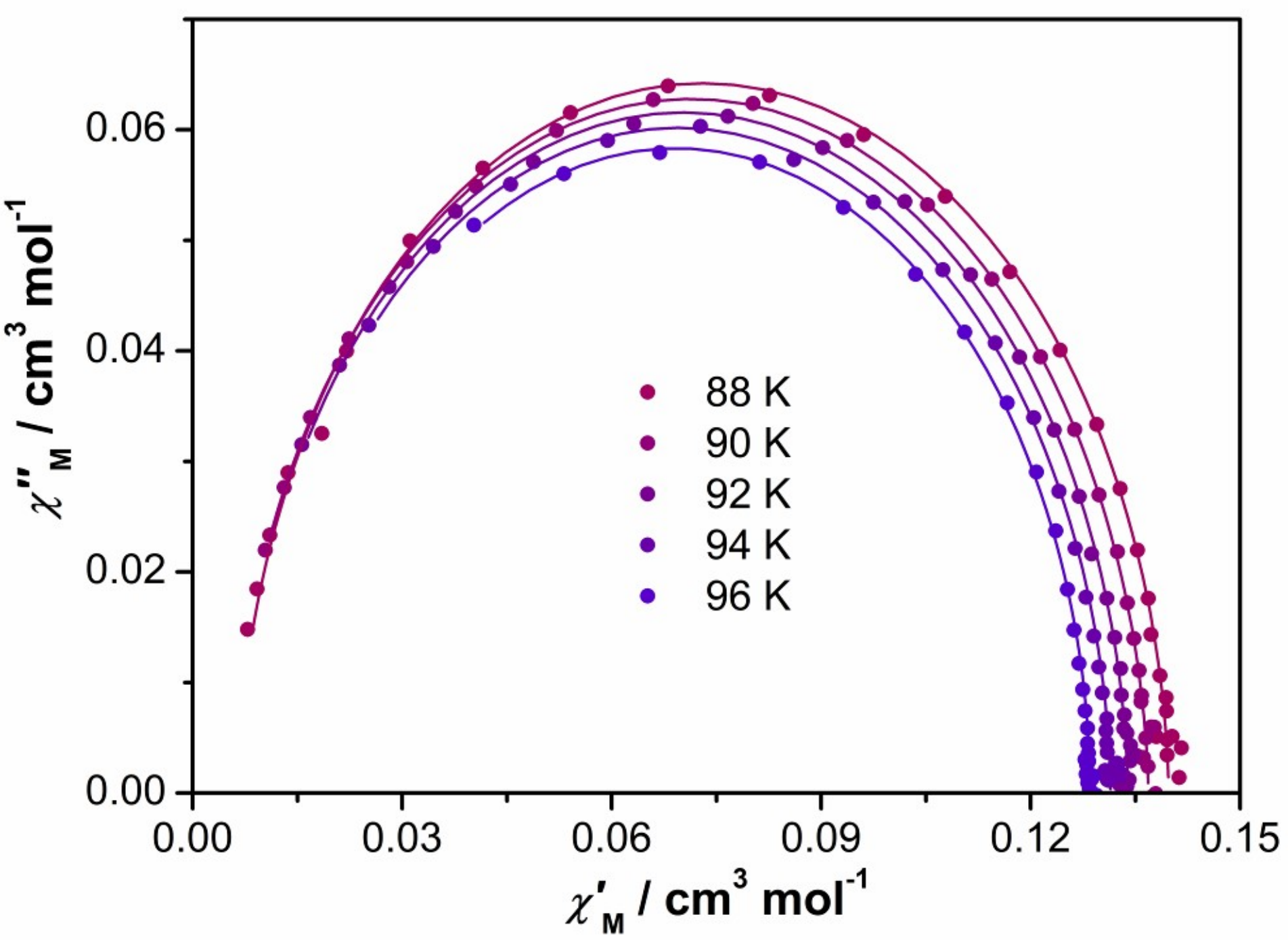

Figure S66. Cole-Cole plots for the AC susceptibilities in zero DC field for **3-Dy** from 88-96 K. Solid lines represent fits to the data using a generalized Debye equation.

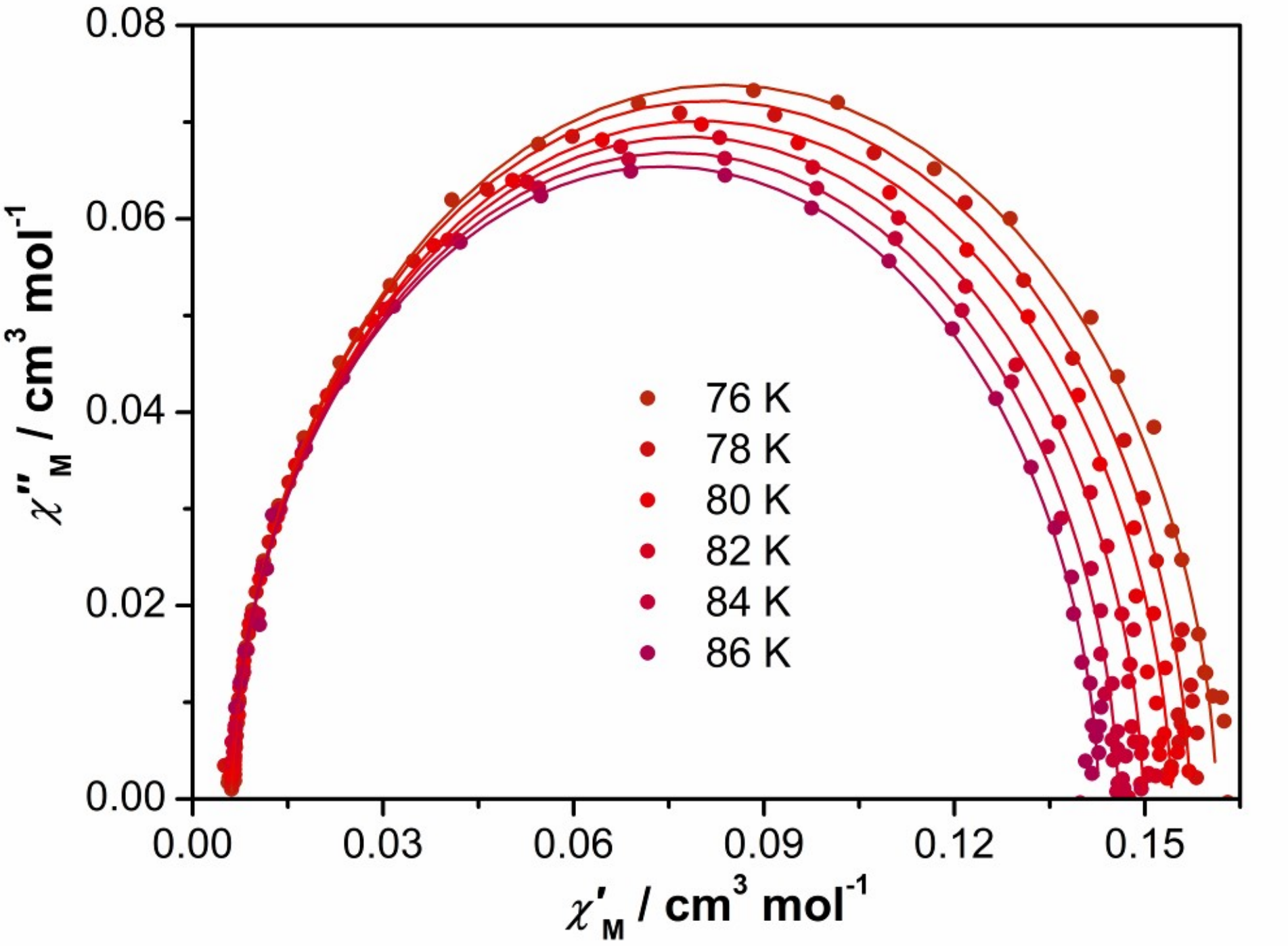

Figure S67. Cole-Cole plots for the AC susceptibilities in zero DC field for **3-Dy** from 76-86 K. Solid lines represent fits to the data using a generalized Debye equation.

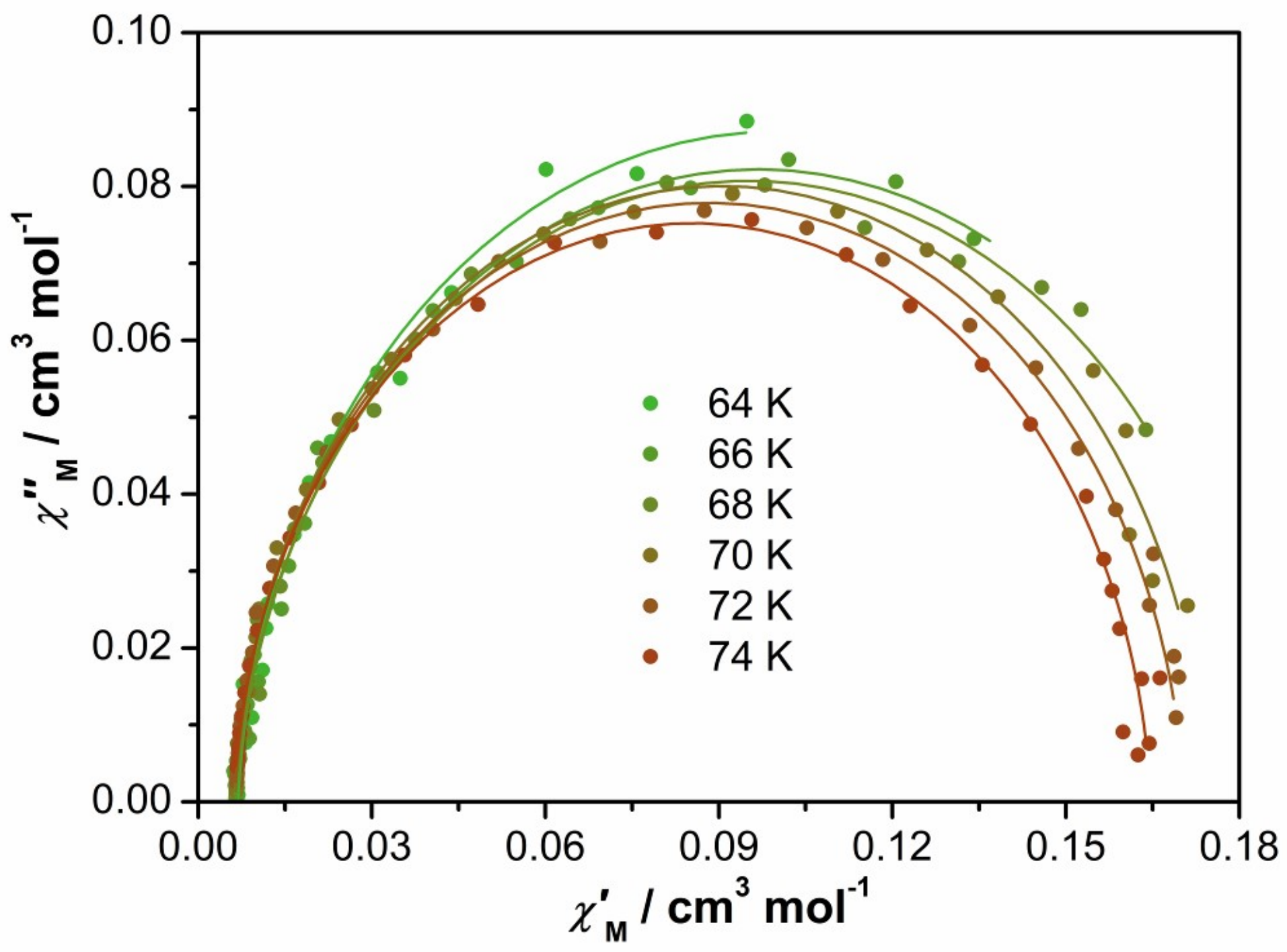

Figure S68. Cole-Cole plots for the AC susceptibilities in zero DC field for **3-Dy** from 64-74 K. Solid lines represent fits to the data using a generalized Debye equation.

Table S8. Relaxation fitting parameters for **3-Dy** corresponding to Figures S50 and S52 using the generalized Debye model.

T / K	χ_∞ / cm^3 mol^{-1}	χ_S / cm^3 mol^{-1}	τ / s	α
62	0.17001(0.0077)	0.00705(2.88E-4)	2.60214(0.15967)	0.01395(0.01363)
64	0.19266(0.0041)	0.00685 (3.06E-4)	1.68878 (0.05166)	0.04082 (0.0091)
66	0.18718(0.0017)	0.00648(2.65E-4)	0.91261(0.01217)	0.05982(0.00559)
68	0.18418(0.0011)	0.0062(3.29E-4)	0.47283(0.00468)	0.0618(0.00527)
70	0.17453(6.99E-4)	0.00637(3.36E-4)	0.2325(0.00169)	0.03124(0.00452)
72	0.17043(5.90E-4)	0.00628(3.99E-4)	0.12113(9.05E-4)	0.03339(0.00468)
74	0.16465(4.66E-4)	0.00613(4.23E-4)	0.06445(4.59E-4)	0.03319(0.00447)
76	0.1613(4.86E-4)	0.00609(3.80E-4)	0.03512(2.92E-4)	0.03151(0.00496)
78	0.15712(2.98E-4)	0.00615(2.81E-4)	0.01944(1.12E-4)	0.02823(0.00345)
80	0.15423(2.63E-4)	0.00589(2.94E-4)	0.01118(6.35E-5)	0.03561(0.00337)
82	0.14977(3.12E-4)	0.00586(4.14E-4)	0.00646(4.84E-5)	0.03143(0.00447)
84	0.14584(3.18E-4)	0.00595(5.01E-4)	0.00383(3.22E-5)	0.02886(0.00507)
86	0.14278(2.33E-4)	0.00577(3.27E-4)	0.00233(1.18E-5)	0.02934(0.0032)
88	0.13979(2.79E-4)	0.00634(4.91E-4)	0.00146(1.00E-5)	0.02435(0.00438)
90	0.13688(2.07E-4)	0.00576(4.77E-4)	9.10E-4(5.53E-6)	0.02733(0.00376)
92	0.13394(1.15E-4)	0.00657(3.50E-4)	5.91E-4(2.42E-6)	0.02143(0.00244)
94	0.13146(1.56E-4)	0.00749(6.68E-4)	3.91E-4(2.89E-6)	0.01876(0.00401)
96	0.12857(1.48E-4)	0.00982(9.23E-4)	2.66E-4(2.70E-6)	0.0112(0.00475)

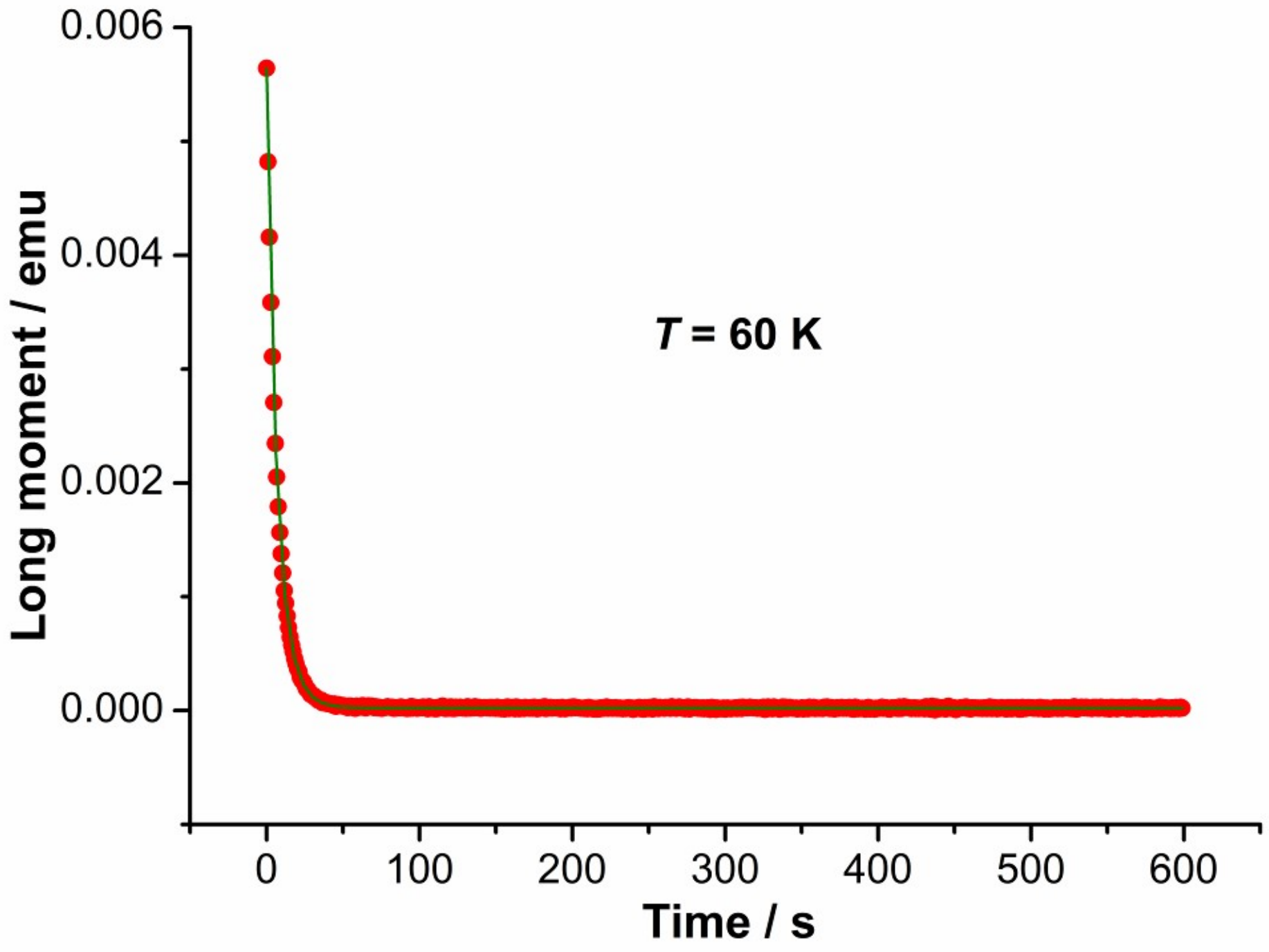

Figure S69. Plot of magnetization decay vs. time used to derive DC relaxation times for **3-Dy** at 60 K. The solid lines are the best fit to the exponential decay as $M(t) = M_f + (M_0 - M_f) \exp[(-(t/\tau)\beta]$, where M_0 is the initial magnetization, M_f is the final magnetization, τ is the relaxation time, and β is a generalized coefficient, which should be equal to 1 for an ideal exponential decay.

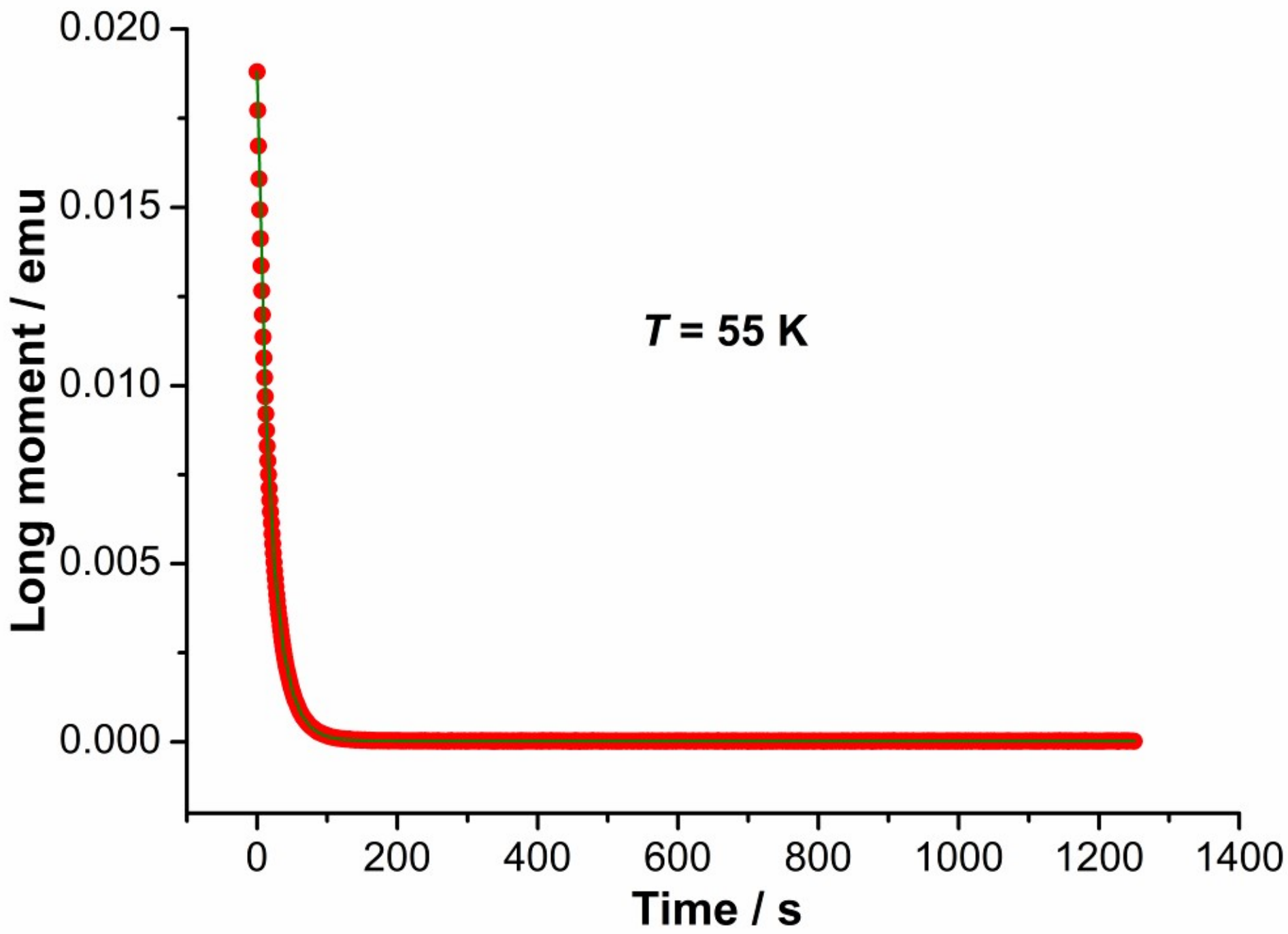

Figure S70. Plot of magnetization decay vs. time used to derive DC relaxation times for **3-Dy** at 55 K.

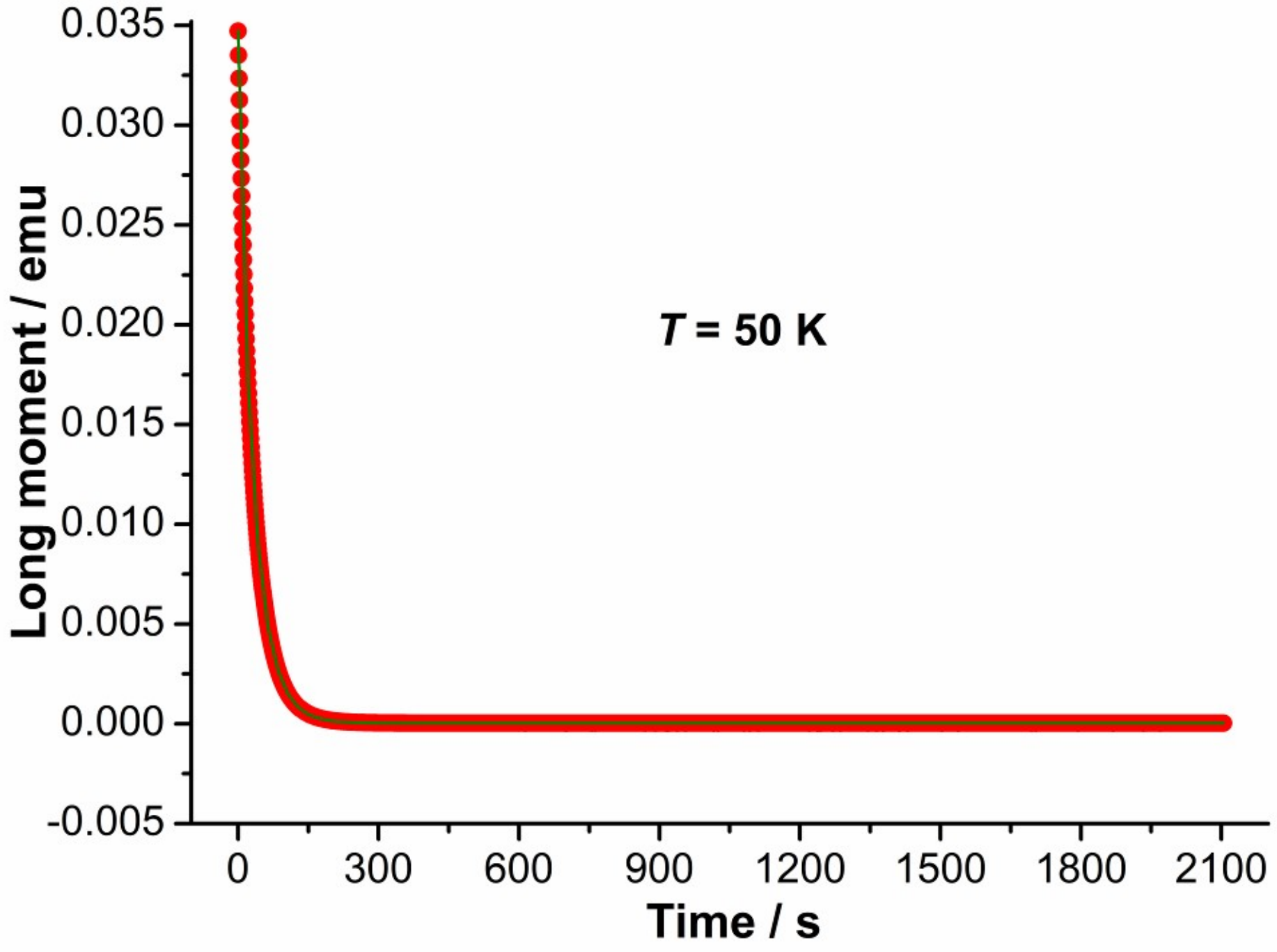

Figure S71. Plot of magnetization decay vs. time used to derive DC relaxation times for **3-Dy** at 50 K.

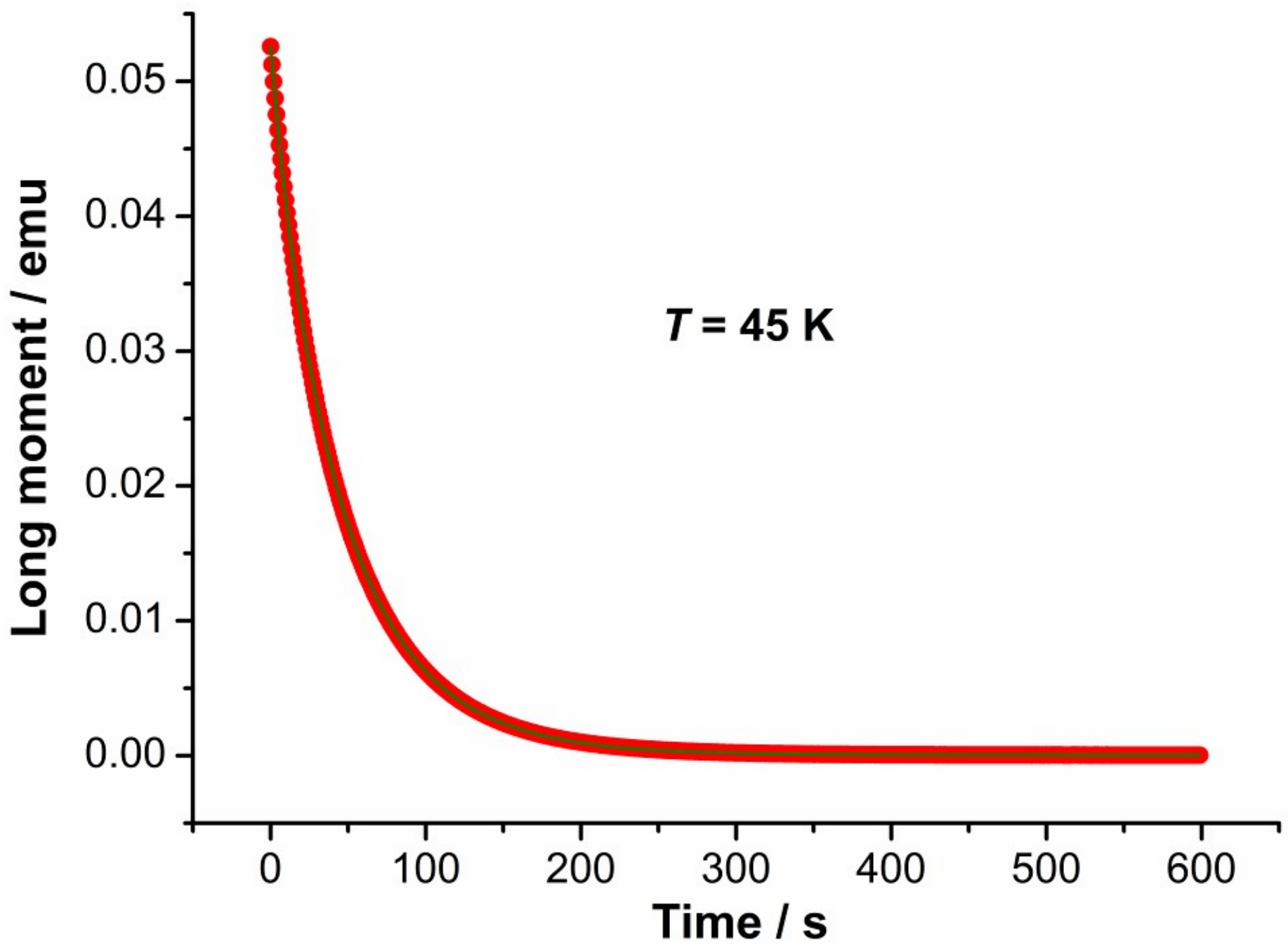

Figure S72. Plot of magnetization decay vs. time used to derive DC relaxation times for **3-Dy** at 45 K.

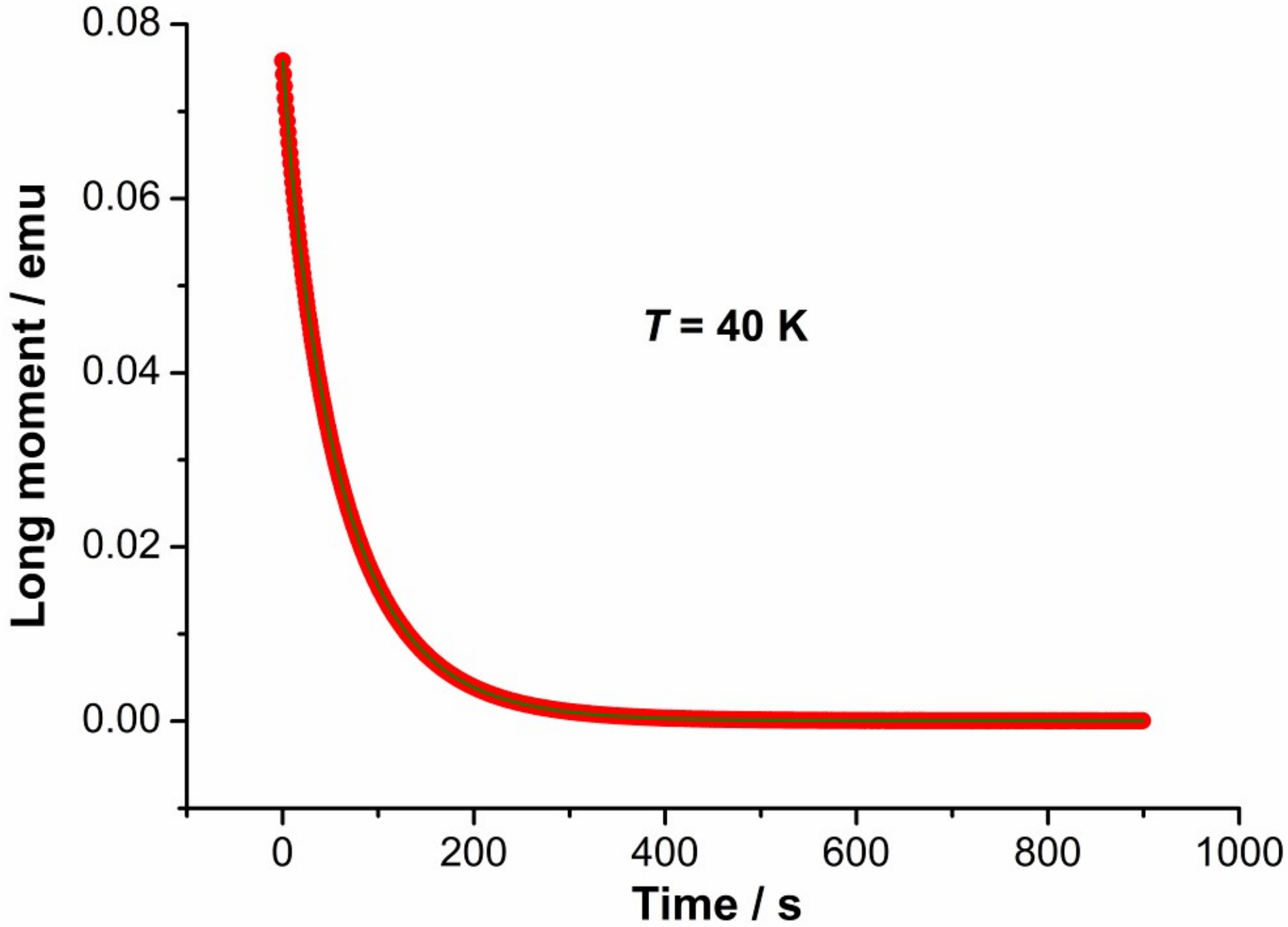

Figure S73. Plot of magnetization decay vs. time used to derive DC relaxation times for **3-Dy** at 40 K.

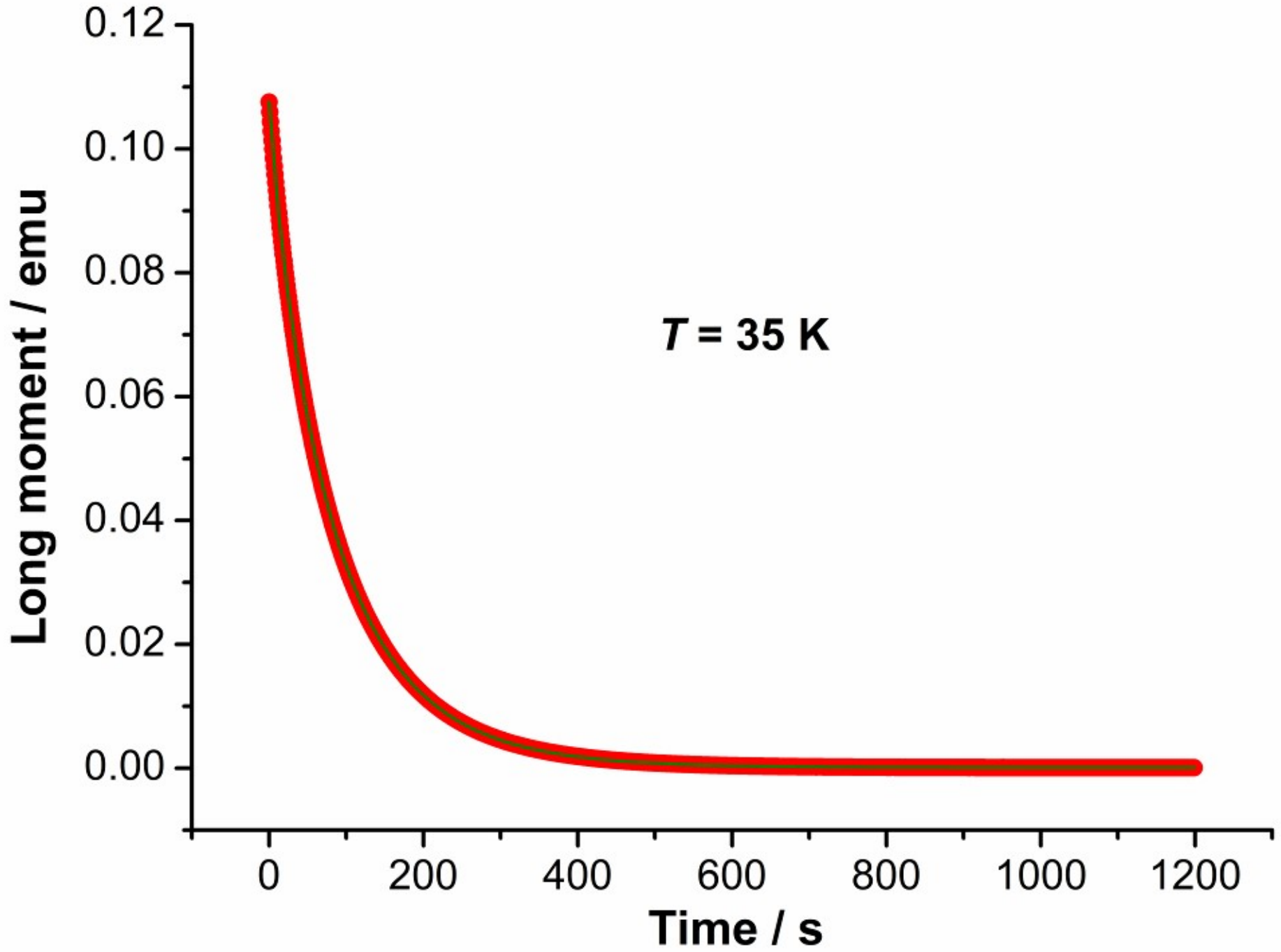

Figure S74. Plot of magnetization decay vs. time used to derive DC relaxation times for **3-Dy** at 335 K.

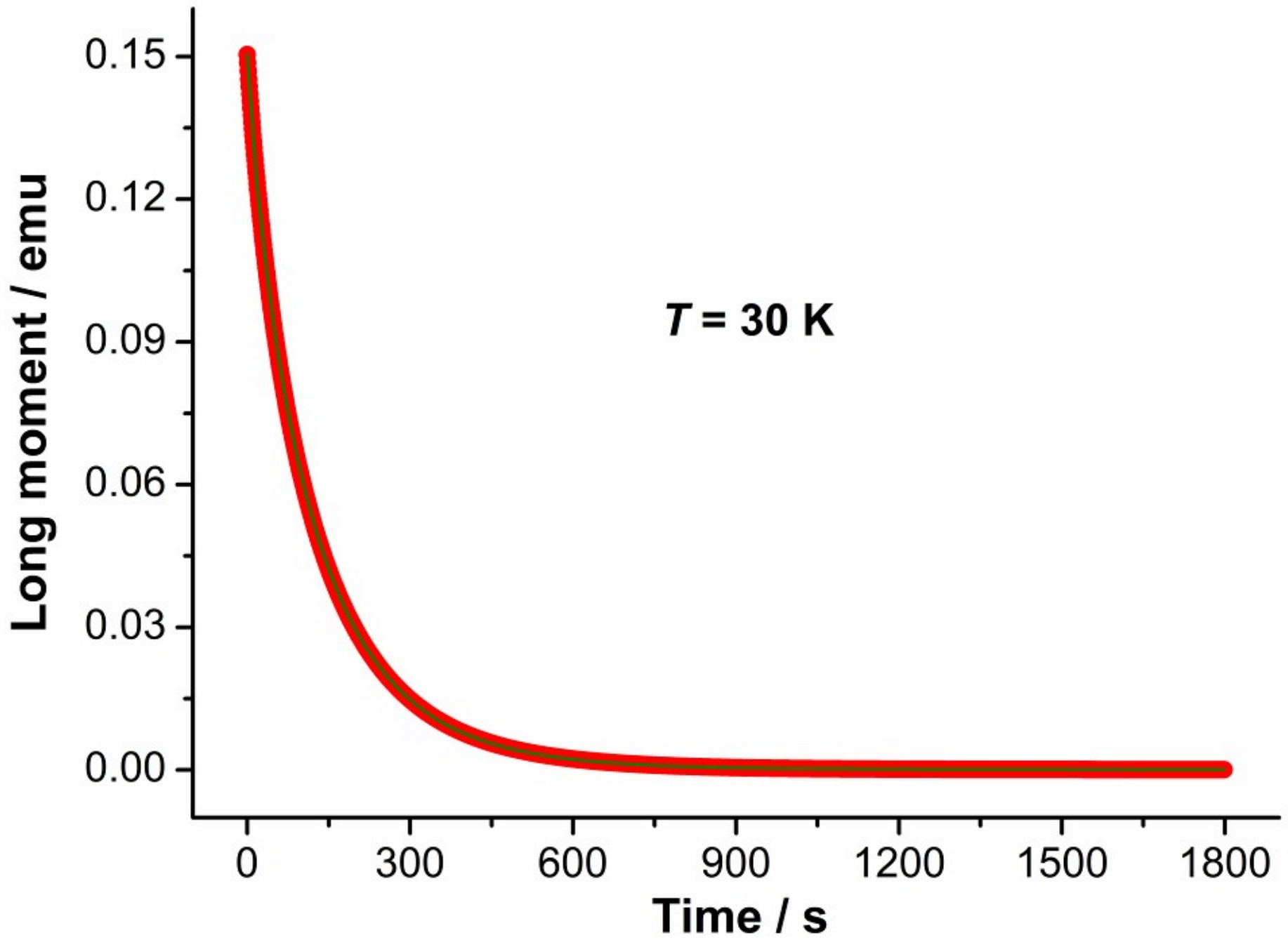

Figure S75. Plot of magnetization decay vs. time used to derive DC relaxation times for **3-Dy** at 30 K.

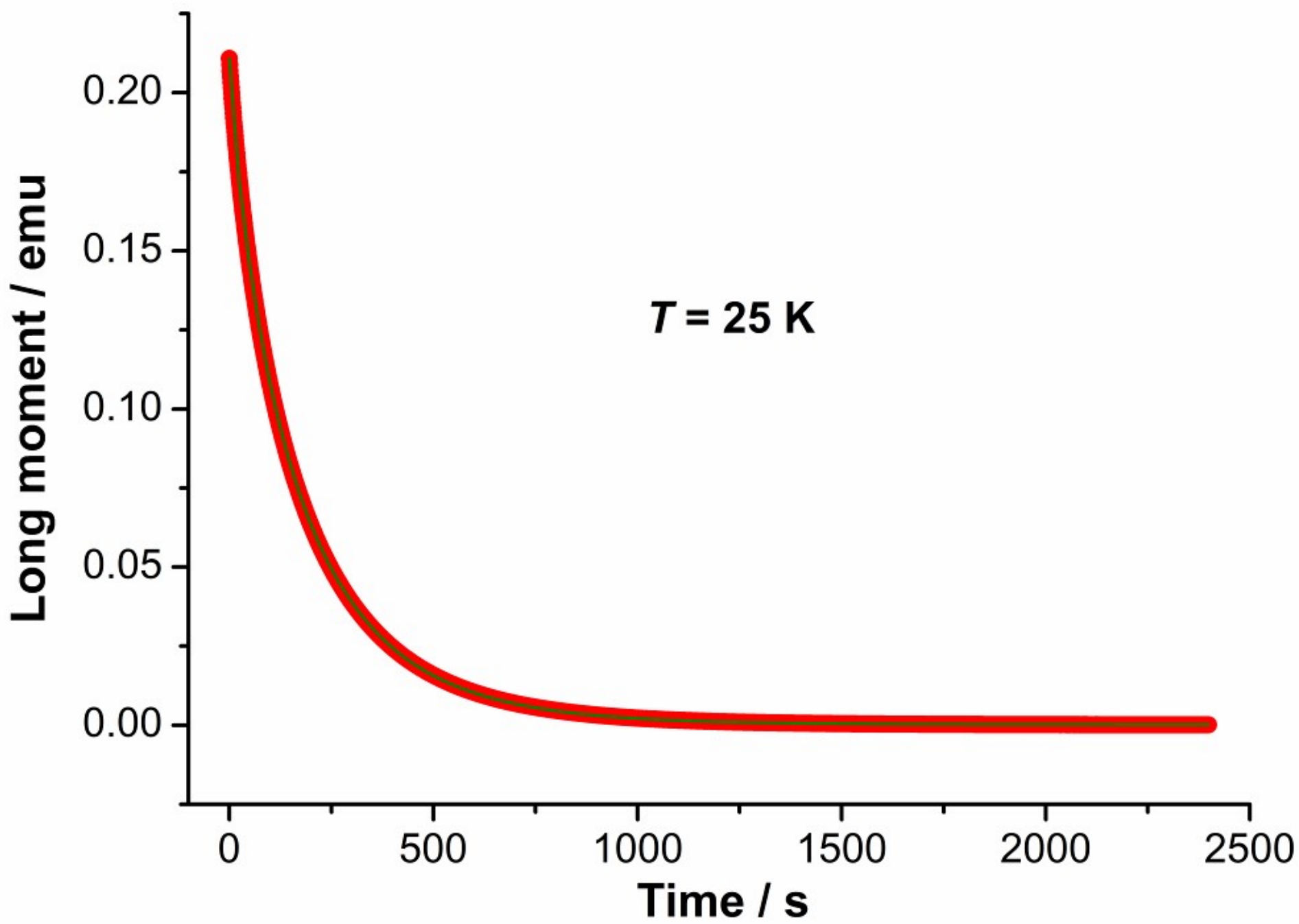

Figure S76. Plot of magnetization decay vs. time used to derive DC relaxation times for **3-Dy** at 25 K.

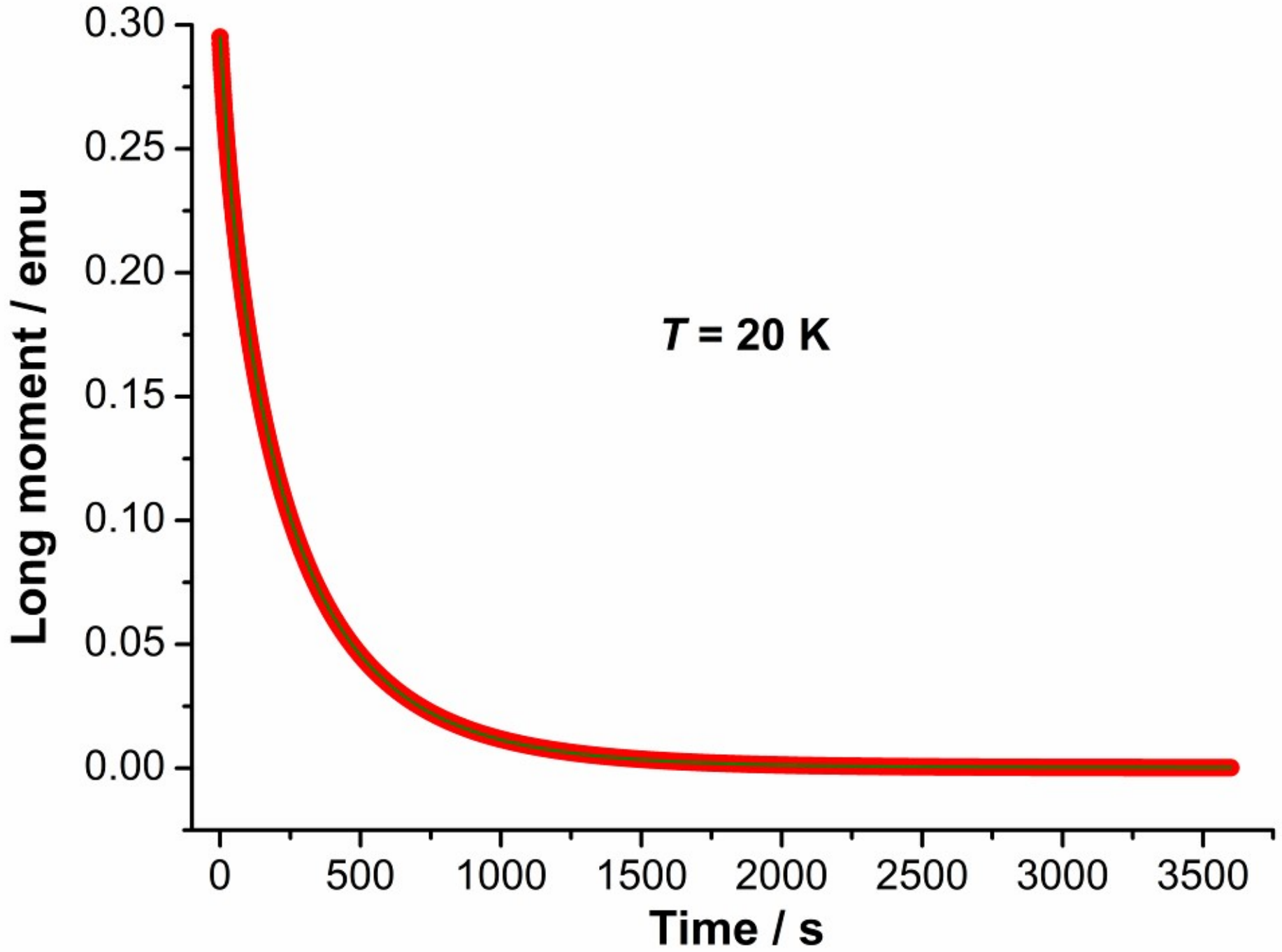

Figure S77. Plot of magnetization decay vs. time used to derive DC relaxation times for **3-Dy** at 20 K.

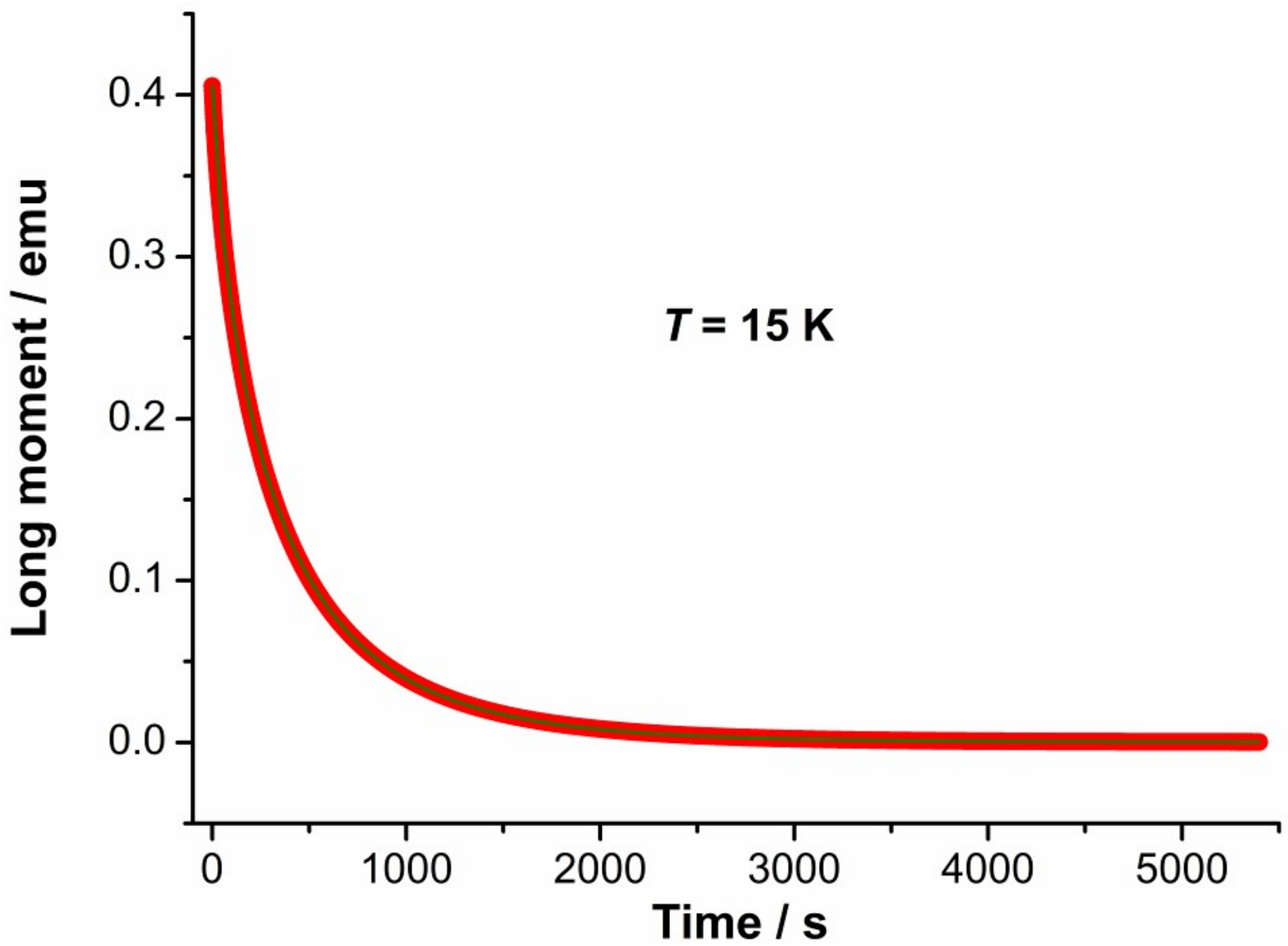

Figure S78. Plot of magnetization decay vs. time used to derive DC relaxation times for **3-Dy** at 15 K.

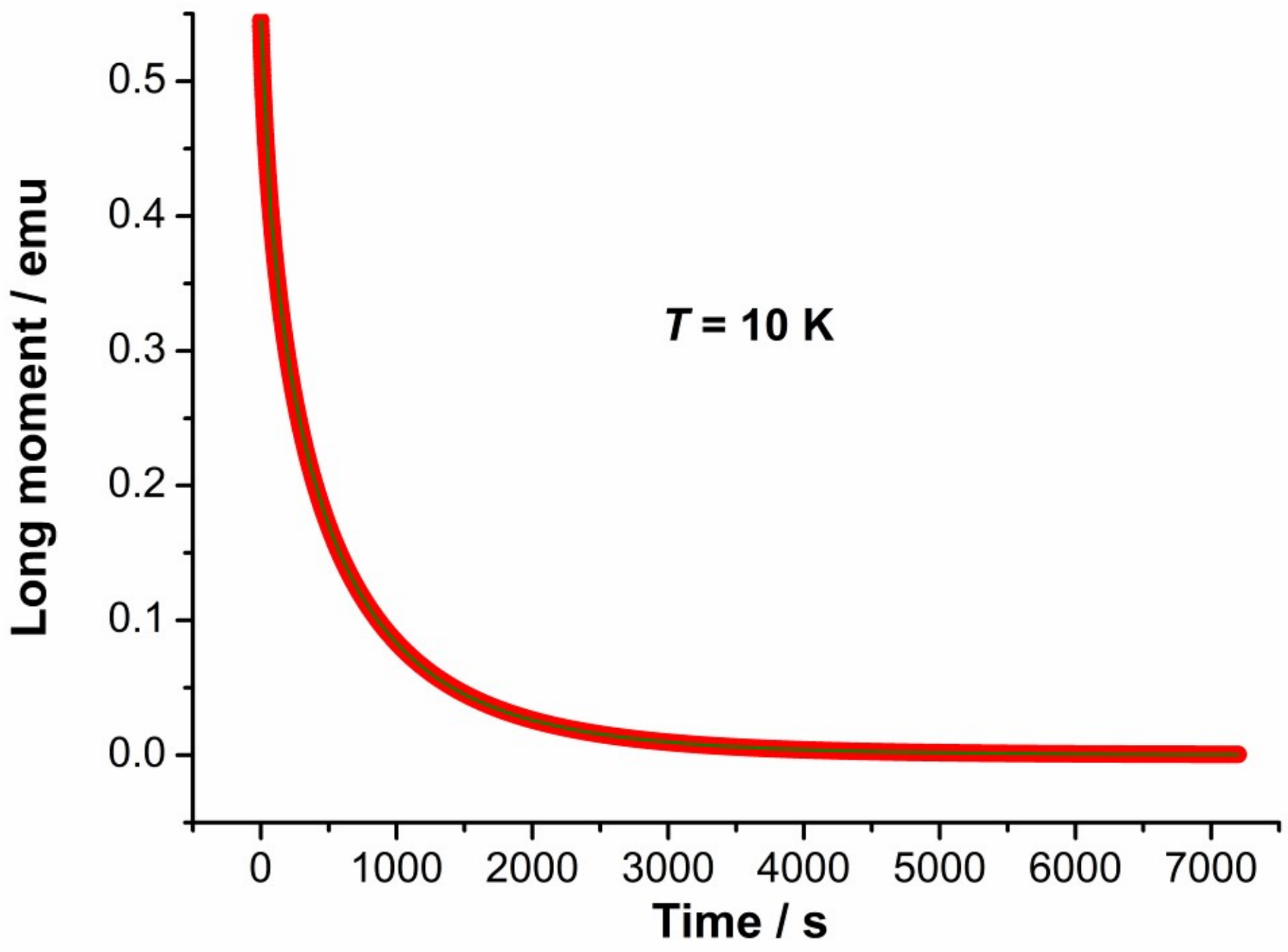

Figure S79. Plot of magnetization decay vs. time used to derive DC relaxation times for **3-Dy** at 10 K.

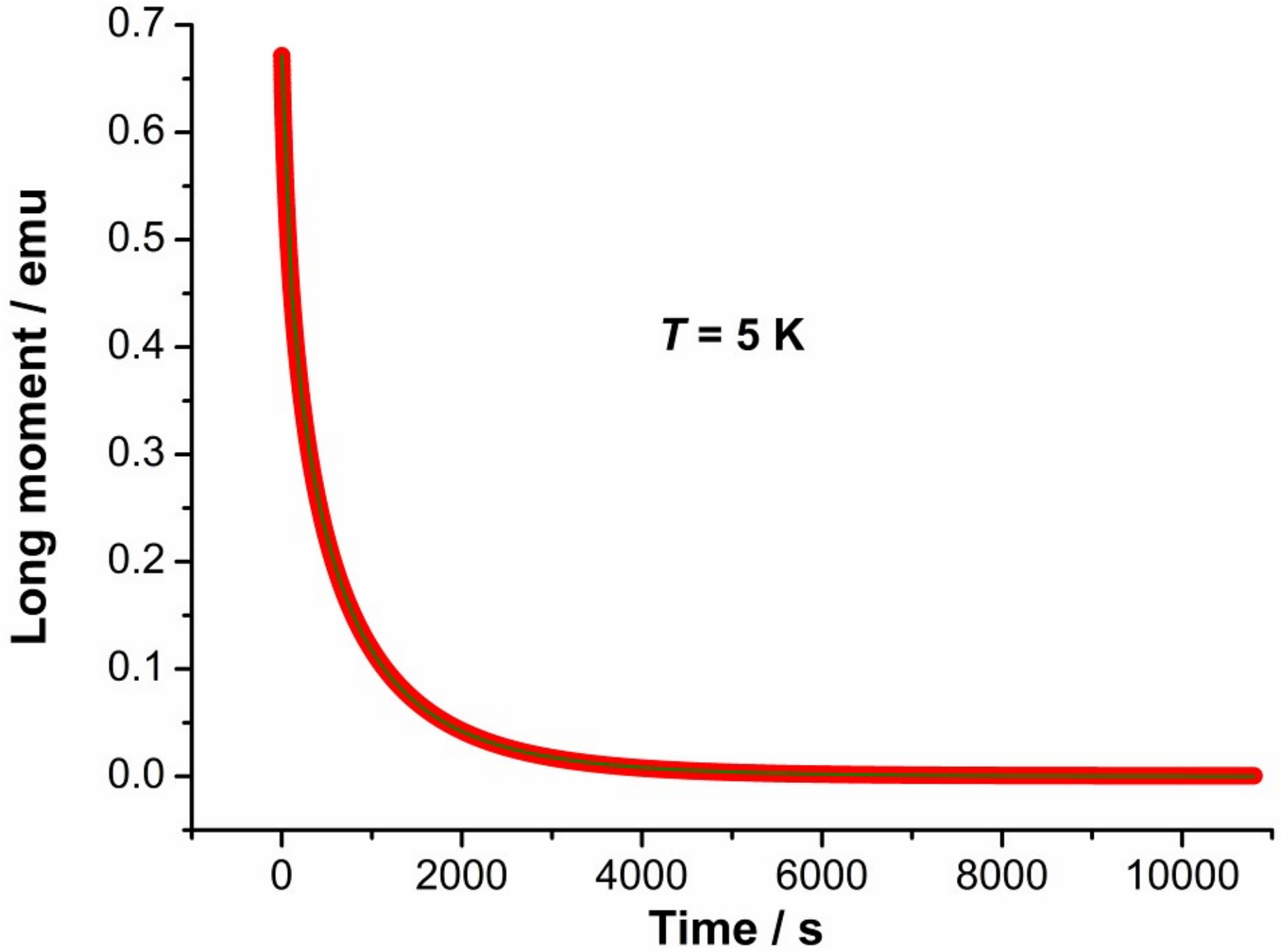

Figure S80. Plot of magnetization decay vs. time used to derive DC relaxation times for **3-Dy** at 5 K.

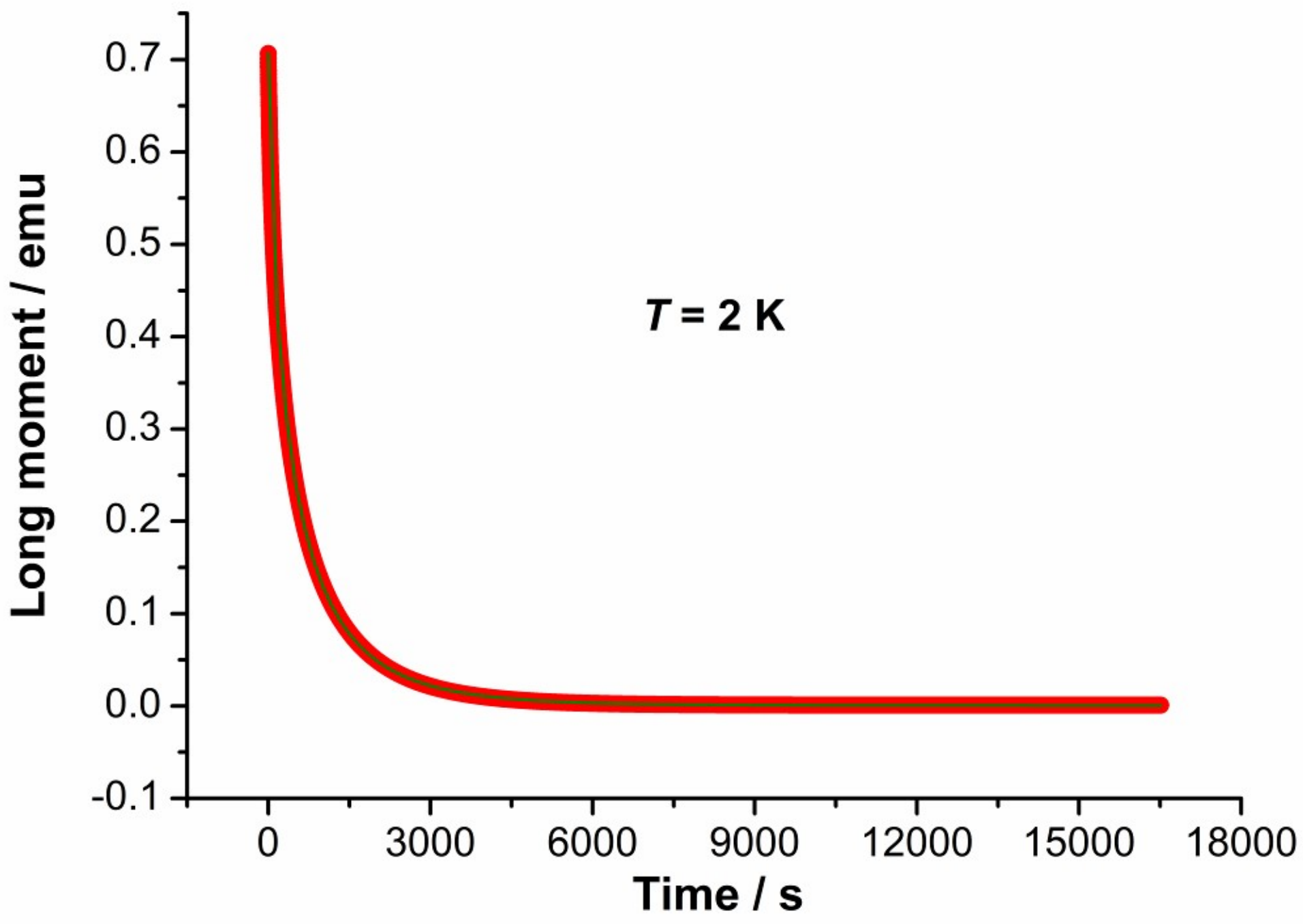

Figure S81. Plot of magnetization decay vs. time used to derive DC relaxation times for **3-Dy** at 2 K.

Table S9. Fitting parameters (initial magnetization (M_0), final magnetization (M_f), relaxation time (τ), and generalized coefficient (β)) at different temperatures from the least-squares fitting to the exponential decay as $M(t) = M_f + (M_0 - M_f) \exp[(-(t/\tau)\beta]$, as shown in Figures S70-82.

T / K	M_f	M_0	τ / s	β
60	2.30122E-5	0.00564	6.63643	0.92675
55	2.6875E-5	0.0188	18.0592	0.9376
50	3.15437E-5	0.03472	30.8568	0.93153
45	5.68813E-5	0.05258	43.93305	0.92295
40	6.42142E-5	0.0758	59.95587	0.90918
35	9.76518E-5	0.10757	81.96395	0.89361
30	1.32845E-4	0.15043	113.78607	0.87283
25	1.97887E-4	0.21086	160.47718	0.84422
20	2.64387E-4	0.29506	228.66183	0.80245
15	3.31346E-4	0.40548	322.85714	0.75245
10	9.77855E-5	0.54488	402.35295	0.69467
5	0	0.67146	430.84086	0.66409
2	5.13732E-4	0.7066	453.32779	0.662

Table S10. Remanent magnetization and coercive fields at different temperatures and field sweep rates.

T / K	Field sweep speed / Oe s^{-1}	Remanent magnetization / $N\beta$	Coercive field/ Oe
2	700	4.639	39085
2	350	4.715	33189
2	200	4.542	29104
2	100	4.391	24561
2	50	4.114	20906
2	25	3.816	17857
5	200	4.366	28913
10	200	3.515	27151
15	200	2.641	24751
20	200	1.901	22040
25	200	1.369	19178
30	200	0.983	16120
35	200	0.702	13209
40	200	0.499	10508
45	200	0.349	7907
50	200	0.234	5658
55	200	0.130	3247
60	200	0.042	1197
65	200	0.011	237
60	25	0.004	111

Computational details

1. DFT and TD-DFT Calculations

Density functional theory (DFT) calculations were performed on the coordinates obtained from the X-ray structure using the ORCA 6.0.0 software package.[9, 10] The position of hydrogen atoms was optimized at the DFT level using pure GGA PBE exchange-correlation function keeping constant the position of other atoms.[11] We have used TPSSh functional[12, 13] and the relativistic effects were included with the Douglas-Kroll-Hess Hamiltonian (DKH), together with the scalar relativistic contracted version of the basis functions def2-QZVP for La, def2-TZVP for Y, Fe, Mg, and def-SVP for B, C and H atoms.[14, 15] The SARC/J auxiliary basis set[16] and RIJCOSX approximation[17, 18] with TightSCF convergence threshold were also used throughout the calculations. The TD-DFT calculations were performed with 70, 80, 90 and 150 excited states for **1**, **2**, **3-La** and **3-Y**, respectively, with a CPCM implicit solvent model for THF (**2**) and hexene (**1**, **3-La** and **3-Y**).[19] To probe the aromaticity, the nucleus-independent chemical shift (NICS)[20] and isochemical shielding surface (ICSS)[21] calculations were performed using the Gaussian09d[22] program suite with B3LYP functional.[23-25] The Stuttgart energy-consistent pseudo-potentials and their optimized valence basis sets were utilized (ECP10MDF for Fe, ECP28MWB for La, ECP28MWB for Y) for metal elements, and the 6-31G* basis set for other elements.

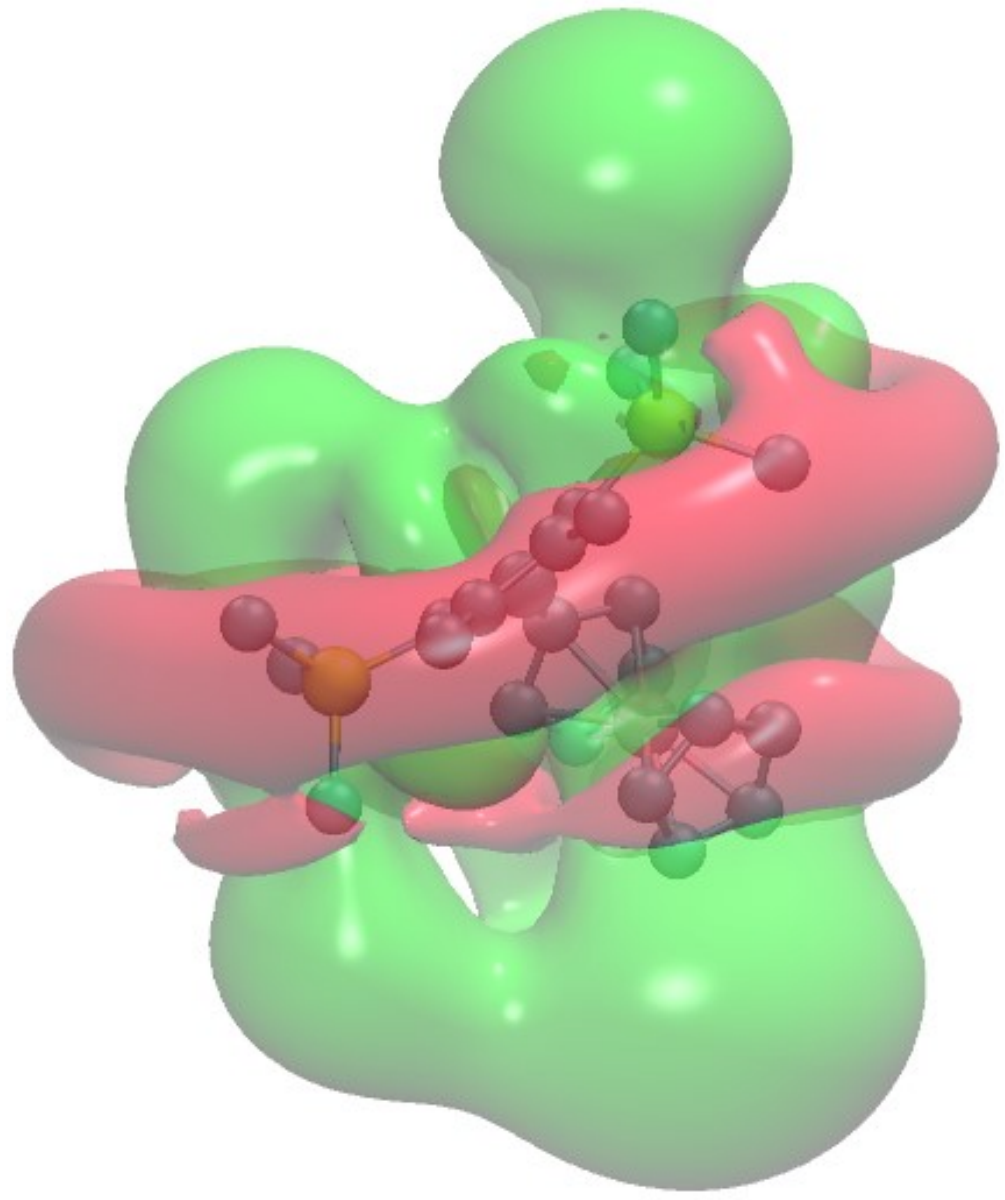

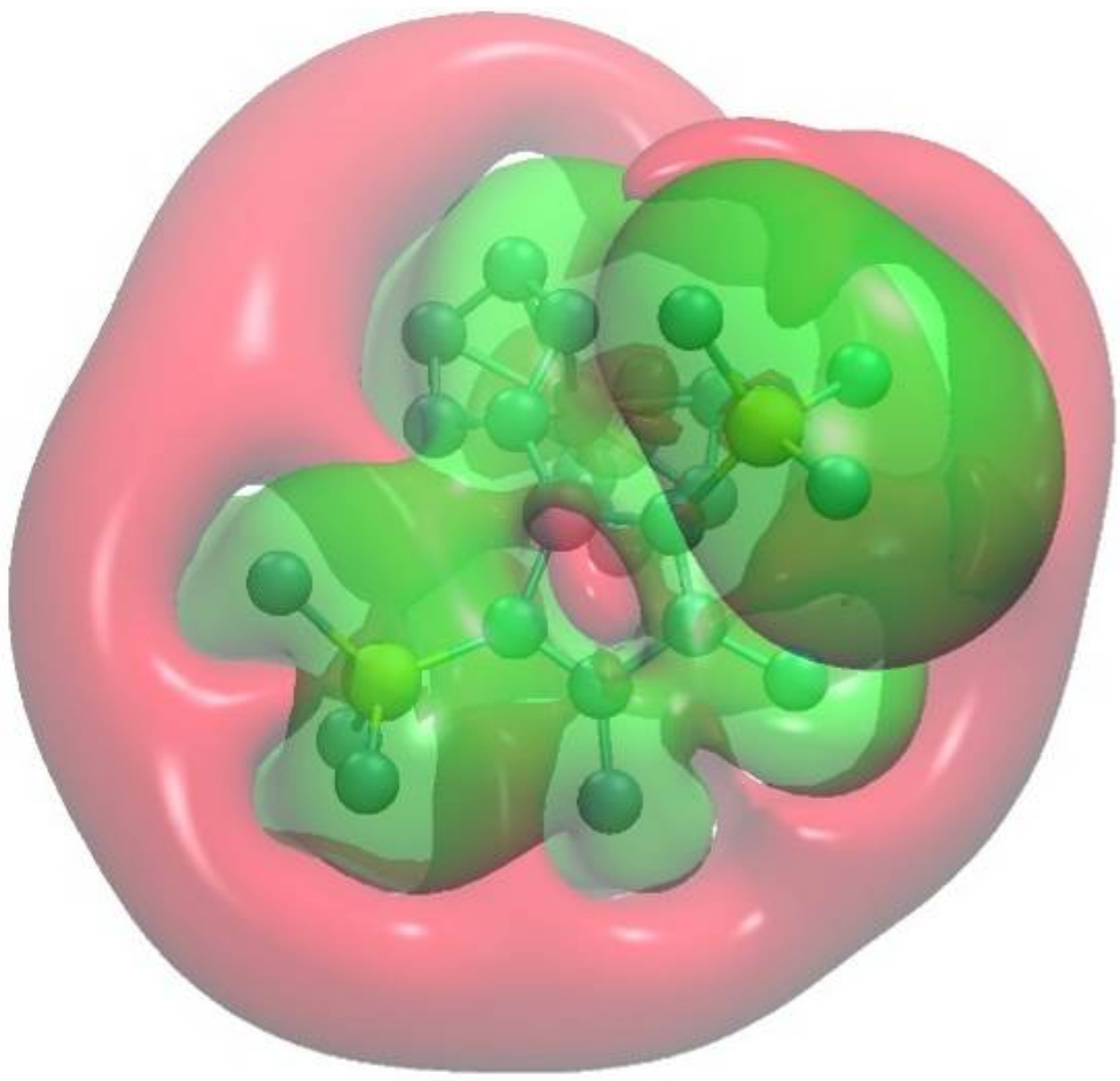

Figure S82. ICSS$_{ZZ}$ plots of **1** in side and top views exhibiting shielding (green) and deshielding (red) surfaces.

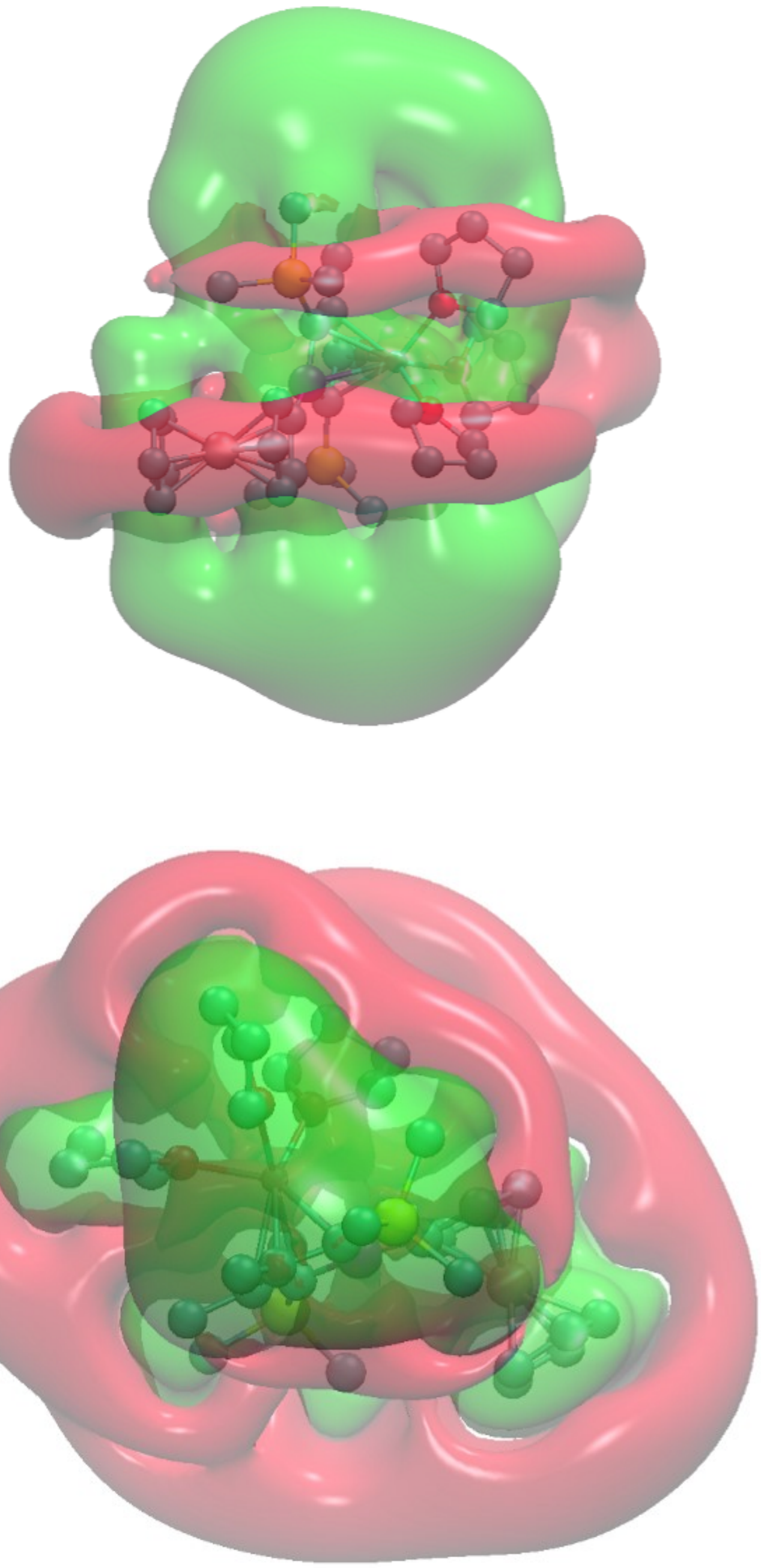

Figure S83. ICSS$_{ZZ}$ plots of **2** in side and top views exhibiting shielding (green) and deshielding (red) surfaces.

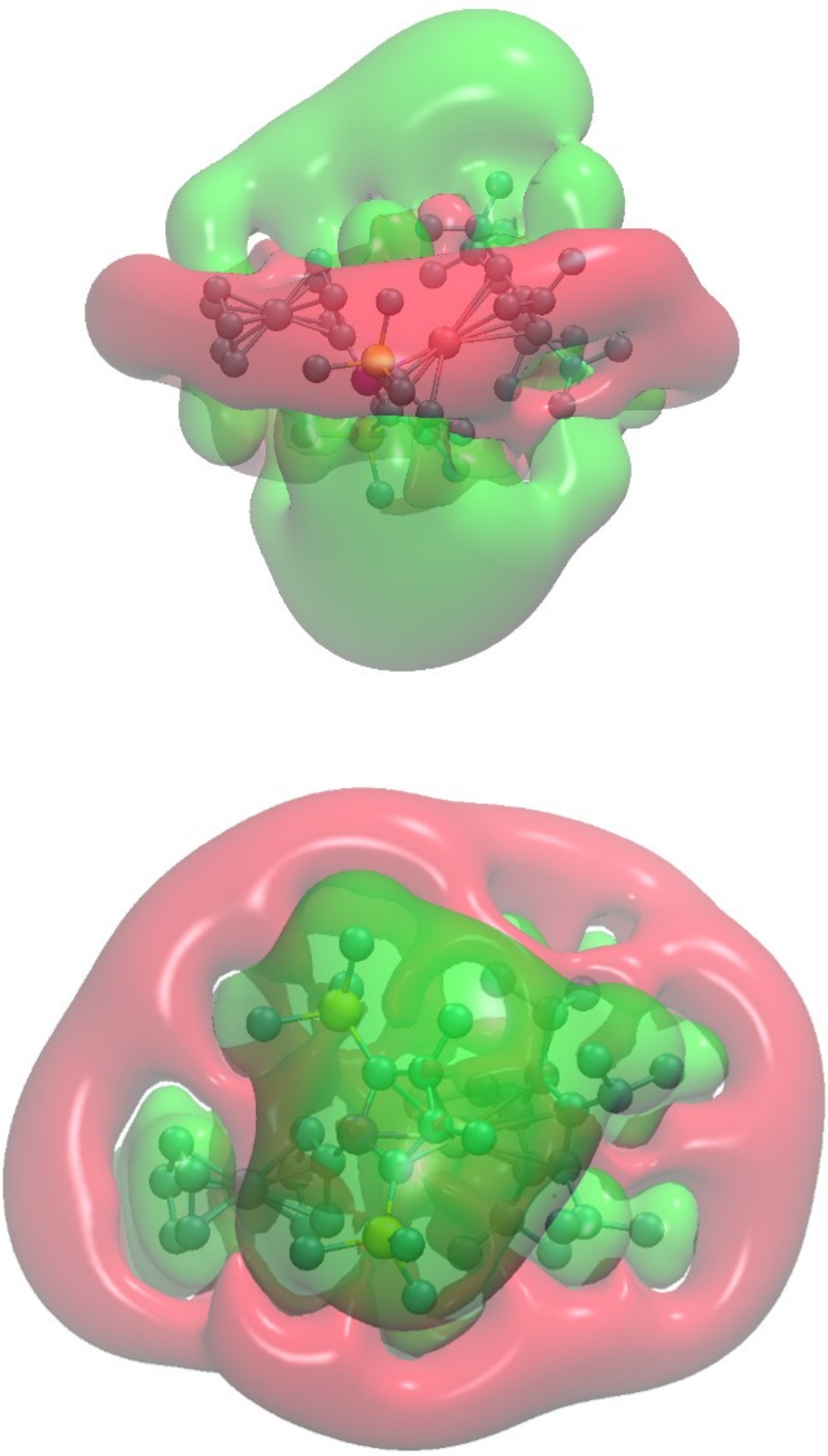

Figure S84. ICSS$_{ZZ}$ plots of **3-La** in side and top views exhibiting shielding (green) and deshielding (red) surfaces.

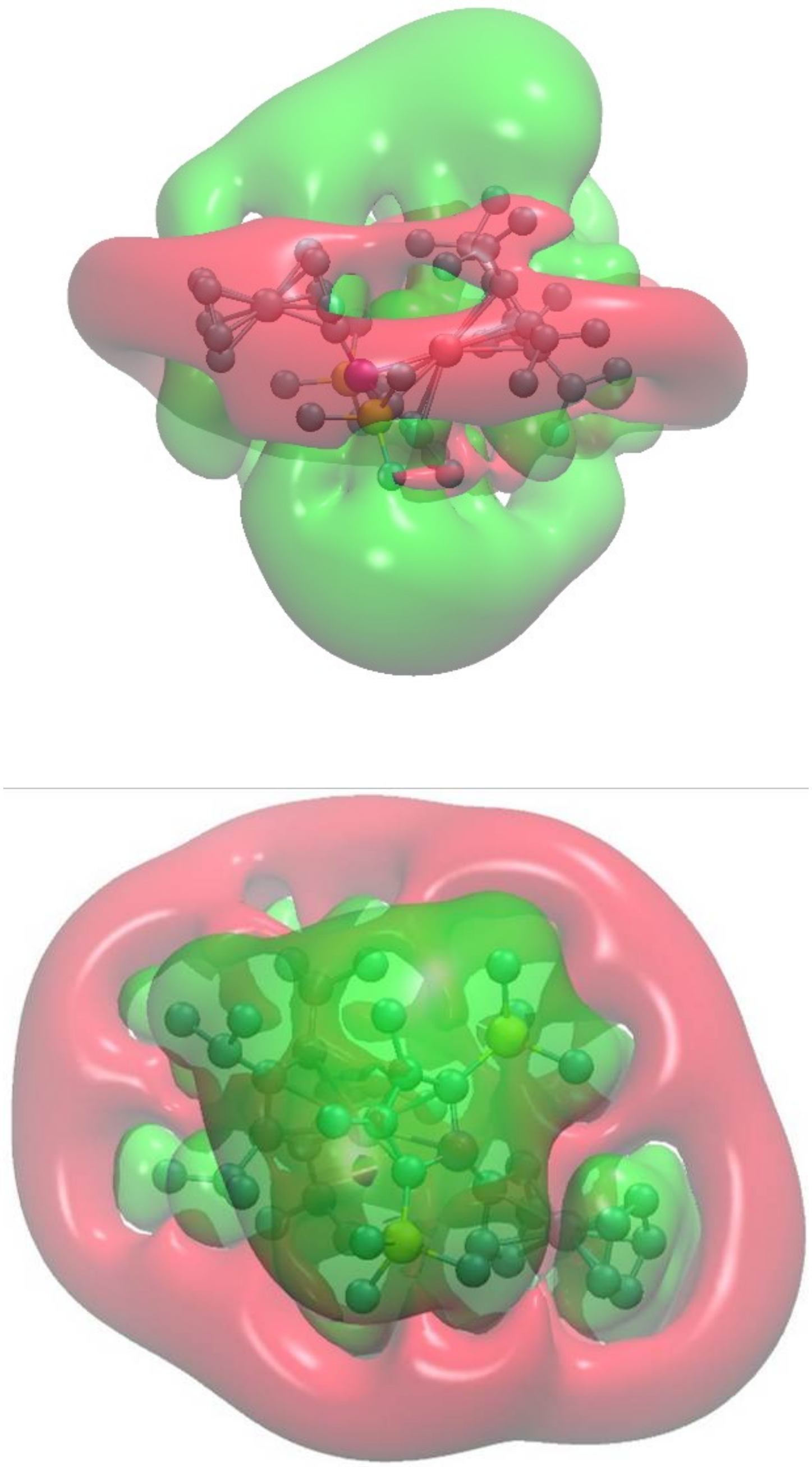

Figure S85. $ICSS_{ZZ}$ plots of **3-Y** in side and top views exhibiting shielding (green) and deshielding (red) surfaces.

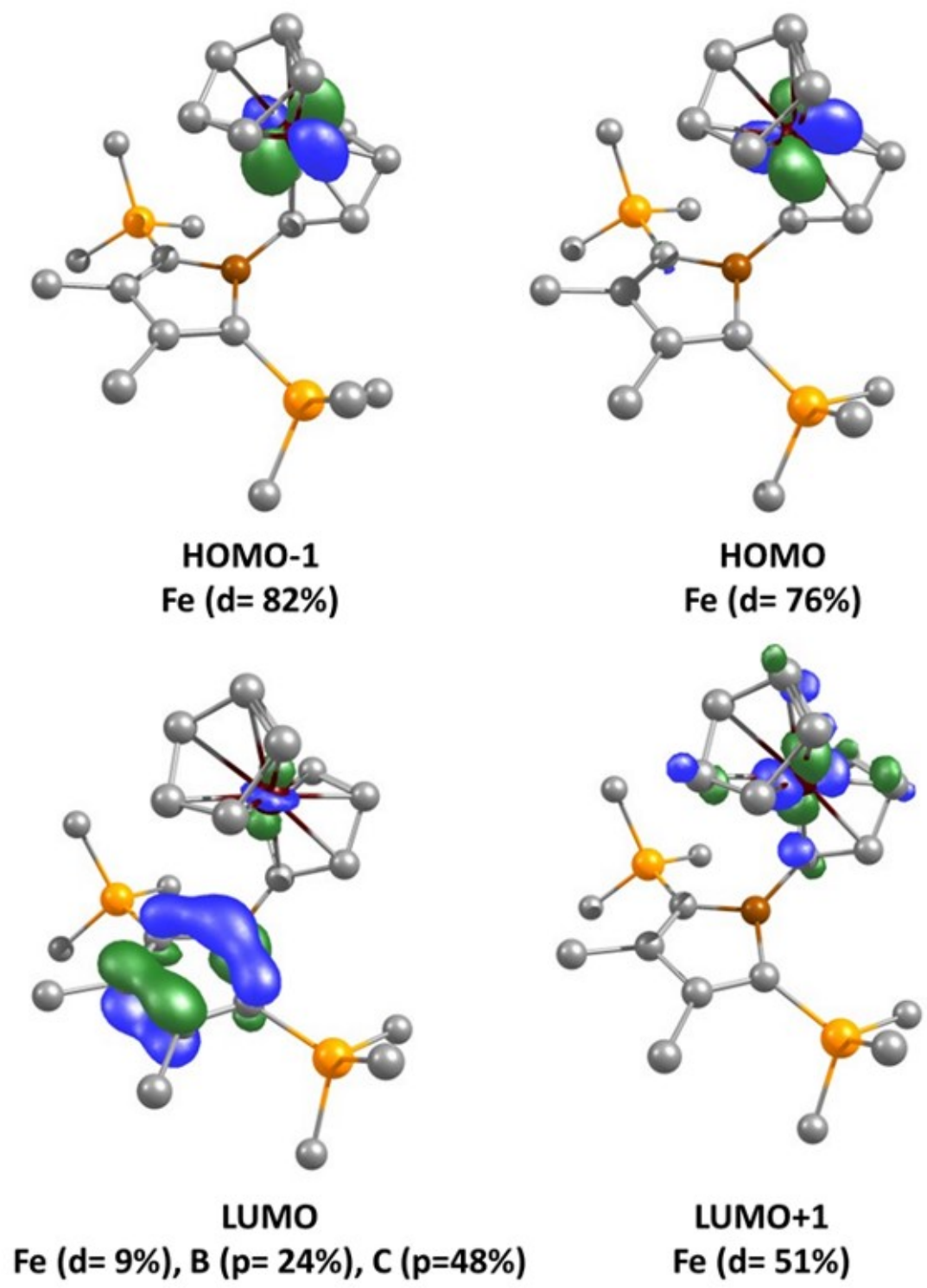

Figure S86. DFT calculated frontier molecular orbital and orbital composition (only from Fe and borole ligand) for **1** using TPSSh/def2-TZVP/def2-SVP level of theory (iso value = 0.07).

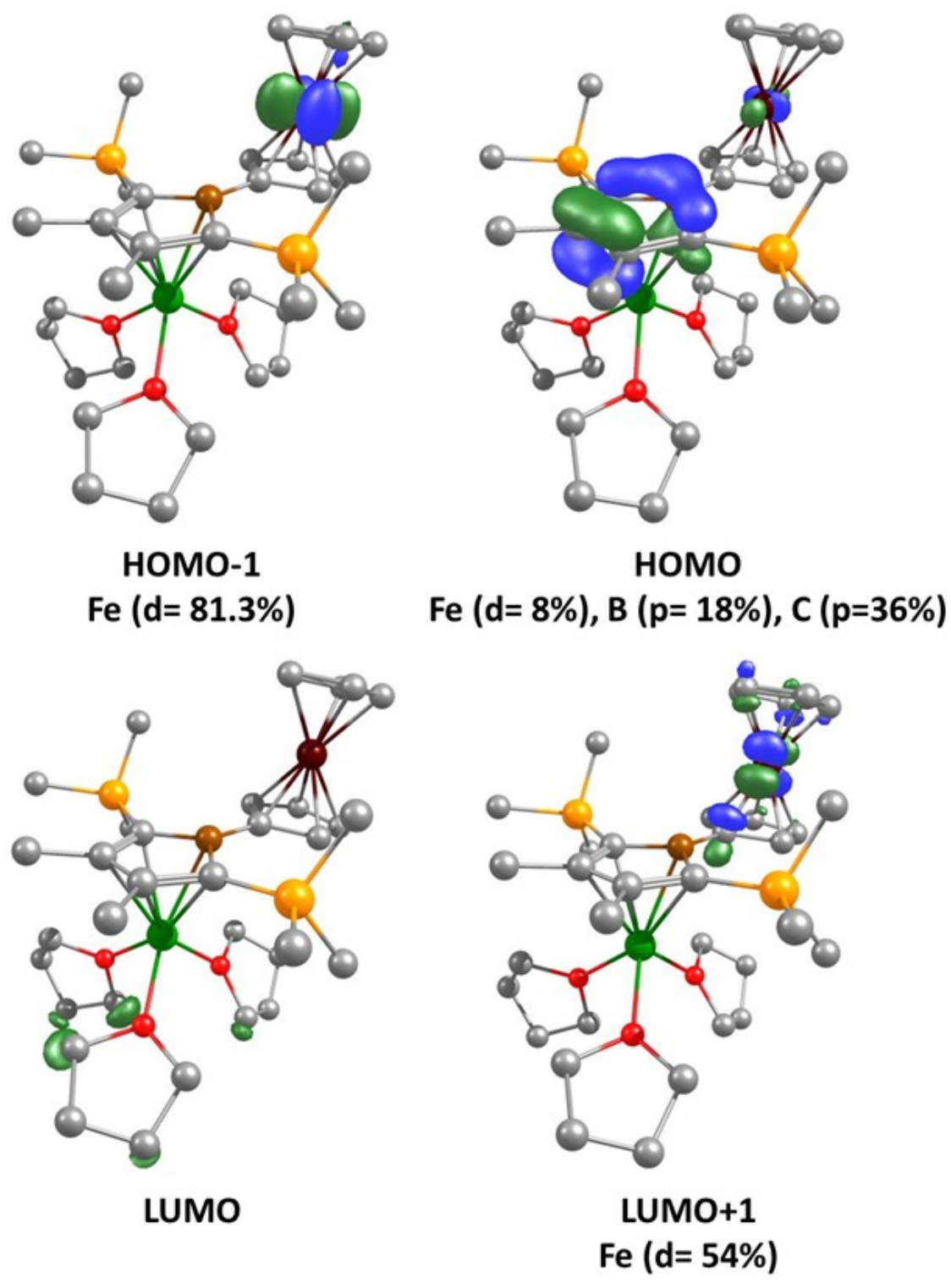

Figure S87. DFT calculated frontier molecular orbital and orbital composition (only from Fe and borole ligand) for **2** using TPSSh/def2-TZVP/def2-SVP level of theory (iso value = 0.07).

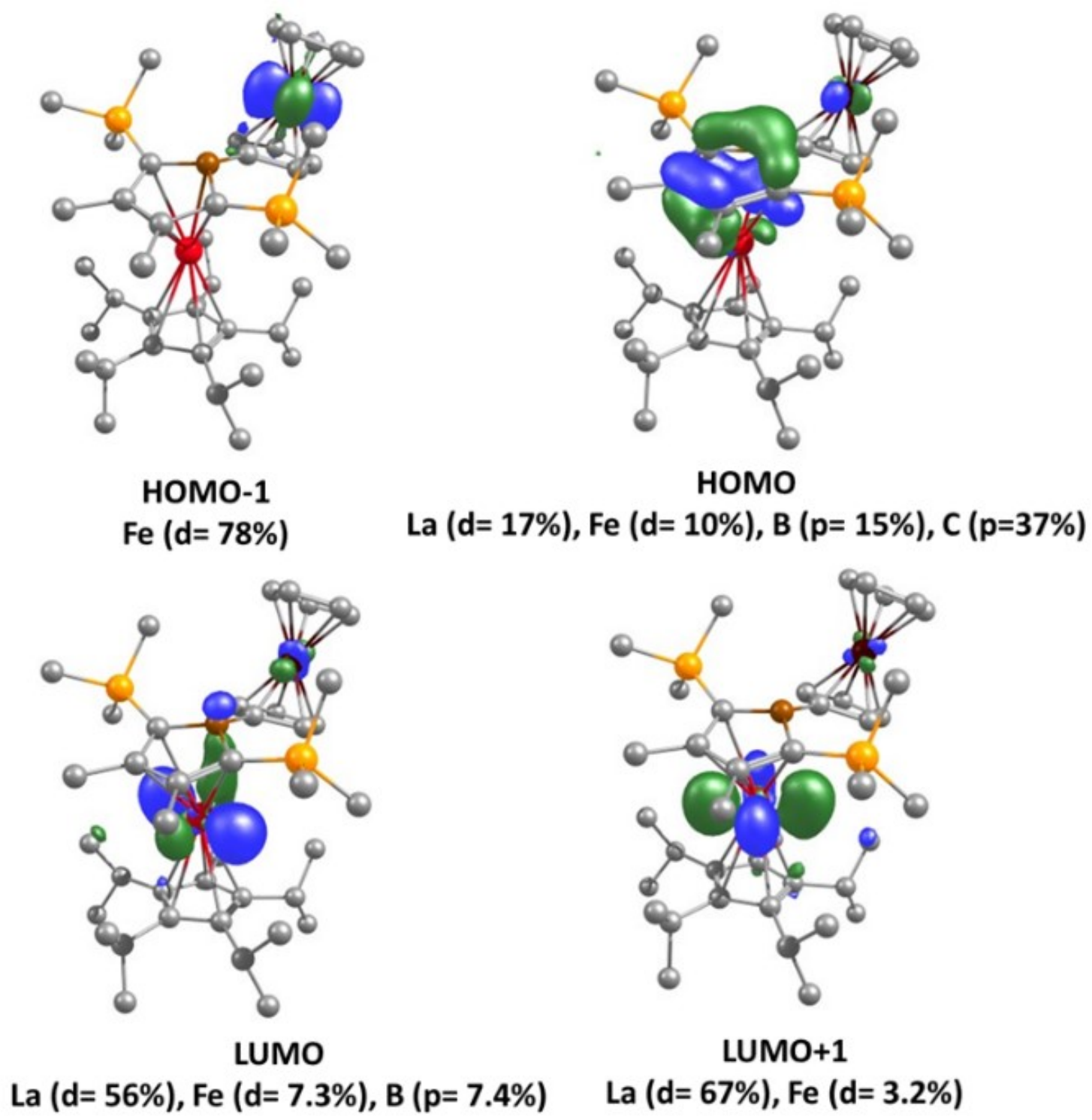

Figure 88. DFT calculated frontier molecular orbital and orbital composition (only from La, Fe and borole ligand) for **3-La** (iso value = 0.057).

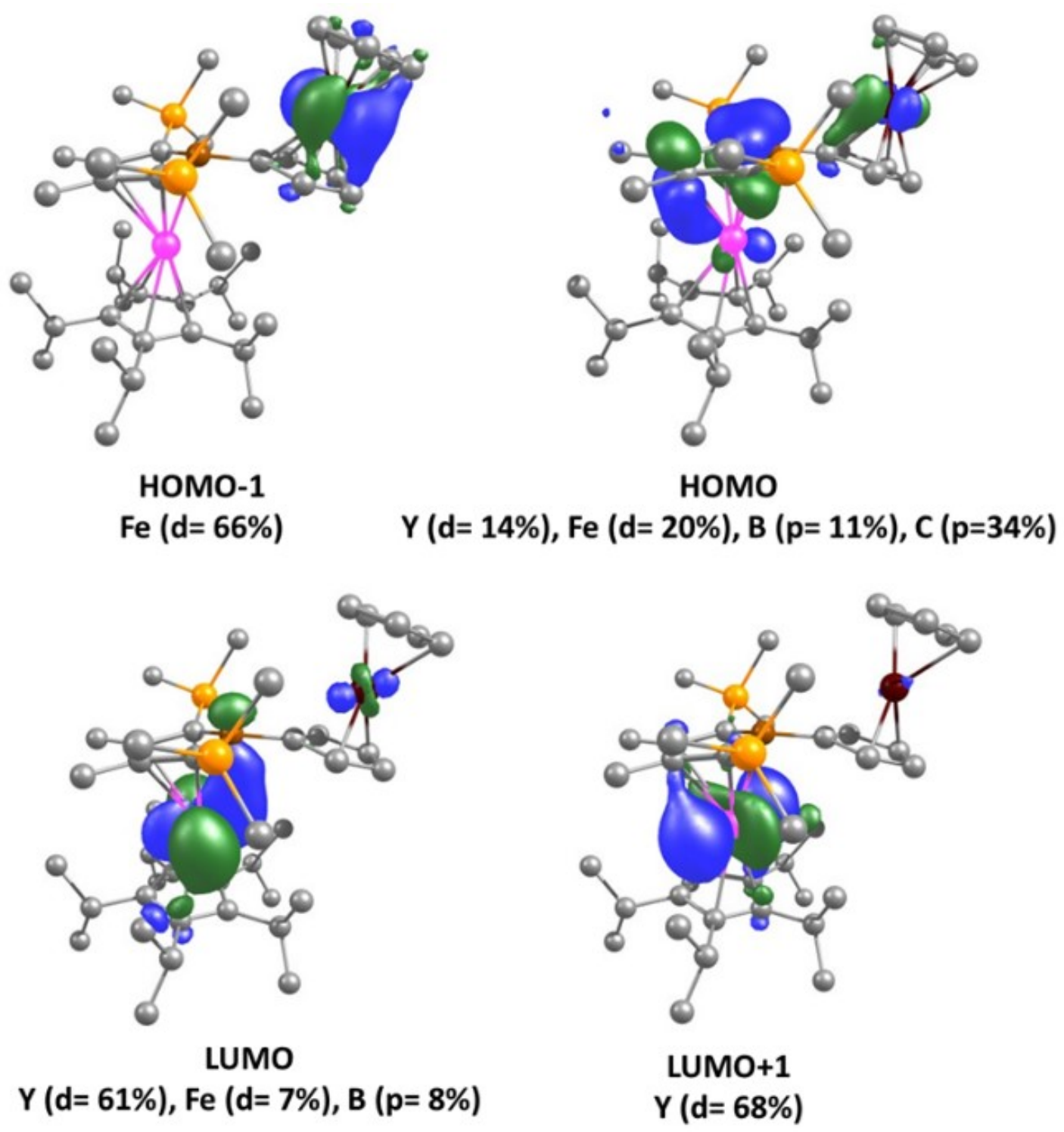

Figure 89. DFT calculated frontier molecular orbital and orbital composition (only from Y, Fe and borole ligand) for **3-Y** (iso value = 0.048).

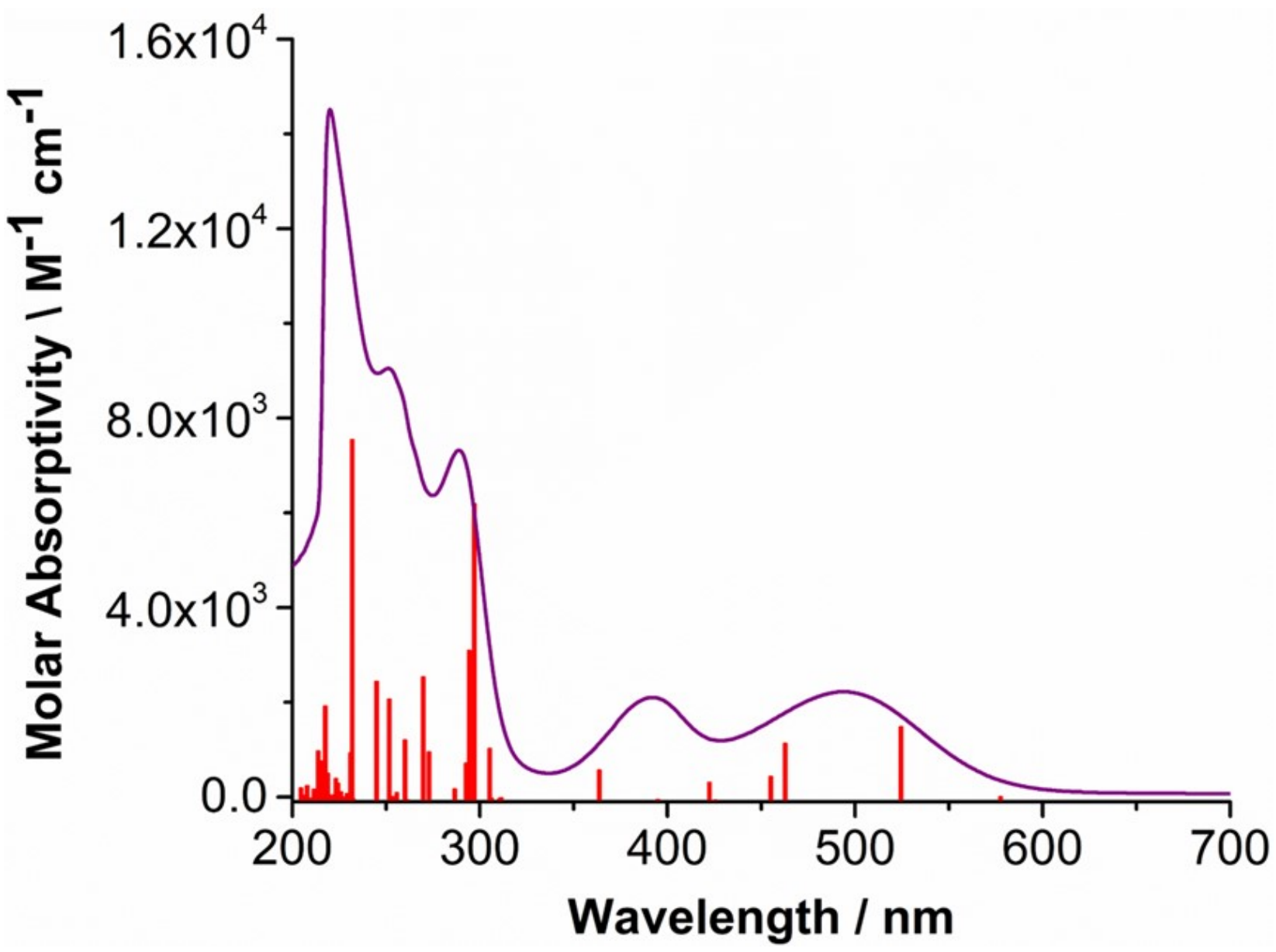

Figure S90. TD-DFT calculated UV-vis spectra for compound **1** using TPSSh/def2-TZVP/def2-SVP level of theory.

Table S11. Computed major excitation wavelengths (λ) and oscillator strengths (*f*) in length representation for **1**.

**λ / nm**	***f***	**Dominant contribution**		
		**Occupied**	**Virtual**	**%Weight**
578	0.00135	111 (HOMO)	112 (LUMO)	85.6
525	0.02893	110 (HOMO-1)	112 (LUMO)	80.2
463	0.02232	109 (HOMO-2)	112 (LUMO)	89.7
455	0.00934	108 (HOMO-3)	112 (LUMO)	74.0
423	0.00702	111 (HOMO)<br>110 (HOMO-1)	114 (LUMO+2)<br>113 (LUMO+1)	42.0<br>38.3
364	0.011904	108 (HOMO-3)<br>108 (HOMO-3)	112 (LUMO)<br>113 (LUMO+1)	21.6<br>42.0
306	0.02030	105 (HOMO-6)<br>107 (HOMO-4)	112 (LUMO)<br>112 (LUMO)	43.5<br>40.0
297	0.11665	106 (HOMO-5)<br>107 (HOMO-4)<br>109 (HOMO-2)	112 (LUMO)<br>112 (LUMO)<br>113 (LUMO+1)	25.4<br>27.4<br>23.8
270	0.048509	102 (HOMO-9)<br>103 (HOMO-8)	112 (LUMO)<br>112 (LUMO)	57.1<br>31.4
245	0.046674	98 (HOMO-13)	112 (LUMO)	93.0
232	0.141805	109 (HOMO-2)	115 (LUMO+3)	73.4

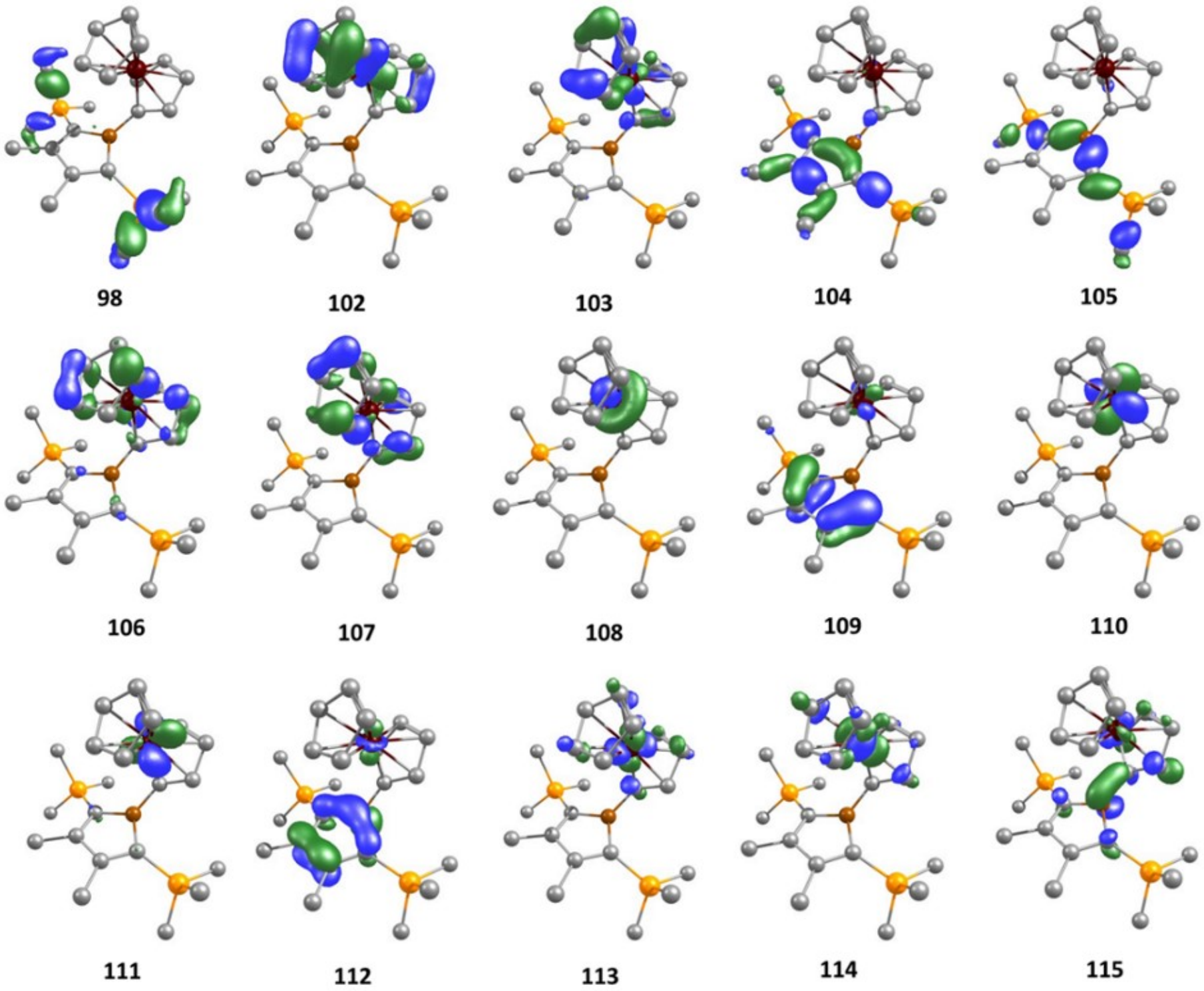

Figure S91. Molecular orbitals for **1** using TPSSh/def2-TZVP/def2-SVP level of theory (iso value = 0.07).

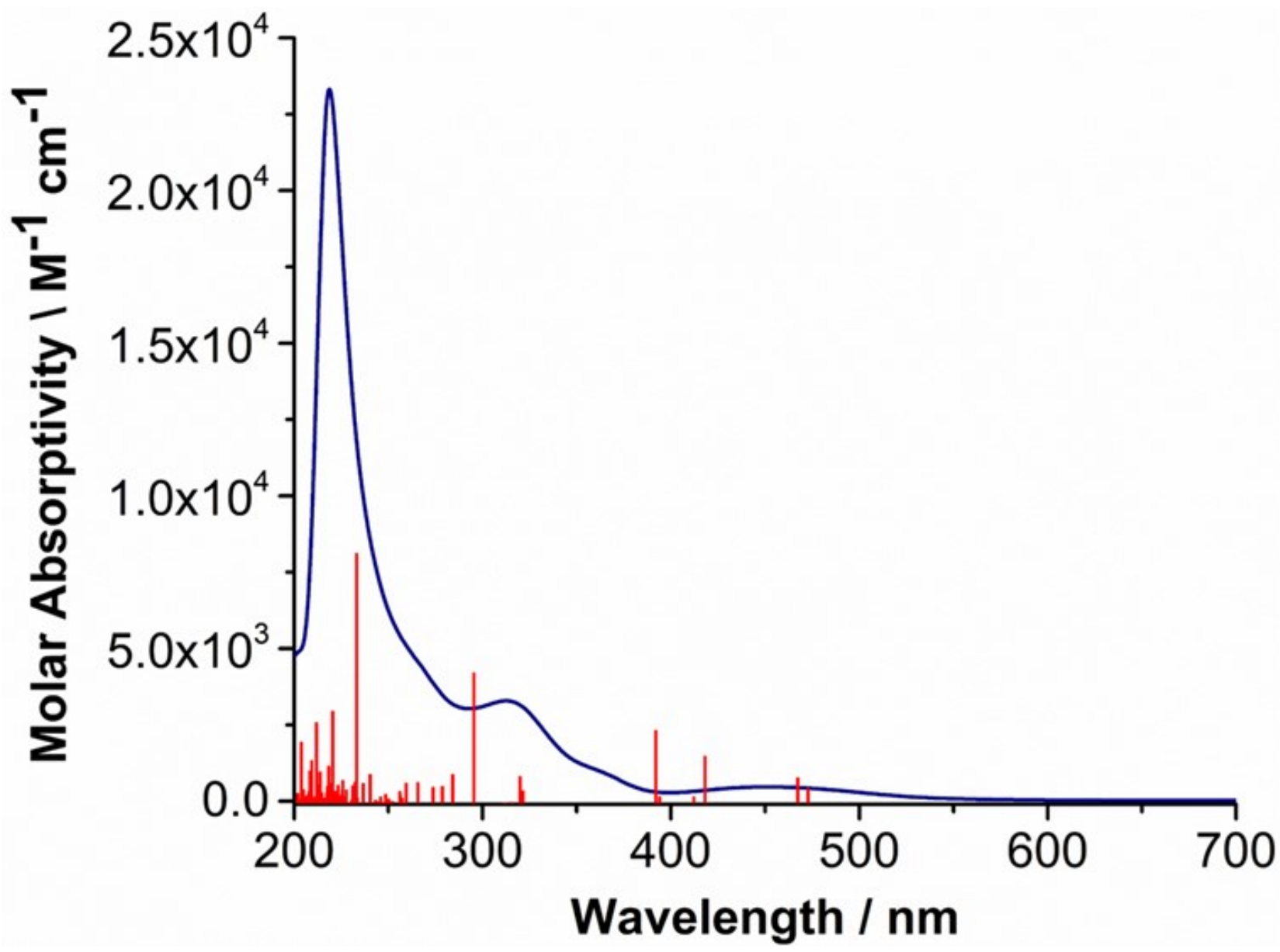

Figure S92. TD-DFT calculated UV-vis spectra for **2** using TPSSh/def2-TZVP/def2-SVP level of theory.

Table S12. Computed major excitation wavelengths (λ) and oscillator strengths (f) in length representation for **2**.

λ / nm	*f*	Dominant contribution		
		**Occupied**	**Virtual**	**%Weight**
474	0.01328	177 (HOMO)	179 (LUMO+1)	20.8
		177 (HOMO)	178 (LUMO)	17.1
		175 (HOMO-2)	179 (LUMO+1)	18.2
468	0.02303	177 (HOMO)	179 (LUMO+1)	14.2
		177 (HOMO)	178 (LUMO)	18.2
		175 (HOMO-2)	178 (LUMO)	29.2
		174 (HOMO-3)	179 (LUMO+1)	21.3
419	0.04271	173 (HOMO-4)	178 (LUMO)	29.4
		175 (HOMO-2)	179 (LUMO+1)	26.3
		177 (HOMO)	178 (LUMO)	19.8
394	0.06635	173 (HOMO-4)	178 (LUMO)	31.0
		174 (HOMO-3)	178 (LUMO)	21.6
322	0.02422	177 (HOMO)	180 (LUMO+2)	76.4
298	0.11894	177 (HOMO)	181 (LUMO+3)	91.6
287	0.02620	177 (HOMO)	182 (LUMO+4)	94.5
268	0.01867	177 (HOMO)	185 (LUMO+7)	87.8
243	0.02626	177 (HOMO)	192 (LUMO+14)	78.3
236	0.228006	176 (HOMO-1)	181 (LUMO+3)	25.5
		177 (HOMO)	192 (LUMO+14)	10.2
		177 (HOMO)	194 (LUMO+16)	8.3
		177 (HOMO)	195 (LUMO+17)	7.0
223	0.083860	177 (HOMO)	196 (LUMO+18)	67.7
		177 (HOMO)	198 (LUMO+20)	5.5

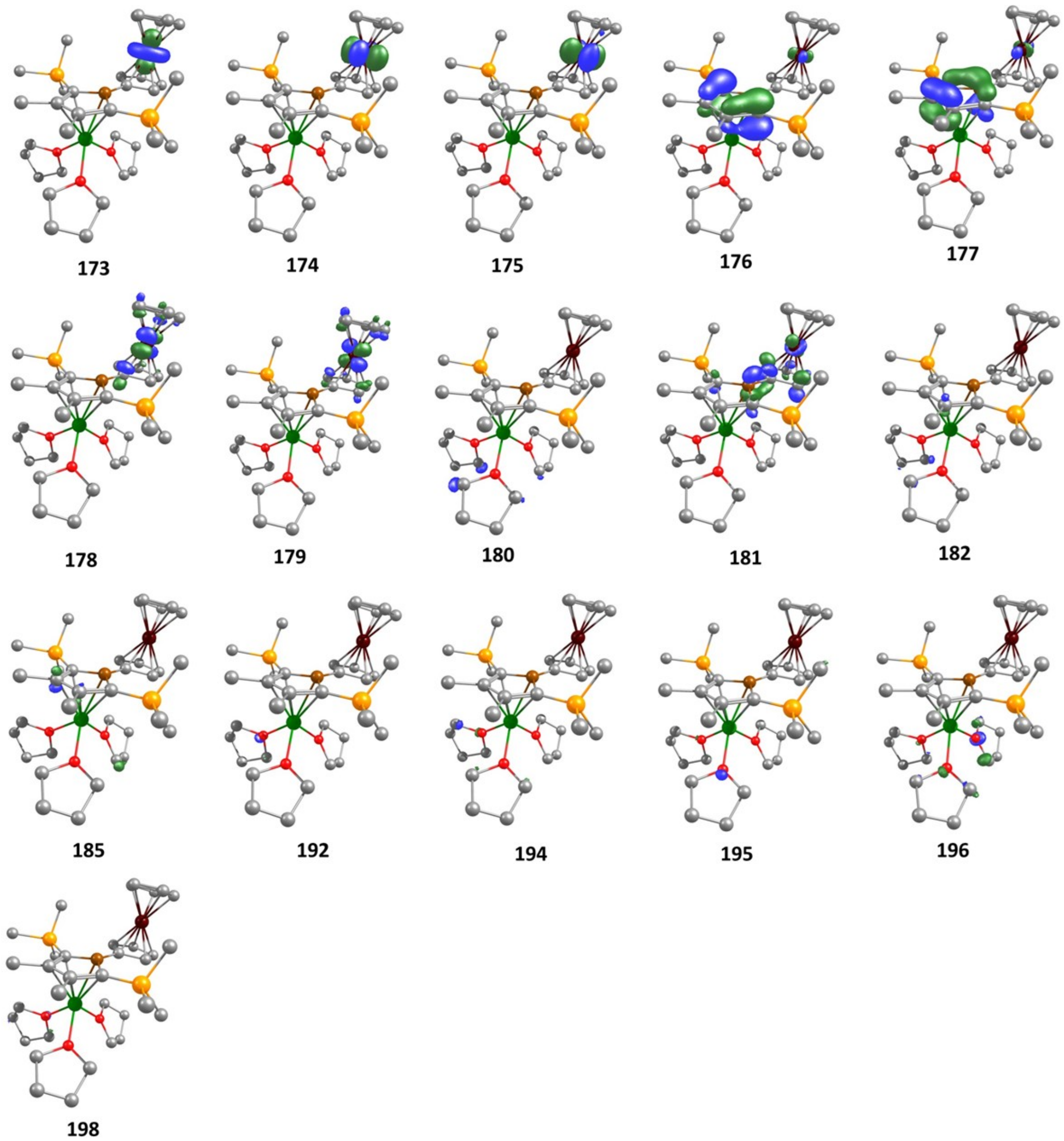

Figure S93. Molecular orbitals for **2** using TPSSh/def2-TZVP/def2-SVP level of theory (iso value = 0.07).

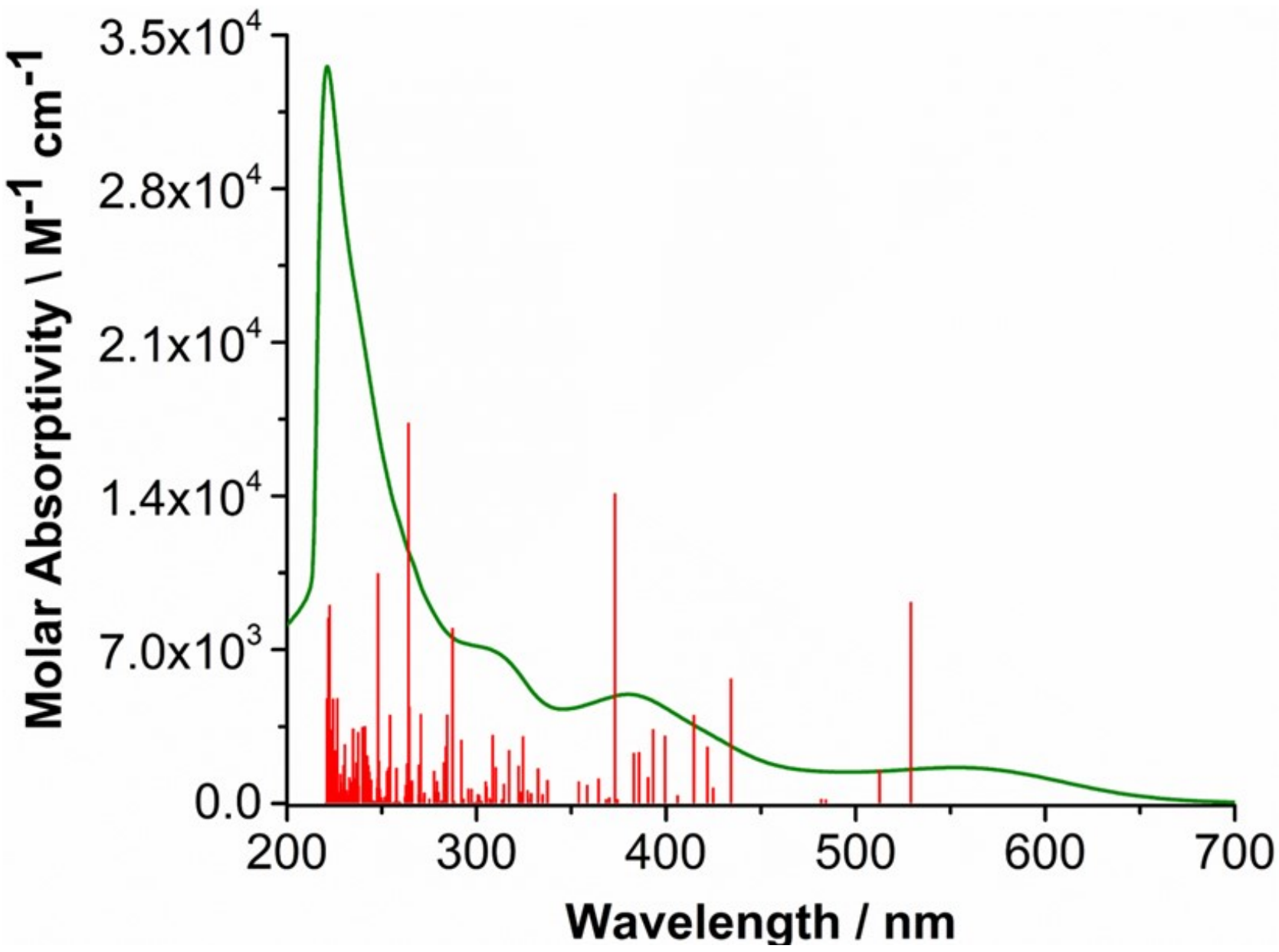

Figure S94. TD-DFT calculated UV-vis spectra for **3-La** using TPSSh/SARC2-DKH-QZVP/def2-TZVP/def2-SVP level of theory.

Table S13. Computed major excitation wavelengths (λ) and oscillator strengths (*f*) in length representation for **3-La**.

λ / nm	*f*	Dominant contribution		
		**Occupied**	**Virtual**	**%Weight**
530	0.052045	217 (HOMO)	218 (LUMO)	87.8
512	0.008559	217 (HOMO)	219 (LUMO+1)	85.6
434	0.032153	217 (HOMO)<br>213 (HOMO-4)	220 (LUMO+2)<br>220 (LUMO+2)	31.5<br>16.2
414	0.022707	217 (HOMO)<br>217 (HOMO)	220 (LUMO+2)<br>221 (LUMO+3)	24.1<br>30.5
393	0.019080	217 (HOMO)<br>217 (HOMO)	223 (LUMO+5)<br>226 (LUMO+8)	55.6<br>17.8
373	0.080253	214 (HOMO-3)<br>216 (HOMO-1)	219 (LUMO+1)<br>219 (LUMO+1)	39.2<br>15.0
324	0.017220	217 (HOMO)<br>214 (HOMO-3)	229 (LUMO+11)<br>221 (LUMO+3)	50.2<br>11.1
308	0.017502	211 (HOMO-6)<br>214 (HOMO-3)	219 (LUMO+1)<br>223 (LUMO+5)	63.3<br>15.0
287	0.0453123	212 (HOMO-5)<br>212 (HOMO-5)	220 (LUMO+2)<br>221 (LUMO+3)	48.9<br>29.0
264	0.098485	217 (HOMO)<br>211 (HOMO-6)	234 (LUMO+16)<br>222 (LUMO+4)	47.2<br>13.5
248	0.059512	209 (HOMO-8)<br>215 (HOMO-2)	219 (LUMO+1)<br>232 (LUMO+14)	60.1<br>9.4
222	0.051273	208 (HOMO-9)<br>217 (HOMO)<br>216 (HOMO-1)	222 (LUMO+4)<br>243 (LUMO+25)<br>235 (LUMO+17)	14.0<br>14.4<br>9.62

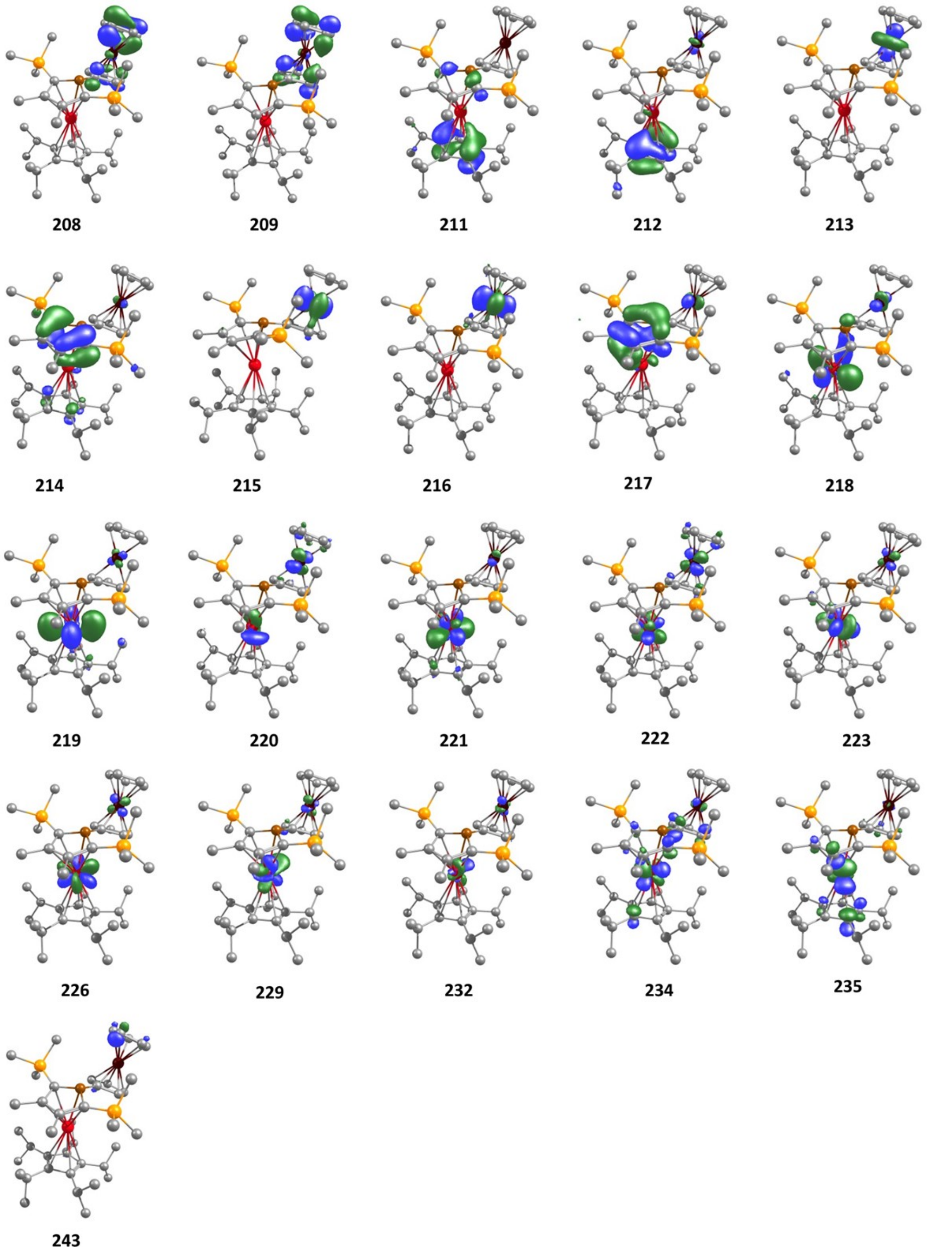

Figure S95. Molecular orbitals for **3-La** using TPSSh/SARC2-DKH-QZVP/def2-TZVP/def2-SVP level of theory (iso value = 0.057).

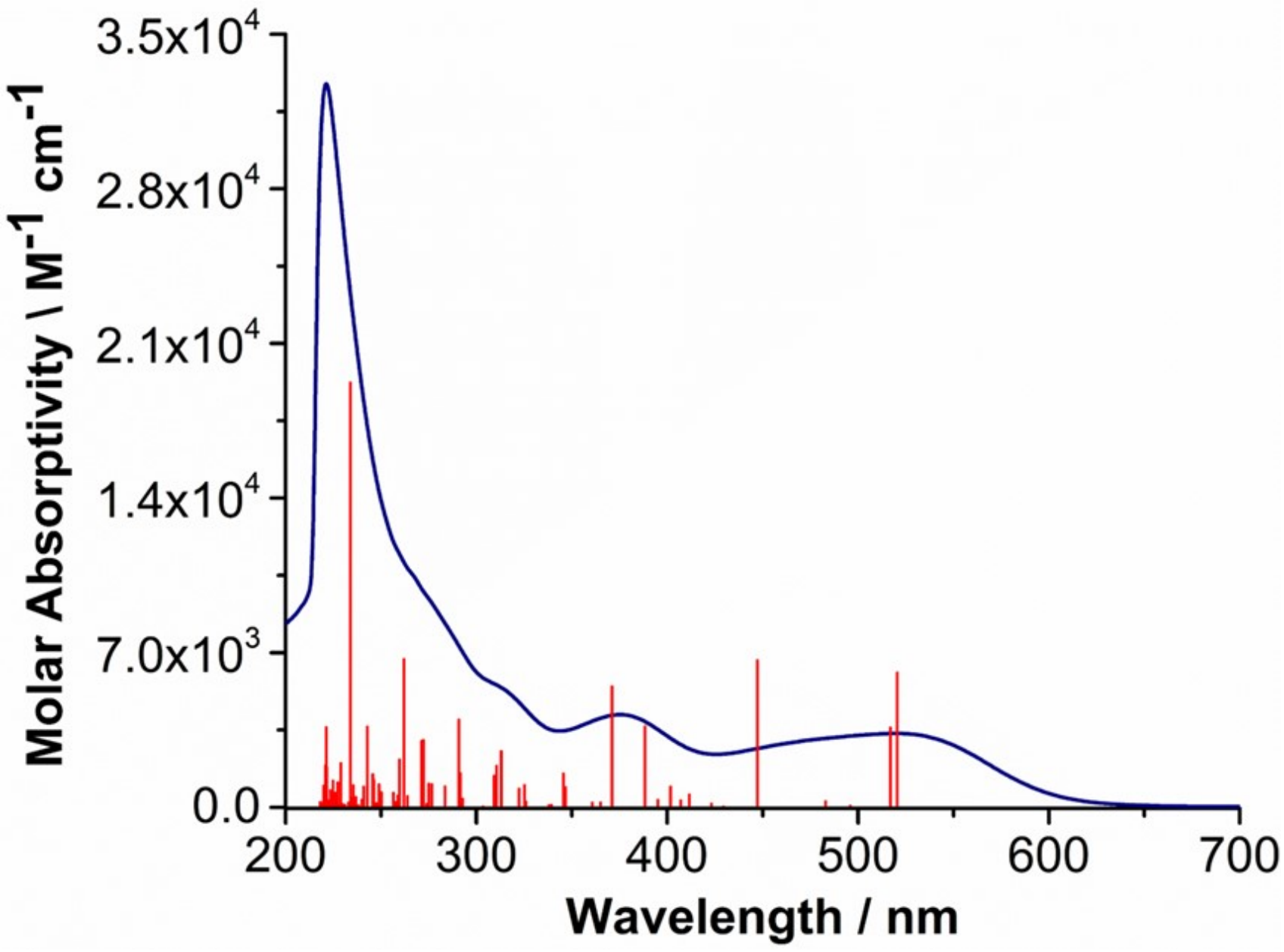

Figure S96. TD-DFT calculated UV-vis spectra for **3-Y** using TPSSh/def2-TZVP/def2-SVP level of theory.

Table S14. Computed major excitation wavelengths (*l*) and oscillator strengths (*f*) in length representation for **3-Y**.

**λ / nm**	***f***	**Dominant contribution**		
		**Occupied**	**Virtual**	**%Weight**
521	0.069231	208 (HOMO)<br>208 (HOMO)	209 (LUMO)<br>210 (LUMO+1)	71.1<br>12.8
517	0.040862	208 (HOMO)<br>208 (HOMO)	210 (LUMO+1)<br>209 (LUMO)	79.6<br>9.6
447	0.075694	208 (HOMO)<br>206 (HOMO-2)	211 (LUMO+2)<br>209 (LUMO)	56.8<br>20.1
388	0.041519	205 (HOMO-3)<br>206 (HOMO-2)	209 (LUMO)<br>210 (LUMO+1)	76.1<br>4.7
371	0.062087	205 (HOMO-3)	210 (LUMO+1)	89.1
347	0.11894	205 (HOMO-3)<br>204 (HOMO-4)<br>208 (HOMO)	211 (LUMO+2)<br>209 (LUMO)<br>213 (LUMO+4)	33.3<br>29.2<br>20.6
313	0.02871	203 (HOMO-5)<br>202 (HOMO-6)<br>202 (HOMO-6)	210 (LUMO+1)<br>209 (LUMO)<br>210 (LUMO+1)	57.5<br>20.6<br>12.5
291	0.04504	203 (HOMO-5)<br>202 (HOMO-6)	211 (LUMO+2)<br>211 (LUMO+2)	52.6<br>37.0
273	0.03438	208 (HOMO)<br>205 (HOMO-3)<br>200 (HOMO-8)	214 (LUMO+5)<br>213 (LUMO+4)<br>209 (LUMO)	30.9<br>24.6<br>27.0
262	0.07627	199 (HOMO-9)<br>200 (HOMO-8)	209 (LUMO)<br>210 (LUMO+1)	69.9<br>22.5
243	0.04132	199 (HOMO-9)	211 (LUMO+2)	83.0
234	0.118981	208 (HOMO)	220 (LUMO+11)	66.6

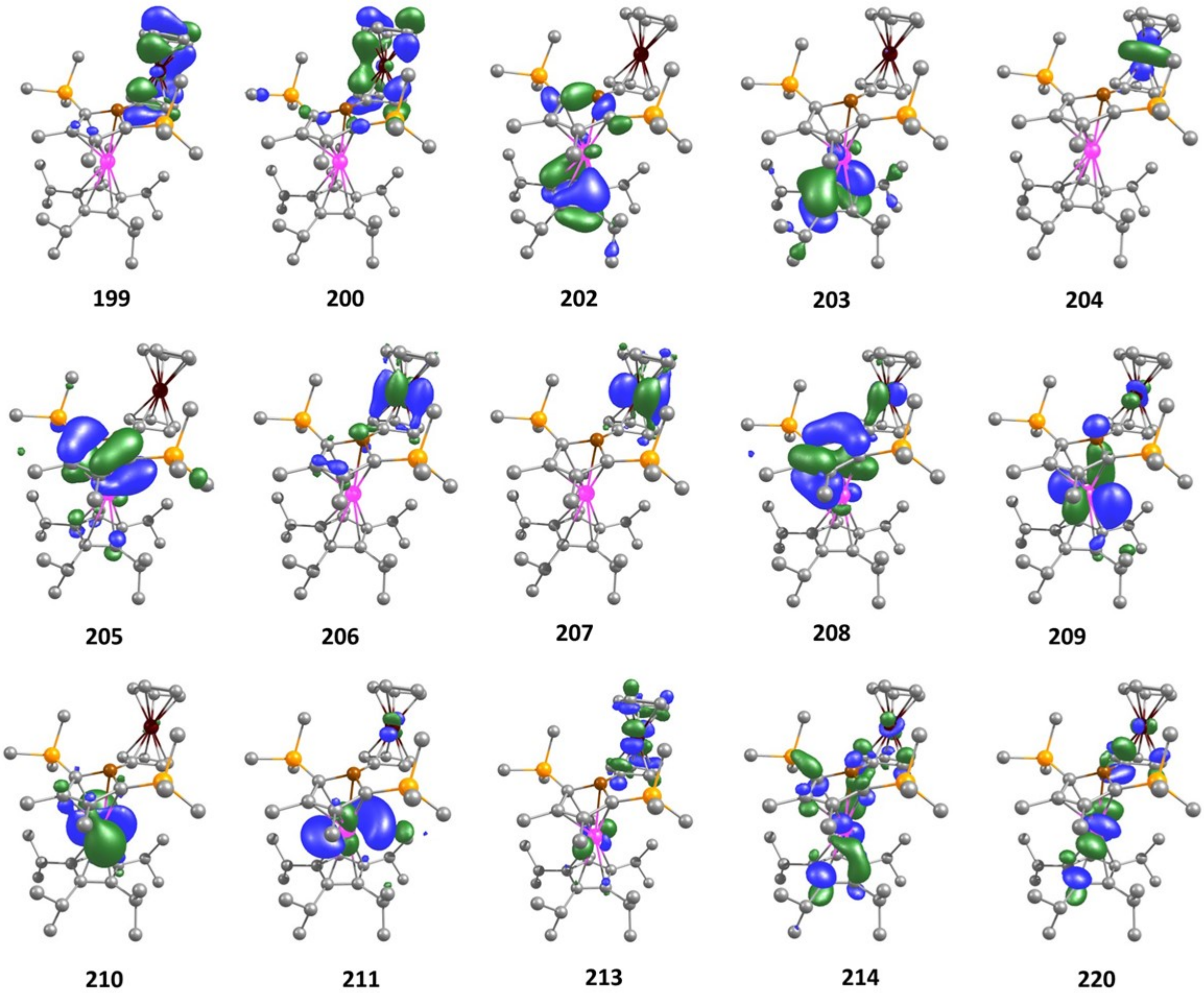

Figure S97. Molecular orbitals for **3-Y** using TPSSh/def2-TZVP/def2-SVP level of theory (iso value = 0.048).

2. CASSCF-SO Calculations:

Complete-active-space self-consistent field (CASSCF) calculations on mononuclear compound **3-Dy** (see Figure S92 for the calculated complete structure) on the basis of single-crystal X-ray determined geometry have been carried out with OpenMolcas program package.[26]

The basis sets for all atoms are atomic natural orbitals from the ANO-RCC library: ANO-RCC-VTZP for Dy(III); VTZ for close N and C; VDZ for other distant atoms. The calculations employed the second order Douglas-Kroll-Hess Hamiltonian, where scalar relativistic contractions were taken into account in the basis set. And then, the spin-orbit couplings were handled separately in the restricted active space state interaction (RASSI-SO) procedure.[27, 28] Active electrons in 7 active orbitals include all *f* electrons (CAS (9 in 7) in the CASSCF calculation. To exclude all the doubts, we calculated all the roots in the active space. We have mixed the maximum number of spin-free state which was possible with our hardware (all from 21 sextets, 128 from 224 quadruplets, 130 from 490 doublets). SINGLE_ANISO[29-31] program was used to obtain the energy levels, *g* tensors, magnetic axes, *et al.* based on the above CASSCF/RASSI-SO calculations.

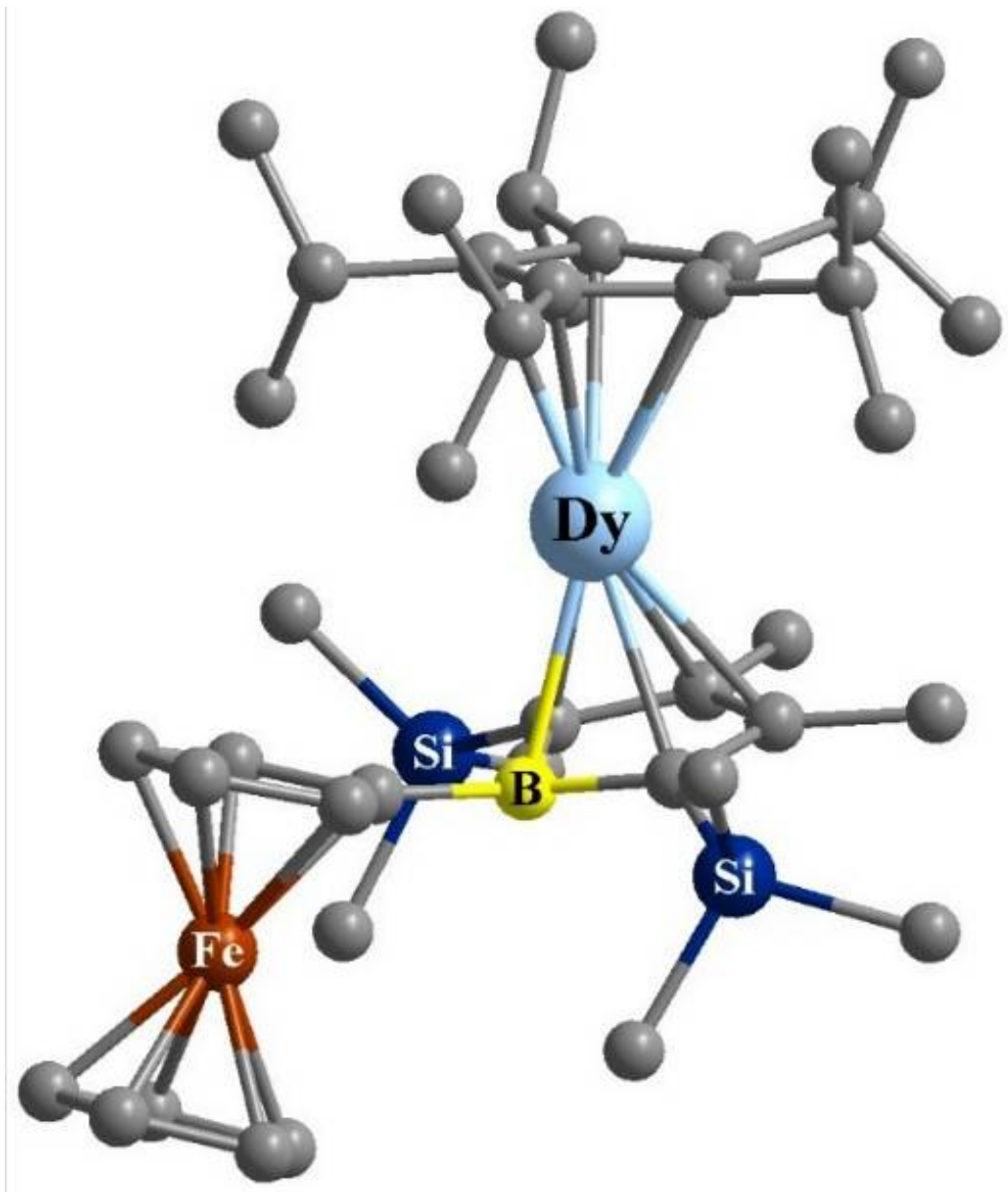

Figure S98. Calculated complete structures of compound **3-Dy**; H atoms are omitted for clarify.

Table S15. Calculated energy levels (cm^{-1}), ***g*** (g_x, g_y, g_z) tensors and predominant m_J values of the lowest eight Kramers doublets (KDs) of compound **3-Dy** using CASSCF/RASSI-SO with OpenMolcas.

KDs	**3-Dy**		
	E	***g***	m_J
1	0.0	0.000<br>0.000<br>19.889	±15/2
2	411.5	0.000<br>0.000<br>17.088	±13/2
3	647.3	0.000<br>0.001<br>14.432	±11/2
4	814.8	0.011<br>0.015<br>11.807	±9/2
5	964.7	0.151<br>0.178<br>9.170	±7/2
6	1101.6	3.133<br>4.056<br>6.214	±5/2
7	1215.4	2.228<br>5.438<br>10.721	±3/2
8	1359.2	0.272<br>0.878<br>17.963	±1/2

Table S16. Wave functions with definite projection of the total moment | m_J > for the lowest eight KDs for compound **3-Dy** using CASSCF/RASSI-SO with OpenMolcas.

	E/cm^{-1}	wave functions
**3-Dy**	0.0	99.8%|±15/2>
	411.5	98.9%|±13/2>
	647.3	96.5%|±11/2>
	814.8	96.1%|±9/2>
	964.7	97.5%|±7/2>
	1101.6	90.4%|±5/2>+6.6%|±1/2>
	1215.4	74.5%|±3/2>+18.0%|±1/2>+6.5%|±5/2>
	1359.2	74.4%|±1/2>+21.9%|±3/2>+3.0%|±5/2>

Table S17. Calculated weight of individual crystal-field parameters *B(k,q)* for **3-Dy**.

k	*q*	*B(k,q)*	Weight	*k*	*q*	*B(k,q)*	Weight
2	–2	2.51×10^{-3}	0.02%	6	–6	-2.91×10^{-7}	0.04%
	–1	-2.96×10^{-1}	2.45%		–5	4.14×10^{-6}	0.58%
	0	-7.33×10^{0}	60.64%		–4	-1.35×10^{-7}	0.02%
	1	7.81×10^{-3}	0.06%		–3	-3.37×10^{-5}	4.74%
	2	7.94×10^{-1}	6.57%		–2	7.82×10^{-7}	0.11%
4	–4	3.13×10^{-5}	0.05%		–1	2.25×10^{-5}	3.17%
	–3	1.81×10^{-3}	2.72%		0	-3.86×10^{-5}	5.43%
	–2	7.63×10^{-5}	0.11%		1	5.43×10^{-7}	0.08%
	–1	-6.60×10^{-4}	0.99%		2	3.42×10^{-5}	4.81%
	0	-5.73×10^{-4}	0.86%		3	4.14×10^{-7}	0.06%
	1	-7.19×10^{-5}	0.11%		4	4.64×10^{-6}	0.65%
	2	-1.74×10^{-3}	2.62%		5	1.11×10^{-6}	0.16%
	3	1.26×10^{-4}	0.19%		6	-3.84×10^{-7}	0.05%
	4	-3.24×10^{-4}	0.49%				

References

1. F.-S. Guo, B. M. Day, Y.-C. Chen, M.-L. Tong, A. Mansikkamäki and R. A. Layfield, *Science*, 2018, **362**, 1400-1403.
2. M. Liu, Y.-C. Chen, A. Mondal, H. Wang, M.-L. Tong, R. A. Layfield and F.-S. Guo, *J. Am. Chem. Soc.*, 2025, **147**, 11359-11367.
3. H. Braunschweig, C. W. Chiu, D. Gamon, M. Kaupp, I. Krummenacher, T. Kupfer, R. Müller and K. Radacki, *Chem. Eur. J.*, 2012, **18**, 11732-11746.
4. W. J. Evans, S. A. Kozimor, J. W. Ziller and N. Kaltsoyannis, *J. Am. Chem. Soc.*, 2004, **126**, 14533-14547.
5. O. V. Dolomanov, L. J. Bourhis, R. J. Gildea, J. A. K. Howard and H. Puschmann, *J. Appl. Crystallogr.*, 2009, **42**, 339-341.
6. G. Sheldrick, *Acta Crystallographica Section A*, 2008, **64**, 112-122.
7. G. Sheldrick, *Acta Crystallographica Section C*, 2015, **71**, 3-8.
8. G. A. Bain and J. F. Berry, *J. Chem. Educ.*, 2008, **85**, 532.
9. F. Neese, *WIREs Computational Molecular Science*, 2022, **12**, e1606.
10. F. Neese, F. Wennmohs, U. Becker and C. Riplinger, *J. Chem. Phys.*, 2020, **152**.
11. J. P. Perdew, K. Burke and M. Ernzerhof, *Phys. Rev. Lett.*, 1996, **77**, 3865-3868.
12. J. Tao, J. P. Perdew, V. N. Staroverov and G. E. Scuseria, *Phys. Rev. Lett.*, 2003, **91**, 146401.
13. F. Neese and G. Olbrich, *Chem. Phys. Lett.*, 2002, **362**, 170-178.
14. D. Aravena, F. Neese and D. A. Pantazis, *Journal of Chemical Theory and Computation*, 2016, **12**, 1148-1156.
15. J. Chmela and M. E. Harding, *Mol. Phys.*, 2018, **116**, 1523-1538.
16. J. D. Rolfes, F. Neese and D. A. Pantazis, *J. Comput. Chem.*, 2020, **41**, 1842-1849.
17. R. Izsák and F. Neese, *J. Chem. Phys.*, 2011, **135**.
18. F. Neese, F. Wennmohs, A. Hansen and U. Becker, *Chem. Phys.*, 2009, **356**, 98-109.
19. M. Garcia-Ratés and F. Neese, *J. Comput. Chem.*, 2020, **41**, 922-939.
20. P. v. R. Schleyer, C. Maerker, A. Dransfeld, H. Jiao and N. J. R. van Eikema Hommes, *J. Am. Chem. Soc.*, 1996, **118**, 6317-6318.
21. S. Klod and E. Kleinpeter, *J. Chem. Soc. Perk. Trans.2*, 2001, DOI: 10.1039/B008809O, 1893-1898.
22. M. J. Frisch, G. W. Trucks, H. B. Schlegel, G. E. Scuseria, M. A. Robb, J. R. Cheeseman, G. Scalmani, V. Barone, G. A. Petersson, H. Nakatsuji, X. Li, M. Caricato, A. V. Marenich, J. Bloino, B. G. Janesko, R. Gomperts, B. Mennucci, H. P. Hratchian, J. V. Ortiz, A. F. Izmaylov, J. L. Sonnenberg, Williams, F. Ding, F. Lipparini, F. Egidi, J. Goings, B. Peng, A. Petrone, T. Henderson, D. Ranasinghe, V. G. Zakrzewski, J. Gao, N. Rega, G. Zheng, W. Liang, M. Hada, M. Ehara, K. Toyota, R. Fukuda, J. Hasegawa, M. Ishida, T. Nakajima, Y. Honda, O. Kitao, H. Nakai, T. Vreven, K. Throssell, J. A. Montgomery Jr., J. E. Peralta, F. Ogliaro, M. J. Bearpark, J. J. Heyd, E. N. Brothers, K. N. Kudin, V. N. Staroverov, T. A. Keith, R. Kobayashi, J. Normand, K. Raghavachari, A. P. Rendell, J. C. Burant, S. S. Iyengar, J. Tomasi, M. Cossi, J. M. Millam, M. Klene, C. Adamo, R. Cammi, J. W. Ochterski, R. L. Martin, K. Morokuma, O. Farkas, J. B. Foresman and D. J. Fox, *Journal*, 2016.
23. A. D. Becke, *J. Chem. Phys.*, 1993, **98**, 5648-5652.
24. C. Lee, W. Yang and R. G. Parr, *Physical Review B*, 1988, **37**, 785-789.
25. B. Miehlich, A. Savin, H. Stoll and H. Preuss, *Chem. Phys. Lett.*, 1989, **157**, 200-206.
26. F. Aquilante, J. Autschbach, R. K. Carlson, L. F. Chibotaru, M. G. Delcey, L. De Vico, I. Fdez. Galván, N. Ferré, L. M. Frutos, L. Gagliardi, M. Garavelli, A. Giussani, C. E. Hoyer, G. Li Manni, H. Lischka, D. Ma, P. Å. Malmqvist, T. Müller, A. Nenov, M. Olivucci, T. B. Pedersen, D. Peng, F. Plasser, B. Pritchard, M. Reiher, I. Rivalta, I. Schapiro, J. Segarra-Martí, M. Stenrup, D. G. Truhlar, L. Ungur, A. Valentini, S. Vancoillie, V. Veryazov, V. P. Vysotskiy, O. Weingart, F. Zapata and R. Lindh, *J. Comput. Chem.*, 2016, **37**, 506-541.
27. P. Å. Malmqvist, B. O. Roos and B. Schimmelpfennig, *Chem. Phys. Lett.*, 2002, **357**, 230-240.
28. B. A. Heß, C. M. Marian, U. Wahlgren and O. Gropen, *Chem. Phys. Lett.*, 1996, **251**, 365-371.
29. L. F. Chibotaru, L. Ungur and A. Soncini, *Angew. Chem. Int. Ed.*, 2008, **47**, 4126-4129.
30. L. Ungur, W. Van den Heuvel and L. F. Chibotaru, *New J. Chem.*, 2009, **33**, 1224-1230.
31. L. F. Chibotaru, L. Ungur, C. Aronica, H. Elmoll, G. Pilet and D. Luneau, *J. Am. Chem. Soc.*, 2008, **130**, 12445-12455.
